# Regioselective Synthesis of New Family of 2-Substituted 1,2,3-Triazoles and Study of Their Fluorescent Properties

**DOI:** 10.3390/molecules28124822

**Published:** 2023-06-16

**Authors:** Vasiliy M. Muzalevskiy, Zoia A. Sizova, Valentine G. Nenajdenko

**Affiliations:** Department of Chemistry, Lomonosov Moscow State University, 119899 Moscow, Russia

**Keywords:** 1,2,3-triazole, alkylation, arylation, fluorine, CF_3_CO-group

## Abstract

Modification of 5-aryl-4-trifluoroacetyltriazoles at the NH-moiety was investigated. Screening of the alkylation conditions revealed that using Na_2_CO_3_ as a base and DMF as a solvent of 2-substituted triazoles can be preferentially prepared in up to 86% yield. In the best cases, the amount of minor 1-alkyl isomer was less than 6%. S_N_Ar reaction of the 5-aryl-4-trifluoroacetyltriazoles with aryl halides having electron-withdrawing groups led to regiospecific formation of 2-aryltriazoles isolated in good-to-high yields. Chan–Lam reaction of the 5-aryl-4-trifluoroacetyltriazoles with boronic acids afforded 2-aryltriazoles as single isomers in up to 89% yield. The subsequent reaction of the prepared 2-aryltriazoles with primary and secondary amines gave a set of amides of 4-(2,5-diaryltriazolyl)carboxylic acid. The fluorescent properties of the prepared 2-substituted derivatives of triazoles were investigated to demonstrate their utility as new efficient luminophores having more than 60% quantum yields.

## 1. Introduction

Nowadays, chemistry of fluorinated compounds is one of the most rapidly growing areas of modern organic chemistry [1,2,3,4]. Fluorinated compounds are widely used as construction materials, components of liquid crystalline compositions, agrochemicals and pharmaceuticals [5,6,7,8,9,10]. By some estimations, about 20% of currently used drugs [11,12,13,14,15,16,17,18,19] contain at least one fluorine atom [20]. In 2022, approximately 21% (four out of nineteen) of “small-molecule drugs” approved by the FDA are fluorinated compounds [21]. On the other hand, about 59% of all small-molecule drugs have a nitrogen heterocyclic motif [22]. Last year revealed that four out of every five small-molecule drugs approved by the FDA in 2022 (16 out of 19) are representatives of that class [21]. It is no doubt that elaboration of novel pathways to fluorinated nitrogen heterocycles is of great demand [23,24,25,26,27,28,29,30].

The most reliable strategy towards fluorinated compounds is a building block strategy, which uses the assembling of simple fluorinated molecules into more complex structures. Last year, our group was tightly engaged with the elaboration and investigation of novel fluorinated building blocks to prepare various trifluoromethylated heterocycles. Thus, we have proposed convenient syntheses of α-CF_3_-β-aryl enamines [31], α,β-diaryl-CF_3_-enones [32] and CF_3_-ynones [33], which appeared to be versatile CF_3_-building blocks for the synthesis of various fluorinated compounds [34,35]. Recently, we have found that reaction of CF_3_-ynones with sodium azide can be used for directed synthesis of either 5-CF_3_-isoxazoles or previously unknown 4-trifluoroacetyltriazoles [36]. The prepared triazoles are a new class of triazolyl compounds. In this article, we have investigated the reactivity and regioselectivity of 4-trifluoroacetyltriazoles in alkylation and arylation as well as the fluorescent properties of the prepared derivatives.

## 2. Results

**Scheme 1:**
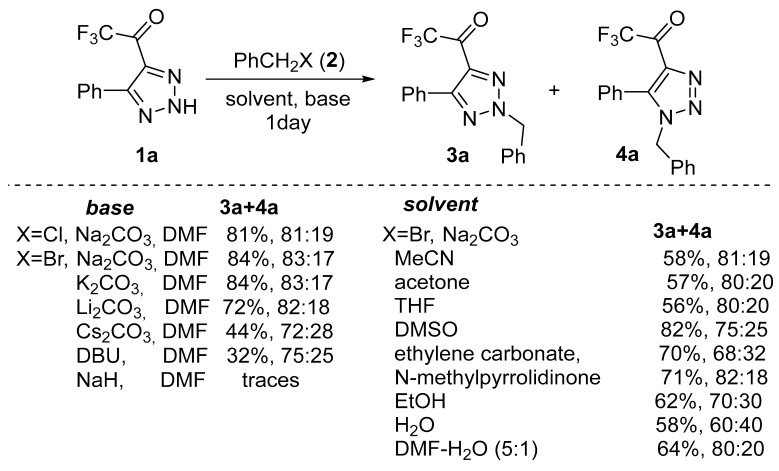
Screening of the reaction conditions for alkylation of triazole **1a** with benzyl bromide and chloride.

As a starting point, we studied alkylation of the model triazole **1a** in DMF by using benzyl chloride and bromide with Na_2_CO_3_ as a base. We found that both reagents afforded benzylated triazoles **3a** and **4a** in high total yield. Moreover, the reaction proceeds regioselectively to form the 2-isomeric triazole **3a** preferentially (Scheme 1).

Next, we tested several bases in the reaction. We found that application of Li_2_CO_3_, Na_2_CO_3_ and K_2_CO_3_ provided similar selectivity and yields. Use of Cs_2_CO_3_ and DBU gave triazoles less selectively and in significantly lower yield. In the case of NaH, alkylated triazoles **3a** and **4a** were not obtained at all, while the starting material was completely consumed (Scheme 1). Recently, we have demonstrated that the trifluoroacetyl group can be transformed into carboxylic or amide function by heating in basic conditions [37]. We believed that a similar transformation of the trifluoroacetyl group takes place in the case of Cs_2_CO_3_, DBU and NaH, leading to formation of a complex reaction mixture. Moreover, Na_2_CO_3_ showed the best results in terms of yield and selectivity and has been chosen as a base for further investigations. Using Na_2_CO_3_, we investigated the influence of the solvent on the reaction. It was found that the reaction can proceed in both protic and aprotic solvents to give *N*-alkylated triazoles in good-to-high yields. The highest yields (84%) and selectivity (only 17% of minor isomer) were observed in polar aprotic solvents (DMF, DMSO). Reaction in polar protic solvents (EtOH, H_2_O) was less selective and produced 30–40% of the isomer **4a**. It should be noted that in all investigated solvents, the yields of triazoles did not drop lower than 57%. Taking into account the obtained results, we chose DMF as a solvent and Na_2_CO_3_ as a base for all subsequent transformations (Scheme 1).

Having in hand the suitable conditions, we performed reactions with a number of alkyl halides 2. It was found that the product distribution is dependent on the activity of the alkylating agent. Thus, the reactions of triazoles **1a,b** with benzyl bromide as well as the reaction of triazole **1a** with 4-nitrobenzyl bromide led to a mixture of isomers **3** and **4** in 81:19 ratio. The reaction of allyl chloride, which compared to benzyl bromide by activity, afforded an 83:17 mixture of isomers. The reaction of MeI (most reactive among non-functionalized alkyl halides) with **1a** led to a mixture of **3e** and **4e** in an 83:17 ratio. The reaction with less reactive aliphatic alkyl bromides proceeds more selectively to give only a 6–9% admixture of minor regioisomer. It should be noted that the reaction outcome is not sensitive to bulkiness of the reagents to give similar results for primary, secondary and cyclic alkyl bromides. However, heating is needed in the case of most bulky *iso*-propyl-, *cyclo*-hexyl bromides and *iso*-butyl chloride. A lower selectivity was observed in the case of the reaction with 1,4-dibromobutane; such a ratio of regioisomers can be explained by statistical factor.

**Scheme 2:**
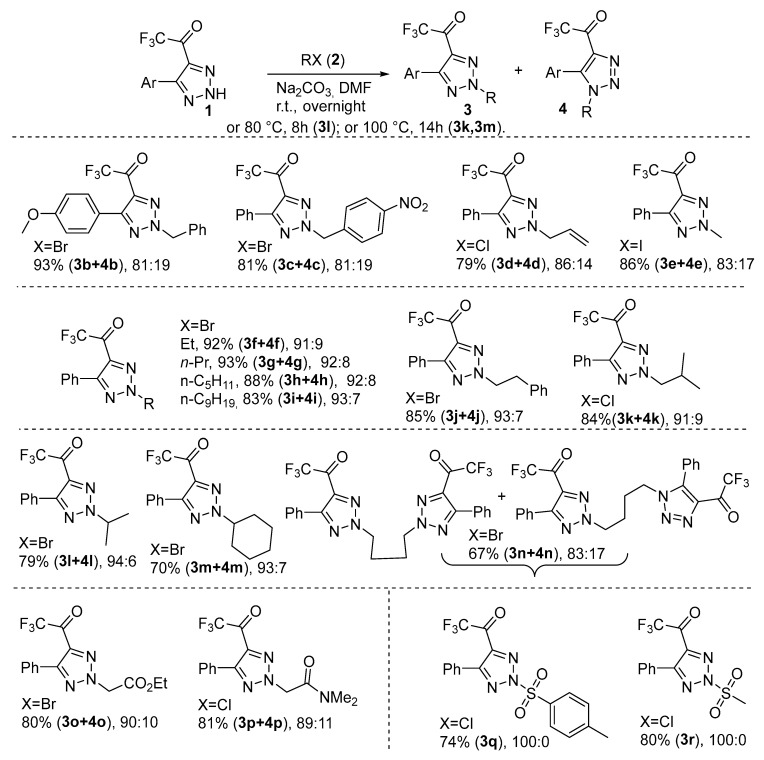
Alkylation and sulfonylation of triazoles **1**.

Another type of the alkylating agents investigated were derivatives of 2-haloacetic acid. The alkylation of triazole **1a** with ethyl bromoacetate and 2-chloro-N,N-dimethylacetamide gave a mixture of isomeric triazoles in almost the same ratio (90:10 and 89:11, correspondingly). Eventually, sulfonylation of **1a** by MsCl and TsCl led to 2-substituted triazoles exclusively (Scheme 2).

**Figure 1:**
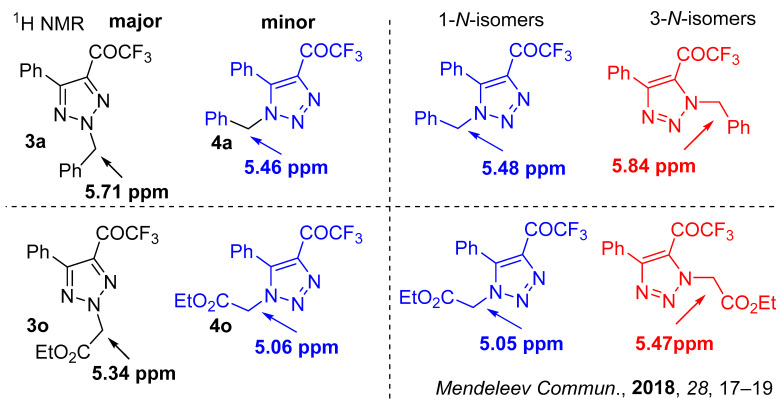
Elucidation of the structure of regioisomers **3** and **4**.

Elucidation of the structure of regioisomers **3** and **4** was carried out by comparison with the literature data. Previously, we have found that 2 + 3 cycloaddition reaction of CF_3_-ynones with alkyl and aryl azides led mostly to 1-substituted triazoles with admixture of 3-substituted triazoles [38]. Careful comparison of CH_2_ signals in ^1^H NMR of triazoles **3** and **4** with those of 1-substituted and 3-substituted isomers showed that CH_2_ signals of minor isomers **4a** and **4o** are in good agreement with CH_2_ signals of 1-alkyl triazole (Figure 1). CH_2_-signals of major isomers of **3a** and **3o** are downfield shifted and differ from the CH_2_ signals of 3-alykyl isomers. Hence, major isomers **3a** and **3o** are 2-substrituted derivatives. It should be noted that substitution at the position 2 in NH-triazoles **1** is favored by both steric and electronic factors. Indeed, positions N-1 and N-3 of triazoles **1** are hindered by the bulky aryl substituent at C-5 and trifluoroacetyl group at C-4 atom of the triazole ring. As a result, the 2-isomer is a major one. In addition, the nucleophilicity of N-3 is diminished by the high electronegativity of the trifluoroacetyl group, which is why 3-N-isomers are not formed at all. It should be noted that major isomer **3** can be easily separated from isomer **4** by column chromatography on silica gel.

**Scheme 3:**
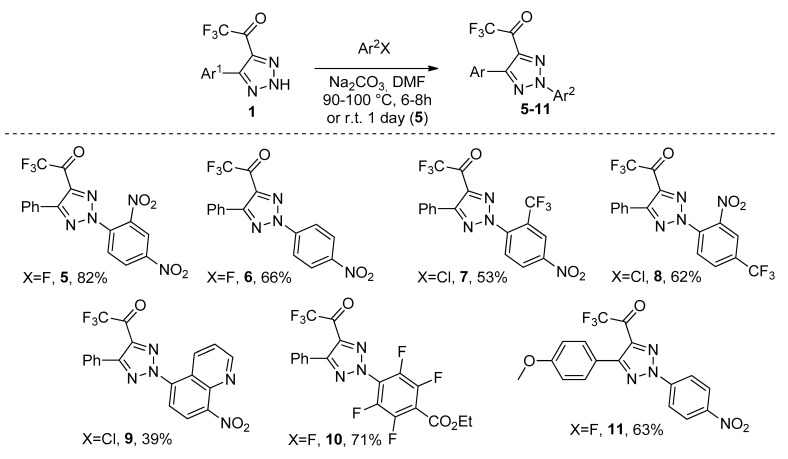
Arylation of triazoles **1** with various aryl halides **5–11**.

Next, we switched our attention to the reaction of triazoles **1a** with aryl halides activated by electron-withdrawing substituents. In contrast to the S_N_2 reaction with alkyl halides **2**, the S_N_Ar reaction with aryl halides proceeds at elevated temperatures (90–100 °C). Only the reaction with highly reactive 1-fluoro-2,4-dinitrobenzene can be performed at room temperature. The arylation is regiospecific to afford only 2-aryl substituted products **5–12**, which is a favorable feature of the arylation. Using this approach, we prepared a set of 2-N arylated triazoles having CF_3_, NO_2_ and CO_2_Et groups in up to 86% yield. In addition, triazole **9** bearing a quinoline moiety was also prepared in moderate yield (Scheme 3).

**Scheme 4:**
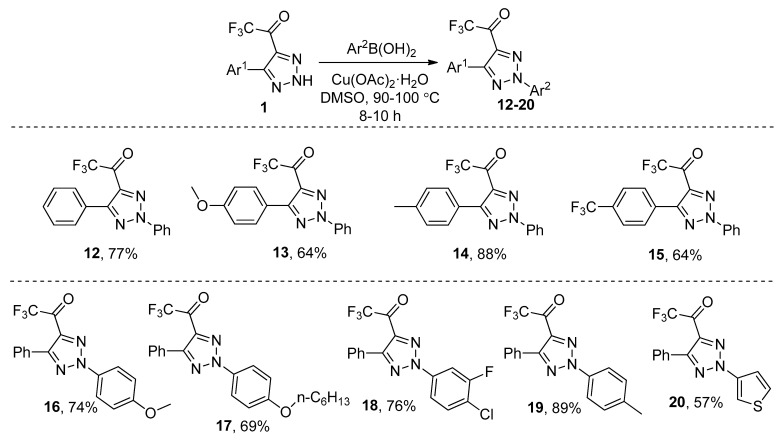
Cham–Lam arylation of triazoles **1**.

In spite of the high utility of the S_N_Ar reaction, obvious restriction of this method is necessary in order to have an activating EWG group in the structure of the aryl halide. Therefore, we also investigated an alternative type of arylation of triazoles **1** in conditions of the Chan–Lam reaction. Carried out in open air (no balloons with oxygen and etc.) reactions of triazoles **1** with boronic acids in DMSO at heating under catalysis with copper (II) acetate afforded 2-N-aryl derivatives **12–20** in 100% regioselectively in up to 89% yields (Scheme 4). It should be noted that no additives of any ligands were needed for successful transformation of triazoles **1**.

**Scheme 5:**
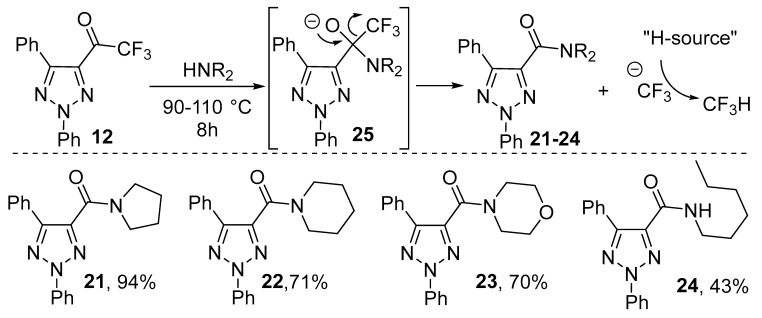
Reactions of triazole **12** with secondary amines.

Recently, we found that N-unsubstituted 4-trifluoroacetyl triazoles react with secondary amines at elevated temperatures to produce corresponding amides as a result of formal substitution of CHF_3_ [37]. The prepared 2-arylated triazoles can be used for the similar transformation as well. We performed the reaction of 2-phenyl substituted triazole **12** with some secondary and primary amines at heating. As a result, a set of amides **21–24** was obtained in good-to-high yields to provide a broad diversity of the synthesized triazole derivatives. Thus, we succeeded in preparing derivatives of pyrrolidine, piperidine, morpholine and *n*-hexylamine (Scheme 5). Taking into account the literature data [37], we proposed a possible mechanism of the reaction. First, the addition of amine to the carbonyl group of **12** led to intermediate **25**. Next, **25** eliminates the trifluoromethyl anion to afford amides **21–24;** protonation of CF_3_-anion gives CF_3_H.

**Figure 2:**
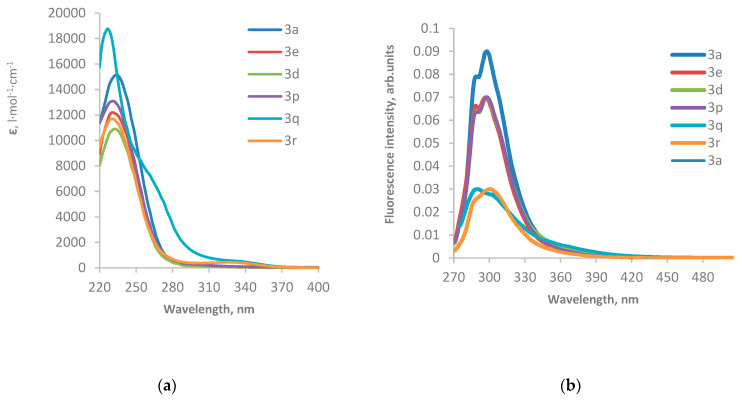
(**a**) Absorption and (**b**) emission spectra of solutions of compounds **3** in methanol.

The 1,2,3-triazole scaffold has been intensively investigated in recent decades, boosted by the discovery of CuAAC-RuAAC reactions (metal-catalyzed alkyne–azide cycloaddition) [39,40]. Many 1,2,3-triazoles have useful practical properties and have found applications as agrochemicals, pigments, metal chelators, photostabilizers and corrosion inhibitors [41]. Many 1,2,3-triazoles are physiologically active compounds and have pharmaceutical and therapeutic applications [42,43,44]. Ion(s) detection capabilities of 1,2,3-triazole ligands using absorption and fluorescence spectroscopy were also reported [45,46,47,48]. Of special interest are 2-Aryl-substituted 1,2,3-triazoles, since these compounds are highly efficient UV/blue-light-emitting fluorophores [49,50]. Therefore, we investigated some photophysical properties of the prepared 2-substituted triazoles. Electronic absorption spectra (c = 10^−5^ M) and fluorescence spectra data were recorded in methanol solutions (c = 10^−6^ M) at room temperature. The quantum yields of fluorescence (φ) were determined by a comparative method using 2-aminopyridine as the standard. The absorption band of compounds **3** bearing alkyl group in the position 2 of triazole has one pronounced peak; however, tosyl-substituted triazole **3q** has several adsorption bands. The adsorption maxima of **3a,d,e,p,r** are in the range of 224–234 nm (Figure 2a). All these derivatives demonstrated light emission in the range 297–303 nm and quantum yields below 10% (Table 1, Figure 2b).

**Figure 3:**
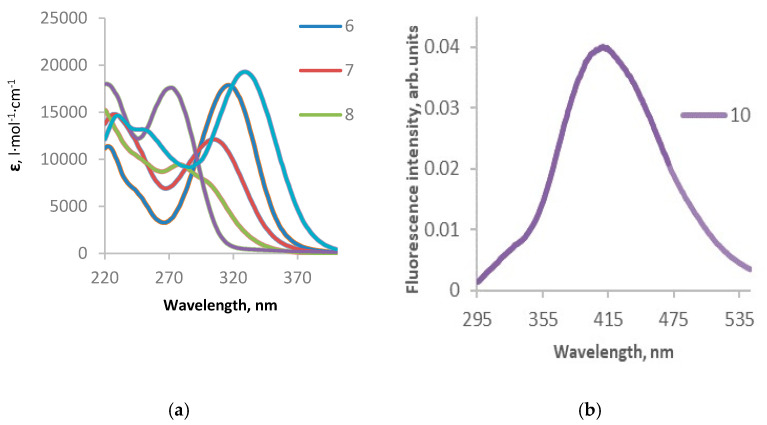
(**a**) Absorption and (**b**) emission spectra of solutions of compounds **6–8,10,11** in methanol.

**Figure 4:**
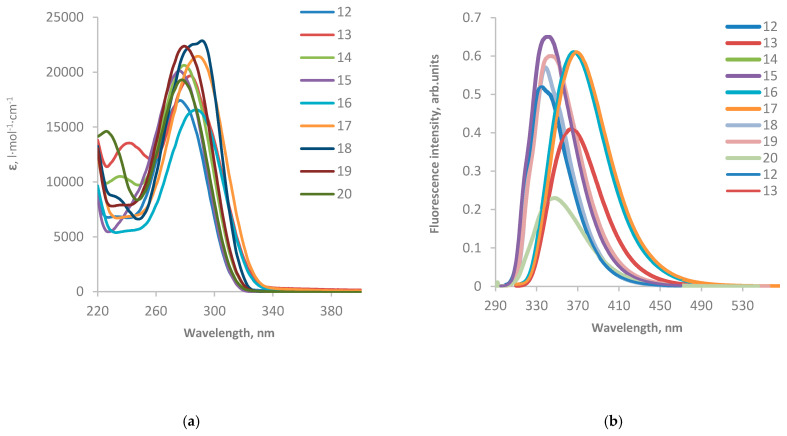
(**a**) Absorption and (**b**) emission spectra of solutions of compounds **12–20** in methanol.

Much more interesting photophysical properties are observed for 2-aryl substituted triazoles **6–24.** Among these types of triazoles, we found highly efficient fluorophores demonstrating emission in the range 335–368 nm and quantum yields up to 65% in methanol solution. However, it was found that the nature of the substituent in the 2-aryl group of triazoles 6 influences dramatically the emission properties (Table 2). For example, compounds **6–8,11** do not fluoresce at all. Complete quenching of fluorescence in these cases can be explained by the presence of the EWG group at the aryl fragment attached to the position 2 of the triazole. In contrast, high quantum yields are observed when the aryl group at the position 2 is electron-rich. The absorption spectra of compounds **6,7,10,11,13,20** have two bands, which is especially pronounced for compounds **7,10.** The introduction of an electron-withdrawing group into the 2-aryl substituent leads to a bathochromic shift of the absorption band. It is most pronounced for compounds **6** and **7** (Figure 3a). The exception is **10**, for which a slight hypsochromic shift is observed. At the same time, despite the low quantum yield, **10** has the largest Stokes shift among the compounds of this series (Figure 3b). Most probably, this is due to the influence of its fluorinated fragment. The introduction of substituents into the *para*-position of 5-aryltriazole does not affect significantly the position of the absorption band, causing only a slight bathochromic shift for **13** (Figure 4a). The quantum yields of **12–20** compounds turned out to be the best among the N-2-aryl-1,2,3-triazoles studied by us and are in the range of 0.23–0.65 (Figure 4b). The highest value for compound **15**, for compounds **14, 16–20,** is about 0.60.

**Figure 5:**
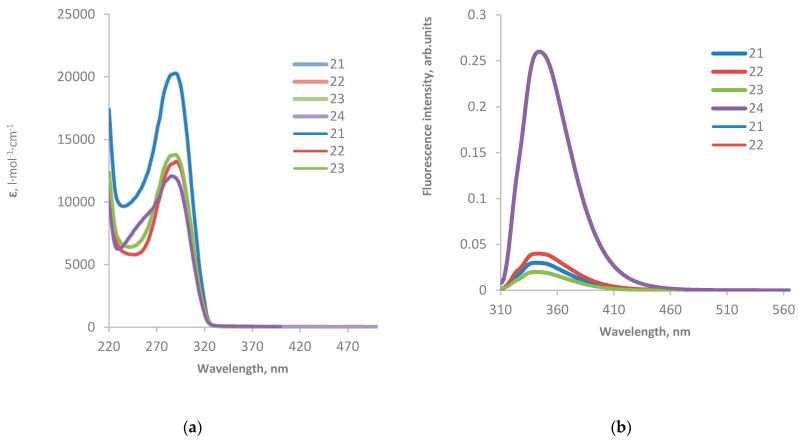
(**a**) Absorption and (**b**) emission spectra of solutions of compounds **21–24** in methanol.

The spectral characteristics of amides **21–24** are presented in Table 3. The maximum of the absorption band is in the range of 286–290 nm. These types of triazoles demonstrate emission in the region of 340–345 nm and quantum yields up to 26%. In spite of structural similarity, the influence of amine is very pronounced. For example, compound **21** has a rather high extinction coefficient of 29,740 L·mol^−1^·cm^−1^, and compound **24** has the highest quantum yield of 26% among the amides obtained (Figure 5).

Thus, the absorption and emission range of all studied compounds is in the ultraviolet region, and **12–19** compounds have sufficiently high quantum yields.

## 3. Materials and Methods

In general, ^1^H, ^13^C and ^19^F NMR spectra were recorded on Bruker AVANCE 400 MHz spectrometer in CD_3_CN and CDCl_3_ at 400.1, 100.6 and 376.5 MHz, respectively. Chemical shifts (*δ*) in ppm are reported with the use of the residual CHD_2_CN and chloroform signals (1.94, 7.25 for ^1^H and 1.30, 77.0 for ^13^C) as internal reference. The ^19^F chemical shifts were referenced to C_6_F_6_ (−162.9 ppm). The coupling constants (*J*) are given in Hertz (Hz). HRMS spectra were measured on the MicroTof Bruker Daltonics instrument. TLC analysis was performed on “Macherey-Nagel ALUGRAM Xtra SIL G/UV_254_” plates. Column chromatography was performed on silica gel “Macherey-Nagel 0.063–0.2 nm (Silica 60)”. All reagents were of reagent grade and were used as such or were distilled prior to use. Triazoles **1** were prepared as reported previously [43]. Melting points were determined on the Electrothermal 9100 apparatus (Electrothermal, Stone, Staffordshire, UK). Electronic absorption spectra were recorded on Genesys 50 (Thermo Scientific) in cuvettes with an optical path length of 1 cm at room temperature using methanol as a solvent. Emission spectra were recorded with a Hitachi F2700 spectrofluorometer (Hitachi, Tokyo, Japan) in 1 cm quartz cuvettes. The relative fluorescence quantum yields (φ) were measured using 2-aminopyridine 0.1 M H_2_SO_4_ (φ = 0.60) as a standard. [51]

**Screening of the optimal conditions for modification of triazoles 1 by alkylating reagents**. A 4 mL vial with a screw cap was charged with triazole **1a** (0.060 g, 0.25 mmol), solvent (0.5 mL), base (0.38 mmol, 1.5 equiv.) and corresponding alkylating reagent (0.275 mmol, 1.1 equiv., 0.035 g (BnCl) or 0.047 g(BnBr)). The reaction mixture was stirred at room temperature overnight. The yields and ratio of **3a** and **4a** were determined by ^19^F NMR using PhCF_3_ as an internal standard.

**Reaction of triazoles (1) with alkylating reagents (general procedure)**. A 4 mL vial with a screw cap was charged with corresponding triazole **1** (0.5 mmol), DMF (1 mL), Na_2_CO_3_ (80 mg, 0.38 mmol, 1.5 equiv.) and corresponding alkylating reagent (0.55 mmol, 1.1 equiv.) The reaction mixture was stirred at room temperature overnight or heated (8 h at 80 °C for **3l**, 14 h at 100 °C for **3k,** 16h at 100 °C for **3m**) and then was broken by 0.1 M HCl (20 mL). The product was extracted by CH_2_Cl_2_ (3 × 10 mL); the organic phase was washed with water (2 × 10 mL), brine (10 mL) and dried over Na_2_SO_4_. Volatiles were evaporated in vacuo; the residue formed was purified by column chromatography on silica gel using gradient eluation by hexane-CH_2_Cl_2_ mixture (3:1) followed by hexane-CH_2_Cl_2_ mixture (1:1) and CH_2_Cl_2_. Evaporation of the solvents afforded corresponding pure triazole **3**. Due to low amounts of minor triazoles **4,** these compounds were not separated completely from major triazoles **3** in some cases. Mostly ^1^H NMR and ^19^NMR were measured for **3**. Only the most characteristic signals of **3** are given in ^13^C NMR.

**1-(2-Benzyl-5-phenyl-2*H*-1,2,3-triazol-4-yl)-2,2,2-trifluoroethanone (3a)**. This was obtained from **1a** (120 mg, 0.498 mmol) and benzyl bromide (94 mg, 0.550 mmol) and purified using gradient eluating by hexane-CH_2_Cl_2_ (3:1) followed by hexane-CH_2_Cl_2_ (1:1) and CH_2_Cl_2_. Total yield (**3a** + **4a**) was 139 mg (84%), ratio **3a:4a** = 83:17. For pure **3a**, it was beige oil, yield 115 mg (70%). ^1^H NMR (CDCl_3_, 400.1 MHz) was δ 7.96–7.86 (m, 2H), 7.53–7.44 (m, 5H), 7.44–7.36(m, 3H), 5.71 (s, 2H). ^13^C{^1^H} NMR (CDCl_3_, 100.6 MHz) was δ 174.2 (q, ^2^*J*_CF_ = 37.0 Hz), 152.7, 136.4, 133.5, 130.1, 129.1, 129.0, 128.9, 128.40, 128.35, 128.2, 116.3 (q, ^1^*J*_CF_ = 290.7 Hz), 59.9. ^19^F NMR (CDCl_3_, 376.5 MHz): δ -74.9 (s, 3F). HRMS (ESI-TOF) was *m*/*z* [M + H]^+^. Calcd for C_17_H_13_F_3_N_3_O^+^ was 332.1005 and found: 332.1005. IR (ν, cm^−1^) was 1723 (C=O).

**1-(1-Benzyl-5-phenyl-1*H*-1,2,3-triazol-4-yl)-2,2,2-trifluoroethanone (4a)**. This was obtained from **1a** as an admixture (83:17) in the synthesis of **3a**. For pure **4a**, see the following: Beige thick oil, yield 24 mg (14%). ^1^H NMR (CDCl_3_, 400.1 MHz): δ 7.58–7.52 (m, 1H), 7.51–7.46 (m, 2H), 7.31–7.25 (m, 3H), 7.24–7.20 (m, 2H), 7.08–7.00 (m, 2H), 5.46 (s, 2H). ^13^C{^1^H} NMR (CDCl_3_, 100.6 MHz): δ 174.2 (q, ^2^*J*_CF_ = 37.3 Hz), 143.9, 138.1, 133.9, 130.9, 129.4, 129.0, 128.9, 128.7, 127.7, 124.4, 116.1 (q, ^1^*J*_CF_ = 290.6 Hz), 52.2. ^19^F NMR (CDCl_3_, 376.5 MHz): δ -75.4 (s, 3F). NMR data are in agreement with those in the literature [38].

**1-(2-Benzyl-5-(4-methoxyphenyl)-2*H*-1,2,3-triazol-4-yl)-2,2,2-trifluoroethanone (3b)**. This was obtained from **1b** (62 mg, 0.229 mmol) and benzyl bromide (43 mg, 0.251 mmol) and purified using gradient eluating by hexane-CH_2_Cl_2_ (3:1) followed by hexane-CH_2_Cl_2_ (1:1) and CH_2_Cl_2_. Total yield (**3b** + **4b**) was 77 mg (93%), ratio **3b:4b** = 81:19. For pure **3b**, see the following: White solid, yield 0.0624 g (75.3%). ^1^H NMR (CDCl_3_, 400.1 MHz): δ 7.89 (d, 2H, ^3^*J* = 8.9 Hz), 7.48–7.33 (m, 5H), 6.97 (d, 2H, ^3^*J* = 8.9 Hz), 5.68 (s, 2H), 3.85 (s, 3H). ^13^C{^1^H} NMR (CDCl_3_, 100.6 MHz): δ 174.3 (q, ^2^*J*_CF_ = 37.0 Hz), 161.0, 152.7, 136.1, 133.6, 130.6, 128.94, 128.89, 128.4, 120.6, 116.3 (q, ^1^*J*_CF_ = 290.8 Hz), 113.8, 59.8, 55.3. ^19^F NMR (CDCl_3_, 376.5 MHz): δ −74.8 (s, 3F). HRMS (ESI-TOF): *m*/*z* [M + H]^+^ Calcd for C_18_H_15_F_3_N_3_O_2_^+^: 362.1111; found: 362.1089.

**1-(1-Benzyl-5-(4-methoxyphenyl)-1*H*-1,2,3-triazol-4-yl)-2,2,2-trifluoroethanone (4b)**. This was obtained from **1b** as an admixture (81:19) in the synthesis of **3b**: colorless oil, yield 14.6 mg (17.3%). ^1^H NMR (CDCl_3_, 400.1 MHz): δ 7.33–7.27 (m, 3H), 7.17 (d, 2H, ^3^*J* = 8.7 Hz), 7.13–7.04 (m, 2H), 6.98 (d, 2H, ^3^*J* = 8.7 Hz), 5.47 (s, 2H), 3.87 (s, 3H). ^13^C{^1^H} NMR (CDCl_3_, 100.6 MHz): δ 174.2 (q, ^2^*J*_CF_ = 37.1 Hz), 161.5, 144.0, 137.9, 134.1, 131.1, 129.0, 128.7, 127.6, 116.0, 116.2 (q, ^1^*J*_CF_ = 290.8 Hz), 114.4, 55.4, 52.0. ^19^F NMR (CDCl_3_, 376.5 MHz): δ −75.2 (s, 3F).

**2,2,2-Trifluoro-1-(2-(4-nitrobenzyl)-5-phenyl-2*H*-1,2,3-triazol-4-yl)ethanone (3c)**. This was obtained from **1a** (48 mg, 0.199 mmol) and 1-(bromomethyl)-4-nitrobenzene (47 mg, 0.218 mmol) and purified using gradient eluating by hexane-CH_2_Cl_2_ (3:1) followed by hexane-CH_2_Cl_2_ (1:1) and CH_2_Cl_2_. Total yield (**3c** + **4c**) was 0.0609 g (81%), ratio **3c:4c** = 83:17. For pure **3c**, see the following: beige solid, m.p. 97–101 °C, yield 0.0493 g (65.6%). ^1^H NMR (CDCl_3_, 400.1 MHz): δ 8.25 (d, 2H, ^3^*J* = 8.7 Hz), 7.91–7.82 (m, 2H), 7.60 (d, 2H, ^3^*J* = 8.7 Hz), 7.49–7.43 (m, 3H), 5.81 (s, 2H). ^13^C{^1^H} NMR (CDCl_3_, 100.6 MHz): δ 174.1 (q, ^2^*J*_CF_ = 37.7 Hz), 153.1, 148.3, 140.1, 136.9, 130.4, 129.3, 129.1, 128.5, 127.8,124.3, 116.1 (q, ^1^*J*_CF_ = 290.6 Hz), 58.8. ^19^F NMR (CDCl_3_, 376.5 MHz): δ −75.0 (s, 3F). HRMS (ESI-TOF): *m*/*z* [M + H]^+^ Calcd for C_17_H_12_F_3_N_4_O_3_^+^: 377.0856; found: 377.0854.

**2,2,2-Trifluoro-1-(1-(4-nitrobenzyl)-5-phenyl-1*H*-1,2,3-triazol-4-yl)ethanone (4c)**. This was obtained from **1a** as an admixture (81:19) in the synthesis of **3c**: pale brown viscous mass, yield 11.6 mg (15.4%). ^1^H NMR (CDCl_3_, 400.1 MHz): δ 5.58 (s, 2H). ^19^F NMR (CDCl_3_, 376.5 MHz): δ -75.4 (s, 3F).

**1-(2-Allyl-5-phenyl-2*H*-1,2,3-triazol-4-yl)-2,2,2-trifluoroethanone (3d)**. This was obtained from **1a** (50.2 mg, 0.208 mmol) and 3-chloroprop-1-ene (21 mg, 0.276 mmol) and purified using gradient eluating by hexane-CH_2_Cl_2_ (3:1) followed by hexane-CH_2_Cl_2_ (1:1) and CH_2_Cl_2_. Total yield (**3d** + **4d)** was 46 mg (79 %), ratio **3d:4d** = 86:14. For pure **3d**, see the following: colorless oil, yield 39.6 mg (68%). ^1^H NMR (CDCl_3_, 400.1 MHz): δ 7.93–7.84 (m, 2H), 7.50–7.43 (m, 3H), 6.21–6.09 (m, 1H), 5.45–5.37 (m, 2H), 5.16 (dt, 2H, ^3^*J* = 6.3 Hz, ^4^*J* = 1.4 Hz). ^13^C{^1^H} NMR (CDCl_3_, 100.6 MHz): δ 174.3 (q, ^2^*J*_CF_ = 37.0 Hz), 152.6, 136.4, 130.1, 130.0, 129.1, 128.4, 128.2, 120.9, 116.2 (q, ^1^*J*_CF_ = 290.6 Hz), 58.5. ^19^F NMR (CDCl_3_, 376.5 MHz): δ −75.0 (s, 3F). HRMS (ESI-TOF): *m*/*z* [M + H]^+^ Calcd for C_13_H_11_F_3_N_3_O^+^: 282.0849; found: 282.0853.

**1-(1-Allyl-5-phenyl-1*H*-1,2,3-triazol-4-yl)2,2,2-trifluoroethanone (4d)**. This was obtained from **1a** as an admixture (86:14) in the synthesis of **3d**: colorless oil, yield 6.4 mg (11%). ^1^H NMR (CDCl_3_, 400.1 MHz): δ 7.58–7.50 (m, 3H), 7.40–7.36 (m, 2H), 5.95 (ddt, 1H, ^2^*J* = 16.9 Hz, ^3^*J* = 10.4 Hz, ^3^*J* = 5.7 Hz), 5.29 (d, 1H, ^3^*J* = 10.3 Hz), 5.06 (*pseudo*-dt, 1H, ^2^*J* = 17.1 Hz, ^4^*J* = 1.3 Hz), 4.89 (dt, ^3^*J* = 5.7 Hz, ^4^*J* = 1.5 Hz). ^19^F NMR (CDCl_3_, 376.5 MHz): δ −75.3 (s, 3F).

**2,2,2-Trifluoro-1-(2-methyl-5-phenyl-2*H*-1,2,3-triazol-4-yl)ethanone (3e)**. This was obtained from **1a** (51.9 mg, 0.215 mmol) and iodomethane (34 mg, 0.239 mmol) and purified using gradient eluating by hexane-CH_2_Cl_2_ (3:1) followed by hexane-CH_2_Cl_2_ (1:1) and CH_2_Cl_2_. Total yield **(3e** + **4e)** was 47.2 mg (86%), ratio **3e:4e** = 83:17. For pure **3e**, see the following: beige oil, yield 39.2 mg (71.4%). ^1^H NMR (CDCl_3_, 400.1 MHz): δ 7.92–7.84 (m, 2H), 7.50–7.44 (m, 3H), 4.35 (s, 3H). ^13^C{^1^H} NMR (CDCl_3_, 100.6 MHz): δ 174.1 (q, ^2^*J*_CF_ = 37.0 Hz), 152.6, 136.3, 130.1, 129.0, 128.4, 128.2, 116.2 (q, ^1^*J*_CF_ = 290.5 Hz), 42.8. ^19^F NMR (CDCl_3_, 376.5 MHz): δ −75.0 (s, 3F). HRMS (ESI-TOF): *m*/*z* [M + H]^+^ Calcd for C_11_H_9_F_3_N_3_O^+^: 256.0692; found: 256.0692.

**2,2,2-Trifluoro-1-(1-methyl-5-phenyl-1*H*-1,2,3-triazol-4-yl)ethanone (4e)**. This was obtained from **1a** as an admixture (83:17) in the synthesis of **3e**: beige oil, yield 8 mg (14.6%). ^1^H NMR (CDCl_3_, 400.1 MHz): δ 7.63–7.51 (m, 3H), 7.44–7.34 (m, 2H), 4.01 (s, 3H). ^13^C{^1^H} NMR (CDCl_3_, 100.6 MHz): δ 131.0, 129.4, 129.1, 35.5. ^19^F NMR (CDCl_3_, 376.5 MHz): δ −75.3 (s, 3F).

**1-(2-Ethyl-5-phenyl-2*H*-1,2,3-triazol-4-yl)-2,2,2-trifluoroethanone (3f)**. This was obtained from **1a** (50.7 mg, 0.210 mmol) and bromoethane (25.5 mg, 0.234 mmol) and purified using gradient eluating by hexane-CH_2_Cl_2_ (3:1) followed by hexane-CH_2_Cl_2_ (1:1) and CH_2_Cl_2_. Total yield (**3f** + **4f)** was 0.052 g (92%), ratio **3f:4f** = 91:9. For pure **3f**, see the following: colorless oil, yield 47.2 mg (83.7%). ^1^H NMR (CDCl_3_, 400.1 MHz): δ 8.00–7.80 (m, 2H), 7.54–7.41 (m, 3H), 4.61 (q, 2H, ^3^*J* = 7.4 Hz), 1.68 (t, 3H, ^3^*J* = 7.4 Hz). ^13^C{^1^H} NMR (CDCl_3_, 100.6 MHz): δ 174.2 (q, ^2^*J*_CF_ = 36.7 Hz), 152.4, 136.1, 130.0, 129.0, 128.4, 116.3 (q, ^1^*J*_CF_ = 290.7 Hz), 51.4, 14.5. ^19^F NMR (CDCl_3_, 376.5 MHz): δ −75.0 (s, 3F). HRMS (ESI-TOF): *m*/*z* [M + H]^+^ Calcd for C_12_H_11_F_3_N_3_O^+^: 270.0851; found: 270.0851.

**1-(1-Ethyl-5-phenyl-1*H*-1,2,3-triazol-4-yl)-2,2,2-trifluoroethanone (4f)**. This was obtained from **1a** as an admixture (91:9) in the synthesis of **3f:** colorless oil, yield 4.7 mg (8.3%). ^1^H NMR (CDCl_3_, 400.1 MHz): δ 7.61–7.52 (m, 3H), 7.39–7.36 (m, 2H), 4.33 (q, 2H, ^3^*J* = 7.3 Hz), 1.48 (t, 3H, ^3^*J* = 7.3 Hz). ^19^F NMR (CDCl_3_, 376.5 MHz): δ −75.3 (s, 3F).

**2,2,2-Trifluoro-1-(5-phenyl-2-propyl-2*H*-1,2,3-triazol-4-yl)ethanone (3g)**. This was obtained from **1a** (53.8 mg, 0.223 mmol) and bromopropane (30.3 mg, 0.246 mmol) and purified using gradient eluating by hexane-CH_2_Cl_2_ (3:1) followed by hexane-CH_2_Cl_2_ (1:1) and CH_2_Cl_2_. Total yield (**3g** + **4g**) was 58.7 mg (93%), ratio **3g:4g** = 92:8. For pure **3g**, see the following: beige oil, yield 54 mg (85.6%). ^1^H NMR (CDCl_3_, 400.1 MHz): δ 7.95–7.83 (m, 2H), 7.53–7.43 (m, 3H), 4.52 (t, 2H, ^3^*J* = 7.1 Hz), 2.10 (h (sextet), 2H, ^3^*J* = 7.3 Hz), 1.02 (t, 3H, ^3^*J* = 7.4 Hz). ^13^C{^1^H} NMR (CDCl_3_, 100.6 MHz): δ 174.3 (q, ^2^*J*_CF_ = 37.0 Hz), 152.3, 136.1, 130.0, 129.0, 128.42, 128.41, 116.3 (q, ^1^*J*_CF_ = 290.8 Hz), 57.8, 22.9, 10.9. ^19^F NMR (CDCl_3_, 376.5 MHz): δ −75.0 (s, 3F). HRMS (ESI-TOF): *m*/*z* [M + H]^+^ Calcd for C_13_H_13_F_3_N_3_O^+^: 284.1005; found: 284.1007.

**2,2,2-Trifluoro-1-(5-phenyl-1-propyl-1*H*-1,2,3-triazol-4-yl)ethanone (4g)**. this was obtained from **1a** as an admixture (92:8) in the synthesis of **3g**: yield 4.7 mg (7.4%). ^1^H NMR (CDCl_3_, 400.1 MHz): δ 7.60–7.52 (m, 3H), 7.36 (dd, 2H, ^3^*J* = 7.5 Hz, ^4^*J* = 1.6 Hz), 4.24 (t, 2H, ^3^*J* = 7.3 Hz), 1.92–1.82 (m, 2H), 0.87 (t, 3H, ^3^*J* = 7.4 Hz). ^13^C{^1^H} NMR (CDCl_3_, 100.6 MHz): δ 174.2 (q, ^2^*J*_CF_ = 37.1 Hz), 143.7, 137.9, 130.8, 129.3, 129.1, 128.4, 116.2 (q, ^1^*J*_CF_ = 289.3 Hz), 50.0, 29.7, 23.3. ^19^F NMR (CDCl_3_, 376.5 MHz): δ −75.3 (s, 3F).

**2,2,2-Trifluoro-1-(2-pentyl-5-phenyl-2*H*-1,2,3-triazol-4-yl)ethanone (3h)**. This was obtained from **1a** (51.2 mg, 0.212 mmol) and bromopentane (35.6 mg, 0.236 mmol) and purified using gradient eluating by hexane-CH_2_Cl_2_ (3:1) followed by hexane-CH_2_Cl_2_ (1:1) and CH_2_Cl_2_. Total yield **(3h** + **4h)** was 58 mg (88 %), ratio **3h:4h** = 92:8. For pure **3h**, see the following: colorless oil, yield 53.4 mg (81%). ^1^H NMR (CDCl_3_, 400.1 MHz): δ 7.96–7.85 (m, 2H), 7.54–7.42 (m, 3H), 4.55 (t, 2H, ^3^*J* = 7.2 Hz), 2.13–2.01 (m, 2H), 1.46–1.30 (m, 4H), 0.96–0.88 (m, 3H). ^13^C{^1^H} NMR (CDCl_3_, 100.6 MHz): δ 174.3 (q, ^2^*J*_CF_ = 37.0 Hz), 152.3, 136.0, 130.0, 129.0, 128.4, 116.3 (q, ^1^*J*_CF_ = 290.7 Hz), 56.2, 29.1, 28.4, 22.0, 13.8. ^19^F NMR (CDCl_3_, 376.5 MHz): δ −75.0 (s, 3F). HRMS (ESI-TOF): *m*/*z* [M + H]^+^ Calcd for C_15_H_17_F_3_N_3_O ^+^: 312.1318; found: 312.1322.

**2,2,2-Trifluoro-1-(1-pentyl-5-phenyl-1*H*-1,2,3-triazol-4-yl)ethanone (4h)**. This was obtained from **1a** as an admixture (92:8) in the synthesis of **3h**: colorless oil, yield 4.6 mg (7%). ^1^H NMR (CDCl_3_, 400.1 MHz): δ 7.61–7.51 (m, 3H), 7.38–7.33 (m, 2H), 4.30–4.20 (m, 2H), 1.87–1.77 (m, 2H), 1.26–1.17 (m, 4H), 0.82 (t, 3H, ^3^*J* = 6.9 Hz). ^13^C{^1^H} NMR (CDCl_3_, 100.6 MHz): δ 130.8, 129.3, 129.1, 124.8, 48.5, 29.6, 28.4, 21.9, 13.7. ^19^F NMR (CDCl_3_, 376.5 MHz): δ −75.3 (s, 3F). NMR data are in agreement with those in the literature [38].

**2,2,2-Trifluoro-1-(2-nonyl-5-phenyl-2*H*-1,2,3-triazol-4-yl)ethanone (3i)**. This was obtained from **1a** (53.9 mg, 0.224 mmol) and bromononan (51.3 mg, 0.248 mmol) and purified using gradient eluating by hexane-CH_2_Cl_2_ (3:1) followed by hexane-CH_2_Cl_2_ (1:1) and CH_2_Cl_2_. Total yield **(3i** + **4i)** was 68.6 mg (83 %), ratio **3i:4i** = 93:7. For pure **3i**, see the following: colorless oil, yield 63.8 mg (77.2%). ^1^H NMR (CDCl_3_, 400.1 MHz): δ 7.94–7.85 (m, 2H), 7.50–7.43 (m, 3H), 4.55 (t, 2H, ^3^*J* = 7.2 Hz), 2.06 (p, 2H, ^3^*J* = 7.3 Hz), 1.44–1.21 (m, 12H), 0.91–0.83 (m, 3H). ^13^C{^1^H} NMR (CDCl_3_, 100.6 MHz): δ 174.3 (q, ^2^*J*_CF_ = 37.0 Hz), 152.3, 136.0, 130.0, 129.0, 128.42, 128.40, 116.3 (q, ^1^*J*_CF_ = 290.7 Hz), 56.3, 31.8, 29.4, 29.3, 29.12, 28.9, 26.32, 22.61, 14.05. ^19^F NMR (CDCl_3_, 376.5 MHz): δ −75.0 (s, 3F). HRMS (ESI-TOF): *m*/*z* [M + H]^+^ Calcd for C_19_H_25_F_3_N_3_O^+^: 368.1944; found: 368.1950.

**2,2,2-Trifluoro-1-(1-nonyl-5-phenyl-1*H*-1,2,3-triazol-4-yl)ethanone (4i)**. This was obtained from **1a** as an admixture (93:7) in the synthesis of **3i**: Colorless oil, yield 4.8 mg (5.8%). ^1^H NMR (CDCl_3_, 400.1 MHz): δ 7.62–7.50 (m, 3H), 7.39–7.30 (m, 2H), 4.32–4.20 (m, 2H), 1.81 (p, 2H, ^3^*J* = 7.3 Hz), 1.32–1.13 (m, 12H), 0.86 (t, 3H, ^3^*J* = 7.0 Hz). ^13^C{^1^H} NMR (CDCl_3_, 100.6 MHz): δ 130.8, 129.3, 129.1, 124.8, 48.5, 31.7, 29.9, 29.2, 29.08, 28.7, 26.27, 22.59, 14.06. ^19^F NMR (CDCl_3_, 376.5 MHz): δ −75.3 (s, 3F).

**2,2,2-Trifluoro-1-(2-phenethyl-5-phenyl-2*H*-1,2,3-triazol-4-yl)ethanone (3j)**. This was obtained from **1a** (52.9 mg, 0.220 mmol) and (2-bromoethyl)benzene (44.8 mg, 0.242 mmol) and purified using gradient eluating by hexane-CH_2_Cl_2_ (3:1) followed by hexane-CH_2_Cl_2_ (1:1) and CH_2_Cl_2_. Total yield **(3j** + **4j)** was 64.8 mg (85 %), ratio **3j:4j** = 93:7. For pure **3j**, see the following: colorless oil, yield 60.3 mg (79%). ^1^H NMR (CDCl_3_, 400.1 MHz): δ 7.92–7.84 (m, 2H), 7.51–7.45 (m, 3H), 7.34–7.19 (m, 5H), 4.84–4.76 (m, 2H), 3.43–3.36 (m, 2H). ^13^C{^1^H} NMR (CDCl_3_, 100.6 MHz): δ 174.2 (q, ^2^*J*_CF_ = 37.2 Hz), 152.3, 136.5, 136.1, 130.1, 129.0, 128.8, 128.7, 128.4, 128.3, 127.1, 116.2 (q, ^1^*J*_CF_ = 290.7 Hz), 57.2, 35.6. ^19^F NMR (CDCl_3_, 376.5 MHz): δ −75.0 (s, 3F). HRMS (ESI-TOF): *m*/*z* [M + H_3_O]^+^ Calcd for C_18_H_17_F_3_N_3_O_2_^+^: 346.1267; found: 346.1270.

**2,2,2-Trifluoro-1-(1-phenethyl-5-phenyl-1*H*-1,2,3-triazol-4-yl)ethanone (4j)**. This was obtained from **1a** as an admixture (93:7) in the synthesis of **3j**: colorless oil, yield 4.5 mg (6%). ^1^H NMR (CDCl_3_, 400.1 MHz): δ 7.53–7.48 (m, 1H), 7.44–7.39 (m, 2H), 7.23–7.17 (m, 3H), 6.96–6.90 (m, 2H), 6.90–6.83 (m, 2H), 4.46 (t, 2H, ^3^*J* = 7.1 Hz), 3.19 (t, 2H, ^3^*J* = 7.1 Hz). ^19^F NMR (CDCl_3_, 376.5 MHz): δ -75.4 (s, 3F). NMR data are in agreement with those in the literature [38].

**2,2,2-Trifluoro-1-(2-isobutyl-5-phenyl-2*H*-1,2,3-triazol-4-yl)ethanone (3k)**. This was obtained from **1a** (48.5 mg, 0.201 mmol) and 1-chloro-2-methylpropane (27.8 mg, 0.300 mmol) by heating for 14 h at 100 °C and purified using gradient eluating by hexane-CH_2_Cl_2_ (3:1) followed by hexane-CH_2_Cl_2_ (1:1) and CH_2_Cl_2_. Total yield **(3k** + **4k)** was 50 mg (84%), ratio **3k:4k** = 91:9. For pure **3k**, see the following: colorless oil, yield 45.5 mg (76.2%). ^1^H NMR (CDCl_3_, 400.1 MHz): δ 7.95–7.82 (m, 2H), 7.53–7.42 (m, 3H), 4.37 (q, 2H, ^3^*J* = 7.3 Hz), 2.45 (hept, 1H, ^3^*J* = 6.9 Hz), 1.01 (s, 3H), 1.00 (s, 3H). ^13^C{^1^H} NMR (CDCl_3_, 100.6 MHz): δ 174.3 (q, ^2^*J*_CF_ = 36.7 Hz), 152.2, 136.0, 130.0, 129.0, 128.41, 128.39, 116.3 (q, ^1^*J*_CF_ = 290.7 Hz), 63.2, 29.4, 19.7. ^19^F NMR (CDCl_3_, 376.5 MHz): δ −75.0 (s, 3F). HRMS (ESI-TOF): *m*/*z* [M + H]^+^ Calcd for C_14_H_15_F_3_N_3_O^+^: 298.1163; found: 298.1163.

**2,2,2-Trifluoro-1-(1-isobutyl-5-phenyl-1*H*-1,2,3-triazol-4-yl)ethanone (4k)**. This was obtained from **1a** as an admixture (91:9) in the synthesis of **3k**: colorless oil, yield 4.5 mg (7.5%). ^1^H NMR (CDCl_3_, 400.1 MHz): δ 7.61–7.52 (m, 3H), 7.39–7.36 (m, 2H), 4.33 (q, 2H, ^3^*J* = 7.3 Hz), 1.48 (t, 3H, ^3^*J* = 7.3 Hz). ^19^F NMR (CDCl_3_, 376.5 MHz): δ −75.3 (s, 3F).

**2,2,2-Trifluoro-1-(2-isopropyl-5-phenyl-2*H*-1,2,3-triazol-4-yl)ethanone (3l)**. This was obtained from **1a** (49.8 mg, 0.207 mmol) and 1-bromo-2-methylpropane (29 mg, 0.238 mmol) by heating for 8 h at 80 °C and purified using gradient eluating by hexane-CH_2_Cl_2_ (3:1) followed by hexane-CH_2_Cl_2_ (1:1) and CH_2_Cl_2_. Total yield (**3l** + **4l)** was 46 mg (79%), ratio **3l:4l** = 94:6. For pure **3l**, see the following: colorless oil, yield 43.2 mg (74.3%). ^1^H NMR (CDCl_3_, 400.1 MHz): δ 7.95–7.87 (m, 2H), 7.50–7.44 (m, 3H), 4.97 (hept, 1H, ^3^*J* = 6.7 Hz), 1.69 (s, 3H), 1.68 (s, 3H). ^13^C{^1^H} NMR (CDCl_3_, 100.6 MHz): δ 174.4 (q, ^2^*J*_CF_ = 36.9 Hz), 152.0, 135.7, 130.0, 129.0, 128.6, 128.4, 116.3 (q, ^1^*J*_CF_ = 290.9 Hz), 59.0, 22.1. ^19^F NMR (CDCl_3_, 376.5 MHz): δ −75.0 (s, 3F). HRMS (ESI-TOF): *m*/*z* [M + H]^+^ Calcd for C_13_H_13_F_3_N_3_O ^+^: 284.1005; found: 284.1007.

**2,2,2-Trifluoro-1-(1-isopropyl-5-phenyl-1*H*-1,2,3-triazol-4-yl)ethanone (4l)**. This was obtained from **1a** as an admixture (94:6) in the synthesis of **3l**: colorless oil, yield 2.8 mg (4.7%). ^1^H NMR (CDCl_3_, 400.1 MHz): δ 7.60–7.52 (m, 3H), 7.36–7.31 (m, 2H), 4.54 (hept, 1H, ^3^*J* = 6.7 Hz), 1.61 (s, 3H), 1.59 (s, 3H). ^19^F NMR (CDCl_3_, 376.5 MHz): δ −75.3 (s, 3F).

**1-(2-Cyclohexyl-5-phenyl-2*H*-1,2,3-triazol-4-yl)-2,2,2-trifluoroethanone (3m)**. This was obtained from **1a** (48 mg, 0.199 mmol) and bromocyclohexane (36 mg, 0.221 mmol) by heating for 14 h at 100 °C and purified using gradient eluating by hexane-CH_2_Cl_2_ (3:1) followed by hexane-CH_2_Cl_2_ (1:1) and CH_2_Cl_2_. Total yield **(3m** + **4m)** was 45.1 mg (70%), ratio **3m:4m** = 93:7. For pure **3m**, see the following: white solid, m.p. 53–54 °C, yield 41.9 mg (65.1%). ^1^H NMR (CDCl_3_, 400.1 MHz): δ 7.97–7.82 (m, 2H), 7.52–7.40 (m, 3H), 4.68–4.50 (m, 1H), 2.34–2.23 (m, 2H), 2.06–1.90 (m, 4H), 1.80–1.72 (m, 1H), 1.54–1.28 (m, 3H). ^13^C{^1^H} NMR (CDCl_3_, 100.6 MHz): δ 174.5 (q, ^2^*J*_CF_ = 36.8 Hz), 151.9, 135.7, 129.9, 129.0, 128.7, 128.4, 116.5 (q, ^1^*J*_CF_ = 290.9 Hz), 65.6, 32.4, 25.0, 24.8. ^19^F NMR (CDCl_3_, 376.5 MHz): δ −74.9 (s, 3F). HRMS (ESI-TOF): *m*/*z* [M + H]^+^ Calcd for C_16_H_17_F_3_N_3_O^+^: 324.1318; found: 324.1318.

**1-(1-Cyclohexyl-5-phenyl-1*H*-1,2,3-triazol-4-yl)2,2,2-trifluoroethanone (4m)**. This was obtained from **1a** as an admixture (93:7) in the synthesis of **3m**: yield 3.2 mg (4.9%). ^1^H NMR (CDCl_3_, 400.1 MHz): δ 7.82–7.68 (m, 2H), 7.48–7.32 (m, 3H), 5.06–4.96 (m, 1H), 2.34–2.17 (m, 2H), 2.06–1.90 (m, 4H), 1.80–1.72 (m, 1H), 1.54–1.28 (m, 3H). ^19^F NMR (CDCl_3_, 376.5 MHz): δ −75.3 (s, 3F).

**1,1′-(2,2′-(Butane-1,4-diyl)bis(5-phenyl-2*H*-1,2,3-triazole-4,2-diyl))bis(2,2,2-trifluoroethanone) (3n)**. This was obtained from **1a** (74.1 mg, 0.307 mmol) and 1,4-dibromobytane (32.4 mg, 0.150 mmol) and purified using gradient eluating by hexane-CH_2_Cl_2_ (3:1) followed by hexane-CH_2_Cl_2_ (1:1) and CH_2_Cl_2_. Total yield **(3n** + **4n)** was 55 mg (67%), ratio **3n:4n** = 83:17. For pure **3n**, see the following: colorless oil, yield 45.7 mg (55.6%). ^1^H NMR (CDCl_3_, 400.1 MHz): δ 7.95–7.76 (m, 4H), 7.53–7.40 (m, 6H), 4.72–4.60 (m, 4H), 2.27–2.13 (m, 4H). ^13^C{^1^H} NMR (CDCl_3_, 100.6 MHz): δ 174.2 (q, ^2^*J*_CF_ = 37.0 Hz), 152.5, 136.3, 130.2, 129.0, 128.5, 128.1, 116.2 (q, ^1^*J*_CF_ = 290.8 Hz), 55.0, 26.1. ^19^F NMR (CDCl_3_, 376.5 MHz): δ −75.0 (s, 6F). HRMS (ESI-TOF): *m*/*z* [M + H]^+^ Calcd for C_24_H_19_F_6_N_6_O_2_^+^: 537.1468; found: 537.1467.

**2,2,2-Trifluoro-1-(5-phenyl-1-(4-(4-phenyl-5-(2,2,2-trifluoroacetyl)-2*H*-1,2,3-triazol-2-yl)butyl)-1*H*-1,2,3-triazol-4-yl)ethanone (4n).** This was obtained from 1a as an admixture (83:17) in the synthesis of 3n: colorless oil, yield 9.4 mg (11.4%). ^1^H NMR (CDCl_3_, 400.1 MHz): δ 7.89–7.83 (m, 2H), 7.55–7.44 (m, 6H), 7.32 (dd, 2H, ^3^*J* = 7.9 Hz, ^4^*J* = 1.4 Hz), 4.53 (t, 2H, ^3^*J* = 6.6 Hz), 4.36 (t, 2H, ^3^*J* = 6.9 Hz), 2.09–2.01 (m, 2H), 1.99–1.86 (m, 2H). ^13^C{^1^H} NMR (CDCl_3_, 100.6 MHz): δ 174.2 (q, ^2^*J*_CF_ = 37.5 Hz), 174.1 (q, ^2^*J*_CF_ = 37.7 Hz), 152.5, 143.7, 138.0, 136.3, 131.0, 130.3, 129.19, 129.16, 129.0, 128.5, 128.3, 128.0, 116.1 (q, ^1^*J*_CF_ = 289.8 Hz), 116.1 (q, ^1^*J*_CF_ = 291.0 Hz), 54.9, 47.5, 26.5, 26.0. ^19^F NMR (CDCl_3_, 376.5 MHz): δ −75.0 (s, 3F), −75.4 (s, 3F).

**Ethyl 2-(4-phenyl-5-(2,2,2-trifluoroacetyl)-2*H*-1,2,3-triazol-2-yl)acetate (3o)**. this was obtained from **1a** (52.9 mg, 0.219 mmol) and ethyl 2-bromoacetate (40.7 mg, 0.0244 mmol) and purified using gradient eluating by hexane-CH_2_Cl_2_ (3:1) followed by hexane-CH_2_Cl_2_ (1:1) and CH_2_Cl_2_. Total yield **(3o** + **4o)** was 57 mg (80%), ratio **3o:4o** = 90:10. For pure **3o**, see the following: Beige solid, m.p. 58–60 °C, yield 51.3 mg (72%). ^1^H NMR (CDCl_3_, 400.1 MHz): δ 7.93–7.84 (m, 2H), 7.50–7.44 (m, 3H), 5.34 (s, 2H), 4.29 (q, 2H, ^3^*J* = 7.1 Hz), 1.30 (t, 3H, ^3^*J* = 7.1 Hz). ^13^C{^1^H} NMR (CDCl_3_, 100.6 MHz): δ 174.2 (q, ^2^*J*_CF_ = 37.4 Hz), 165.3, 152.9, 137.1, 130,2, 129.1, 128.4, 127.9, 116.1 (q, ^1^*J*_CF_ = 290.8 Hz), 62.6, 56.5, 14.0. ^19^F NMR (CDCl_3_, 376.5 MHz): δ −75.1 (s, 3F). HRMS (ESI-TOF): *m*/*z* [M + H]^+^ Calcd for C_14_H_13_F_3_N_3_O_3_^+^: 328.0904; found: 328.0908.

**Ethyl 2-(5-phenyl-4-(2,2,2-trifluoroacetyl)-1*H*-1,2,3-triazol-1-yl)acetate (4o)**. This was obtained from **1a** as an admixture (90:10) in the synthesis of **3o**: colorless oil, yield 5.7 mg (8%). ^1^H NMR (CDCl_3_, 400.1 MHz): δ 7.56–7.52 (m, 2H), 7.38–7.36 (m, 2H), 5.04 (s, 2H), 4.21 (q, 2H, ^3^*J* = 7.1 Hz), 1.23 (t, 3H, ^3^*J* = 7.1 Hz). ^13^C{^1^H} NMR (CDCl_3_, 100.6 MHz): δ 131.1, 129.2, 62.7, 49.1. ^19^F NMR (CDCl_3_, 376.5 MHz): δ −75.3 (s, 3F). NMR data are in agreement with those in the literature [38].

***N,N*-Dimethyl-2-(4-phenyl-5-(2,2,2-trifluoroacetyl)-2*H*-1,2,3-triazol-2-yl)acetamide (3p)**. This was obtained from **1a** (47.6 mg, 0.198 mmol) and 2-chloro-*N,N*-dimethylacetamide (26.7 mg, 0.220 mmol) and purified using gradient eluating by hexane-CH_2_Cl_2_ (3:1) followed by hexane-CH_2_Cl_2_ (1:1) and CH_2_Cl_2_. Total yield **(3p** + **4p)** was 52 mg (81%), ratio **3p:4p** = 89:11. For the mixture of **3p** and **4p**, see the following: light yellow solid, m.p. 126–128 °C. For **3p**: ^1^H NMR (CDCl_3_, 400.1 MHz): δ 7.92–7.84 (m, 2H), 7.47–7.41 (m, 3H), 5.43 (s, 2H), 3.08 (s, 3H), 3.00 (s, 3H). ^13^C{^1^H} NMR (CDCl_3_, 100.6 MHz): δ 174.2 (q, ^2^*J*_CF_ = 37.2 Hz), 163.9, 152.8, 136.9, 130.1, 129.2, 128.3, 128.1, 116.2 (q, ^1^*J*_CF_ = 291.0 Hz), 56.9, 36.4, 35.9. ^19^F NMR (CDCl_3_, 376.5 MHz): δ −74.9 (s, 3F). ^1^H NMR (CD_3_CN, 400.1 MHz): δ 7.88–7.80 (m, 2H), 7.56–7.46 (m, 3H), 5.54 (s, 2H), 3.03 (s, 3H), 3.00 (s, 3H). ^13^C{^1^H} NMR (CD_3_CN, 100.6 MHz): δ 174.8 (q, ^2^*J*_CF_ = 36.5 Hz), 165.8, 153.2, 137.6, 131.0, 130.0, 129.4, 129.4, 117.2 (q, ^1^*J*_CF_ = 290.2 Hz), 58.0, 38.4, 35.9. ^19^F NMR (CD_3_CN, 376.5 MHz): δ −73.0 (s, 3F). HRMS (ESI-TOF): *m*/*z* [M + H]^+^ Calcd for C_14_H_14_F_3_N_4_O_2_^+^: 327.1063; found: 327.1070.

***N,N*-Dimethyl-2-(5-phenyl-4-(2,2,2-trifluoroacetyl)-1*H*-1,2,3-triazol-1-yl)acetamide (4p)**. This was obtained from **1a** as an admixture (89:11) in the synthesis of **3p**: ^1^H NMR (CDCl_3_, 400.1 MHz): δ 7.55–7.47 (m, 3H), 5.08 (s, 2H), 2.97 (s, 3H), 2.95 (s, 3H). ^13^C{^1^H} NMR (CDCl_3_, 100.6 MHz): δ 130.9, 129.4, 128.9, 55.3. ^19^F NMR (CDCl_3_, 376.5 MHz): δ −75.3 (s, 3F). ^1^H NMR (CD_3_CN, 400.1 MHz): δ 5.21 (s, 2H). ^13^C{^1^H} NMR (CD_3_CN, 100.6 MHz): δ 131.8, 130.4, 129.7, 129.4, 117.2 (q, ^1^*J*_CF_ = 290.2 Hz), 38.8, 36.9, 36.3. ^19^F NMR (CD_3_CN, 376.5 MHz): δ −73.2 (s, 3F).

**2,2,2-Trifluoro-1-(5-phenyl-2-tosyl-2*H*-1,2,3-triazol-4-yl)ethanone (3q)**. This was obtained from **1a** (60 mg, 0.249 mmol) and 4-toluenesulfonyl chloride (52 mg, 0.274 mmol) and purified using gradient eluating by hexane-CH_2_Cl_2_ (1:1) followed by CH_2_Cl_2_: white crystals, m.p. 156–160 °C, yield 73 mg (74%). ^1^H NMR (CDCl_3_, 400.1 MHz): δ 8.08 (d, 2H, ^3^*J* = 8.4 Hz), 7.83 (dd, 2H, ^3^*J* = 7.9 Hz, ^4^*J* = 1.4 Hz) 7.52–7.39 (m, 5H), 2.46 (s, 3H). ^13^C{^1^H} NMR (CDCl_3_, 100.6 MHz): δ 174.5 (q, ^2^*J*_CF_ = 37.9 Hz), 153.4, 148.1, 138.6, 131.6, 130.8, 130.6, 129.6, 129.3, 128.5, 126.9, 115.8 (q, ^1^*J*_CF_ = 290.5 Hz), 21.9. ^19^F NMR (CDCl_3_, 376.5 MHz): δ −75.2 (s, 3F). HRMS (ESI-TOF): *m*/*z* [M + H]^+^ Calcd for C_17_H_13_F_3_N_3_O_3_S^+^: 396.0324; found: 396.0626. IR (ν, cm^−1^): 1731 (C=O).

**2,2,2-Trifluoro-1-(2-(methylsulfonyl)-5-phenyl-2*H*-1,2,3-triazol-4-yl)ethanone (3r)**. This was obtained from **1a** (58.5 mg, 0.243 mmol) and methanesulfonyl chloride (30.5 mg, 0.267 mmol) and purified using gradient eluating by hexane-CH_2_Cl_2_ (3:1) followed by hexane-CH_2_Cl_2_ (1:1) and CH_2_Cl_2:_ pale yellow solid, m.p. 99–100 °C, yield 62 mg (80%). ^1^H NMR (CDCl_3_, 400.1 MHz): δ 7.93–7.82 (m, 2H), 7.56–7.46 (m, 3H), 3.62 (s, 3H). ^13^C{^1^H} NMR (CDCl_3_, 100.6 MHz): δ 174.4 (q, ^2^*J*_CF_ = 38.6 Hz), 153.6, 138.6, 131.1, 129.3, 128.7, 126.6, 115.8 (q, ^1^*J*_CF_ = 290.4 Hz), 41.7. ^19^F NMR (CDCl_3_, 376.5 MHz): δ -75.2 (s, 3F). HRMS (ESI-TOF): *m*/*z* [M + H]^+^ Calcd for C_11_H_9_F_3_N_3_O_3_S ^+^: 320.0313; found: 320.0315.

**Synthesis of 2-aryltriazoles (5–11) by the reaction of triazoles (1) with aryl halogenides (general procedure)**. A 4 mL vial with a screw cap was charged with corresponding triazole **1** (0.5 mmol), DMF (1 mL), Na_2_CO_3_ (80 mg, 0.38 mmol, 1.5 equiv.) and corresponding aryl halogenide (0.55 mmol, 1.1 equiv.). The reaction mixture was stirred at room temperature for 1 day (for **5**) or heated at 90–100 °C for 6–8 h until full consumption of the starting material (^19^F NMR control) occurred. The reaction mixture was broken by 0.1 M HCl (20 mL). The product was extracted by CH_2_Cl_2_ (3 × 10 mL); the organic phase was washed with water (2 × 10 mL), brine (10 mL) and dried over Na_2_SO_4_. Volatiles were evaporated in vacuo; the residue formed was purified by column chromatography on silica gel using gradient eluation by hexane-CH_2_Cl_2_ mixture (3:1) followed by hexane-CH_2_Cl_2_ mixture (1:1). Evaporation of the solvents afforded corresponding pure triazole **5–11**.

**1-(2-(2,4-Dinitrophenyl)-5-phenyl-2*H*-1,2,3-triazol-4-yl)-2,2,2-trifluoroethanone (5)**. This was obtained from **1a** (57 mg, 0.237 mmol) and 1-fluoro-2,4-dinitrobenzene (49 mg, 0.263 mmol) and purified using gradient eluating by hexane-CH_2_Cl_2_ (3:1) followed by hexane-CH_2_Cl_2_ (1:1): pale yellow powder, m.p. 132–134 °C, yield 79 mg (82%). ^1^H NMR (CDCl_3_, 400.1 MHz): δ 8.67 (d, 1H, ^4^*J* = 2.4 Hz), 8.56 (dd, 1H, ^3^*J* = 8.9 Hz, ^4^*J* = 2.4 Hz), 8.31 (d, 1H, ^3^*J* = 8.9 Hz), 7.83 (dd, 2H, ^3^*J* = 7.7 Hz, ^4^*J* = 1.7 Hz), 7.49–7.40 (m, 3H). ^13^C{^1^H} NMR (CDCl_3_, 100.6 MHz): δ 173.7 (q, ^2^*J*_CF_ = 38.2 Hz), 153.8, 147.1, 142.8, 138.7, 134.1, 130.8, 129.0, 128.4, 127.4, 126.4, 120.7, 115.6 (q, ^1^*J*_CF_ = 290.5 Hz). ^19^F NMR (CDCl_3_, 376.5 MHz): δ −75.0 (s, 3F). HRMS (ESI-TOF): *m*/*z* [M + H]^+^ Calcd for C_16_H_9_F_3_N_5_O_5_^+^: 408.0550; found: 408.0549. IR (ν, cm^−1^): 1738 (C=O); 1545, 1540, 1349, 1335 (NO_2_).

**2,2,2-Trifluoro-1-(2-(4-nitrophenyl)-5-phenyl-2*H*-1,2,3-triazol-4-yl)ethanone (6)**. This was obtained from **1a** (60 mg, 0.249 mmol) and 1-fluoro-4-nitrobenzene (44 mg, 0.312 mmol) and purified using gradient eluating by hexane-CH_2_Cl_2_ (3:1) followed by hexane-CH_2_Cl_2_ (1:1): yellow solid, m.p. 166–168 °C, yield 59.1 mg (66%). ^1^H NMR (CDCl_3_, 400.1 MHz): δ 8.47–8.37 (m, 4H), 8.04–7.91 (m, 2H), 7.58–7.49 (m, 3H). ^13^C{^1^H} NMR (CDCl_3_, 100.6 MHz): δ 174.3 (q, ^2^*J*_CF_ = 37.8 Hz), 153.7, 147.7, 142.5, 138.3, 130.8, 129.2, 128.6, 127.3, 125.3, 120.2, 116.1 (q, ^1^*J*_CF_ = 290.4 Hz). ^19^F NMR (CDCl_3_, 376.5 MHz): δ −60.5 (s, 3F), −75.0 (s, 3F). HRMS (ESI-TOF): *m*/*z* [M + H]^+^ Calcd for C_16_H_10_F_3_N_4_O_3_^+^: 363.0700; found: 363.0704.

**2,2,2-Trifluoro-1-(2-(4-nitro-2-(trifluoromethyl)phenyl)-5-phenyl-2*H*-1,2,3-triazol-4-yl)ethanone (7)**. This was obtained from **1a** (48.7 mg, 0.202 mmol) and 1-chloro-4-nitro-2-(trifluoromethyl)benzene (50.9 mg, 0.226 mmol) and purified using gradient eluating by hexane-CH_2_Cl_2_ (3:1) followed by hexane-CH_2_Cl_2_ (1:1): light yellow solid, m.p. 109–111 °C, yield 46 mg (53%). ^1^H NMR (CDCl_3_, 400.1 MHz): δ 8.81 (d, 1H, ^4^*J* = 2.4 Hz), 8.62 (dd, 1H, ^3^*J* = 8.8 Hz, ^4^*J* = 2.5 Hz), 8.22 (d, 1H, ^3^*J* = 8.8 Hz), 8.02–7.94 (m, 2H), 7.56–7.49 (m, 3H). ^13^C{^1^H} NMR (CDCl_3_, 100.6 MHz): δ 174.3 (q, ^2^*J*_CF_ = 38.3 Hz), 153.7, 147.7, 140.8, 138.8, 130.9, 129.2, 128.7, 128.3, 127.9, 127.0, 126.2 (q, ^2^*J*_CF_ = 35.1 Hz), 124.2 (q, ^3^*J*_CF_ = 5.3 Hz), 121.5 (q, ^1^*J*_CF_ = 274.4 Hz), 116.0 (q, ^1^*J*_CF_ = 290.4 Hz). ^19^F NMR (CDCl_3_, 376.5 MHz): δ −60.5 (s, 3F), −75.3 (s, 3F). HRMS (ESI-TOF): *m*/*z* [M + H_3_O]^+^ Calcd for C_17_H_10_F_6_N_4_O_4_^+^: 449.0679; found: 449.0675.

**2,2,2-Trifluoro-1-(2-(2-nitro-4-(trifluoromethyl)phenyl)-5-phenyl-2*H*-1,2,3-triazol-4-yl)ethanone (8)**. This was obtained from **1a** (51 mg, 0.216 mmol) and 1-chloro-2-nitro-4-(trifluoromethyl)benzene (54 mg, 0.24 mmol) and purified using gradient eluating by hexane-CH_2_Cl_2_ (3:1) followed by hexane-CH_2_Cl_2_ (1:1): pale yellow oil, yield 57 mg (62%). ^1^H NMR (CDCl_3_, 400.1 MHz): δ 8.27 (d, 1H, ^3^*J* = 8.5 Hz), 8.19 (*pseudo*-d, 1H, ^4^*J* = 1.1 Hz), 8.05 (dd, 1H, ^3^*J* = 8.5 Hz, ^4^*J* = 4.1 Hz), 7.97–7.88 (m, 2H), 7.57–7.46 (m, 3H). ^13^C{^1^H} NMR (CDCl_3_, 100.6 MHz): δ 174.1 (q, ^2^*J*_CF_ = 38.0 Hz), 153.9, 143.4, 138.8, 133.3, 132.7 (q, ^2^*J*_CF_ = 35.0 Hz), 131.0, 129.9 (q, ^3^*J*_CF_ = 3.5 Hz), 129.3, 128.7, 126.9, 126.1, 122.8 (q, ^3^*J*_CF_ = 3.6 Hz), 122.2 (q, ^1^*J*_CF_ = 273.3 Hz), 116.0 (q, ^1^*J*_CF_ = 290.7 Hz). ^19^F NMR (CDCl_3_, 376.5 MHz): δ −64.1 (s, 3F), −75.2 (s, 3F). HRMS (ESI-TOF): *m*/*z* [M + H]^+^ Calcd for C_17_H_9_F_6_N_4_O_3_^+^: 431.0573; found: 431.0576.

**2,2,2-Trifluoro-1-(2-(8-nitroquinolin-5-yl)-5-phenyl-2*H*-1,2,3-triazol-4-yl)ethanone (9)**. This was obtained from **1a** (53.8 mg, 0.223 mmol) and 5-chloro-8-nitroquinoline (66 mg, 0.317 mmol) and purified using gradient eluating by hexane-CH_2_Cl_2_ (3:1) followed by hexane-CH_2_Cl_2_ (1:1): yellow powder, m.p. 114–116 °C, yield 36 mg (39%). ^1^H NMR (CDCl_3_, 400.1 MHz): δ 9.09 (dd, 1H, ^3^*J* = 4.1 Hz, ^4^*J* = 1.6 Hz), 9.04 (dd, 1H, ^3^*J* = 8.9 Hz, ^4^*J* = 1.6 Hz), 8.49 (d, 1H, ^3^*J* = 8.3 Hz), 8.20 (d, 1H, ^3^*J* = 8.3 Hz), 8.03–7.95 (m, 2H), 7.76 (dd, 1H, ^3^*J* = 8.9 Hz, ^4^*J* = 4.1 Hz), 7.53–7.47 (m, 3H). ^13^C{^1^H} NMR (CDCl_3_, 100.6 MHz): δ 174.5 (q, ^2^*J*_CF_ = 37.5 Hz), 153.3, 152.8, 146.5, 142.5, 140.9, 138.3, 132.0, 130.4, 129.3, 128.5, 127.7, 125.5, 125.0, 123.6, 122.3, 121.2, 116.2 (q, ^1^*J*_CF_ = 290.6 Hz). ^19^F NMR (CDCl_3_, 376.5 MHz): δ −75.0 (s, 3F). HRMS (ESI-TOF): *m*/*z* [M + H]^+^ Calcd for C_19_H_11_F_3_N_5_O_3_^+^: 414.0809; found: 414.0809.

**Ethyl 2,3,5,6-tetrafluoro-4-(4-phenyl-5-(2,2,2-trifluoroacetyl)-2*H*-1,2,3-triazol-2-yl)benzoate (10)**. This was obtained from **1a** (58.5 mg, 0.243 mmol) and ethyl 2,3,4,5,6-pentafluorobenzoate (64 mg, 0.267 mmol) and purified using gradient eluating by hexane-CH_2_Cl_2_ (3:1) followed by hexane-CH_2_Cl_2_ (1:1): pale yellow solid, m.p. 72–75 °C, yield 79.5 mg (71%). ^1^H NMR (CDCl_3_, 400.1 MHz): δ 7.98–7.85 (m, 2H), 7.57–7.47 (m, 3H), 4.51 (q, 2H, ^3^*J* = 7.2 Hz), 1.43 (t, 3H, ^3^*J* = 7.2 Hz). ^13^C{^1^H} NMR (CDCl_3_, 100.6 MHz): δ 174.2 (q, ^2^*J*_CF_ = 38.2 Hz), 158.4, 153.7, 145.1 (ddt, ^2^*J*_CF_ = 255.2 Hz, ^3^*J*_CF_ = 13.1 Hz, ^4^*J*_CF_ = 5.4 Hz), 142.2 (ddd, ^2^*J*_CF_ = 255.2 Hz, ^3^*J*_CF_ = 15.6 Hz, ^4^*J*_CF_ = 4.4 Hz), 138.9, 130.8, 129.3, 128.6, 126.9, 120.9 (tt, ^3^*J*_CF_ = 12.7 Hz, ^4^*J*_CF_ = 2.7 Hz), 116.0 (q, ^1^*J*_CF_ = 290.6 Hz), 115.2 (t, ^3^*J*_CF_ = 16.9 Hz), 63.4, 14.0. ^19^F NMR (CDCl_3_, 376.5 MHz): δ -75.2 (s, 3F), −138.13 – −138.23 (m, 2F), −145.58 – −145.74 (m, 2F). HRMS (ESI-TOF): *m*/*z* [M + H]^+^ Calcd for C_19_H_11_F_7_N_3_O_3_^+^: 462.0683; found: 462.0681.

**2,2,2-Trifluoro-1-(5-(4-methoxyphenyl)-2-(4-nitrophenyl)-2*H*-1,2,3-triazol-4-yl)ethanone (11)**. This was obtained from **1b** (52 mg, 0.192 mmol) and 1-fluoro-4-nitrobenzene (30 mg, 0.213 mmol) and purified using gradient eluating by hexane-CH_2_Cl_2_ (3:1) followed by hexane-CH_2_Cl_2_ (1:1): white solid, m.p. 55–57 °C, yield 47.7 mg (63%). ^1^H NMR (CDCl_3_, 400.1 MHz): δ 8.50–8.34 (m, 4H), 7.99 (d, 2H, ^3^*J* = 8.8 Hz), 7.02 (d, 2H, ^3^*J* = 8.8 Hz), 3.88 (s, 3H). ^13^C{^1^H} NMR (CDCl_3_, 100.6 MHz): δ 174.4 (q, ^2^*J*_CF_ = 37.4 Hz), 161.7, 153.6, 147.7, 142.6, 138.1, 130.8, 125.3, 120.2, 119.7, 116.2 (q, ^1^*J*_CF_ = 290.7 Hz), 114.1, 55.4. ^19^F NMR (CDCl_3_, 376.5 MHz): δ −74.8 (s, 3F). HRMS (ESI-TOF): *m*/*z* [M + H]^−^ Calcd for C_17_H_12_F_3_N_4_O_4_^−^: 393.0816; found: 393.0822.

**Synthesis of 2-aryltriazoles (12–20) by the reaction of triazoles (1) with boronic acids (general procedure).**

A 30 mL vial was charged with corresponding triazole (**1**) (0.25 mmol), DMSO (1.5 mL), corresponding boronic acid (0.385 mmol, 1.5 equiv.) and Cu(OAc)_2_·H_2_O (5.1 mg, 0.257 mmol, 0.1 equiv.). The reaction mixture was heated at 90–100 °C for 8–10 h at open air using a magnetic stirrer with heating. The reaction mixture was poured into 0.1 M HCl (20 mL) and extracted with CH_2_Cl_2_ (3 × 20 mL). The combined organic phase was washed with water (3 × 20 mL), dried over Na_2_SO_4_, and then volatiles were evaporated in vacuo. The residue was purified by column chromatography on silica gel using gradient eluating by hexane-CH_2_Cl_2_ (3:1) followed by hexane-CH_2_Cl_2_ (1:1) as eluents.

**1-(2,5-diPhenyl-2*H*-1,2,3-triazol-4-yl)-2,2,2-trifluoroethanone (12)**. This was obtained from triazole **1a** (400 mg, 1.660 mmol) and PhB(OH)_2_ (309 mg, 2.553 mmol): colorless crystals, m.p. 93–95 °C yield 367 mg (77%). ^1^H NMR (CDCl_3_, 400.1 MHz): δ 8.28–8.18 (m, 2H), 8.03–7.95 (m, 2H), 7.59–7.46 (m, 6H). ^13^C{^1^H} NMR (CDCl_3_, 100.6 MHz): δ 174.4 (q, ^2^*J*_CF_ = 37.5 Hz), 152.9, 138.8, 137.1, 130.4, 129.6, 129.4, 129.2, 128.5, 128.1, 119.7, 116.3 (q, ^1^*J*_CF_ = 290.9 Hz). ^19^F NMR (CDCl_3_, 376.5 MHz): δ −74.9 (s, 3F). HRMS (ESI-TOF): *m*/*z* [M + H]^+^ Calcd for C_16_H_11_F_3_N_3_O^+^: 318.0849; found: 318.0851. IR (ν, cm^−1^): 1716 (C=O).

**2,2,2-Trifluoro-1-(5-(4-methoxyphenyl)-2-phenyl-2*H*-1,2,3-triazol-4-yl)-ethanone (13)**. This was obtained from triazole **1b** (77 mg, 0.284 mmol) and PhB(OH)_2_ (57 mg, 0.471 mmol): beige solid, m.p. 124–126 °C, yield 63 mg (64%). ^1^H NMR (CDCl_3_, 400.1 MHz): δ 8.28–8.15 (m, 2H), 8.02 (d, 2H, ^3^*J* = 9.0 Hz), 7.58–7.51 (m, 2H), 7.49–7.43 (m, 1H), 7.02 (d, 2H, ^3^*J* = 8.9 Hz), 3.87 (s, 3H). ^13^C{^1^H} NMR (CDCl_3_, 100.6 MHz): δ 174.4 (q, ^2^*J*_CF_ = 37.1 Hz), 161.3, 152.8, 138.8, 136.8, 130.7, 129.5, 129.3, 120.4, 119.7, 116.4 (q, ^1^*J*_CF_ = 290.9 Hz), 55.3. ^19^F NMR (CDCl_3_, 376.5 MHz): δ −74.7 (s, 3F). HRMS (ESI-TOF): *m*/*z* [M + H]^+^ Calcd for C_17_H_13_F_3_N_3_O_2_^+^: 348.0954; found: 348.0956.

**2,2,2-Trifluoro-1-(2-phenyl-5-(*p*-tolyl)-2*H*-1,2,3-triazol-4-yl)-ethanone (14)**. This was obtained from triazole **1c** (95 mg, 0.373 mmol) and PhB(OH)_2_ (68 mg, 0.562 mmol): white solid, m.p. 124–126 °C, yield 109 mg (88%). ^1^H NMR (CDCl_3_, 400.1 MHz): δ 8.22 (d, 2H, ^3^*J* = 7.6 Hz), 7.93 (d, 2H, ^3^*J* = 8.2 Hz), 7.55 (t, 2H, ^3^*J* = 7.7 Hz), 7.47 (t, 1H, ^3^*J* = 7.3 Hz), 7.32 (d, 2H, ^3^*J* = 8.0 Hz), 2.44 (s, 3H). ^13^C{^1^H} NMR (CDCl_3_, 100.6 MHz): δ 174.4 (q, ^2^*J*_CF_ = 37.2 Hz), 152.9, 140.6, 138.8, 137.0, 129.5, 129.3, 129.2, 129.1, 125.2, 119.6, 116.4 (q, ^1^*J*_CF_ = 290.9 Hz), 21.4. ^19^F NMR (CDCl_3_, 376.5 MHz): δ -74.6 (s, 3F). HRMS (ESI-TOF): *m*/*z* [M + H]^+^ Calcd for C_17_H_13_F_3_N_3_O^+^: 332.1005; found: 332.1007.

**2,2,2-Trifluoro-1-(2-phenyl-5-(4-(trifluorometyl)phenyl)-2*H*-1,2,3-triazol-4-yl)-ethanone (15)**. This was obtained from triazole **1d** (54 mg, 0.175 mmol) and PhB(OH)_2_ (31 mg, 0.256 mmol): white solid, m.p. 78–80 °C, yield 43 mg (64%). ^1^H NMR (CDCl_3_, 400.1 MHz): δ 8.22 (d, 2H, ^3^*J* = 7.8 Hz), 8.14 (d, 2H, ^3^*J* = 8.1 Hz), 7.77 (t, 2H, ^3^*J* = 8.1 Hz), 7.61–7.46 (m, 3H). ^13^C{^1^H} NMR (CDCl_3_, 100.6 MHz): δ 174.5 (q, ^2^*J*_CF_ = 37.8 Hz), 151.4, 138.6, 137.3, 132.1 (q, ^2^*J*_CF_ = 32.9 Hz), 131.6, 129.72, 129.67, 129.6, 125.5 (q, ^4^*J*_CF_ = 3.5 Hz), 123.8 (q, ^1^*J*_CF_ = 272.4 Hz), 119.8, 116.2 (q, ^1^*J*_CF_ = 290.6 Hz). ^19^F NMR (CDCl_3_, 376.5 MHz): δ −64.1 (s, 3F), −74.6 (s, 3F). HRMS (ESI-TOF): *m*/*z* [M + H]^+^ Calcd for C_17_H_10_F_6_N_3_O^+^: 386.0723; found: 386.0737.

**2,2,2-Trifluoro-1-(2-(4-methoxyphenyl)-5-phenyl-2*H*-1,2,3-triazol-4-yl)-ethanone (16)**. This was obtained from triazole **1a** (45 mg, 0.187 mmol) and (4-methoxyphenyl)boronic acid (51 mg, 0.338 mmol): white solid, m.p. 79–81 °C, yield 48 mg (74%). ^1^H NMR (CDCl_3_, 400.1 MHz): δ 8.13 (d, 2H, ^3^*J* = 9.2 Hz), 8.03–7.96 (m, 2H), 7.53–7.47 (m, 3H), 7.03 (d, 2H, ^3^*J* = 9.2 Hz), 3.88 (s, 3H). ^13^C{^1^H} NMR (CDCl_3_, 100.6 MHz): δ 174.3 (q, ^2^*J*_CF_ = 37.0 Hz), 160.4, 152.8, 136.7, 132.4, 130.3, 129.2, 128.4, 128.2, 121.2, 116.4 (q, ^1^*J*_CF_ = 290.6 Hz), 114.6, 55.6. ^19^F NMR (CDCl_3_, 376.5 MHz): δ −74.8 (s, 3F). HRMS (ESI-TOF): *m*/*z* [M + H]^+^ Calcd for C_17_H_13_F_3_N_3_O_2_^+^: 348.0954; found: 348.0957.

**2,2,2-Trifluoro-1-(2-(4-(hexyloxy)phenyl)-5-phenyl-2*H*-1,2,3-triazol-4-yl)-ethanone (17)**. This was obtained from triazole **1a** (53 mg, 0.220 mmol) and (4-(hexyloxy)phenyl)boronic acid (73 mg, 0.329 mmol): white solid, m.p. 67–69 °C, yield 63 mg (69%). ^1^H NMR (CDCl_3_, 400.1 MHz): δ 8.15–8.06 (m, 2H), 8.05–7.93 (m, 2H), 7.54–7.45 (m, 3H), 7.06–6.96 (m, 2H), 4.02 (t, 2H, ^3^*J* = 6.6 Hz), 1.86–1.76 (m, 2H), 1.53–1.44 (m, 2H), 1.40–1.31 (m, 4H), 0.92 (t, 3H, ^3^*J* = 7.0 Hz). ^13^C{^1^H} NMR (CDCl_3_, 100.6 MHz): δ 174.3 (q, ^2^*J*_CF_ = 37.1 Hz), 160.0, 152.8, 136.7, 132.2, 130.2, 129.2, 128.5, 128.2, 121.2, 116.4 (q, ^1^*J*_CF_ = 291.0 Hz), 115.1, 68.5, 31.6, 29.1, 25.7, 22.6, 14.0. ^19^F NMR (CDCl_3_, 376.5 MHz): δ −74.9 (s, 3F). HRMS (ESI-TOF): *m*/*z* [M + H]^+^ Calcd for C_22_H_23_F_3_N_3_O_2_^+^: 418.1737; found: 418.1733.

**1-(2-(4-Chloro-3-fluorophenyl)-5-phenyl-2*H*-1,2,3-triazol-4-yl)-2,2,2-trifluoroethanone (18)**. This was obtained from triazole **1a** (38 mg, 0.158 mmol) and (4-chloro-3-fluorophenyl)boronic acid (41 mg, 0.235 mmol): pale yellow solid, m.p. 110–113 °C, yield 44.3 mg (76%). ^1^H NMR (CDCl_3_, 400.1 MHz): δ 8.05 (dd, 1H, ^3^*J* = 9.3 Hz, ^4^*J* = 2.4 Hz), 8.02–7.94 (m, 3H), 7.58 (dd, 1H, ^3^*J* = 8.7 Hz, ^3^*J* = 7.6 Hz), 7.54–7.48 (m, 3H). ^13^C{^1^H} NMR (CDCl_3_, 100.6 MHz): δ 174.2 (q, ^2^*J*_CF_ = 37.5 Hz), 158.3 (d, ^1^*J*_CF_ = 250.9 Hz), 153.3, 138.0 (d, ^3^*J*_CF_ = 9.1 Hz), 137.6, 131.1 (d, ^2^*J*_CF_ = 92.7 Hz), 129.2, 128.6, 127.6, 122.3 (d, ^3^*J*_CF_ = 17.9 Hz), 116.2 (q, ^1^*J*_CF_ = 291.2 Hz), 115.8 (d, ^4^*J*_CF_ = 3.9 Hz), 108.6 (d, ^3^*J*_CF_ = 26.9 Hz). ^19^F NMR (CDCl_3_, 376.5 MHz): δ −75.0 (s, 3F), −112.2 (t, 1F, *J* = 8.4 Hz). HRMS (ESI-TOF): *m*/*z* [M + H]^+^ Calcd for C_16_H_9_ClF_4_N_3_O^+^: 370.0365; found: 370.0366.

**2,2,2-Trifluoro-1-(5-phenyl-2-(*p*-tolyl)-2*H*-1,2,3-triazol-4-yl)-ethanone (19)**. This was obtained from triazole **1a** (36 mg, 0.149 mmol) and *p*-tolylboronic acid (30 mg, 0.221 mmol): white solid, m.p. 112–115 °C, yield 44 mg (89%). ^1^H NMR (CDCl_3_, 400.1 MHz): δ 8.13–8.06 (m, 2H), 8.05–7.96 (m, 2H), 7.55–7.47 (m, 3H), 7.34 (d, 2H, ^3^*J* = 8.2 Hz), 2.44 (s, 3H). ^13^C{^1^H} NMR (CDCl_3_, 100.6 MHz): δ 174.4 (q, ^2^*J*_CF_ = 37.4 Hz), 152.8, 139.7, 136.9, 136.6, 130.3, 130.1, 129.2, 128.5, 128.2, 119.6, 116.3 (q, ^1^*J*_CF_ = 290.5 Hz), 21.2. ^19^F NMR (CDCl_3_, 376.5 MHz): δ −74.9 (s, 3F). HRMS (ESI-TOF): *m*/*z* [M + H]^+^ Calcd for C_17_H_13_F_3_N_3_O^+^: 332.1005; found: 332.1007.

**2,2,2-Trifluoro-1-(5-phenyl-2-(thiophen-3-yl)-2H-1,2,3-triazol-4-yl)-ethanone (20).** This was obtained from **1a** (43 mg, 0.178 mmol) and thiophen-3-ylboronic acid (34 mg, 0.266 mmol): colorless solid, m.p. 98–100 °C, yield 59 mg (57%). ^1^H NMR (CDCl_3_, 400.1 MHz): δ 8.03–7.92 (m, 3H), 7.75 (dd, 1H, ^3^*J* = 5.3 Hz, ^4^*J* = 1.4 Hz), 7.55–7.48 (m, 3H), 7.45 (dd, 2H, ^3^*J* = 5.3 Hz, ^4^*J* = 1.4 Hz). ^13^C{1H} NMR (CDCl_3_, 100.6 MHz): δ 174.3 (q, ^2^*J*_CF_ = 37.1 Hz), 152.8, 138.1, 136.6, 130.4, 129.2, 128.5, 127.9, 127.2, 120.6, 116.3 (q, ^1^*J*_CF_ = 290.7 Hz), 115.6. ^19^F NMR (CDCl_3_, 376.5 MHz): δ −74.9 (s, 3F). HRMS (ESI-TOF): *m*/*z* [M + H]^+^ Calcd for C_14_H_9_F_3_N_3_OS^+^: 324.0413; found: 324.0413.

**Synthesis of amides 21–24 (general procedure).** A 4 mL vial with a screw cap was charged with 1-(2,5-diзhenyl-2*H*-1,2,3-triazol-4-yl)-2,2,2-trifluoroethanone **(12)**, (50–59 mg, 0.158–0.186 mmol) and corresponding amine (190–230 mg, ~2.7 mmol, ~12 equiv.) The reaction mixture was heated for 8 h at 90 °C (for **21**) or at 110 °C (for **22–24**) using a magnetic stirrer with heating. The reaction mixture was transferred into a round-bottom 50 mL flask using CH_2_Cl_2_ (10–15 mL), and then volatiles were evaporated in vacuo. The residue was passed through a short silica gel pad using CH_2_Cl_2_ followed by CH_2_Cl_2_ -MeOH (100:1) as eluents. Evaporation of volatiles afforded corresponding pure amides **21–24**.

**(2,5-Diphenyl-2*H*-1,2,3-triazol-4-yl)(pyrrolidin-1-yl)methanone (21)**. This was obtained from **12** (58.6 mg, 0.185 mmol) and pyrrolidine (190 mg, 2.66 mmol) by heating at 90 °C for 8 h: white solid, m.p. 94–96 °C, yield 55 mg (94%). ^1^H NMR (CDCl_3_, 400.1 MHz): δ 8.14–8.11 (m, 2H), 7.96–7.93 (m, 2H), 7.50–7.34 (m, 6H), 3.71 (t, 2H, ^3^*J* = 6.9 Hz), 3.46 (t, 2H, ^3^*J* = 6.9 Hz), 1.97–1.82 (m, 4H). ^13^C{^1^H} NMR (CDCl_3_, 100.6 MHz): δ 161.5, 146.8, 141.1, 139.3, 129.5, 129.2, 129.0, 128.6, 127.8, 127.6, 118.9, 48.3, 46.2, 25.9, 24.2. HRMS (ESI-TOF): *m*/*z* [M + H]^+^ Calcd for C_19_H_19_N_4_O^+^: 319.1553; found: 319.1560.

**(2,5-Diphenyl-2*H*-1,2,3-triazol-4-yl)(piperidin-1-yl)methanone (22)**. This was obtained from **12** (50 mg, 0.158 mmol) and piperidine (205 mg, 2.41 mmol) by heating at 110 °C for 8 h: beige viscous oil, yield 37.4 mg (71%). ^1^H NMR (CDCl_3_, 400.1 MHz): δ 8.16–8.11 (m, 2H), 7.93–7.85 (m, 2H), 7.52–7.34 (m, 6H), 3.87–3.73 (m, 2H), 3.32–3.21 (m, 2H), 1.71–1.57 (m, 4H), 1.39–1.31 (m, 2H). ^13^C{^1^H} NMR (CDCl_3_, 100.6 MHz): δ 162.1, 146.1, 140.6, 139.4, 129.31, 129.26, 129.1, 128.8, 127.8, 127.3, 118.9, 48.2, 43.0, 26.1, 25.4, 24.4. HRMS (ESI-TOF): *m*/*z* [M + H]^+^ Calcd for C_20_H_21_N_4_O^+^: 333.1710; found: 333.1715.

**(2,5-Diphenyl-2*H*-1,2,3-triazol-4-yl)(morpholino)methanone (23)**. This was obtained from **12** (50 mg, 0.158 mmol) and morpholine (220 mg, 2.53 mmol) by heating at 110 °C for 8 h: beige solid, m.p. 140–142 °C, yield 37 mg (70%). ^1^H NMR (CDCl_3_, 400.1 MHz): δ 8.17–8.08 (m, 2H), 7.91–7.80 (m, 2H), 7.55–7.33 (m, 6H), 3.91–3.82 (m, 2H), 3.80–3.73 (m, 2H), 3.50–3.43 (m, 2H), 3.42–3.34 (m, 2H). ^13^C{^1^H} NMR (CDCl_3_, 100.6 MHz): δ 162.2, 146.8, 139.6, 139.3, 129.3, 129.1, 128.9, 128.0, 127.5, 119.0, 66.58, 66.54, 47.4, 42.5. HRMS (ESI-TOF): *m*/*z* [M + H]^+^ Calcd for C_19_H_19_N_4_O_2_^+^: 335.1503; found: 335.1506.

***N*-Hexyl-2,5-diphenyl-2*H*-1,2,3-triazole-4-carboxamide (24)**. This was obtained from **12** (53 mg, 0.167 mmol) and hexan-1-amine (240 mg, 2.38 mmol) by heating at 110 °C for 8 h: white solid, m.p. 86–88 °C, yield 25 mg (43%). ^1^H NMR (CDCl_3_, 400.1 MHz): δ 8.20–8.06 (m, 4H), 7.54–7.37 (m, 6H), 6.98 (t, 1H, ^3^*J* = 4.7 Hz), 3.46 (dd, 2H, ^3^*J* = 13.4 Hz, ^3^*J* = 7.1 Hz), 1.67–1.60 (m, 2H), 1.44–1.28 (m, 6H), 0.91–0.87 (m, 3H). ^13^C{^1^H} NMR (CDCl_3_, 100.6 MHz): δ 160.4, 139.5, 139.2, 129.38, 129.35, 129.2, 128.3, 128.2, 119.1, 39.5, 31.5, 29.6, 26.7, 22.5, 14.0. HRMS (ESI-TOF): *m*/*z* [M + H]^+^ Calcd for C_21_H_25_N_4_O ^+^: 349.2023; found: 349.2029.

## 4. Conclusions

In conclusion, we investigated modification of 5-aryl-4-trifluoroacetyl-1,2,3-triazoles at NH-moiety. We found that alkylation can be performed selectively in DMF using Na_2_CO_3_ as a base. The reaction proceeds at room temperature to produce selectively 2-isomers in high yields as major isomers. The selectivity of the reaction reaches a 94:6 ratio of 2- and 1-isomers. Activated by electron-withdrawing groups, aryl halides react regioselectively to form 2-aryltriazoles in good-to-high yields. Similarly, the copper catalyzed reaction with boronic acids led to 2-aryltriazoles exclusively. Transformation of the latter compounds into amides of 4-(2,5-diaryltriazolyl)carboxylic acid were achieved by heating with primary and secondary amines. Fluorescent properties of prepared 2-derivatives of 1,2,3-triazoles were investigated to reveal that some of them have quantum yields of more than 60%.

## Figures and Tables

**Table 1:** Photophysical data of compounds **3**.

Compd.	$\lambda_{max}^{abs}$$,\;\mathrm{nm}/{\tilde{v}}_{max}^{abs}$, cm^−1^	ε, L·mol^−1^·cm^−1^	$\lambda_{max}^{fl}$$,\;\mathrm{nm}/{\tilde{v}}_{max}^{fl}$, cm^−1^	φ ^a^	Δν_ST_, cm^−1^
**3a**	234/42,730	15,140	298/33,550	0.09	9180
**3d**	232/43,100	10,900	297/33,670	0.07	9430
**3e**	232/43,100	12,200	297/33,670	0.07	9430
**3p**	231/43,290	13,090	298/33,550	0.07	9740
**3q**	224/44,640	18,750	303/33,000	0.03	11,640
**3r**	230/43,470	11,700	299/33,440	0.03	10,030

^a^ Quantum yield φ relative to 2-aminopyridine in 0.1 M H_2_SO_4_ (φ = 0.6).

**Table 2:** Photophysical data of compounds **6–8, 10–20**.

Compd.	$\lambda_{max}^{abs}$ $,\;\mathrm{nm}/{\tilde{v}}_{max}^{abs},\;{{\mathrm{cm}}^{-1}}^{a}$	ε, L·mol^−1^· cm^−1^	$\lambda_{max}^{fl}$$,\;\mathrm{nm}/{\tilde{v}}_{max}^{fl}$, cm^−1^	φ ^b^	Δν_ST_, cm^−1^
**6**	222/45,040 316/31,640	11,370 17,880		—	
**7**	227/44,050 305/32,780	14,800 12,120		—	
**8**	278/35,970	15,240		—	
**10**	221/45,240 271/36,900	18,020 17,600	408/24,500	0.04	12,400
**11**	230/43,480 329/30,390	14,650 19,290		—	
**12**	276/36,230	17,420	335/29,850	0.52	6380
**13**	241/41,490 283/35,330	13,560 19,660	364/27,470	0.41	7860
**14**	279/35,840	20,630	339/29,490	0.60	6350
**15**	276/36,230	20,120	342/29,240	0.65	6990
**16**	287/34,840	16,580	366/27,320	0.59	7520
**17**	289/34,600	21,450	368/27,180	0.60	7420
**18**	291/34,360	22,860	338/29,580	0.57	4780
**19**	279/35,840	22,370	345/28,980	0.60	6860
**20**	226/44,240 278/35,970	14,620 19,300	347/28,810	0.23	7160

^a^ For compounds with two absorption bands, the positions of both bands are given, ^b^ quantum yield φ relative to 2-aminopyridine in 0.1 M H_2_SO_4_ (φ = 0.6).

**Table 3:** Photophysical data of compounds **21–24**.

Compd.	$\lambda_{max}^{abs}$$,\;\mathrm{nm}/{\tilde{v}}_{max}^{abs}$, cm^−1^	ε, L·mol^−1^· cm^−1^	$\lambda_{max}^{fl}$$,\;\mathrm{nm}/{\tilde{v}}_{max}^{fl}$, cm^−1^	φ ^a^	Δν_ST_, cm^−1^
**21**	290/34,480	29,740	342/29,240	0.03	5240
**22**	290/34,480	20,310	342/29,240	0.04	5240
**23**	289/34,600	21,450	340/29,410	0.02	5190
**24**	286/34,960	19,420	345/28,980	0.26	5980

^a^ Quantum yield φ relative to 2-aminopyridine in 0.1 M H_2_SO_4_ (φ = 0.6).

## Data Availability

The data presented in this study are available in Supplementary Materials.

## Supplementary Materials

The following supporting information can be downloaded at: https://www.mdpi.com/article/10.3390/molecules28124822/s1, Copies of all ^1^H, ^13^C and ^19^F NMR spectra.

## Sample Availability

Samples of the compounds **3–24** are available from the authors.
