# Supplementary material for: Regioselective Synthesis of New Family of 2-Substituted 1,2,3-Triazoles and Study of Their Fluorescent Properties
Source: Molecules. 2023 Jun 16;28(12):4822. doi: 10.3390/molecules28124822 (PMC10301629; doi:10.3390/molecules28124822)
Supplement: Supplementary file 1 [file molecules-28-04822-s001.zip › molecules-2441232-supplementary.pdf]

Supporting Information

Regioselective synthesis of new family of 2-substituted 1,2,3-triazoles and study of their fluorescent properties

Vasiliy M. Muzalevskiy, Zoia A. Sizova and Valentine G. Nenajdenko*

Department of Chemistry, Lomonosov Moscow State University, 119899 Moscow, Russia

* Correspondence: nenajdenko@org.chem.msu.ru (V.G.N.)

Copies of all NMR spectra **S2-S152**

18 May 2022

Acquisition Time (sec)	4.0894	***Comment***	Imported from UXNMR.			***Date***	29 Mar 2022 14:45:06
File Name	C:\DOCS\OUTPUT_301\2022\03.март\SZA-BM-2448-7.H.H_001001r					***Frequency (MHz)***	400.13
Nucleus	1H	***Number of Transients***	4	***Original Points Count***	32768	***Points Count***	131072
Pulse Sequence	zg30	***Solvent***	CHLOROFORM-D			***Sweep Width (Hz)***	8012.82
Temperature (degree C)	27.000						

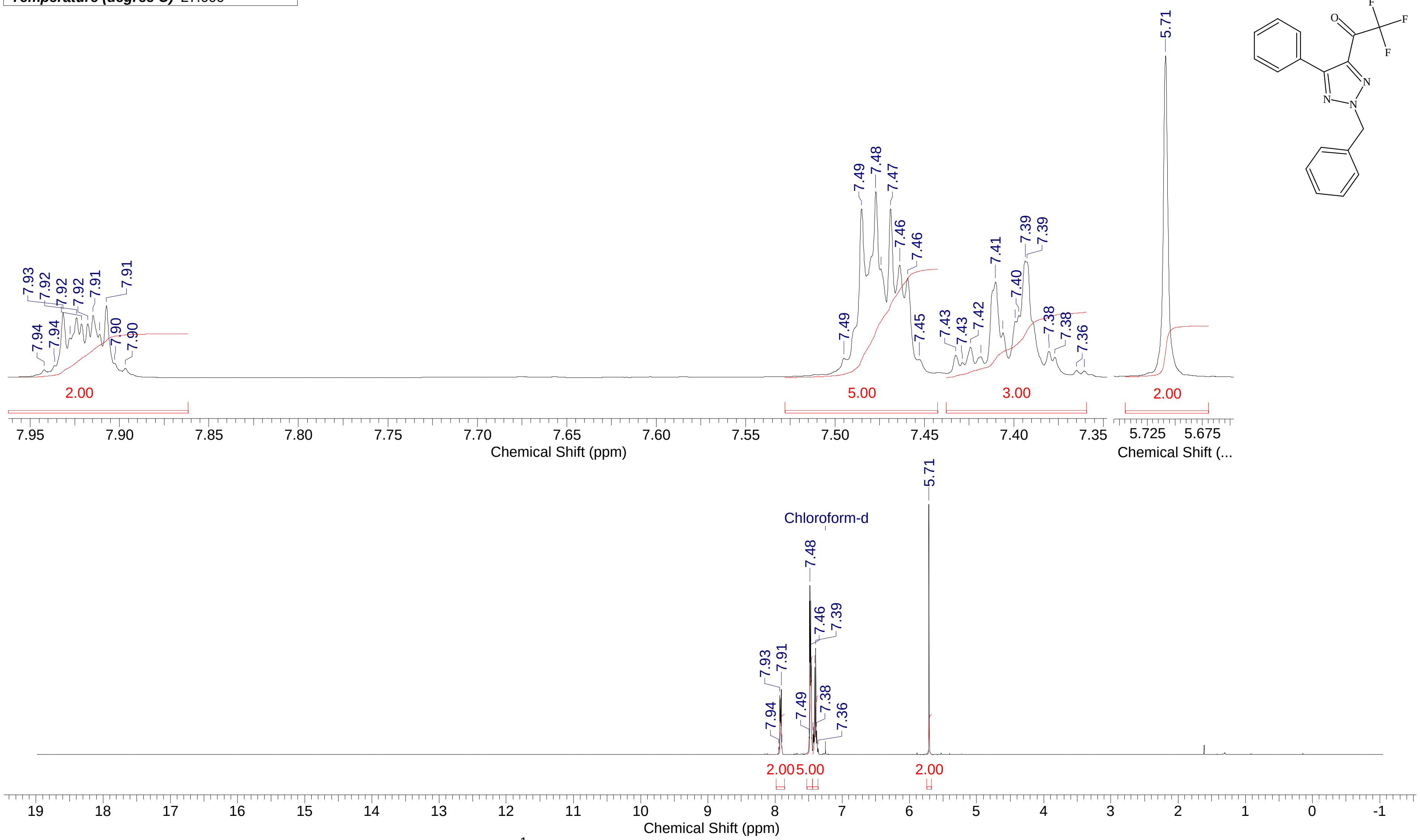

^{1}H NMR spectrum of **3a** (400.1 MHz, $CDCl_3$)

18 May 2022

Acquisition Time (sec)	1.7433	Comment	Imported from UXNMR.			Date	29 Mar 2022 14:56:42
File Name	C:\DOCS\OUTPUT_301\2022\03.март\SZA-BM-2448-7.F_005001r					Frequency (MHz)	376.50
Nucleus	19F	Number of Transients	4	Original Points Count	131072	Points Count	262144
Pulse Sequence	zgflqn	Solvent	CHLOROFORM-D			Sweep Width (Hz)	75187.97
Temperature (degree C)	27.000						

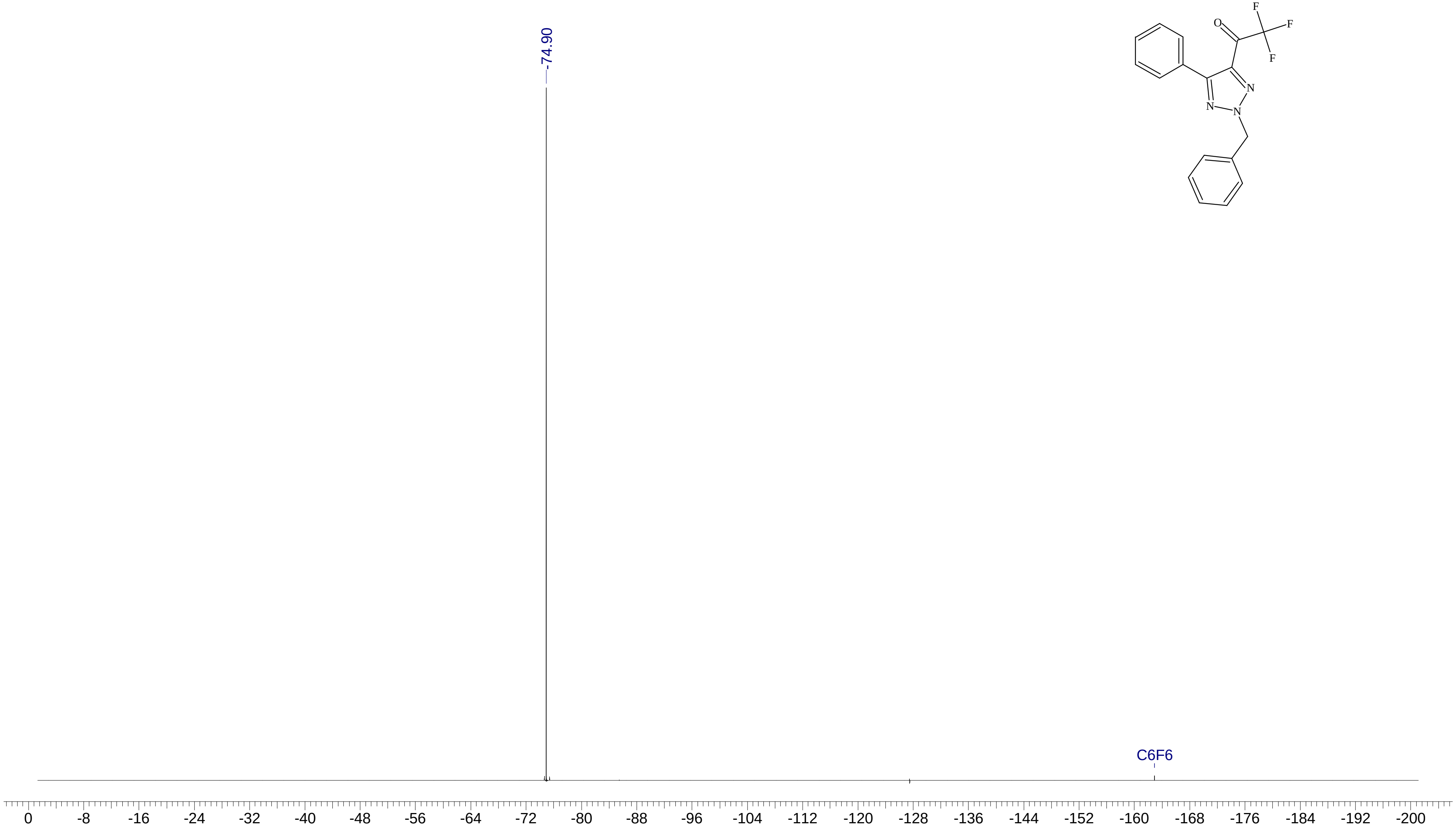

^{19}F NMR spectrum of **3a** (376.5 MHz, $CDCl_3$)

18 May 2022

Acquisition Time (sec)	0.6783	***Comment***	Imported from UXNMR.			***Date***	31 Mar 2022 12:08:00
File Name	C:\DOCS\OUTPUT_301\2022\03.март\SZA-BM-2448-7.C_002001r					***Frequency (MHz)***	100.61
Nucleus	13C	***Number of Transients***	73	***Original Points Count***	16384	***Points Count***	131072
Pulse Sequence	zgpg30	***Solvent***	DMSO-D6	***Sweep Width (Hz)***	24154.59	***Temperature (degree C)***	27.000

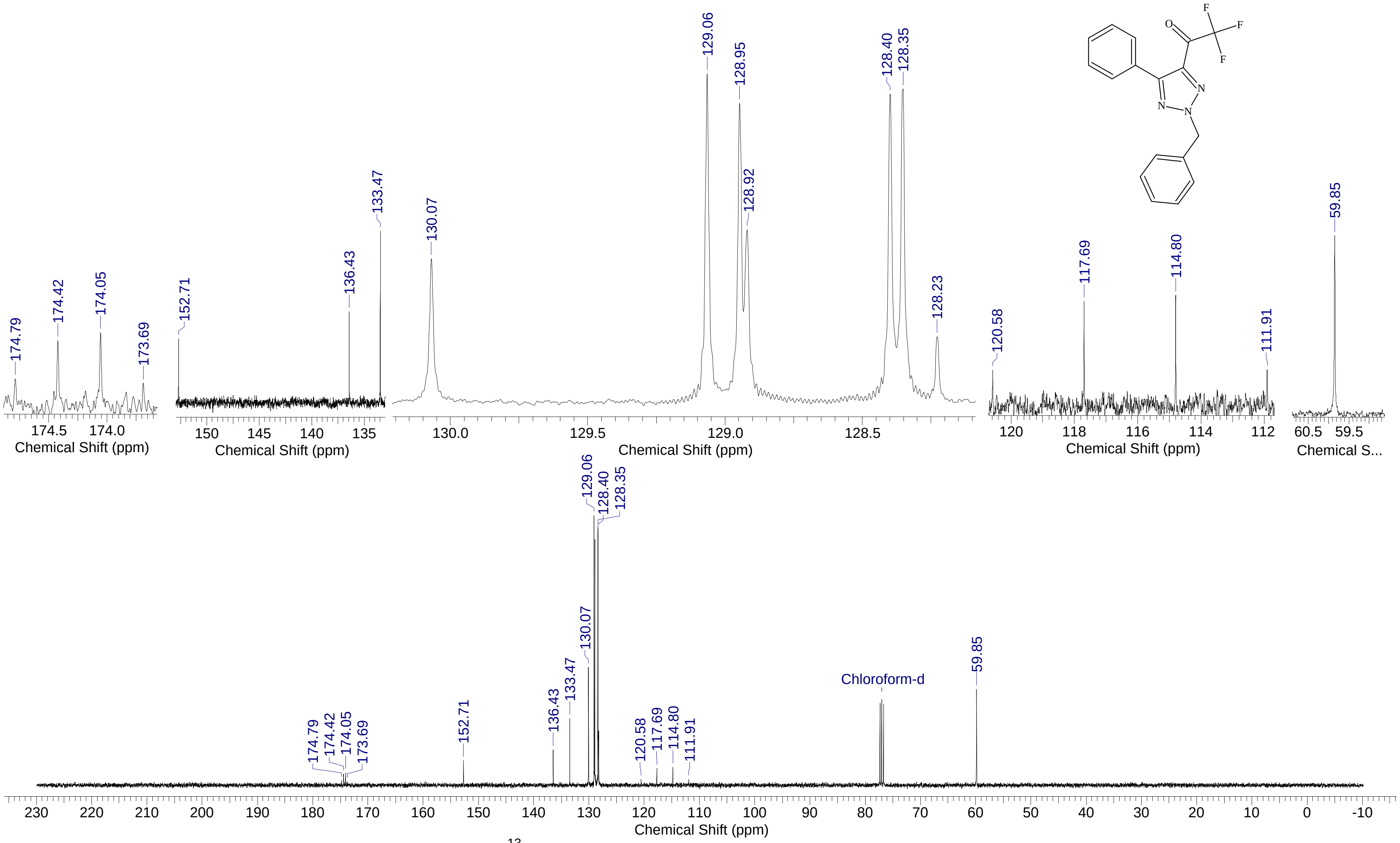

^{13}C NMR spectrum of **3a** (100.6 MHz, $CDCl_3$)

18 May 2022

Acquisition Time (sec)	4.0894	***Comment***	Imported from UXNMR.			***Date***	30 Mar 2022 17:43:12
File Name	C:\DOCS\OUTPUT_301\2022\03.март\SZA-BM-2448-25.H_001001r					***Frequency (MHz)***	400.13
Nucleus	1H	***Number of Transients***	4	***Original Points Count***	32768	***Points Count***	131072
Pulse Sequence	zg30	***Solvent***	CHLOROFORM-D			***Sweep Width (Hz)***	8012.82
Temperature (degree C)	27.000						

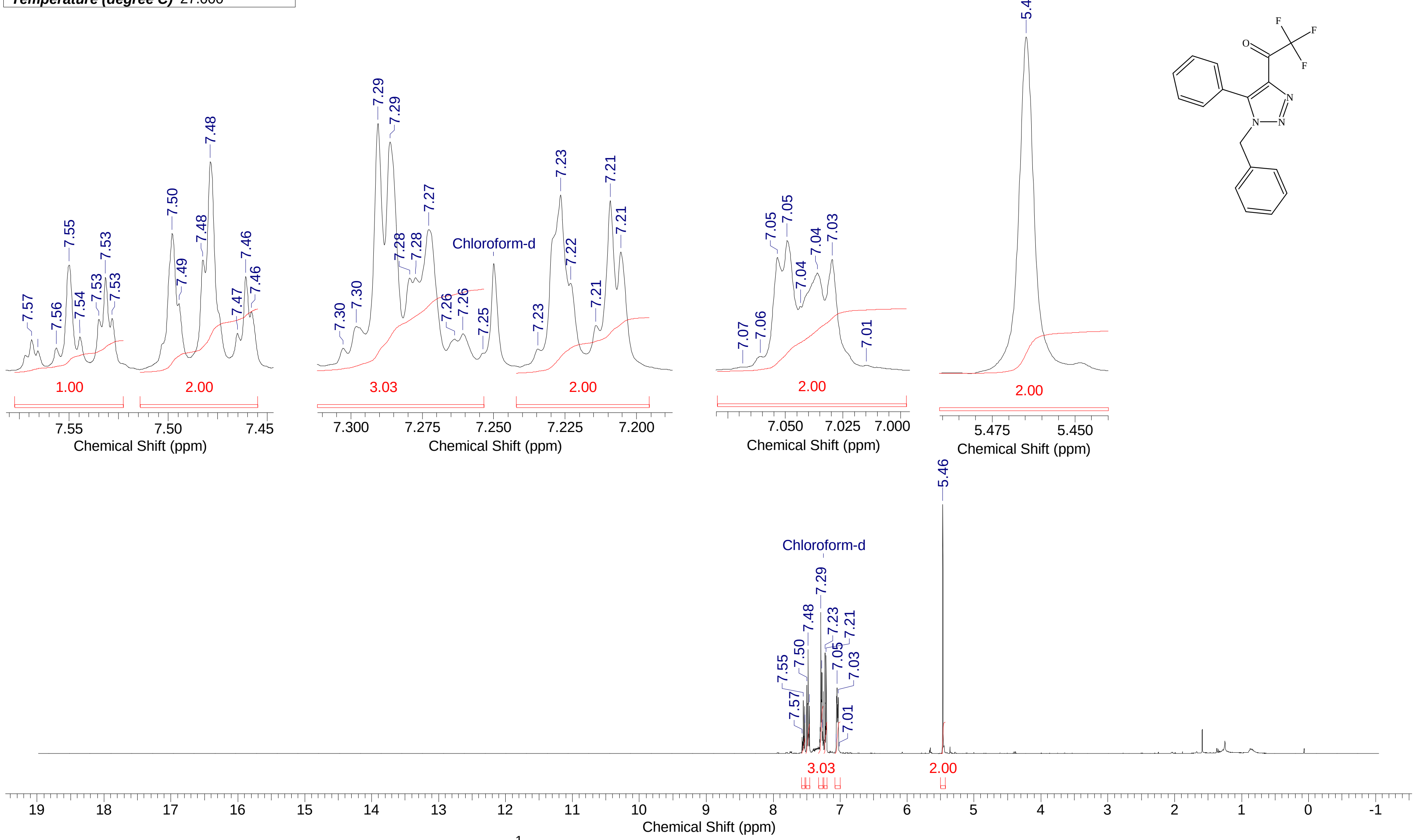

^{1}H NMR spectrum of **4a** (400.1 MHz, $CDCl_3$)

18 May 2022

Acquisition Time (sec)	1.7433	***Comment***	Imported from UXNMR.			***Date***	29 Mar 2022 15:15:50
File Name	C:\DOCS\OUTPUT_301\2022\03.март\SZA-BM-2448-21.F_005001r					***Frequency (MHz)***	376.50
Nucleus	19F	***Number of Transients***	4	***Original Points Count***	131072	***Points Count***	262144
Pulse Sequence	zgflqn	***Solvent***	CHLOROFORM-D			***Sweep Width (Hz)***	75187.97
Temperature (degree C)	27.000						

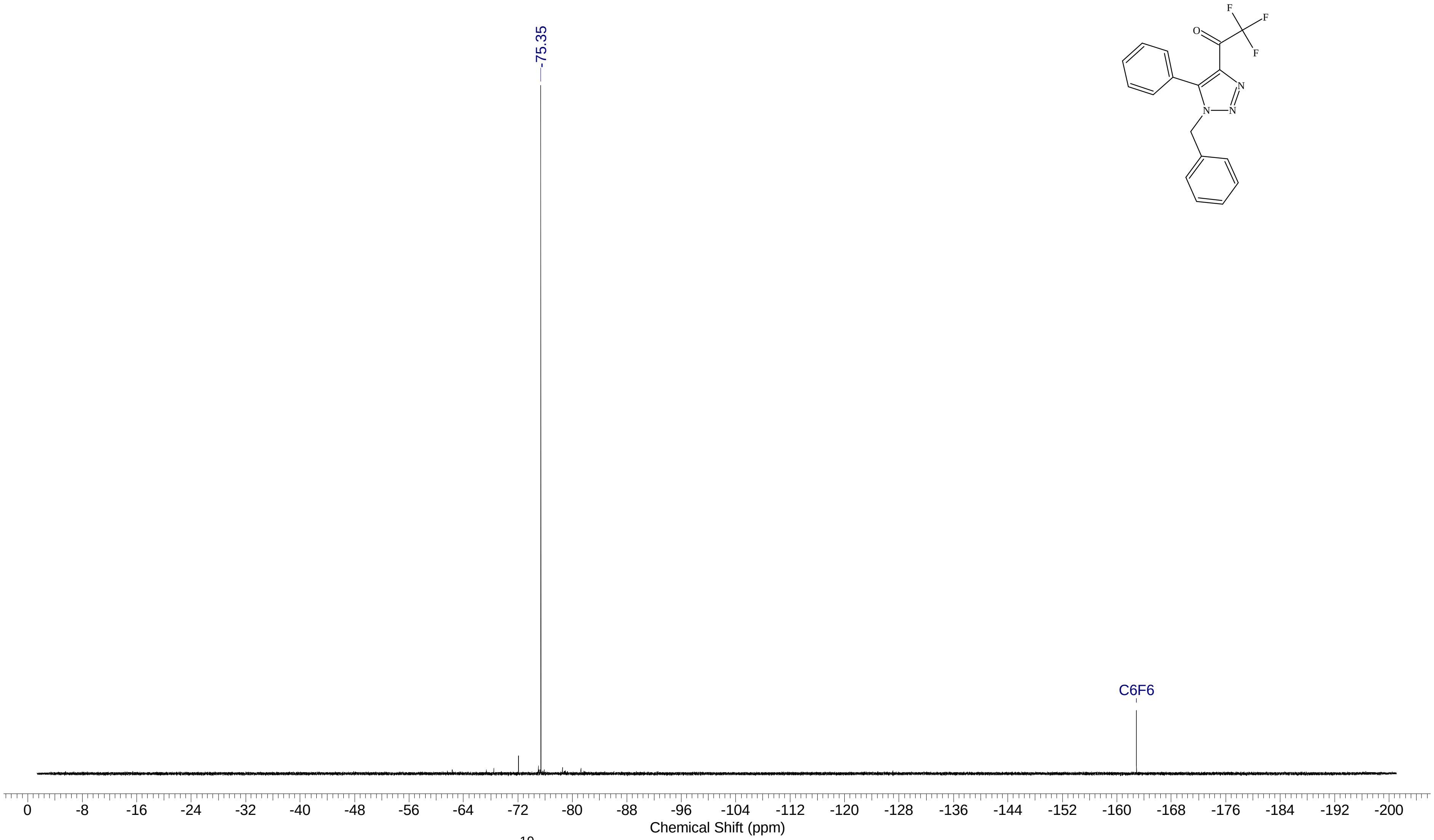

^{19}F NMR spectrum of **4a** (376.5 MHz, $CDCl_3$)

18 May 2022

Acquisition Time (sec)	0.6783	Comment	Imported from UXNMR.			Date	31 Mar 2022 15:24:40
File Name	C:\DOCS\OUTPUT_301\2022\03.март\SZA-BM-2448-25.C_002001r					Frequency (MHz)	100.61
Nucleus	13C	Number of Transients	347	Original Points Count	16384	Points Count	131072
Pulse Sequence	zgpg30	Solvent	DMSO-D6	Sweep Width (Hz)	24154.59	Temperature (degree C)	27.000

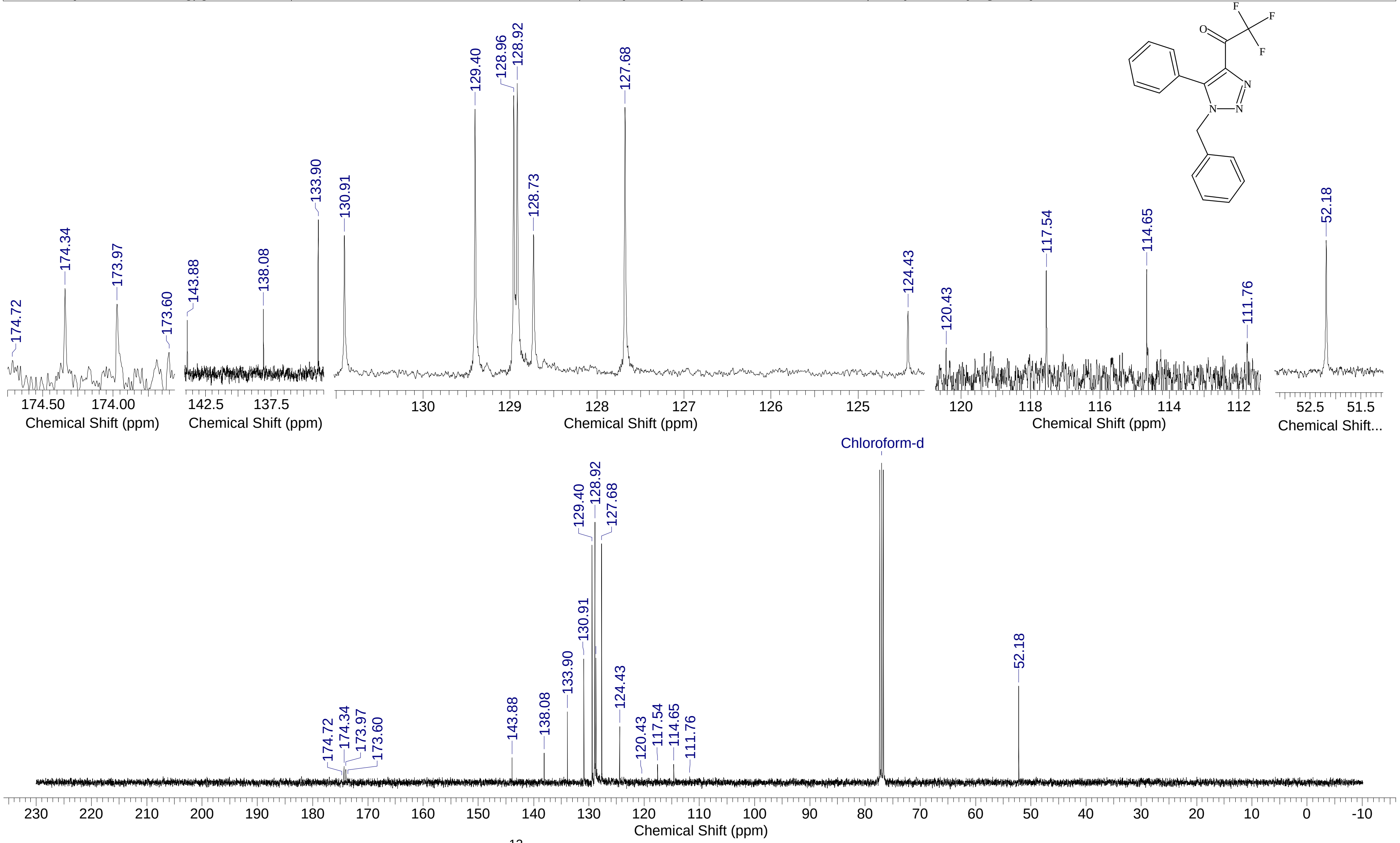

^{13}C NMR spectrum of **4a** (100.6 MHz, $CDCl_3$)

31 May 2022

Acquisition Time (sec)	4.0894	Comment	Imported from UXNMR.			Date	31 May 2022 15:08:20
File Name	C:\DOCS\OUTPUT_301\2022\05.май\BM-2513-1.H_001001r			Frequency (MHz)	400.13	Nucleus	1H
Number of Transients	4	Original Points Count	32768	Points Count	131072	Pulse Sequence	zg30
Solvent	CHLOROFORM-D			Sweep Width (Hz)	8012.82	Temperature (degree C)	27.000

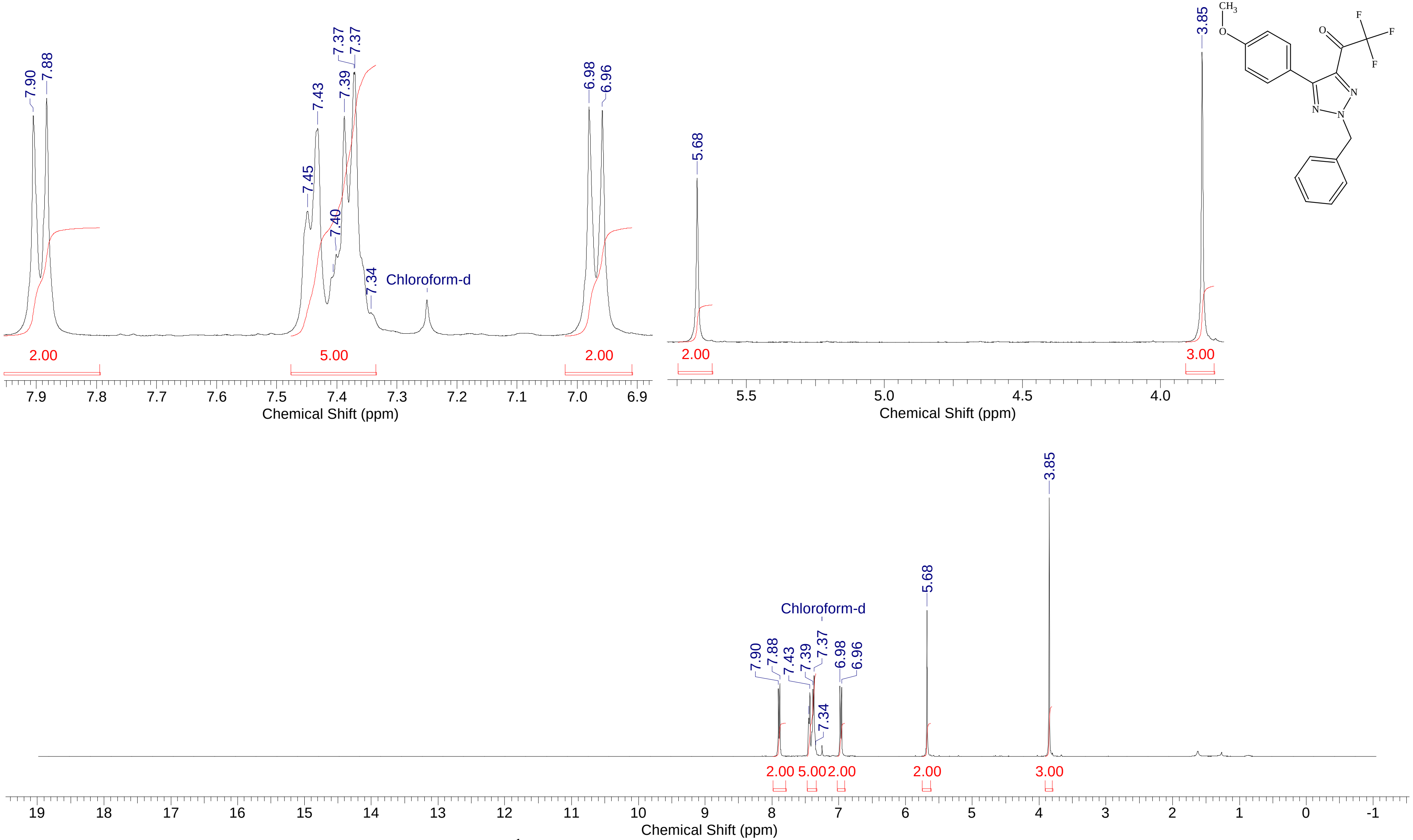

^{1}H NMR spectrum of **3b** (400.1 MHz, $CDCl_3$)

Acquisition Time (sec)	1.7433	Comment	Imported from UXNMR.			Date	31 May 2022 15:21:54
File Name	C:\DOCS\OUTPUT_301\2022\05.май\BM-2513-1.F_005001r			Frequency (MHz)	376.50	Nucleus	19F
Number of Transients	16	Original Points Count	131072	Points Count	262144	Pulse Sequence	zgflqn
Solvent	CHLOROFORM-D			Sweep Width (Hz)	75187.97	Temperature (degree C)	27.000

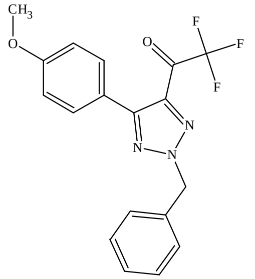

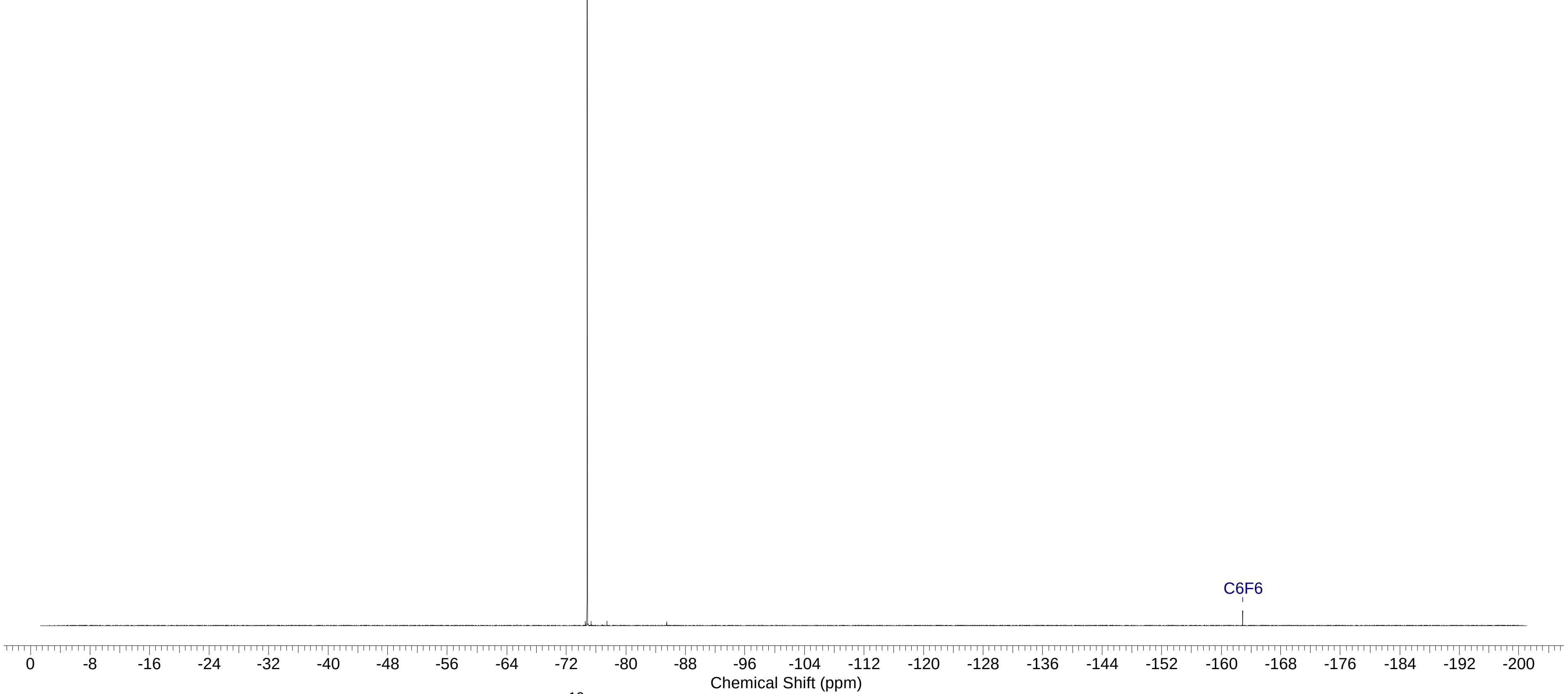

^{19}F NMR spectrum of **3b** (376.5 MHz, $CDCl_3$)

31 May 2022

Acquisition Time (sec)	0.6783	***Comment***	Imported from UXNMR.	***Date***	31 May 2022 15:17:28
File Name	C:\DOCS\OUTPUT_301\2022\05.май\BM-2513-1.C_002001r	***Frequency (MHz)***	100.61	***Nucleus***	13C
Number of Transients	225	***Original Points Count*** 16384	***Points Count*** 131072	***Pulse Sequence***	zgpg30
Solvent	CHLOROFORM-D	***Sweep Width (Hz)***	24154.59	***Temperature (degree C)***	27.000

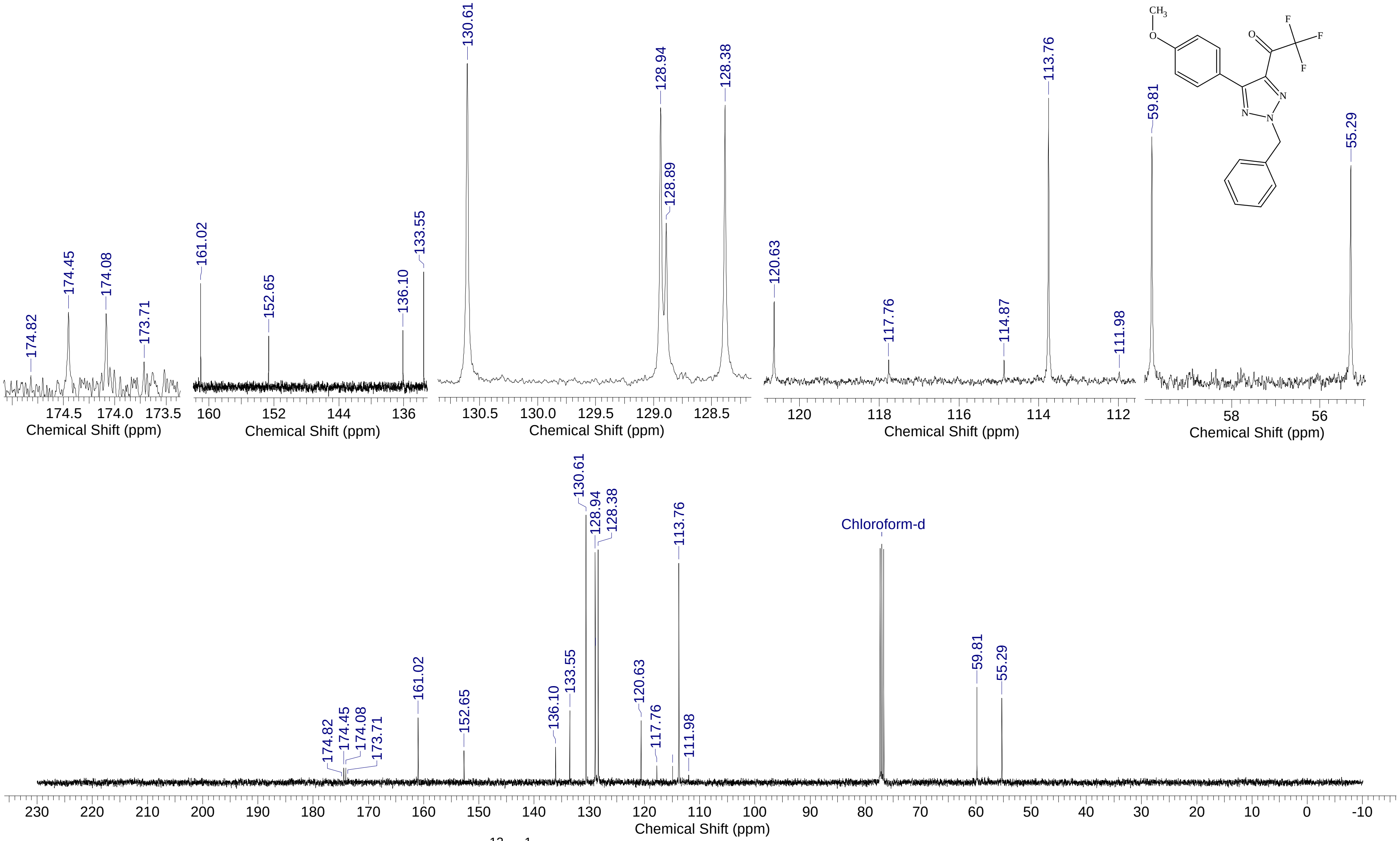

$^{13}C\{^{1}H\}$ NMR spectrum of **3b** (100.6 MHz, $CDCl_3$)

31 May 2022

Acquisition Time (sec)	4.0894	***Comment***	Imported from UXNMR.			***Date***	31 May 2022 15:36:48
File Name	C:\DOCS\OUTPUT_301\2022\05.май\BM-2513-3.H_001001r			***Frequency (MHz)***	400.13	***Nucleus***	1H
Number of Transients	4	***Original Points Count***	32768	***Points Count***	131072	***Pulse Sequence***	zg30
Solvent	CHLOROFORM-D			***Sweep Width (Hz)***	8012.82	***Temperature (degree C)***	27.000

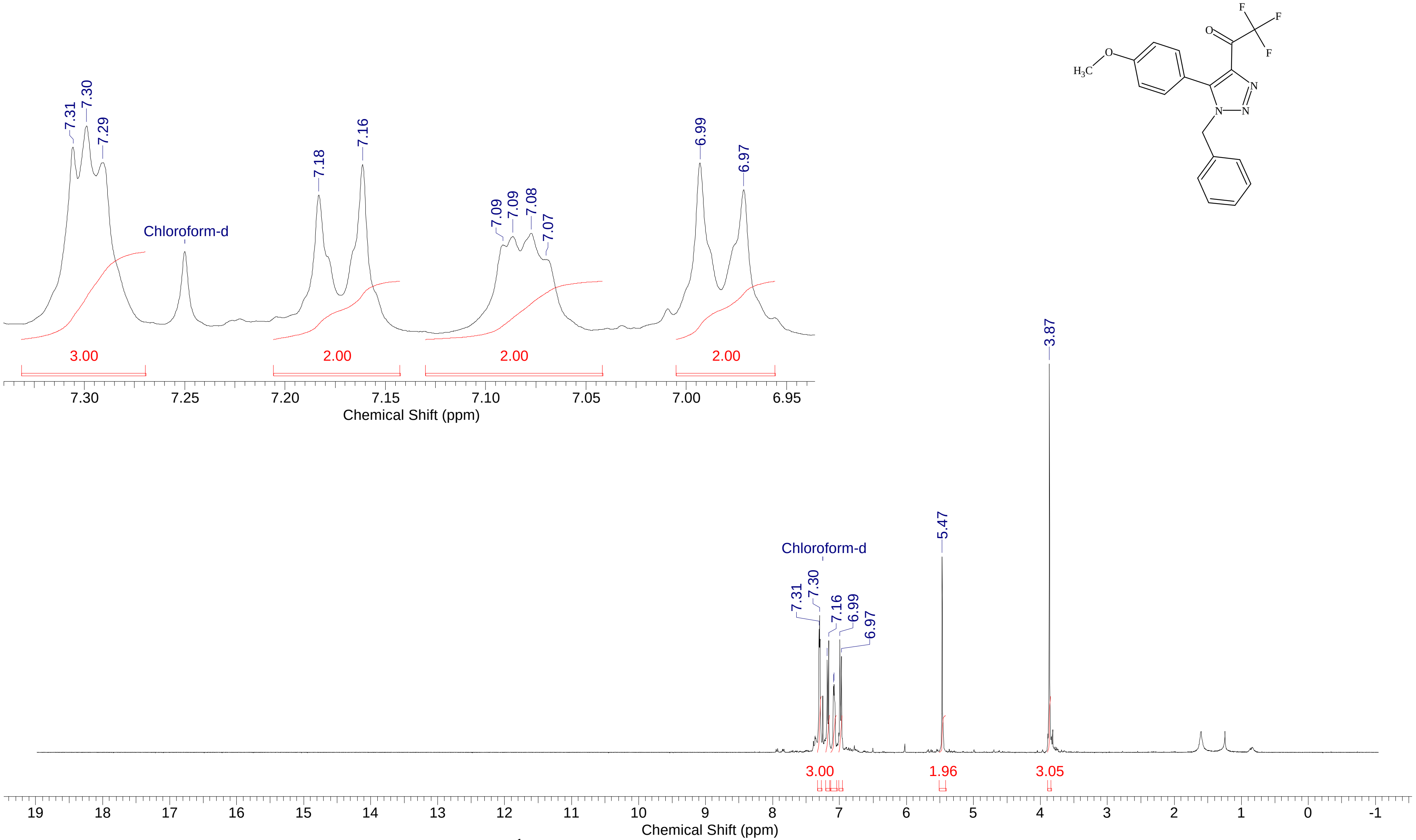

^{1}H NMR spectrum of **4b** (400.1 MHz, $CDCl_3$)

31 May 2022

Acquisition Time (sec)	1.7433	Comment	Imported from UXNMR.			Date	31 May 2022 15:27:58
File Name	C:\DOCS\OUTPUT_301\2022\05.май\BM-2513-3.F_005001r			Frequency (MHz)	376.50	Nucleus	19F
Number of Transients	16	Original Points Count	131072	Points Count	262144	Pulse Sequence	zgflqn
Solvent	CHLOROFORM-D			Sweep Width (Hz)	75187.97	Temperature (degree C)	27.000

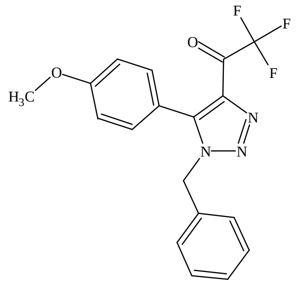

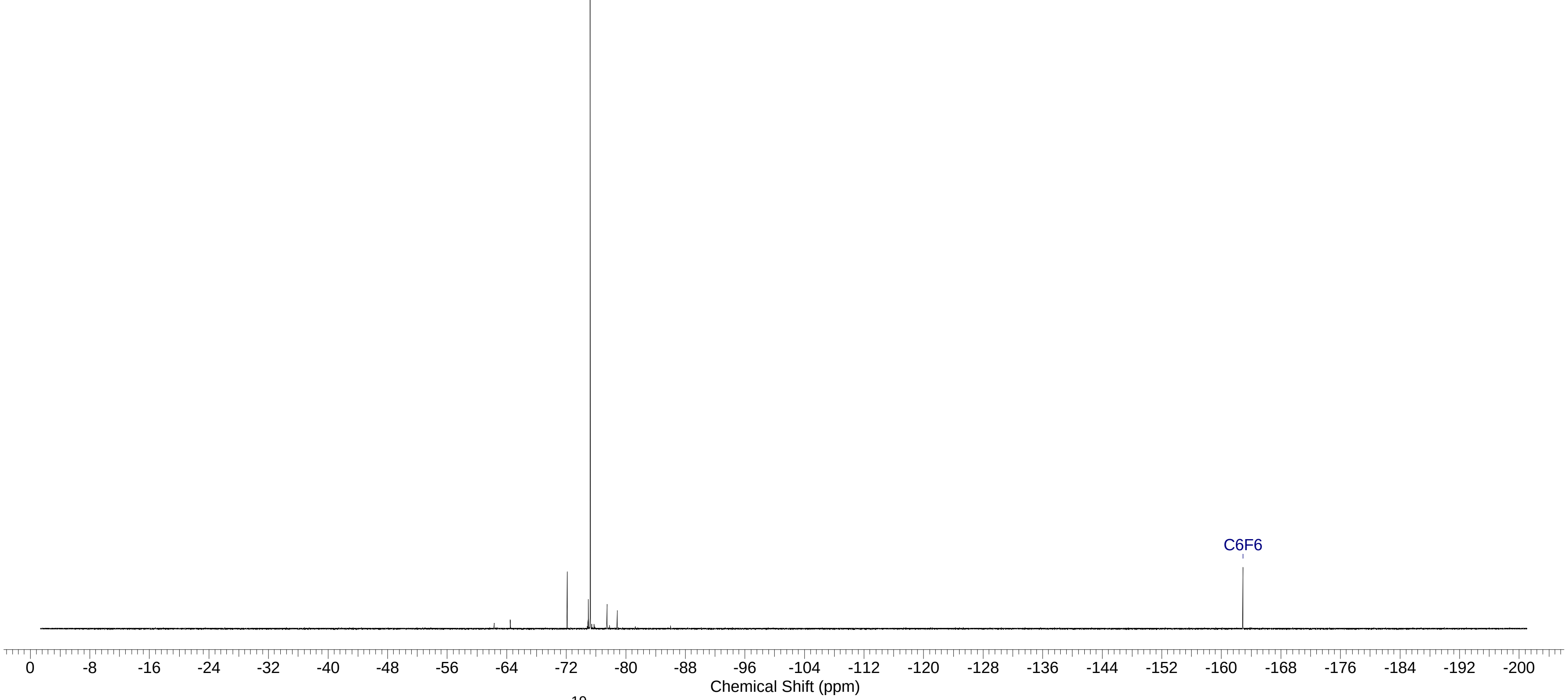

^{19}F NMR spectrum of **4b** (376.5 MHz, $CDCl_3$)

Acquisition Time (sec)	0.6783	Comment	Imported from UXNMR.			Date	31 May 2022 16:02:26
File Name	C:\DOCS\OUTPUT_301\2022\05.май\BM-2513-3.C_002001r			Frequency (MHz)	100.61	Nucleus	13C
Number of Transients	668	Original Points Count	16384	Points Count	131072	Pulse Sequence	zgpg30
Solvent	CHLOROFORM-D			Sweep Width (Hz)	24154.59	Temperature (degree C)	27.000

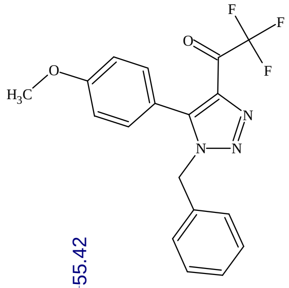

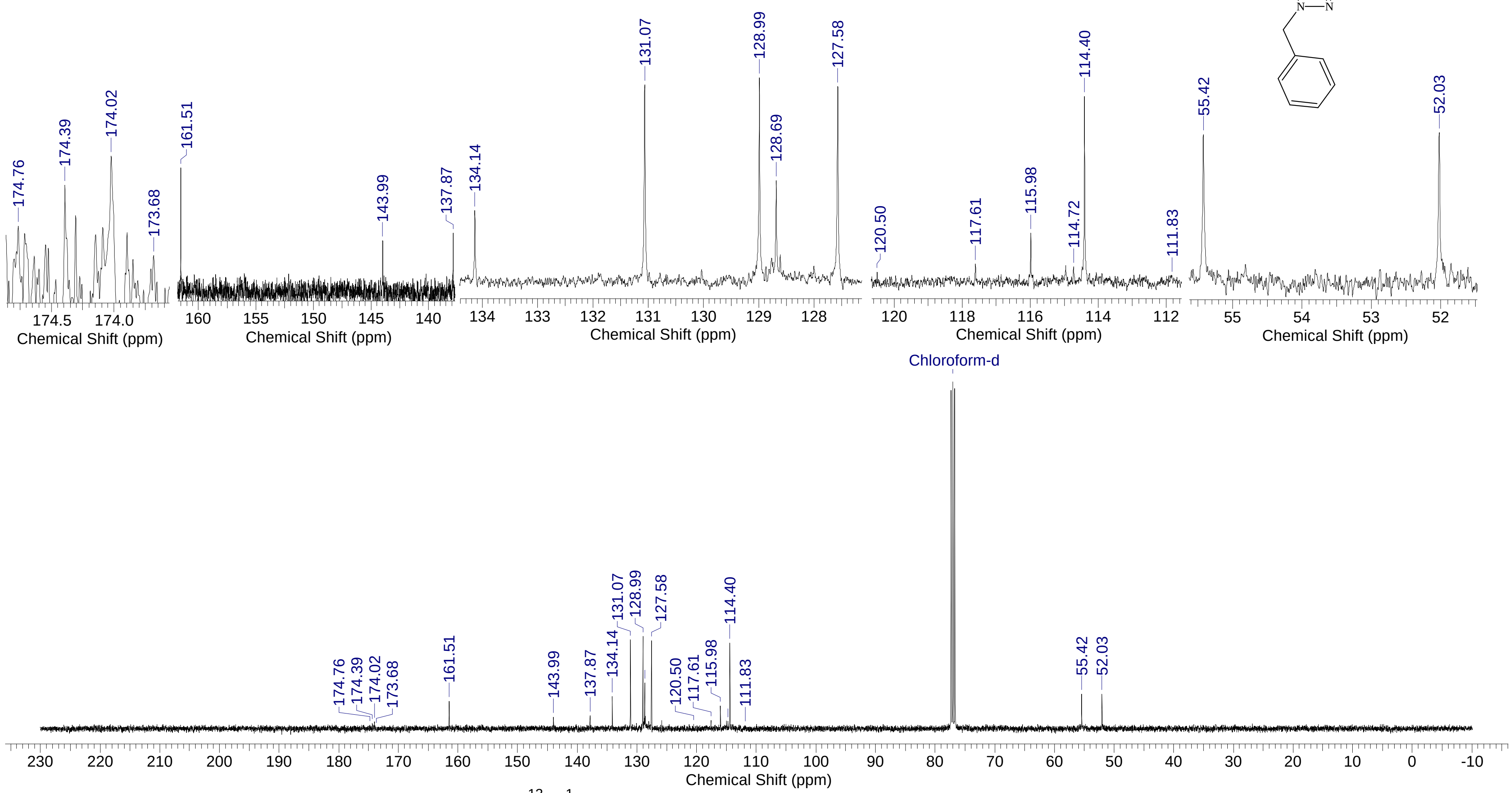

$^{13}C\{^{1}H\}$ NMR spectrum of **4b** (100.6 MHz, $CDCl_3$)

18 May 2022

Acquisition Time (sec)	4.0894	***Comment***	Imported from UXNMR.			***Date***	23 Mar 2022 17:27:40
File Name	C:\DOCS\OUTPUT_301\2022\03.март\SZA-BM-2463-15.H_001001r					***Frequency (MHz)***	400.13
Nucleus	1H	***Number of Transients***	4	***Original Points Count***	32768	***Points Count***	131072
Pulse Sequence	zg30	***Solvent***	BENZENE-D6	***Sweep Width (Hz)***	8012.82	***Temperature (degree C)***	27.000

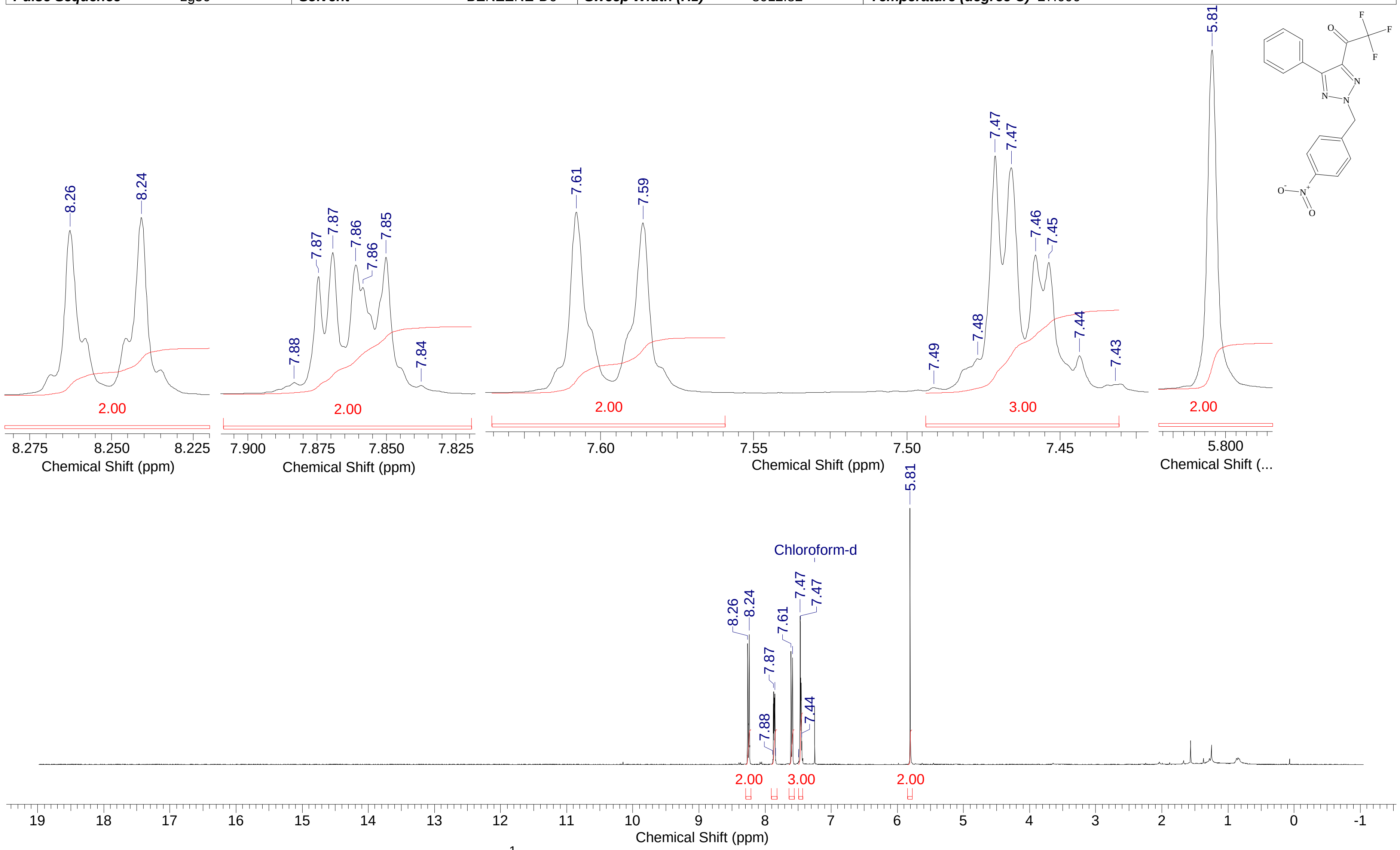

^{1}H NMR spectrum of **3c** (400.1 MHz, $CDCl_3$)

18 May 2022

Acquisition Time (sec)	1.7433	***Comment***	Imported from UXNMR.			***Date***	23 Mar 2022 17:49:42
File Name	C:\DOCS\OUTPUT_301\2022\03.март\SZA-BM-2463-15.F_005001r					***Frequency (MHz)***	376.50
Nucleus	19F	***Number of Transients***	16	***Original Points Count***	131072	***Points Count***	262144
Pulse Sequence	zgflqn	***Solvent***	CHLOROFORM-D			***Sweep Width (Hz)***	75187.97
Temperature (degree C)	27.000						

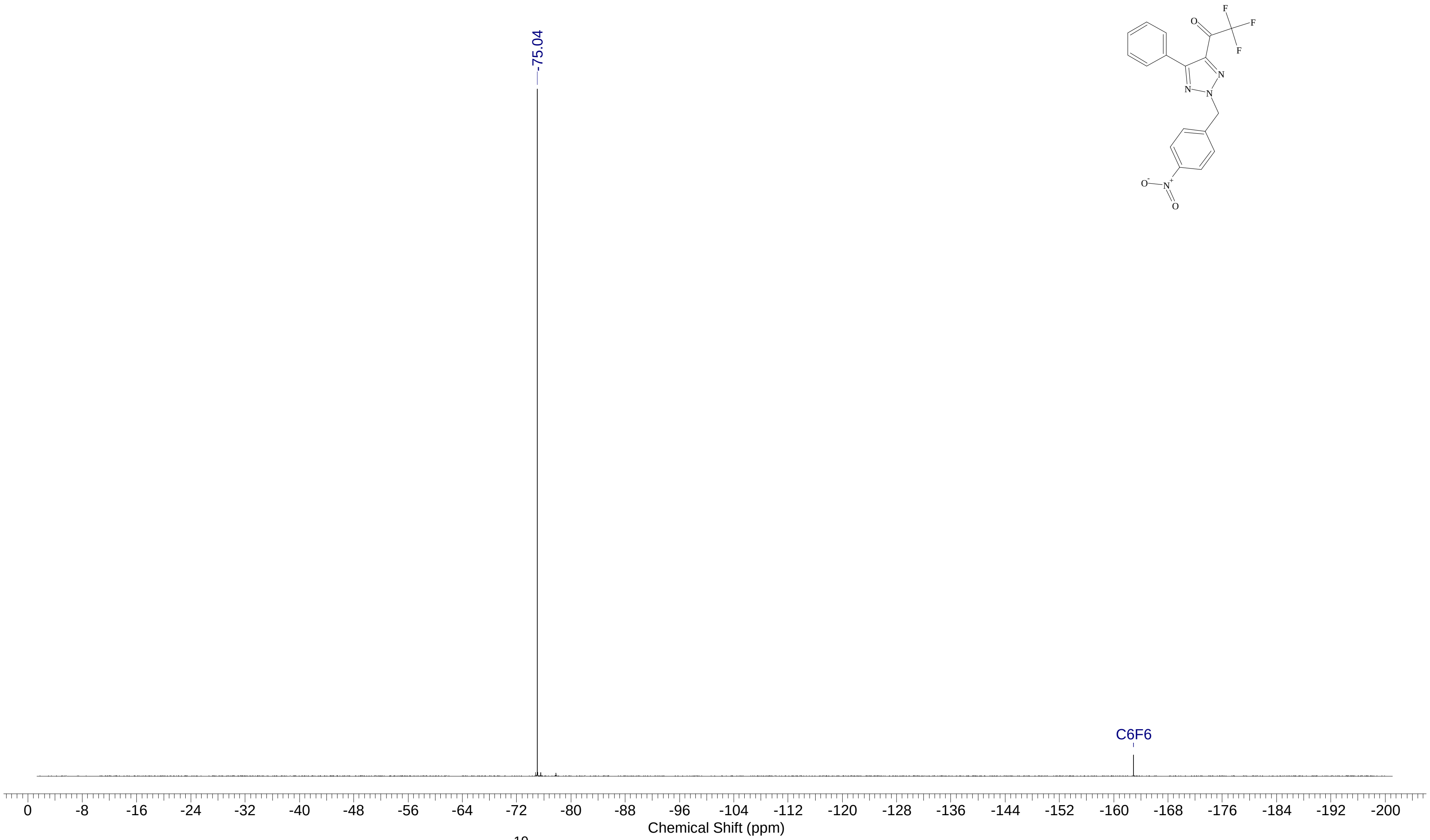

^{19}F NMR spectrum of **3c** (376.5 MHz, $CDCl_3$)

18 May 2022

Acquisition Time (sec)	0.6783	Comment	Imported from UXNMR.			Date	24 Mar 2022 13:21:38
File Name	C:\DOCS\OUTPUT_301\2022\03.март\SZA-BM-2463-15.C_002001r					Frequency (MHz)	100.61
Nucleus	13C	Number of Transients	353	Original Points Count	16384	Points Count	131072
Pulse Sequence	zgpg30	Solvent	DMSO-D6	Sweep Width (Hz)	24154.59	Temperature (degree C)	27.000

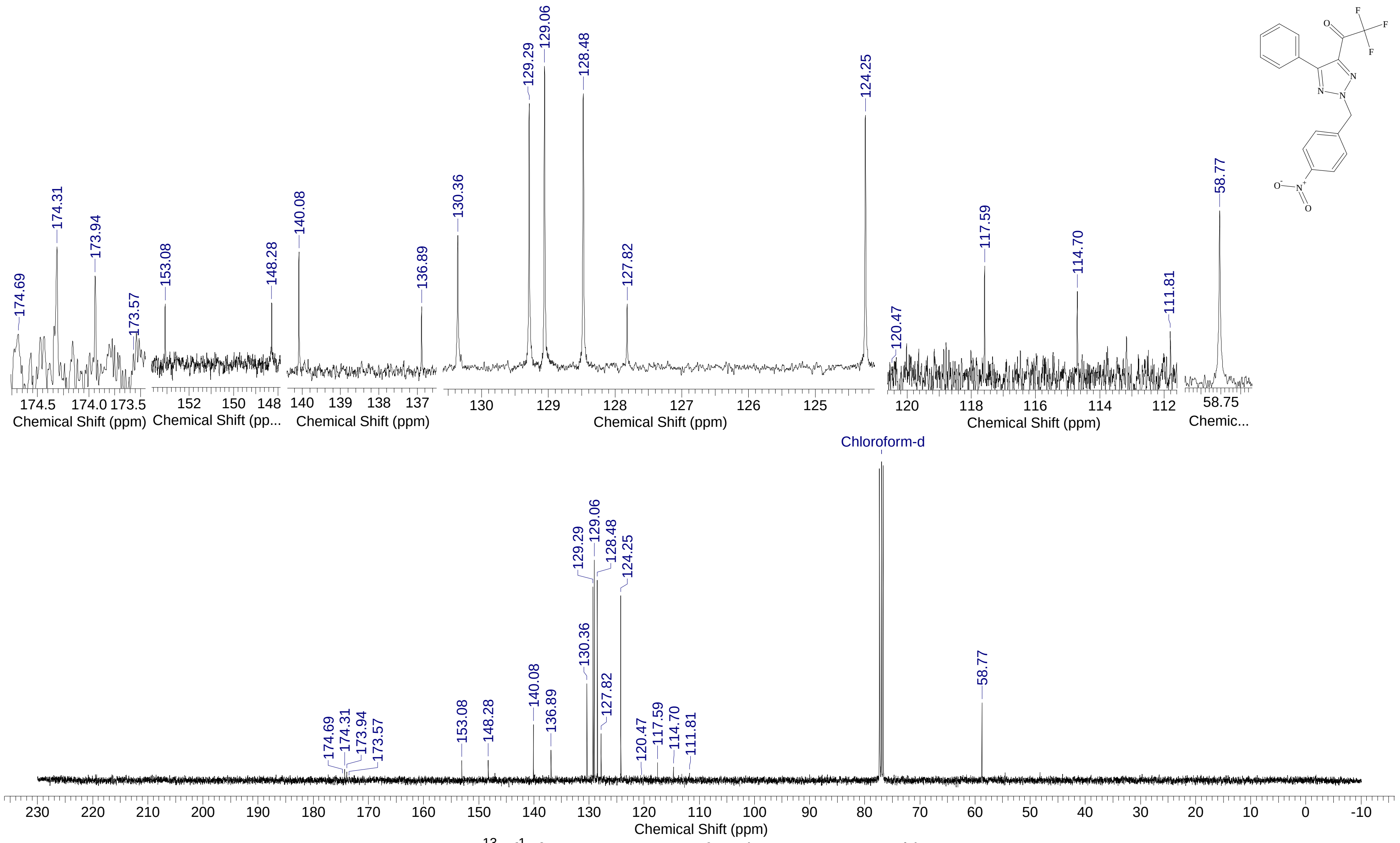

$^{13}C\{^1H\}$ NMR spectrum of **3c** (100.6 MHz, $CDCl_3$)

18 May 2022

Acquisition Time (sec)	4.0894	***Comment***	Imported from UXNMR.			***Date***	21 Mar 2022 15:24:14
File Name	C:\DOCS\OUTPUT_301\2022\03.март\SZA-BM-2463.H_001001r					***Frequency (MHz)***	400.13
Nucleus	1H	***Number of Transients***	4	***Original Points Count***	32768	***Points Count***	131072
Pulse Sequence	zg30	***Solvent***	CHLOROFORM-D			***Sweep Width (Hz)***	8012.82
Temperature (degree C)	27.000						

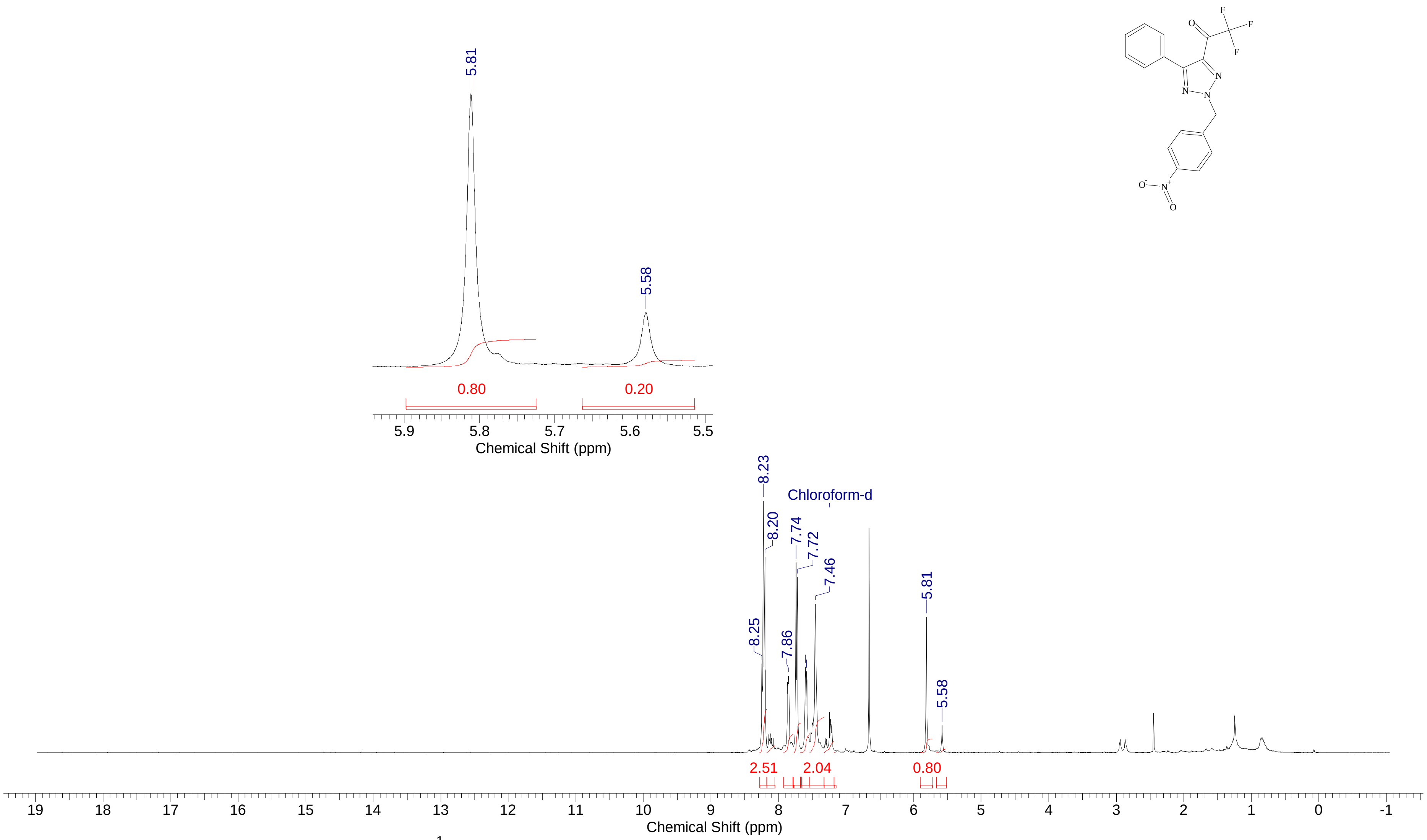

^{1}H NMR spectrum of mixture of **3c** and **4c** (400.1 MHz, $CDCl_3$)

18 May 2022

Acquisition Time (sec)	1.7433	***Comment***	Imported from UXNMR.			***Date***	21 Mar 2022 15:35:50
File Name	C:\DOCS\OUTPUT_301\2022\03.март\SZA-BM-2463.F_005001r					***Frequency (MHz)***	376.50
Nucleus	19F	***Number of Transients***	16	***Original Points Count***	131072	***Points Count***	262144
Pulse Sequence	zgflqn	***Solvent***	CHLOROFORM-D			***Sweep Width (Hz)***	75187.97
Temperature (degree C)	27.000						

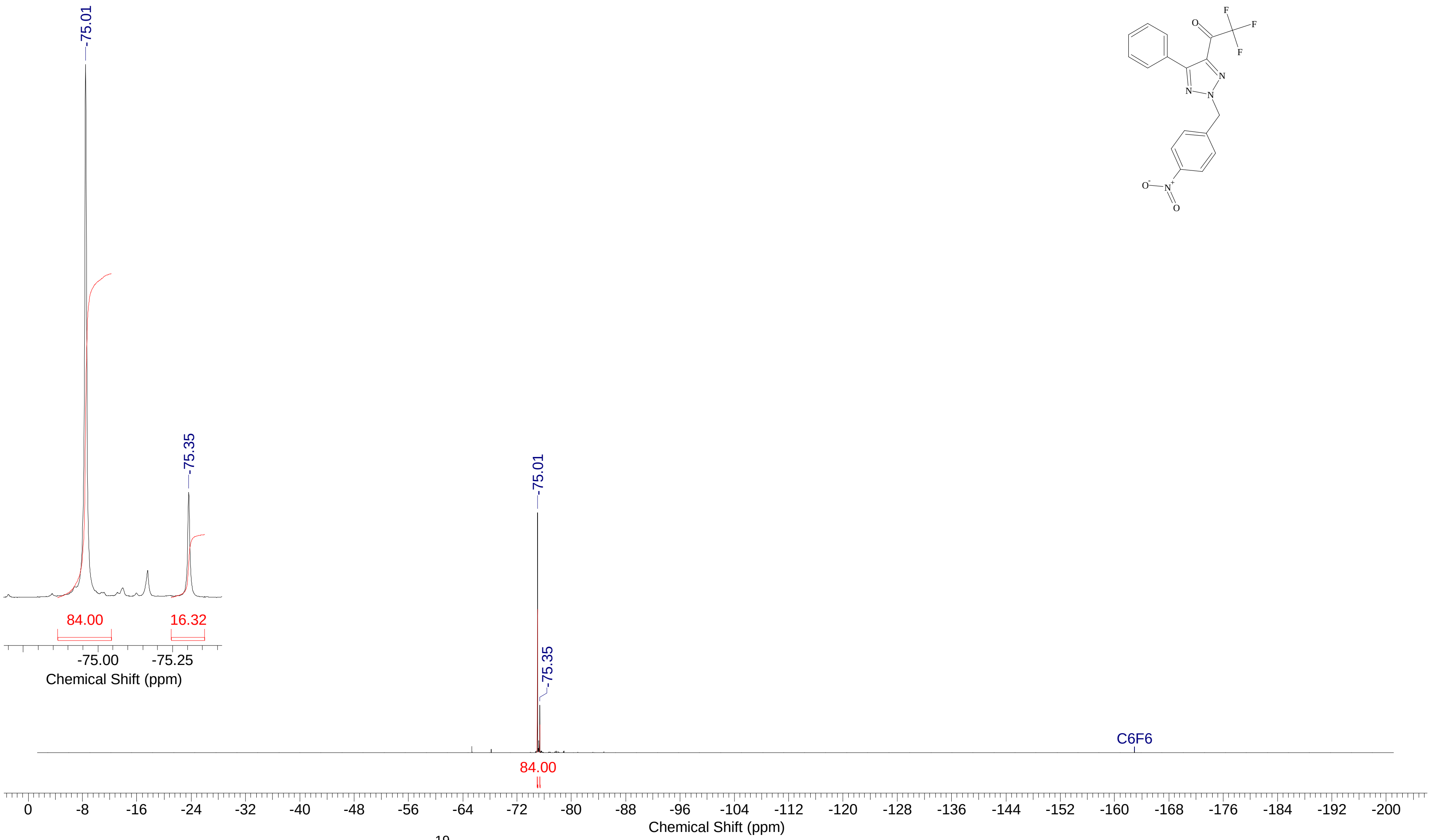

^{19}F NMR spectrum of mixture of **3c** and **4c** (376.5 MHz, $CDCl_3$)

17 May 2022

Acquisition Time (sec)	4.0894	Comment	Imported from UXNMR.			Date	07 Apr 2022 11:55:52
File Name	C:\DOCS\OUTPUT_301\2022\04.апрель\BM-2477-1.H_001001r					Frequency (MHz)	400.13
Nucleus	1H	Number of Transients	4	Original Points Count	32768	Points Count	131072
Pulse Sequence	zg30	Solvent	CHLOROFORM-D			Sweep Width (Hz)	8012.82
Temperature (degree C)	27.000						

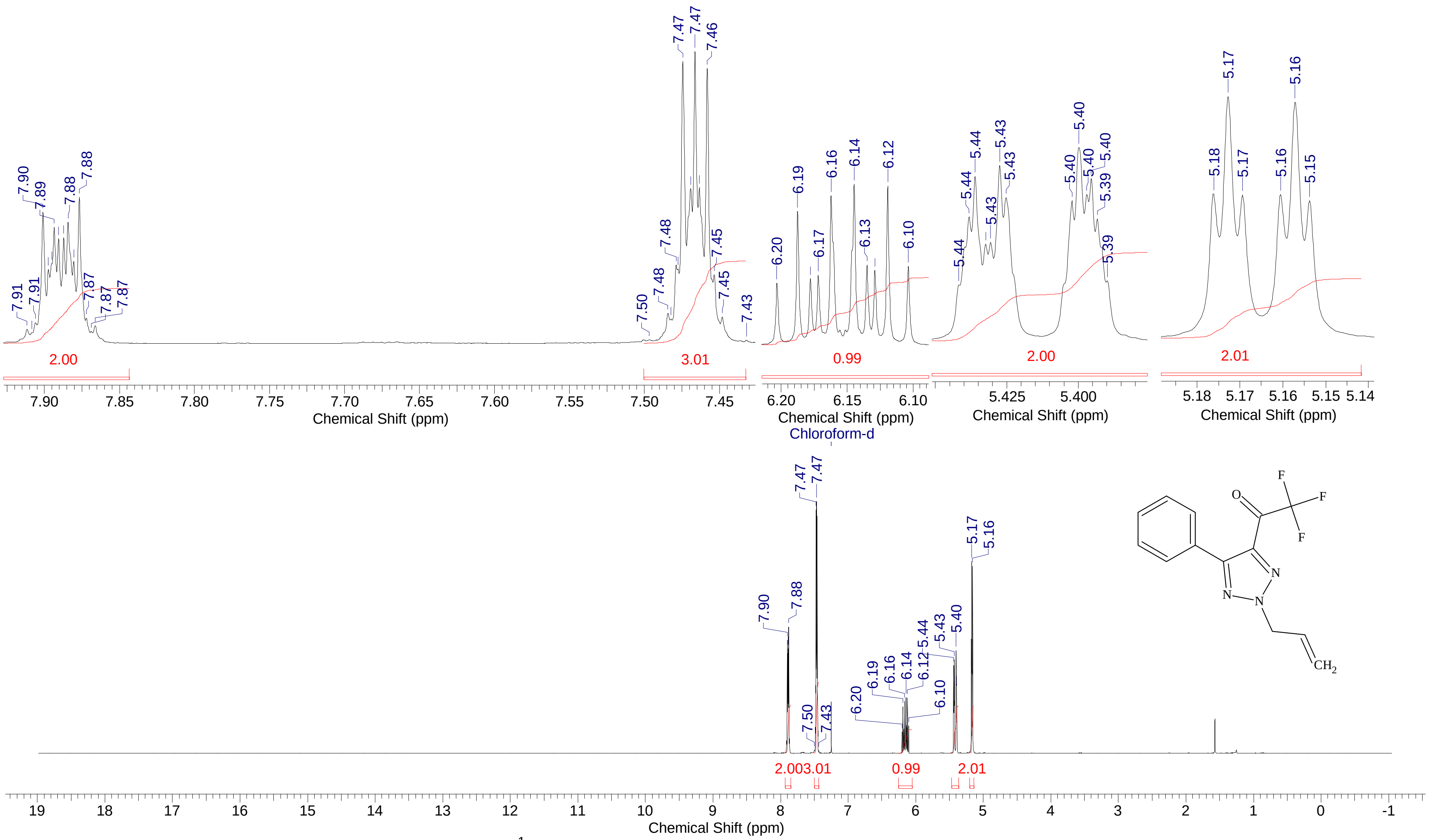

^{1}H NMR spectrum of **3d** (400.1 MHz, $CDCl_3$)

17 May 2022

Acquisition Time (sec)	1.7433	***Comment***	Imported from UXNMR.			***Date***	07 Apr 2022 12:29:52
File Name	C:\DOCS\OUTPUT_301\2022\04.апрель\BM-2477-1.F_005001r					***Frequency (MHz)***	376.50
Nucleus	19F	***Number of Transients***	16	***Original Points Count***	131072	***Points Count***	262144
Pulse Sequence	zgflqn	***Solvent***	CHLOROFORM-D			***Sweep Width (Hz)***	75187.97
Temperature (degree C)	27.000						

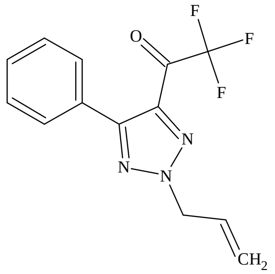

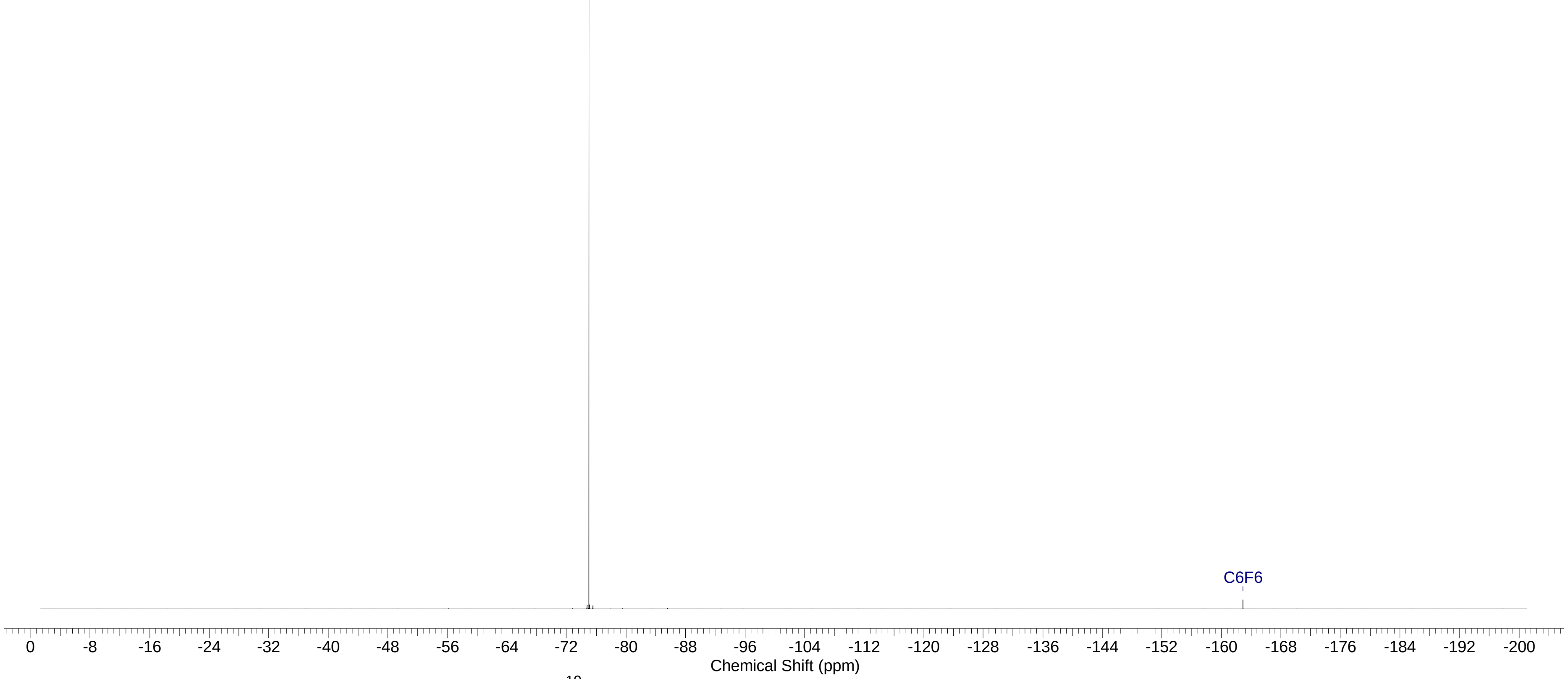

^{19}F NMR spectrum of **3d** (376.5 MHz, $CDCl_3$)

17 May 2022

Acquisition Time (sec)	0.6783	***Comment***	Imported from UXNMR.	***Date***	07 Apr 2022 12:06:12
File Name	C:\DOCS\OUTPUT_301\2022\04.апрель\BM-2477-1.C_002001r			***Frequency (MHz)***	100.61
Nucleus	13C	***Number of Transients***	257 ***Original Points Count*** 16384	***Points Count***	131072
Pulse Sequence	zgpg30	***Solvent***	CHLOROFORM-D	***Sweep Width (Hz)***	24154.59
Temperature (degree C)	27.000				

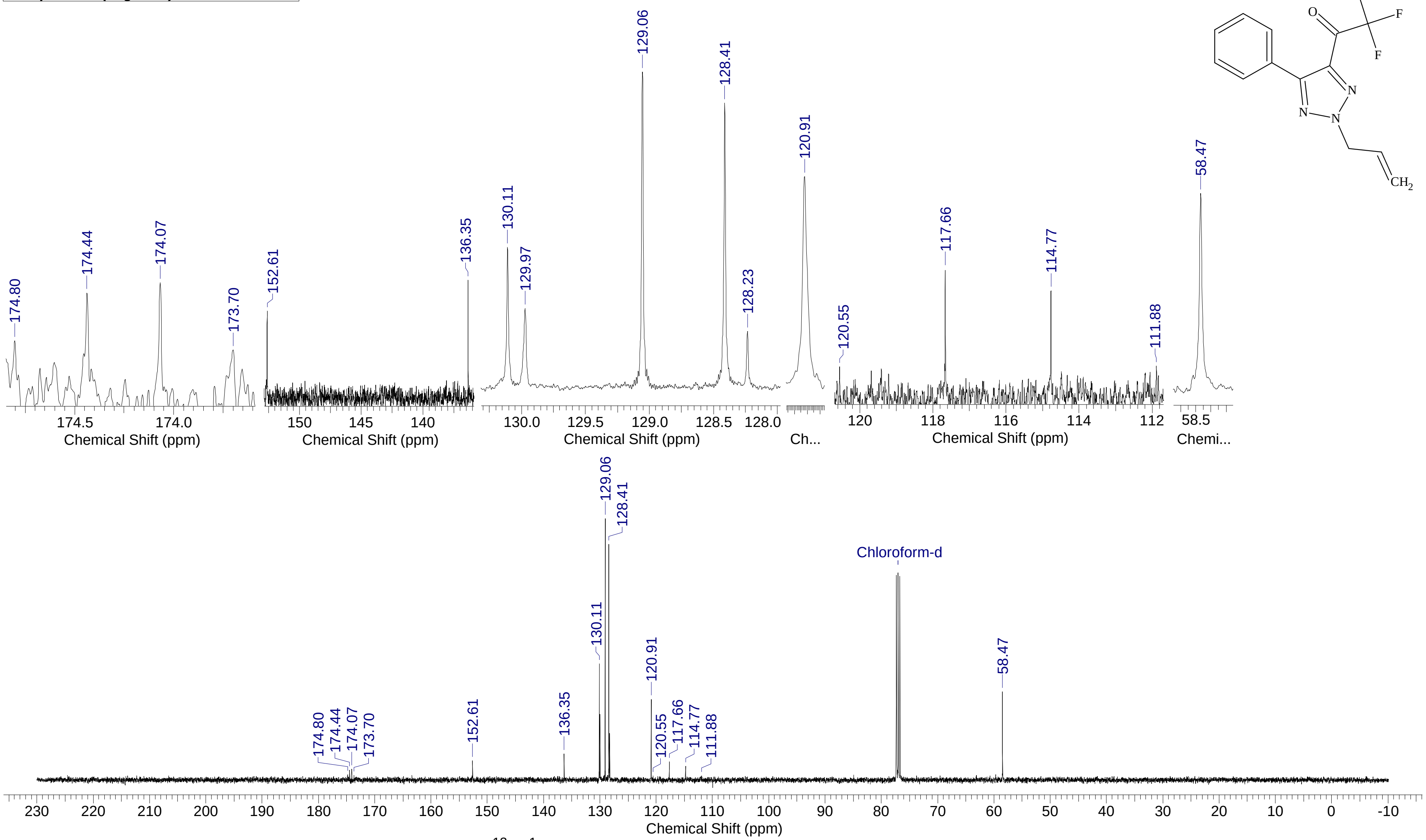

$^{13}C\{^{1}H\}$ NMR spectrum of **3d** (100.6 MHz, $CDCl_3$)

17 May 2022

Acquisition Time (sec)	4.0894	***Comment***	Imported from UXNMR.			***Date***	07 Apr 2022 11:33:34
File Name	C:\DOCS\OUTPUT_301\2022\04.апрель\BM-2477-2.H_001001r					***Frequency (MHz)***	400.13
Nucleus	1H	***Number of Transients***	4	***Original Points Count***	32768	***Points Count***	131072
Pulse Sequence	zg30	***Solvent***	CHLOROFORM-D			***Sweep Width (Hz)***	8012.82
Temperature (degree C)	27.000						

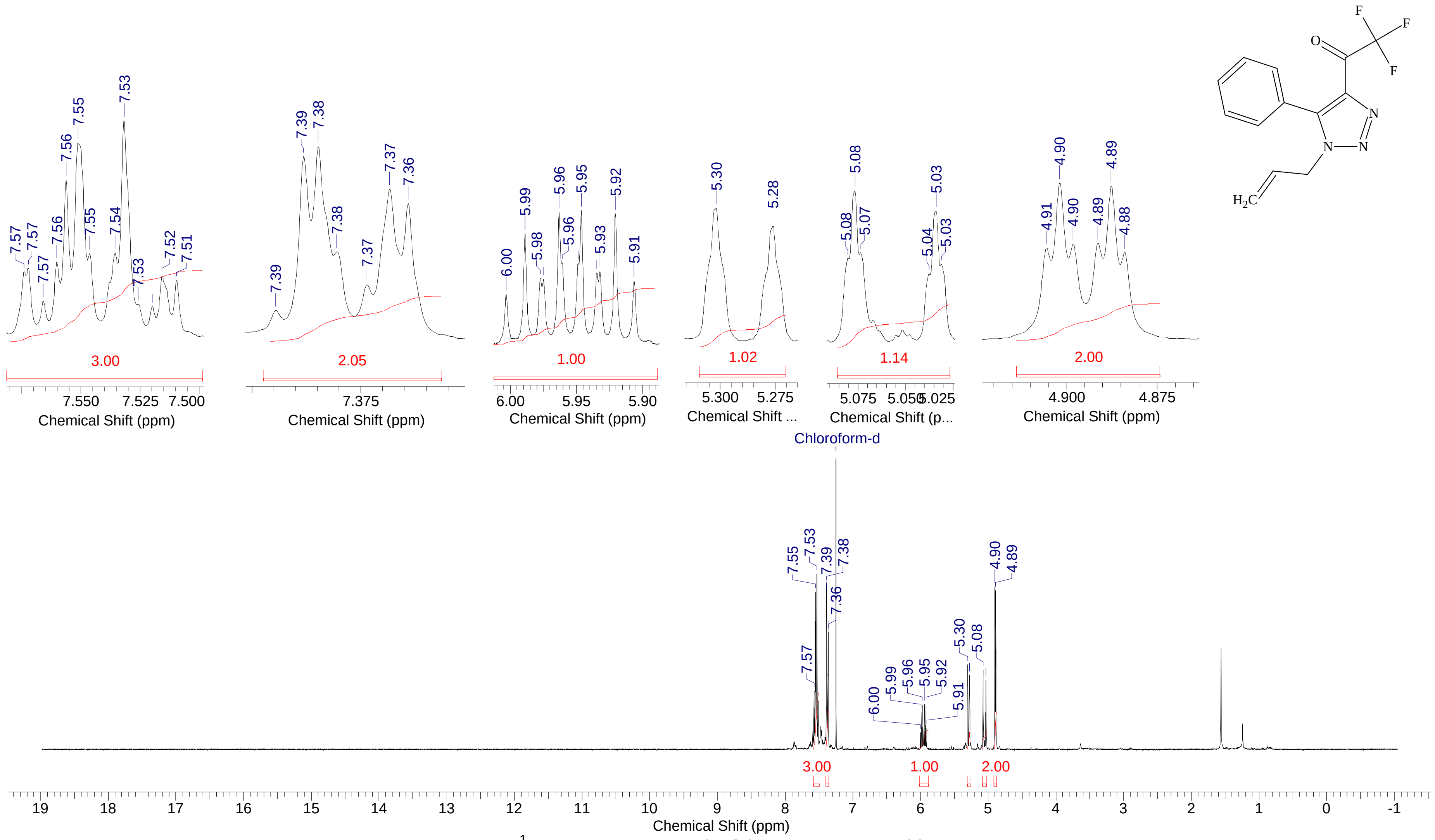

^{1}H NMR spectrum of **4d** (400.1 MHz, $CDCl_3$)

17 May 2022

Acquisition Time (sec)	1.7433	***Comment***	Imported from UXNMR.			***Date***	07 Apr 2022 12:20:12
File Name	C:\DOCS\OUTPUT_301\2022\04.апрель\BM-2477-2.F_005001r					***Frequency (MHz)***	376.50
Nucleus	19F	***Number of Transients***	16	***Original Points Count***	131072	***Points Count***	262144
Pulse Sequence	zgflqn	***Solvent***	CHLOROFORM-D			***Sweep Width (Hz)***	75187.97
Temperature (degree C)	27.000						

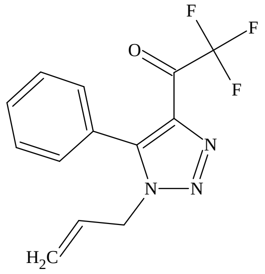

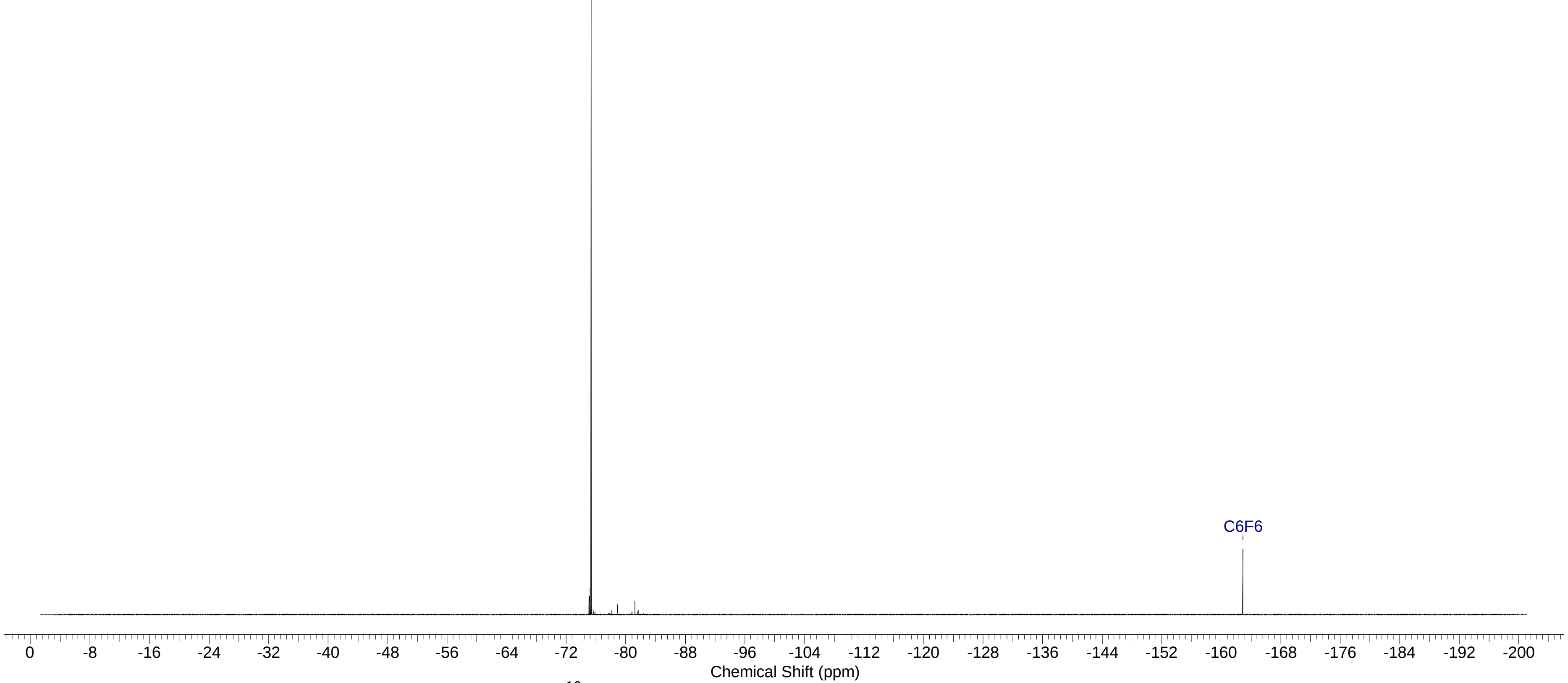

^{19}F NMR spectrum of **4d** (376.5 MHz, $CDCl_3$)

Acquisition Time (sec)	4.0894	***Comment***	Imported from UXNMR.	***Date***	18 Mar 2022 15:09:20
File Name	C:\DOCS\OUTPUT_301\2022\03.март\SZA-BM-2460-7.H_001001r			***Frequency (MHz)***	400.13
Nucleus	1H	***Number of Transients***	4	***Original Points Count*** 32768	
				Points Count	131072
Pulse Sequence	zg30	***Solvent***	CHLOROFORM-D	***Sweep Width (Hz)***	8012.82
Temperature (degree C)	27.000				

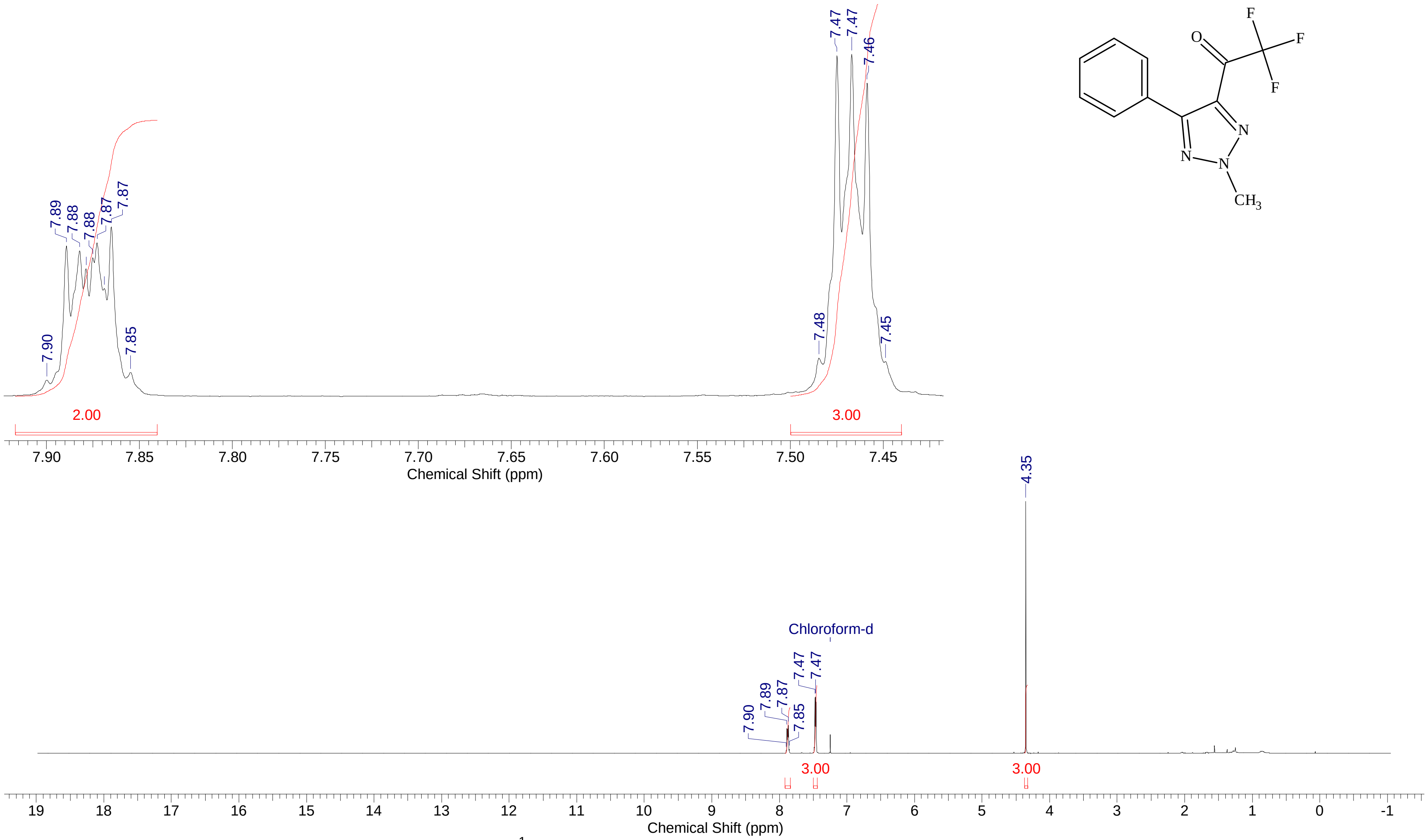

^{1}H NMR spectrum of **3e** (400.1 MHz, $CDCl_3$)

Acquisition Time (sec)	1.7433	Comment	Imported from UXNMR.			Date	18 Mar 2022 15:43:00
File Name	C:\DOCS\OUTPUT_301\2022\03.март\SZA-BM-2460-7.F_005001r					Frequency (MHz)	376.50
Nucleus	19F	Number of Transients	4	Original Points Count	131072	Points Count	262144
Pulse Sequence	zgflqn	Solvent	CHLOROFORM-D			Sweep Width (Hz)	75187.97
Temperature (degree C)	27.000						

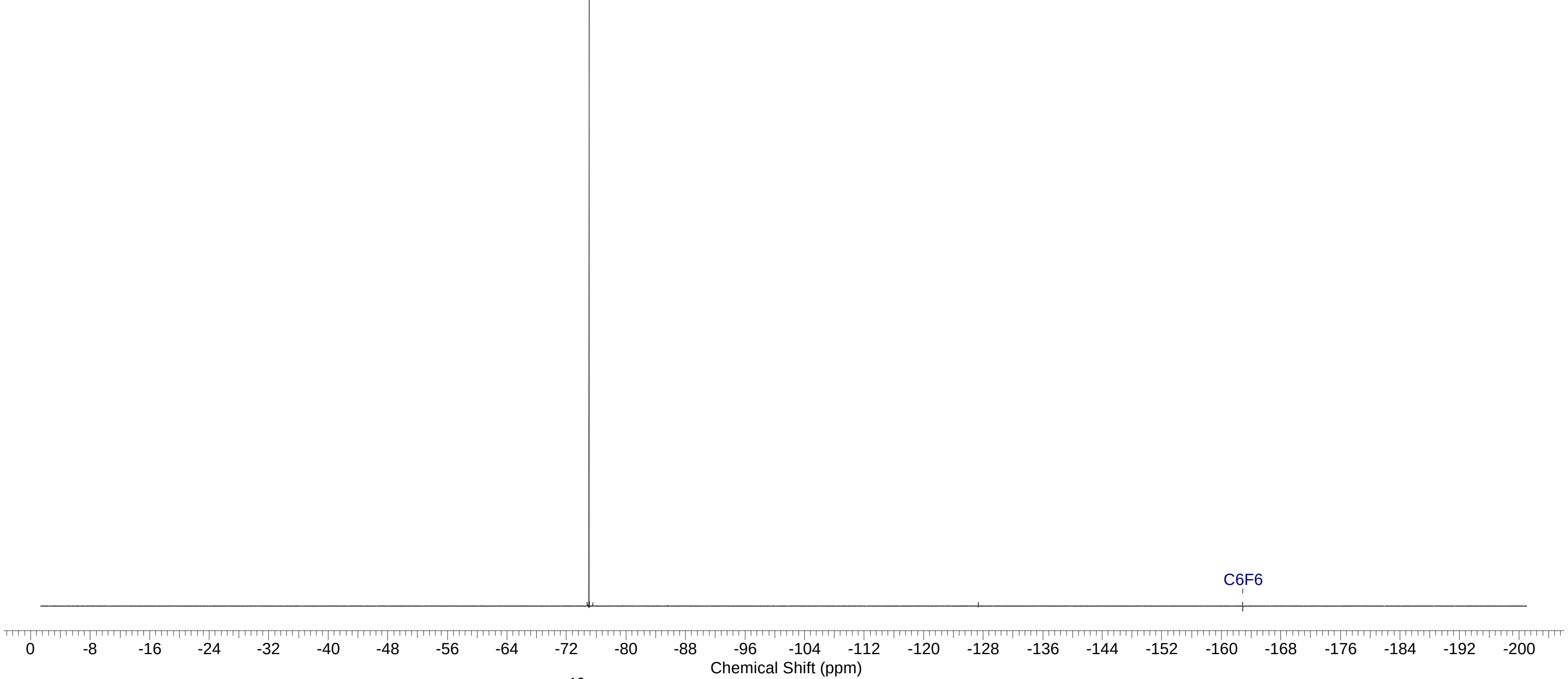

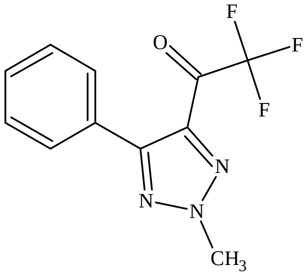

^{19}F NMR spectrum of **3e** (376.5 MHz, $CDCl_3$)

14 May 2022

Acquisition Time (sec)	0.6783	***Comment***	Imported from UXNMR.			***Date***	21 Mar 2022 15:59:54
File Name	C:\DOCS\OUTPUT_301\2022\03.март\SZA-BM-2460-7.C_002001r					***Frequency (MHz)***	100.61
Nucleus	13C	***Number of Transients***	161	***Original Points Count***	16384	***Points Count***	131072
Pulse Sequence	zgpg30	***Solvent***	DMSO-D6	***Sweep Width (Hz)***	24154.59	***Temperature (degree C)***	27.000

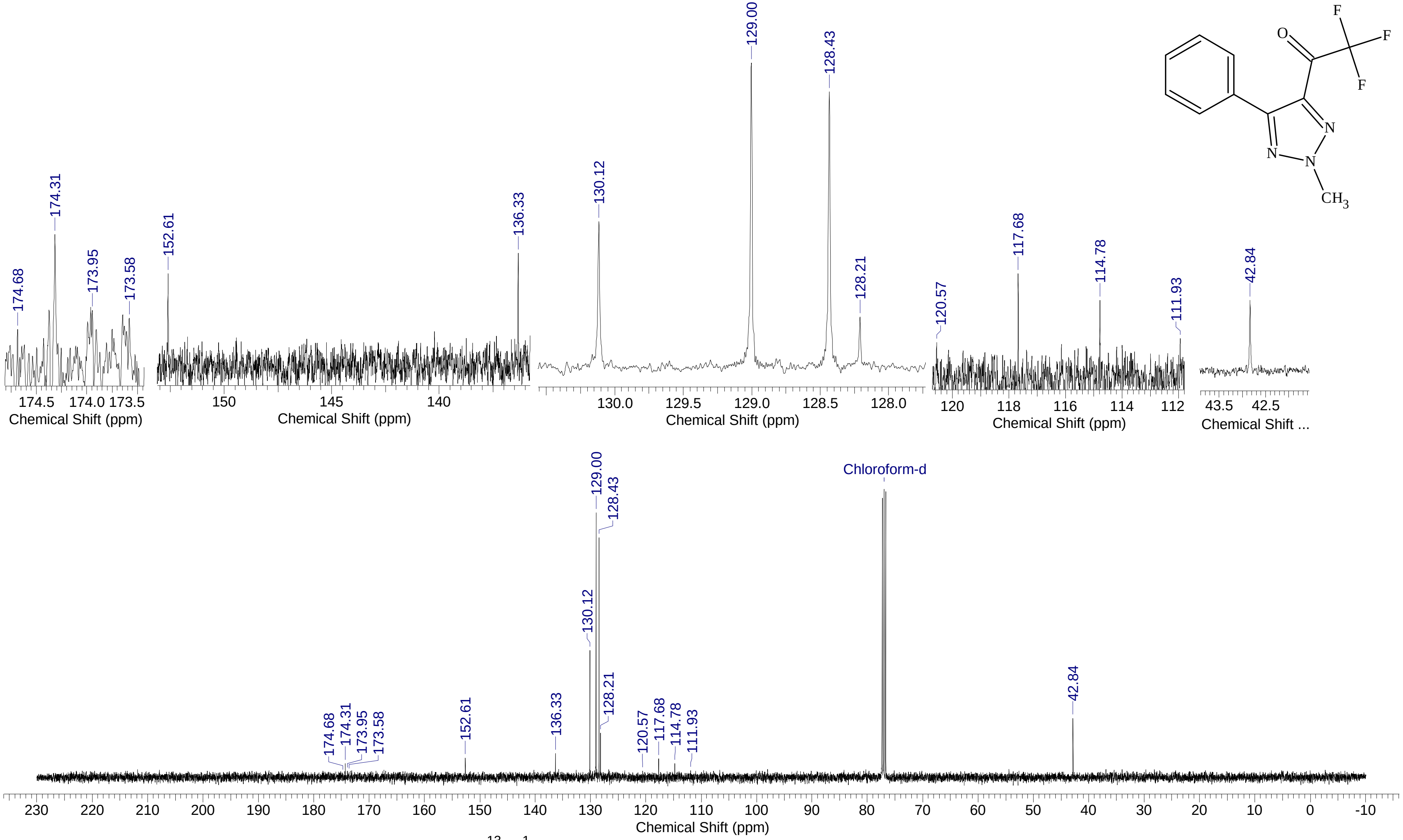

$^{13}C\{^{1}H\}$ NMR spectrum of **3e** (100.6 MHz, $CDCl_3$)

14 May 2022

Acquisition Time (sec)	4.0894	***Comment***	Imported from UXNMR.	***Date***	23 Mar 2022 00:03:58
File Name	C:\DOCS\BM\ЯМР_СОСЕДИ\2022\SZA-BM-2460-14\SZA-BM-2460-14_001001r			***Frequency (MHz)***	400.13
Nucleus	1H	***Number of Transients***	8 ***Original Points Count*** 32768	***Points Count***	131072
Pulse Sequence	zg30	***Solvent***	CHLOROFORM-D	***Sweep Width (Hz)***	8012.82
Temperature (degree C)	27.000				

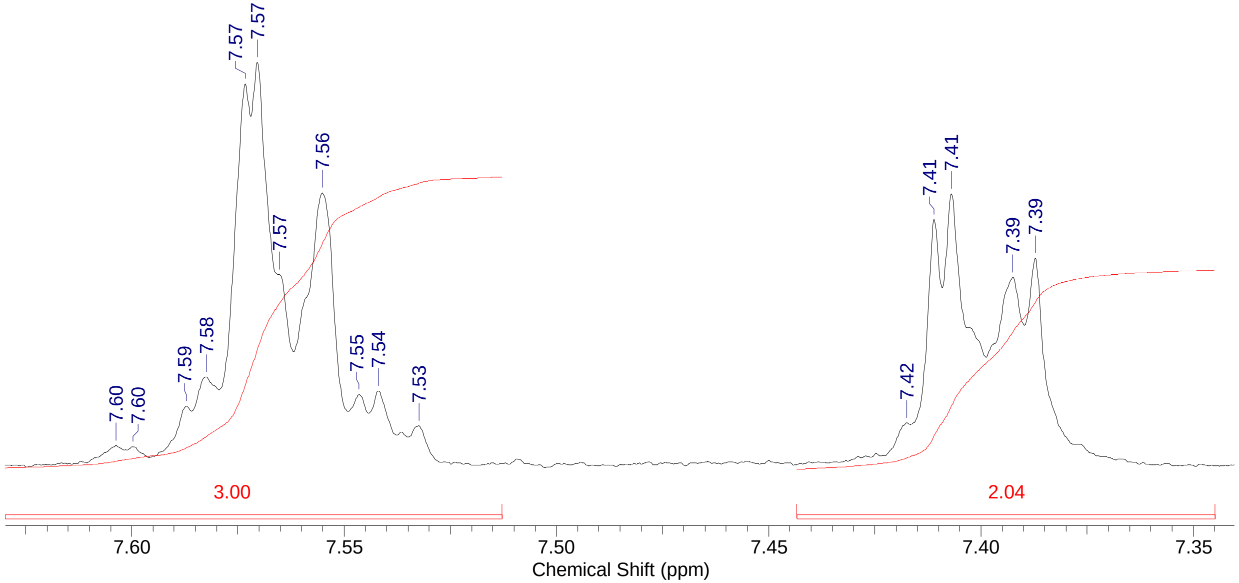

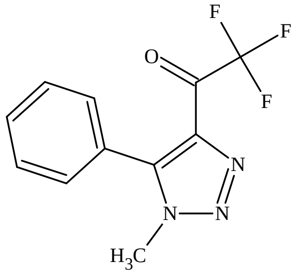

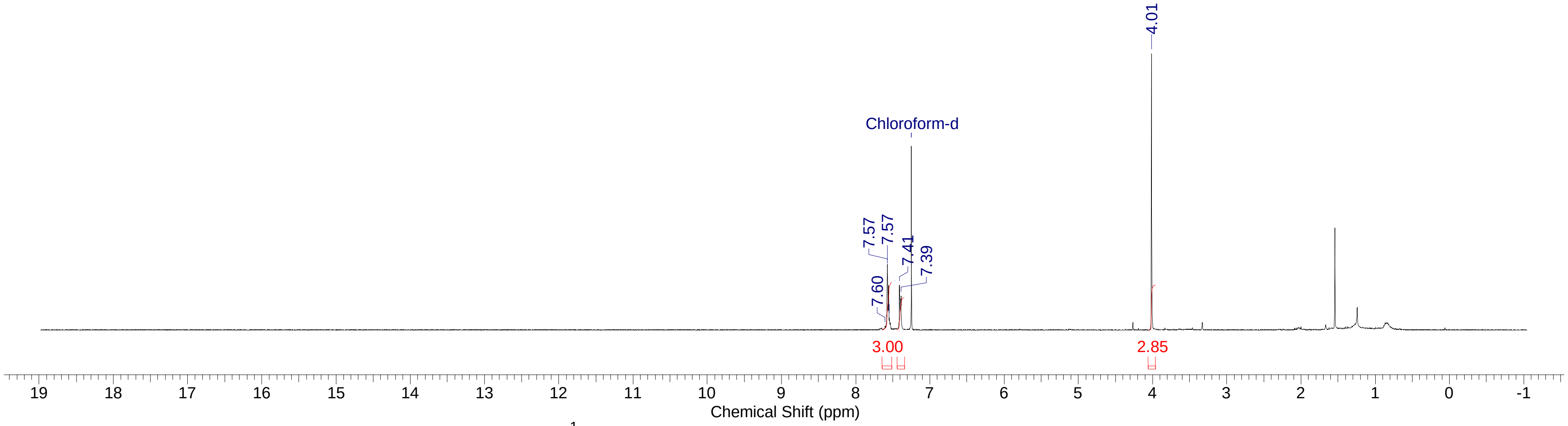

^{1}H NMR spectrum of **4e** (400.1 MHz, $CDCl_3$)

Acquisition Time (sec)	1.7433	***Comment***	Imported from UXNMR.			***Date***	22 Mar 2022 15:09:02
File Name	C:\DOCS\OUTPUT_301\2022\03.март\SZA-BM-2460-14.F_005001r					***Frequency (MHz)***	376.50
Nucleus	19F	***Number of Transients***	4	***Original Points Count***	131072	***Points Count***	262144
Pulse Sequence	zgflqn	***Solvent***	CHLOROFORM-D			***Sweep Width (Hz)***	75187.97
Temperature (degree C)	27.000						

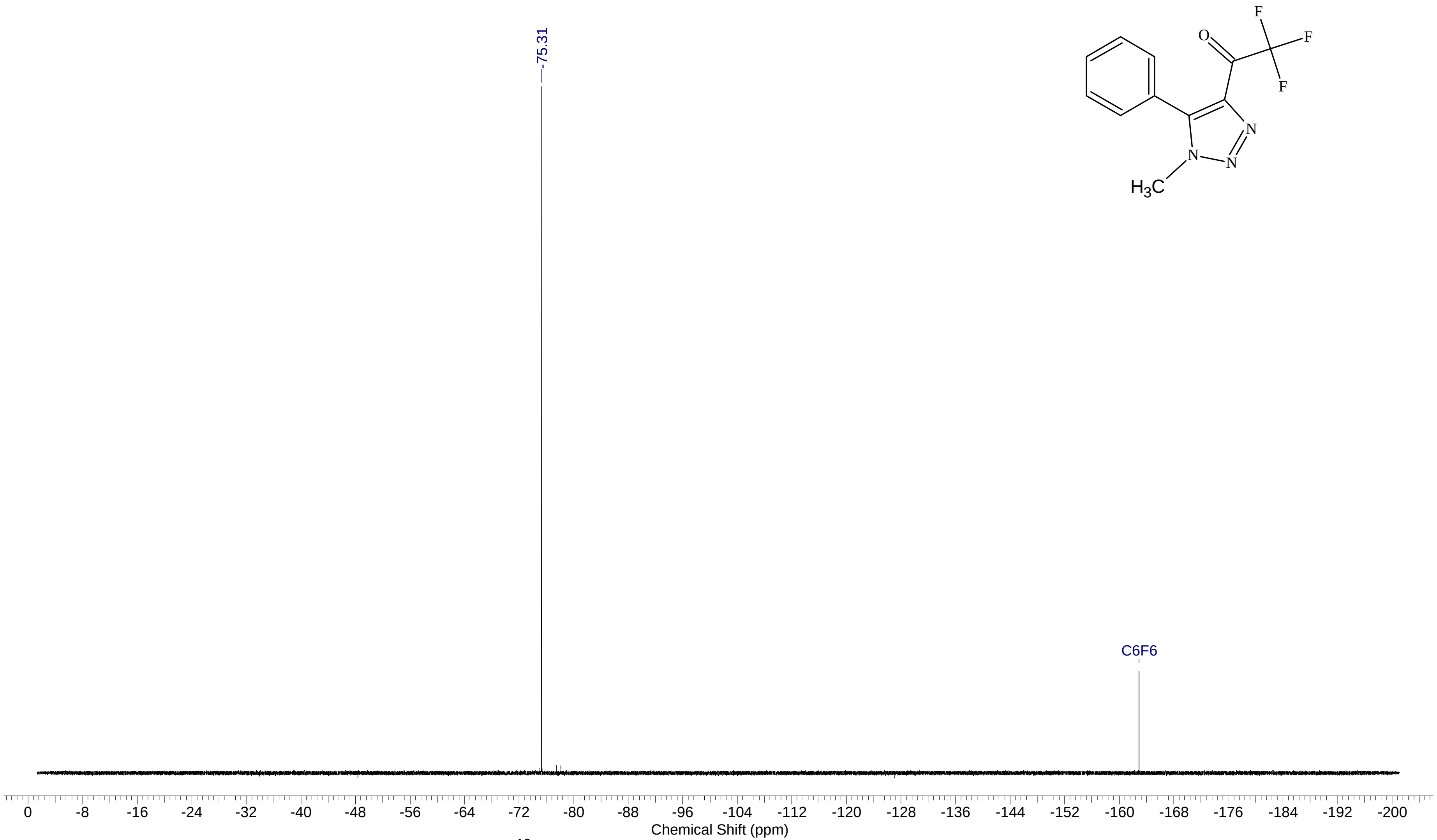

^{19}F NMR spectrum of **4e** (376.5 MHz, $CDCl_3$)

14 May 2022

Acquisition Time (sec)	0.6783	***Comment***	Imported from UXNMR.			***Date***	25 Mar 2022 15:18:18
File Name	C:\DOCS\OUTPUT_301\2022\03.март\SZA-BM-2460-14.C_002001r					***Frequency (MHz)***	100.61
Nucleus	13C	***Number of Transients***	2169	***Original Points Count***	16384	***Points Count***	131072
Pulse Sequence	zgpg30	***Solvent***	CHLOROFORM-D			***Sweep Width (Hz)***	24154.59
Temperature (degree C)	27.000						

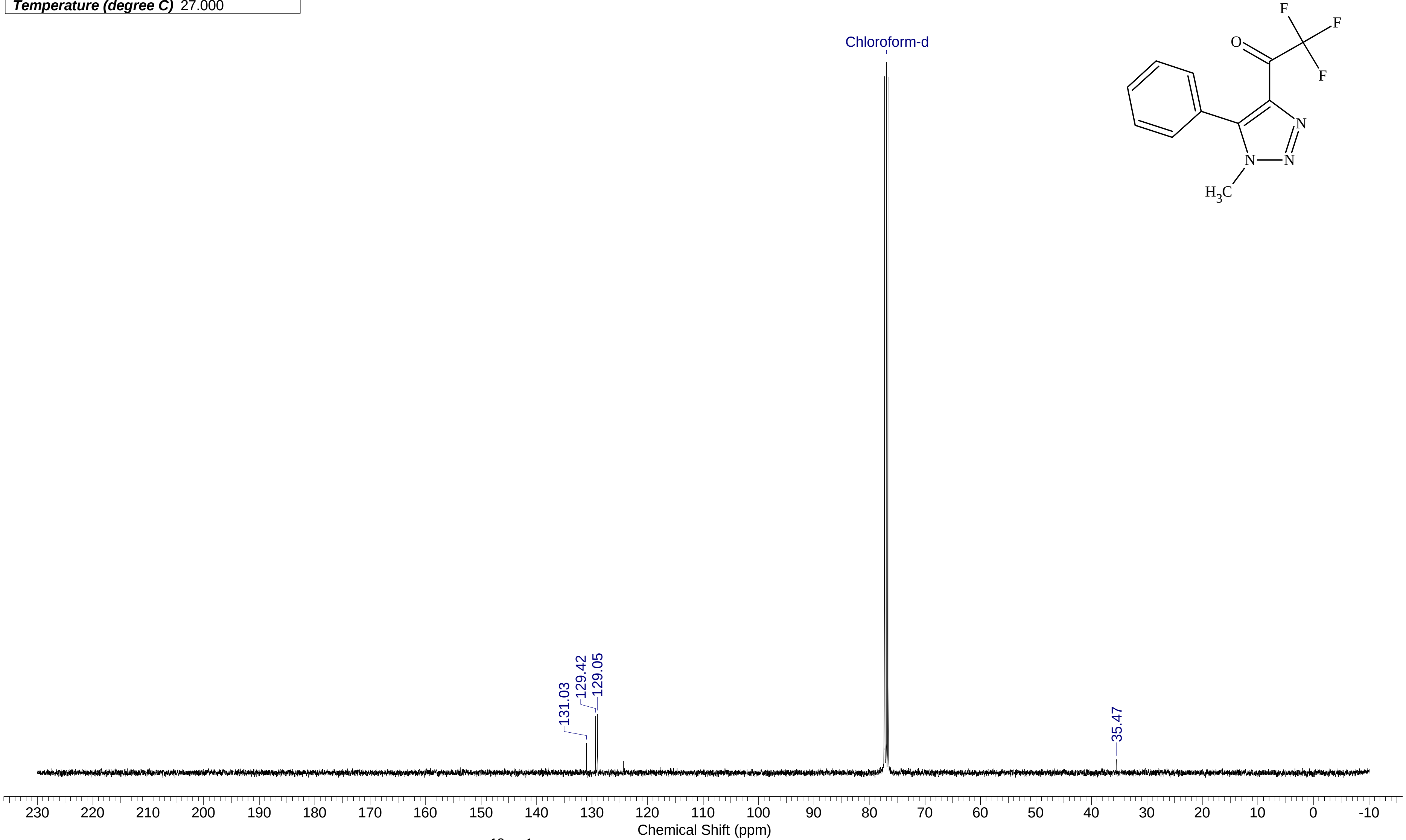

$^{13}C\{^{1}H\}$ NMR spectrum of **4e** (100.6 MHz, $CDCl_3$)

14 May 2022

Acquisition Time (sec)	4.0894	Comment	Imported from UXNMR.			Date	05 Apr 2022 11:46:08
File Name	C:\DOCS\OUTPUT_301\2022\04.апрель\BM-2478-1.H\BM-2478-1.H_001000fid					Frequency (MHz)	400.13
Nucleus	1H	Number of Transients	4	Original Points Count	32768	Points Count	32768
Pulse Sequence	zg30	Solvent	BENZENE-D6	Sweep Width (Hz)	8012.82	Temperature (degree C)	27.000

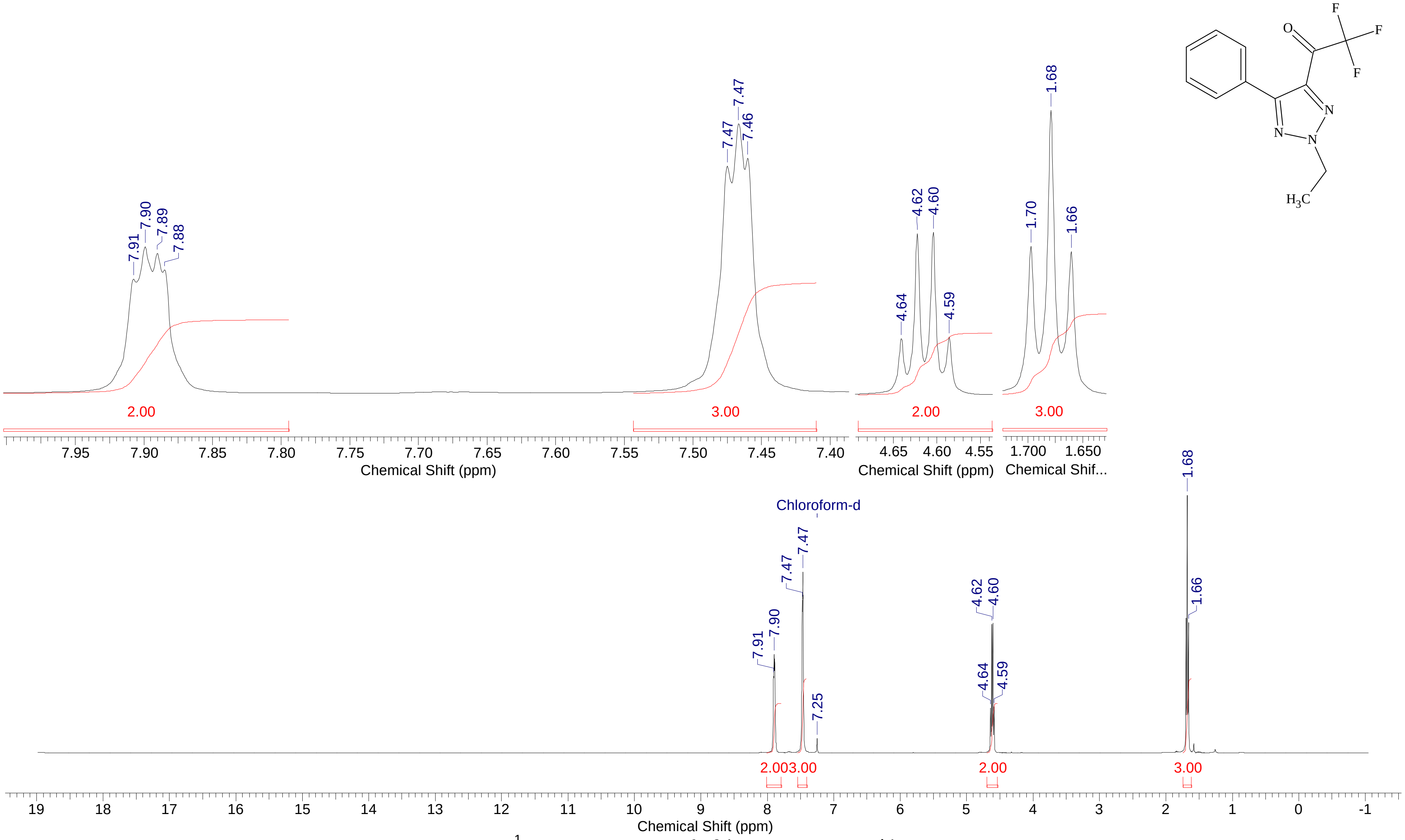

^{1}H NMR spectrum of **3f** (400.1 MHz, $CDCl_3$)

14 May 2022

Acquisition Time (sec)	1.7433	***Comment***	Imported from UXNMR.			***Date***	05 Apr 2022 15:25:08
File Name	C:\DOCS\OUTPUT_301\2022\04.апрель\BM-2478-1.F.F_005001r					***Frequency (MHz)***	376.50
Nucleus	19F	***Number of Transients***	4	***Original Points Count***	131072	***Points Count***	262144
Pulse Sequence	zgflqn	***Solvent***	CHLOROFORM-D			***Sweep Width (Hz)***	75187.97
Temperature (degree C)	27.000						

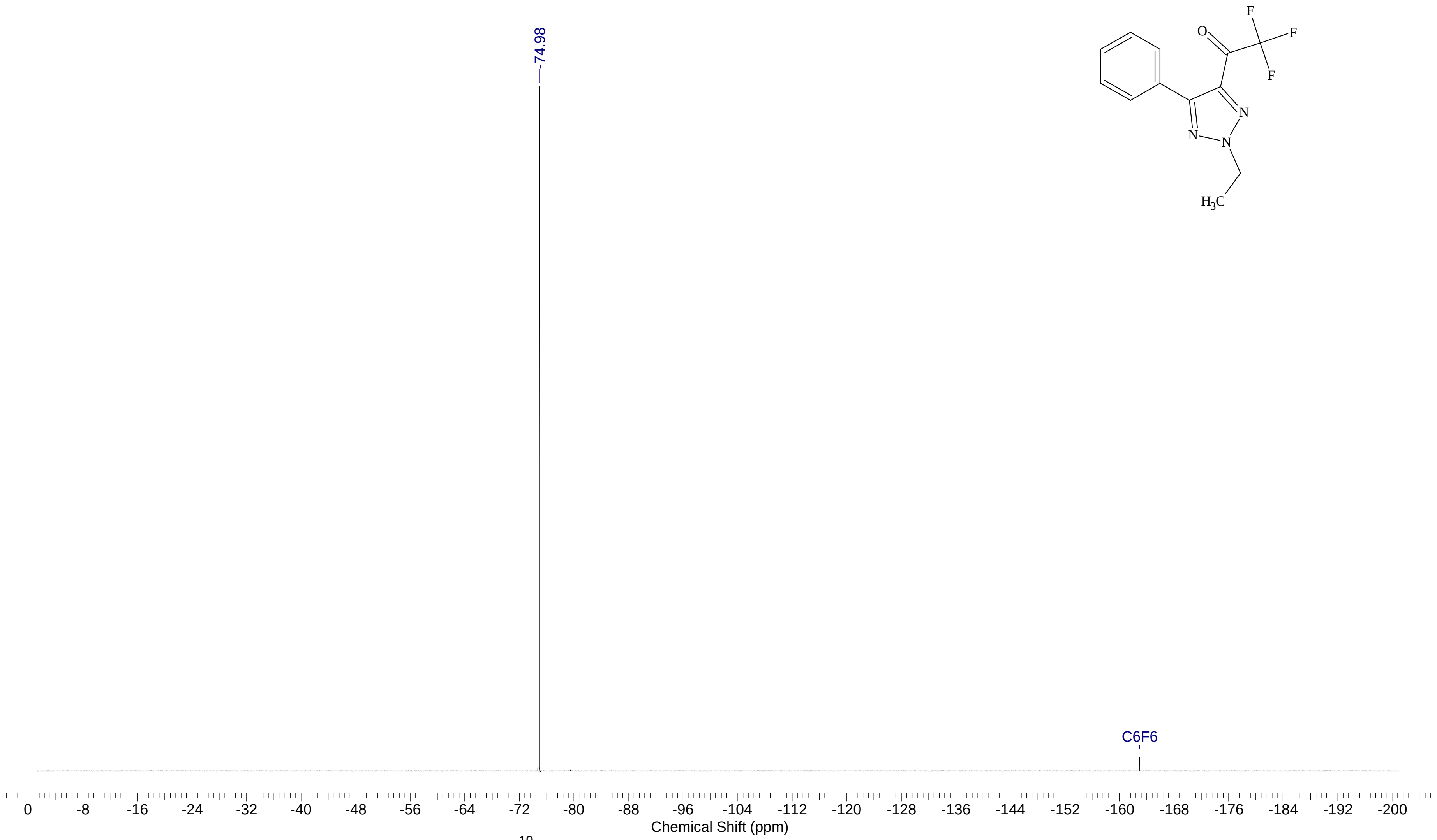

^{19}F NMR spectrum of **3f** (376.5 MHz, $CDCl_3$)

14 May 2022

Acquisition Time (sec)	0.6783	***Comment***	Imported from UXNMR.			***Date***	05 Apr 2022 14:57:30
File Name	C:\DOCS\OUTPUT_301\2022\04.апрель\BM-2478-1.C_002001r					***Frequency (MHz)***	100.61
Nucleus	13C	***Number of Transients***	281	***Original Points Count***	16384	***Points Count***	131072
Pulse Sequence	zgpg30	***Solvent***	CHLOROFORM-D			***Sweep Width (Hz)***	24154.59
Temperature (degree C)	27.000						

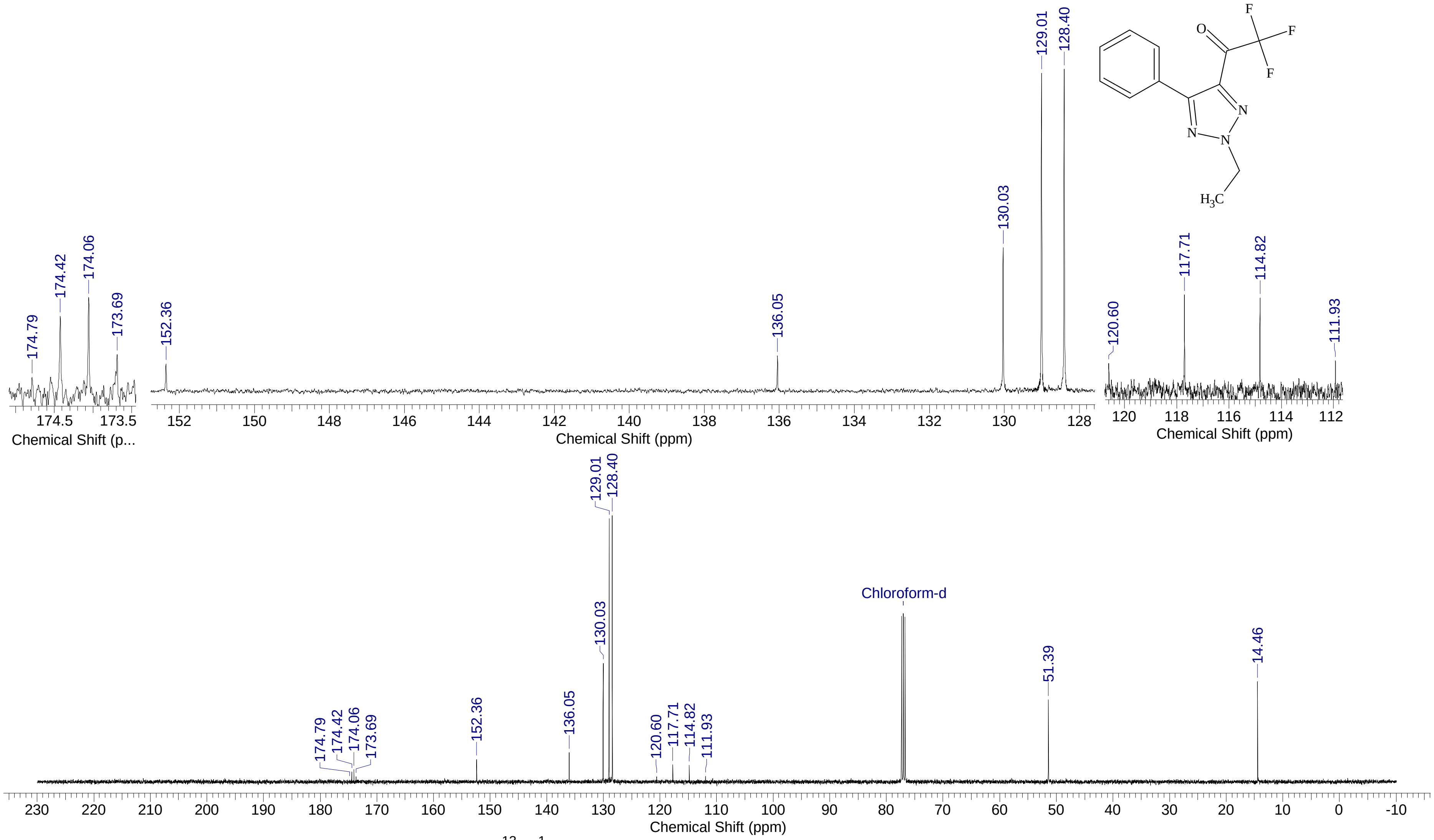

$^{13}C\{^{1}H\}$ NMR spectrum of **3f** (100.6 MHz, $CDCl_3$)

14 May 2022

Acquisition Time (sec)	4.0894	***Comment***	Imported from UXNMR.			***Date***	05 Apr 2022 15:03:36
File Name	C:\DOCS\OUTPUT_301\2022\04.апрель\BM-2478-2.H_001001r					***Frequency (MHz)***	400.13
Nucleus	1H	***Number of Transients***	4	***Original Points Count***	32768	***Points Count***	131072
Pulse Sequence	zg30	***Solvent***	BENZENE-D6	***Sweep Width (Hz)***	8012.82	***Temperature (degree C)***	27.000

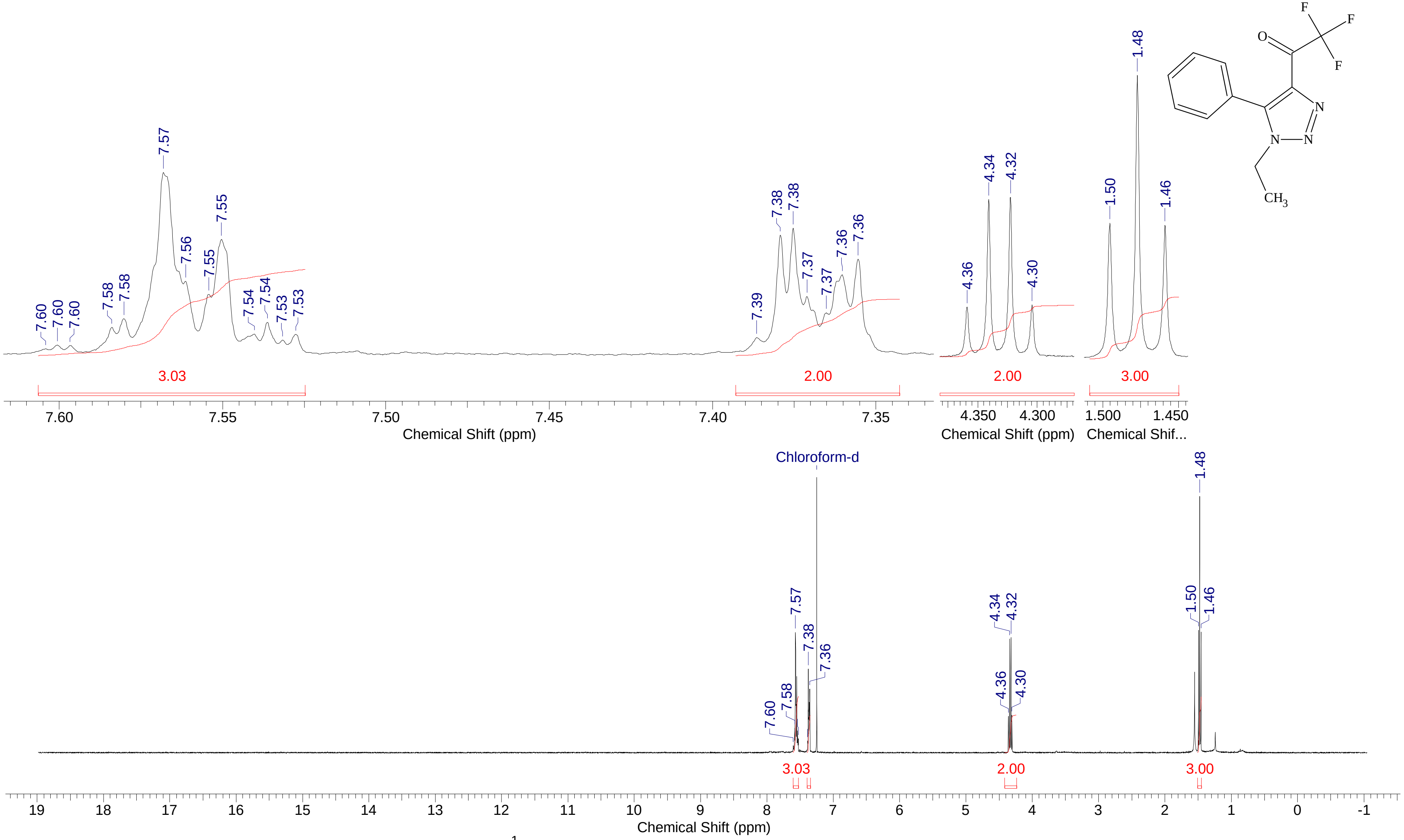

^{1}H NMR spectrum of **4f** (400.1 MHz, $CDCl_3$)

14 May 2022

Acquisition Time (sec)	1.7433	***Comment***	Imported from UXNMR.			***Date***	05 Apr 2022 15:28:58
File Name	C:\DOCS\OUTPUT_301\2022\04.апрель\BM-2478-2.F_005001r					***Frequency (MHz)***	376.50
Nucleus	19F	***Number of Transients***	4	***Original Points Count***	131072	***Points Count***	262144
Pulse Sequence	zgflqn	***Solvent***	CHLOROFORM-D			***Sweep Width (Hz)***	75187.97
Temperature (degree C)	27.000						

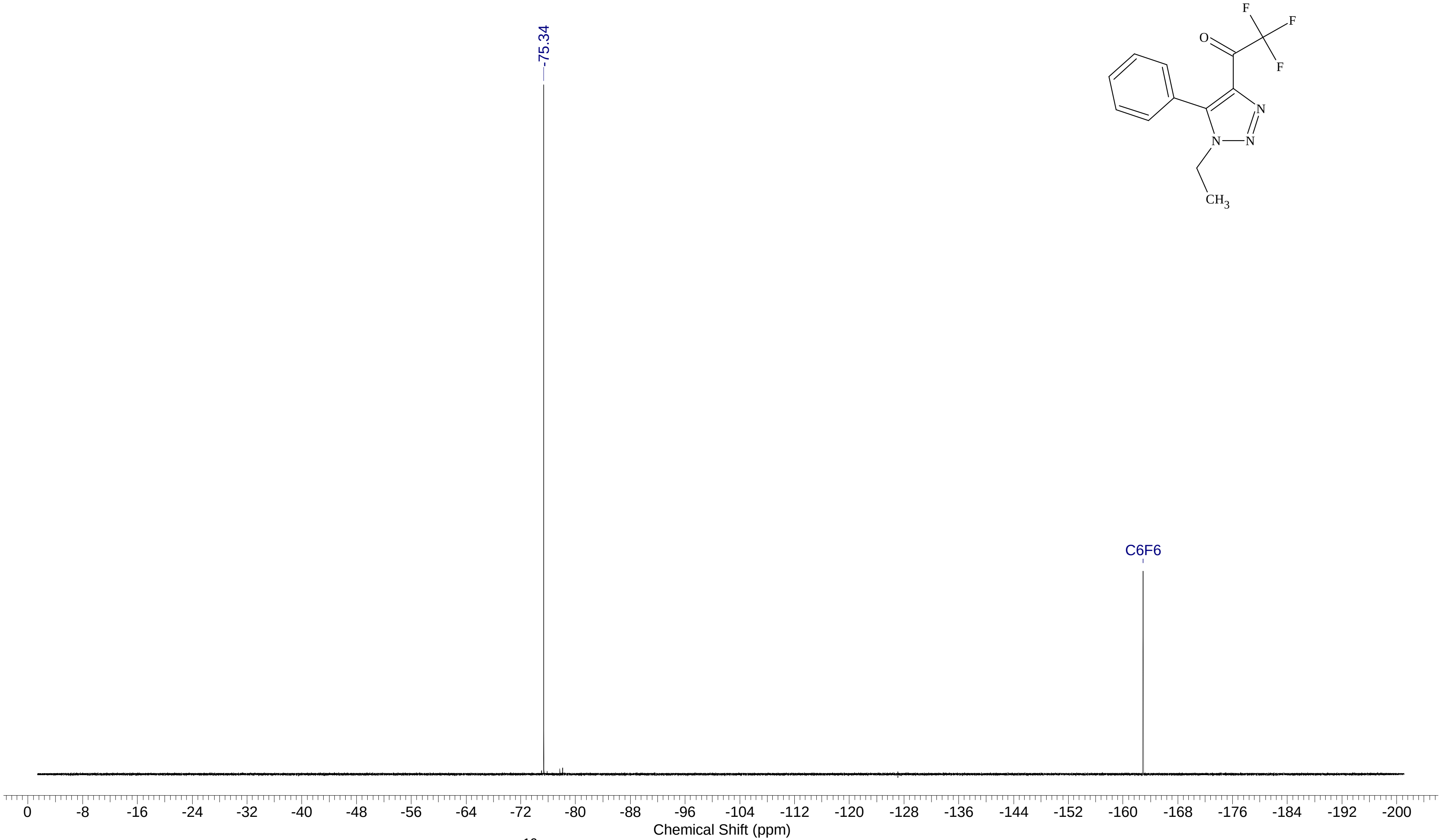

^{19}F NMR spectrum of **4f** (376.5 MHz, $CDCl_3$)

14 May 2022

Acquisition Time (sec)	4.0894	***Comment***	Imported from UXNMR.			***Date***	23 Mar 2022 17:38:50
File Name	C:\DOCS\OUTPUT_301\2022\03.март\SZA-BM-2458-4.H_001001r					***Frequency (MHz)***	400.13
Nucleus	1H	***Number of Transients***	4	***Original Points Count***	32768	***Points Count***	131072
Pulse Sequence	zg30	***Solvent***	BENZENE-D6	***Sweep Width (Hz)***	8012.82	***Temperature (degree C)***	27.000

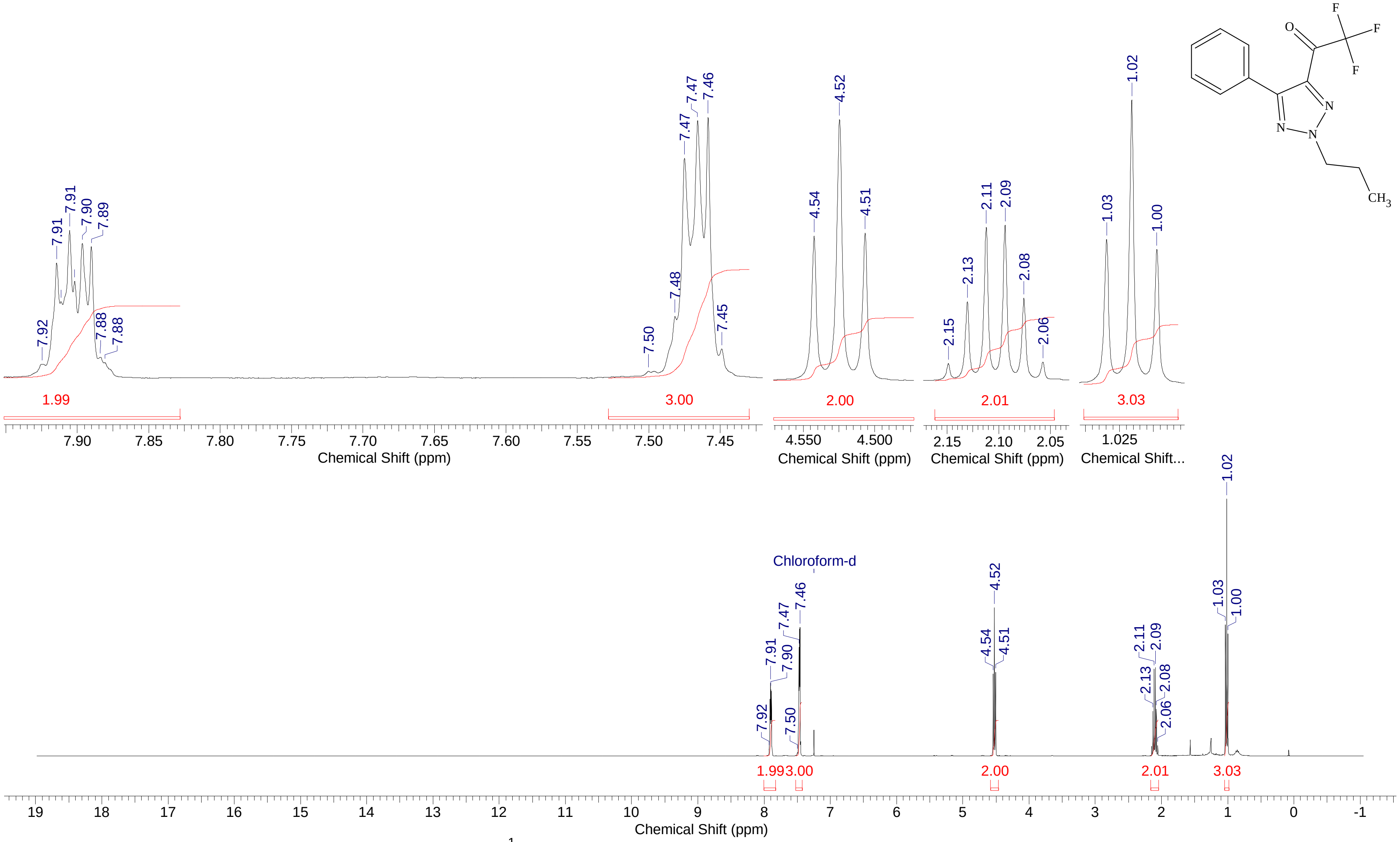

^{1}H NMR spectrum of **3g** (400.1 MHz, $CDCl_3$)

14 May 2022

Acquisition Time (sec)	1.7433	Comment	Imported from UXNMR.			Date	23 Mar 2022 17:43:32
File Name	C:\DOCS\OUTPUT_301\2022\03.март\SZA-BM-2458-4.F_005001r					Frequency (MHz)	376.50
Nucleus	19F	Number of Transients	16	Original Points Count	131072	Points Count	262144
Pulse Sequence	zgflqn	Solvent	CHLOROFORM-D			Sweep Width (Hz)	75187.97
Temperature (degree C)	27.000						

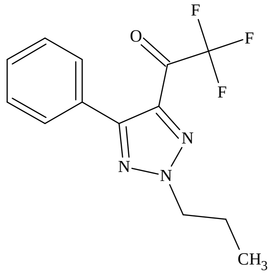

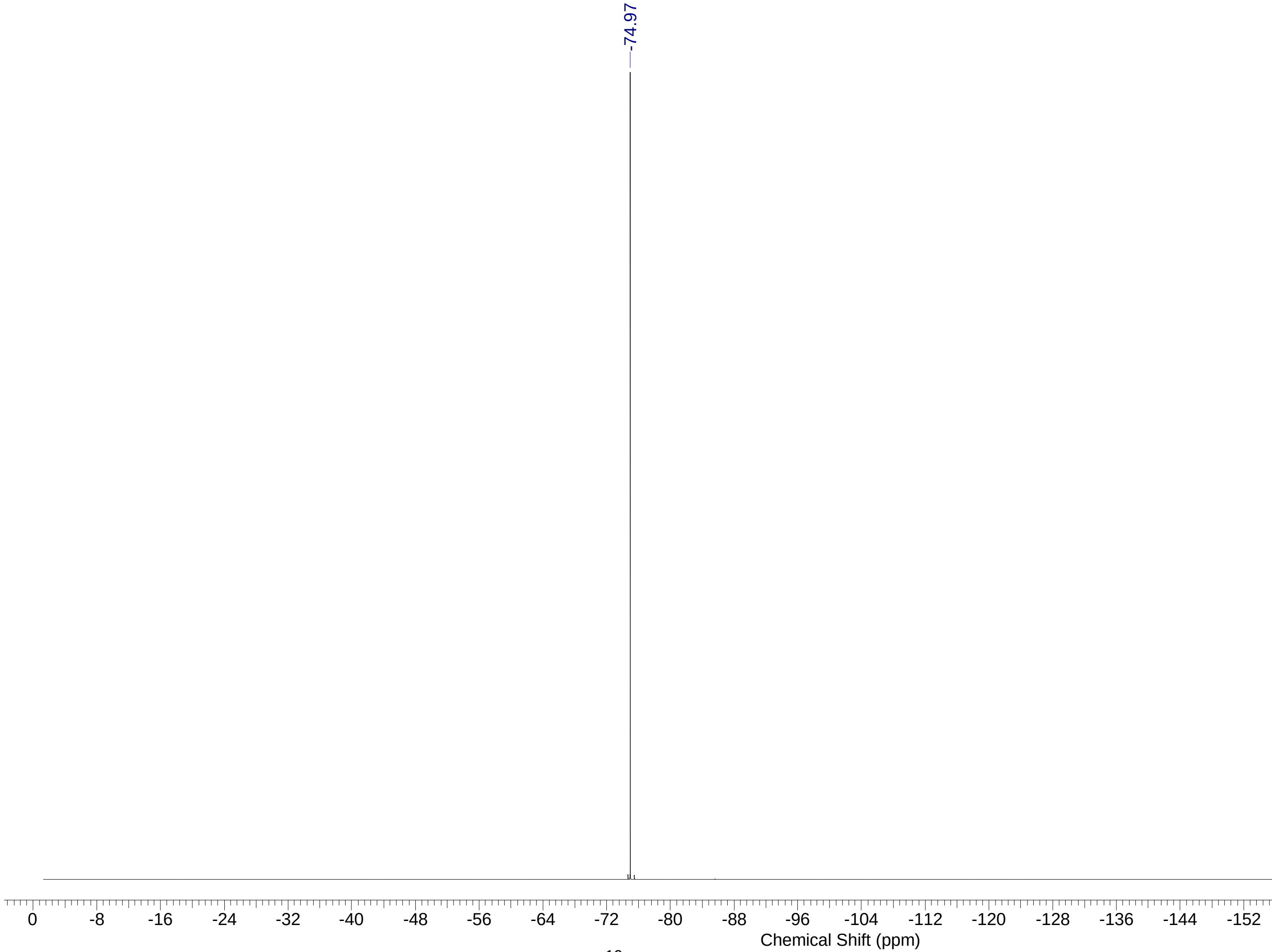

^{19}F NMR spectrum of **3g** (376.5 MHz, $CDCl_3$)

14 May 2022

**Acquisition Time (sec)**	0.6783	**Comment**	Imported from UXNMR.			**Date**	24 Mar 2022 12:35:36
**File Name**	C:\DOCS\OUTPUT_301\2022\03.март\SZA-BM-2458-4.C_002001r					**Frequency (MHz)**	100.61
**Nucleus**	13C	**Number of Transients**	121	**Original Points Count**	16384	**Points Count**	131072
**Pulse Sequence**	zgpg30	**Solvent**	DMSO-D6	**Sweep Width (Hz)**	24154.59	**Temperature (degree C)**	27.000

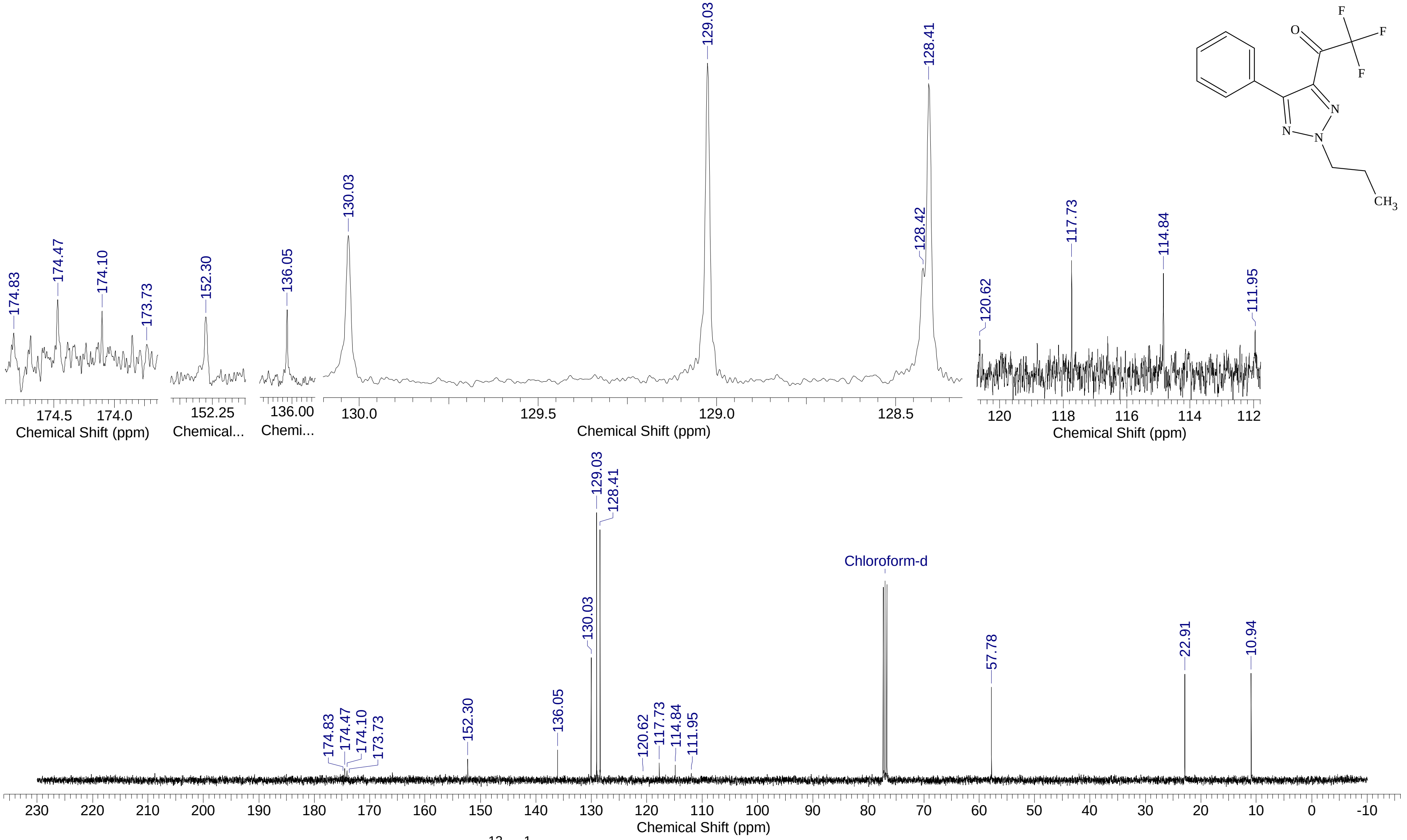

$^{13}C\{^1H\}$ NMR spectrum of **3g** (100.6 MHz, $CDCl_3$)

14 May 2022

Acquisition Time (sec)	4.0894	Comment	Imported from UXNMR.			Date	21 Mar 2022 15:26:36
File Name	C:\DOCS\OUTPUT_301\2022\03.март\SZA-BM-2458.H_001001r					Frequency (MHz)	400.13
Nucleus	1H	Number of Transients	4	Original Points Count	32768	Points Count	131072
Pulse Sequence	zg30	Solvent	CHLOROFORM-D			Sweep Width (Hz)	8012.82
Temperature (degree C)	27.000						

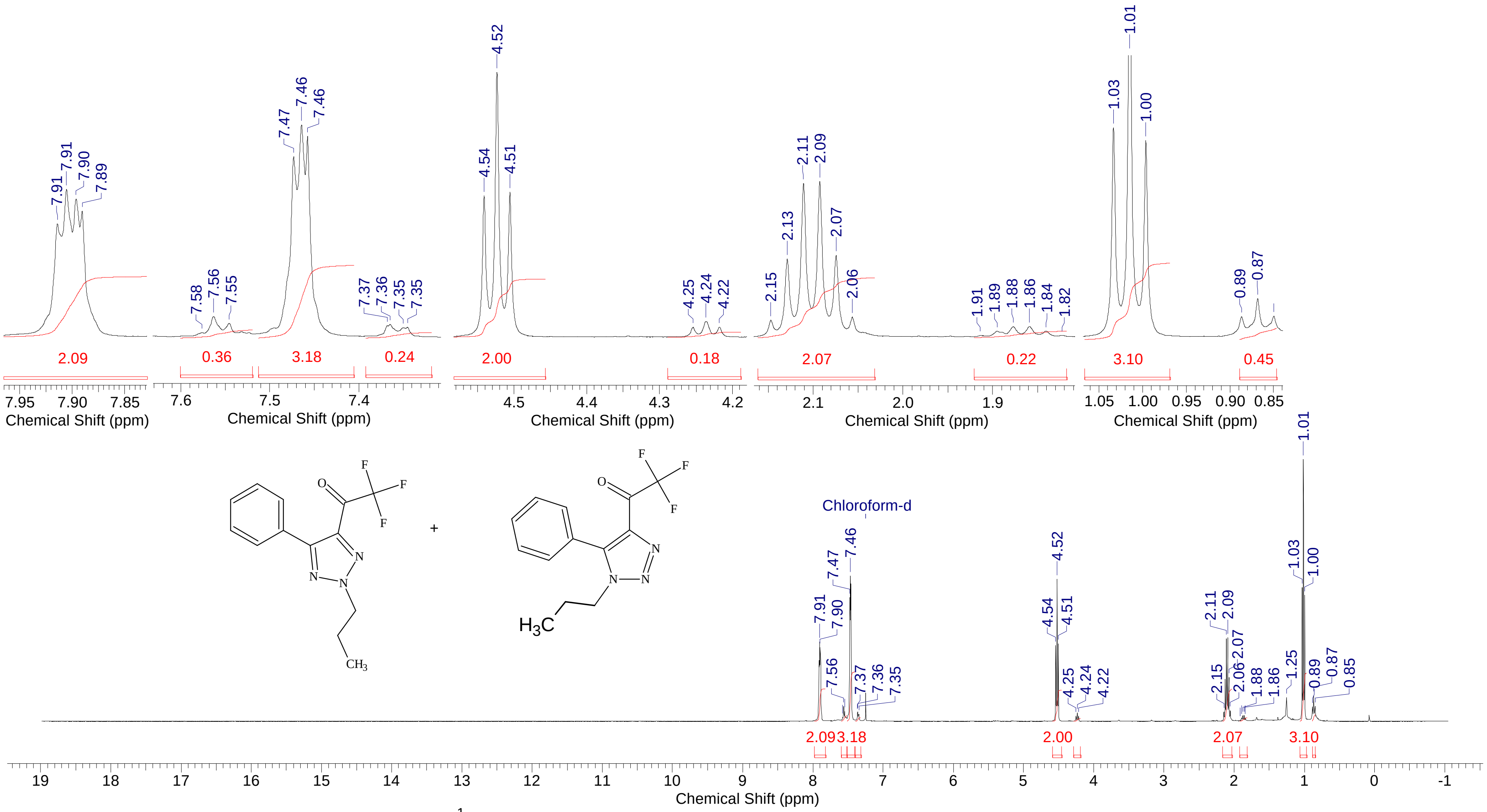

^{1}H NMR spectrum of mixture of **3g** and **4g** (400.1 MHz, $CDCl_3$)

14 May 2022

Acquisition Time (sec)	1.7433	***Comment***	Imported from UXNMR.			***Date***	21 Mar 2022 15:38:44
File Name	C:\DOCS\OUTPUT_301\2022\03.март\SZA-BM-2458.F_005001r					***Frequency (MHz)***	376.50
Nucleus	19F	***Number of Transients***	16	***Original Points Count***	131072	***Points Count***	262144
Pulse Sequence	zgflqn	***Solvent***	CHLOROFORM-D			***Sweep Width (Hz)***	75187.97
Temperature (degree C)	27.000						

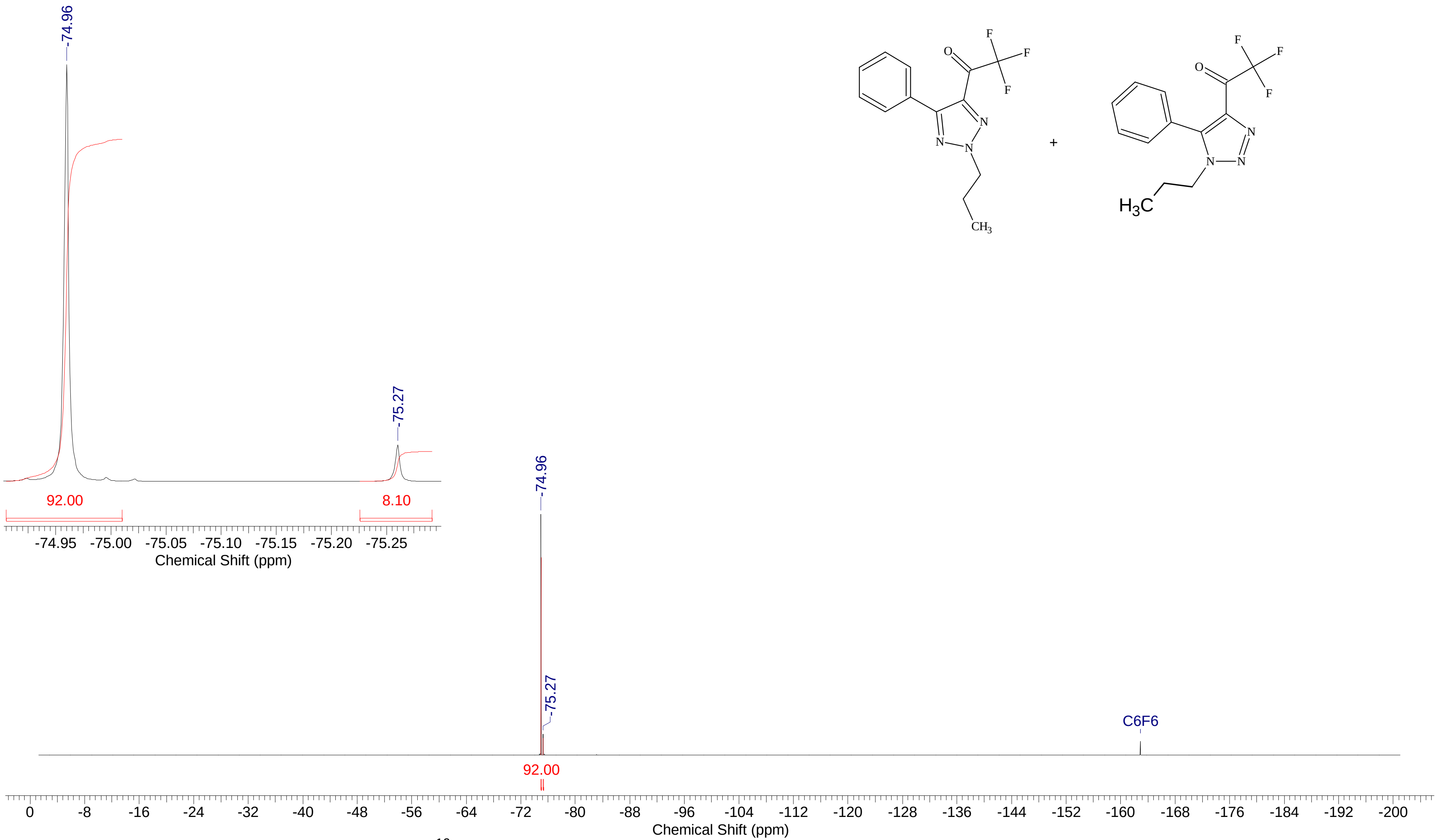

^{19}F NMR spectrum of mixture of **3g** and **4g** (376.5 MHz, $CDCl_3$)

14 May 2022

Acquisition Time (sec)	0.6783	***Comment***	Imported from UXNMR.			***Date***	22 Mar 2022 14:03:50
File Name	C:\DOCS\OUTPUT_301\2022\03.март\SZA-BM-2458.C_002001r					***Frequency (MHz)***	100.61
Nucleus	13C	***Number of Transients***	2145	***Original Points Count***	16384	***Points Count***	131072
Pulse Sequence	zgpg30	***Solvent***	DMSO-D6	***Sweep Width (Hz)***	24154.59	***Temperature (degree C)***	27.000

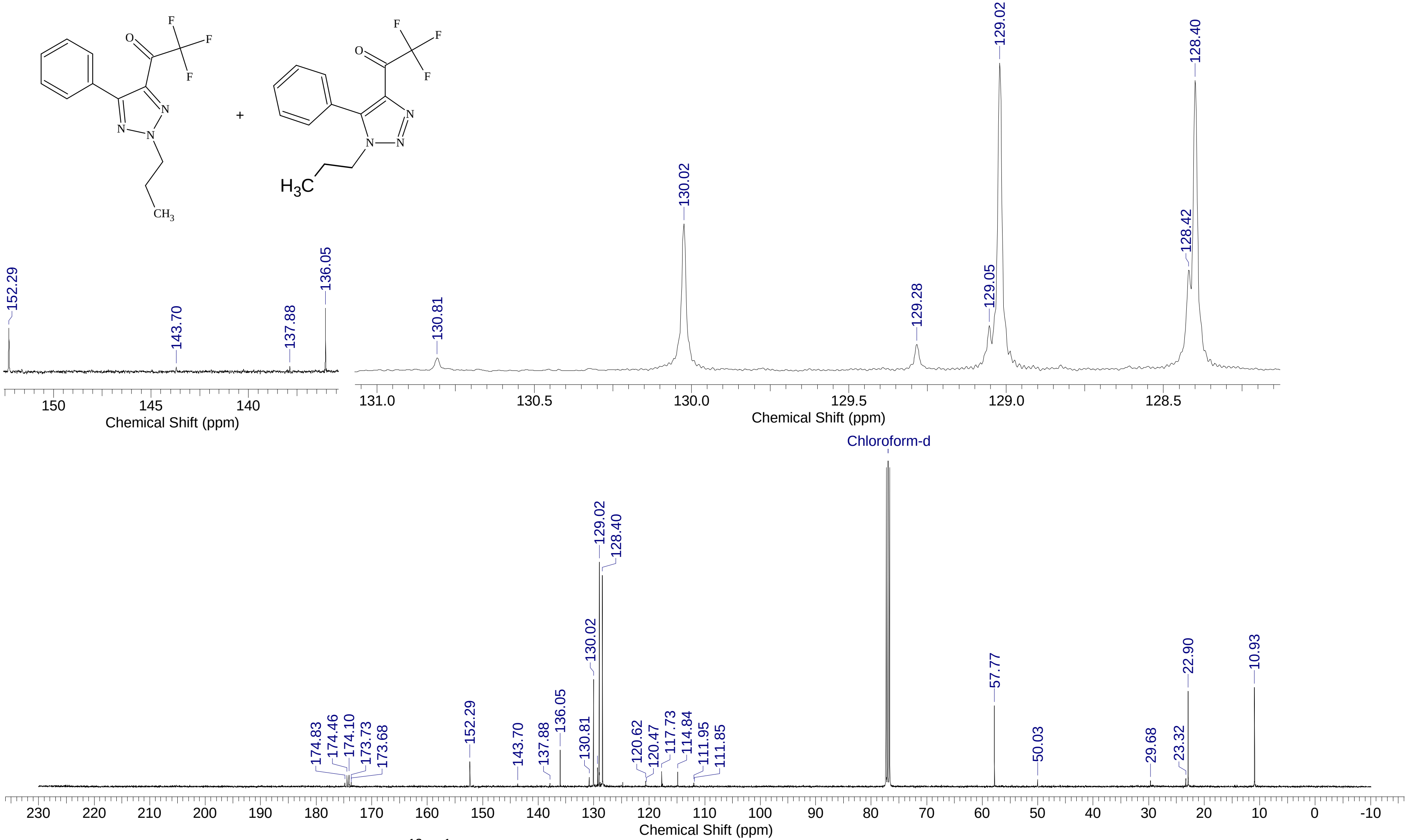

$^{13}C\{^{1}H\}$ NMR spectrum of mixture of **3g** and **4g** (100.6 MHz, $CDCl_3$)

14 May 2022

Acquisition Time (sec)	0.6783	***Comment***	Imported from UXNMR.			***Date***	22 Mar 2022 14:03:50
File Name	C:\DOCS\OUTPUT_301\2022\03.март\SZA-BM-2458.C_002001r					***Frequency (MHz)***	100.61
Nucleus	13C	***Number of Transients***	2145	***Original Points Count***	16384	***Points Count***	131072
Pulse Sequence	zgpg30	***Solvent***	DMSO-D6	***Sweep Width (Hz)***	24154.59	***Temperature (degree C)***	27.000

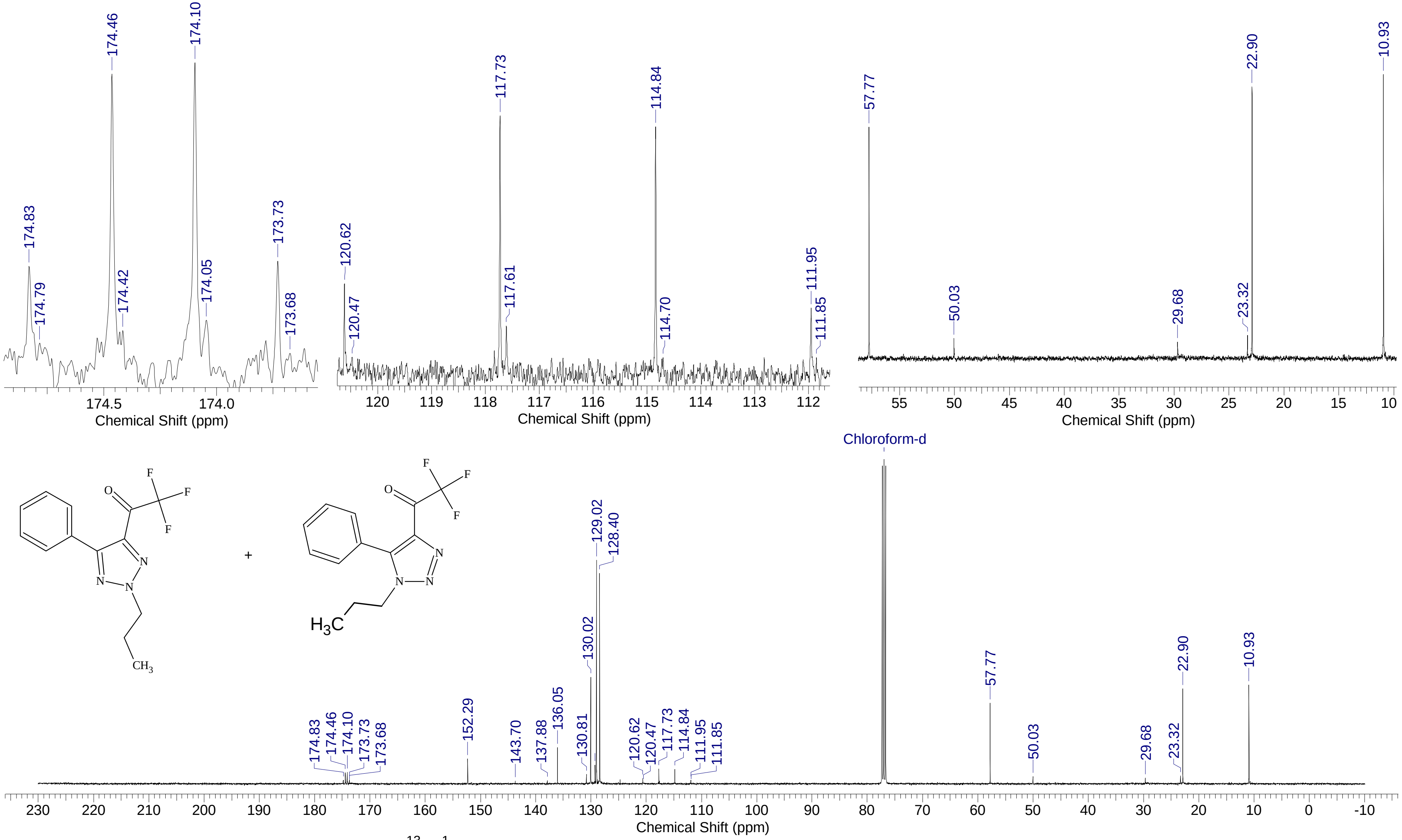

$^{13}C\{^1H\}$ NMR spectrum of mixture of **3g** and **4g** (100.6 MHz, $CDCl_3$)

16 May 2022

Acquisition Time (sec)	4.0894	***Comment***	Imported from UXNMR.			***Date***	05 Apr 2022 15:05:28
File Name	C:\DOCS\OUTPUT_301\2022\04.апрель\BM-2479-1.H_001001r					***Frequency (MHz)***	400.13
Nucleus	1H	***Number of Transients***	4	***Original Points Count***	32768	***Points Count***	131072
Pulse Sequence	zg30	***Solvent***	BENZENE-D6	***Sweep Width (Hz)***	8012.82	***Temperature (degree C)***	27.000

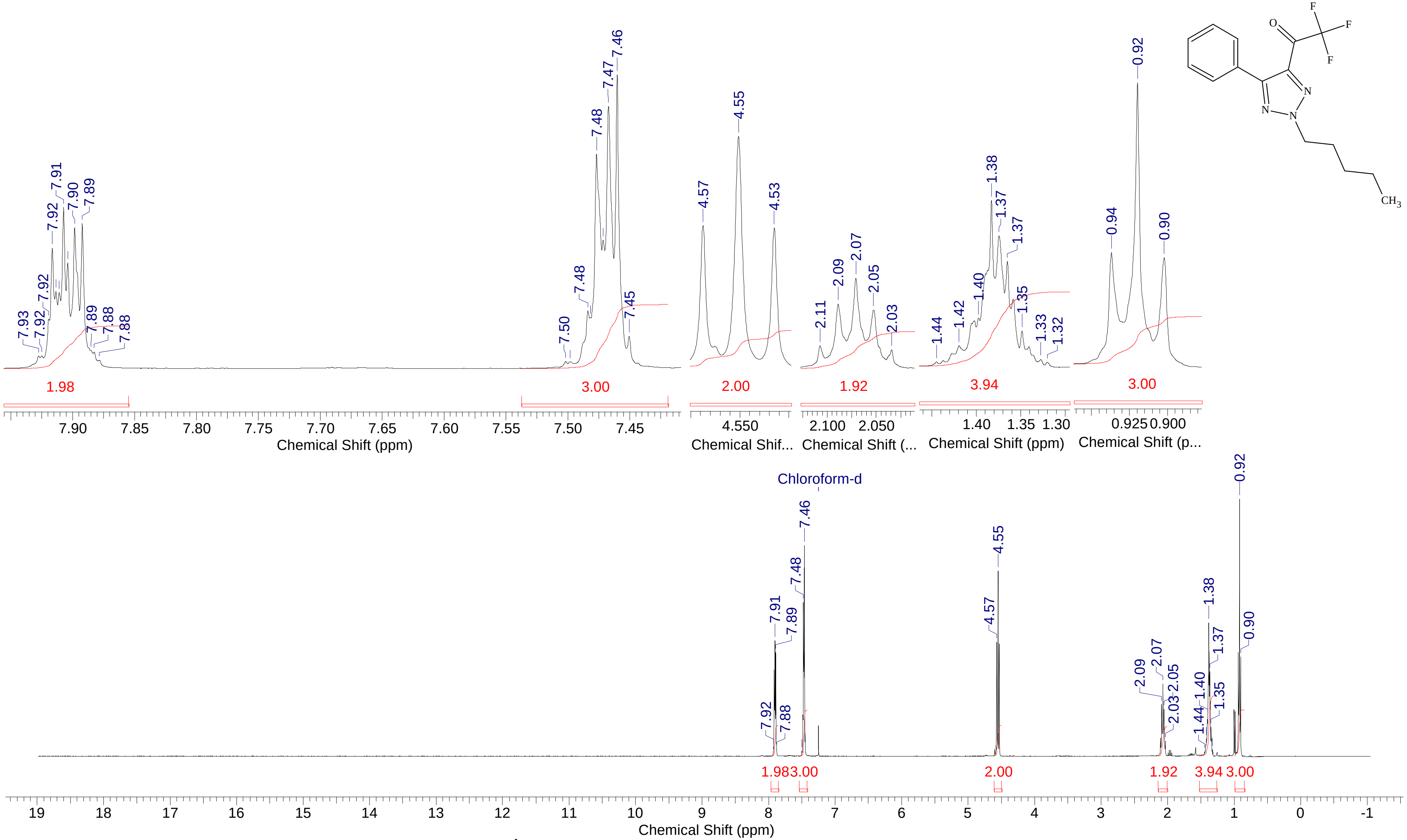

^{1}H NMR spectrum of **3h** (400.1 MHz, $CDCl_3$)

16 May 2022

Acquisition Time (sec)	1.7433	***Comment***	Imported from UXNMR.			***Date***	05 Apr 2022 15:17:16
File Name	C:\DOCS\OUTPUT_301\2022\04.апрель\BM-2479-1.F_005001r					***Frequency (MHz)***	376.50
Nucleus	19F	***Number of Transients***	4	***Original Points Count***	131072	***Points Count***	262144
Pulse Sequence	zgflqn	***Solvent***	CHLOROFORM-D			***Sweep Width (Hz)***	75187.97
Temperature (degree C)	27.000						

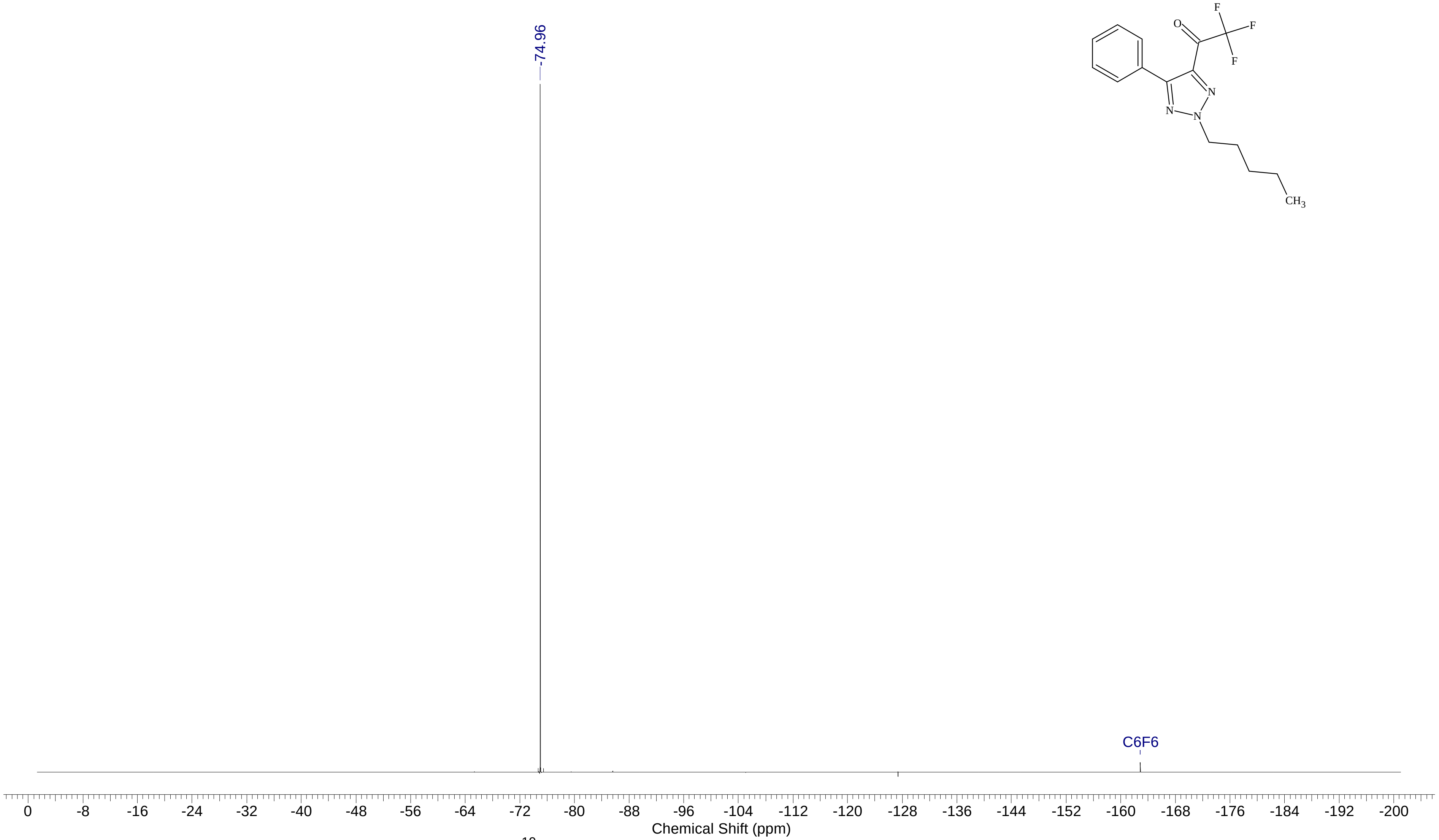

^{19}F NMR spectrum of **3h** (376.5 MHz, $CDCl_3$)

16 May 2022

Acquisition Time (sec)	0.6783	Comment	Imported from UXNMR.			Date	05 Apr 2022 15:13:32
File Name	C:\DOCS\OUTPUT_301\2022\04.апрель\BM-2479-1.C_002001r					Frequency (MHz)	100.61
Nucleus	13C	Number of Transients	193	Original Points Count	16384	Points Count	131072
Pulse Sequence	zgpg30	Solvent	CHLOROFORM-D			Sweep Width (Hz)	24154.59
Temperature (degree C)	27.000						

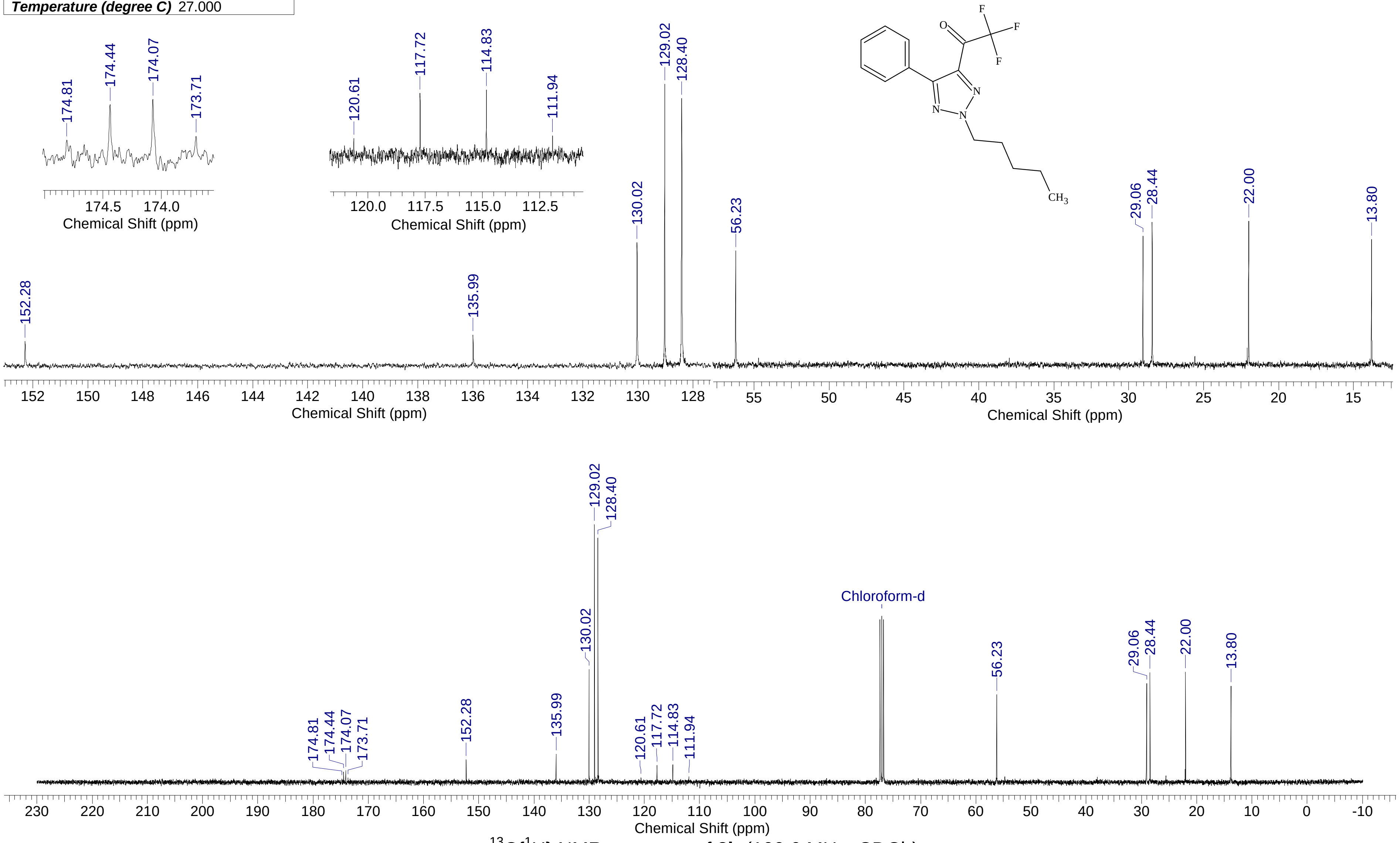

$^{13}C\{^{1}H\}$ NMR spectrum of **3h** (100.6 MHz, $CDCl_3$)

16 May 2022

Acquisition Time (sec)	4.0894	***Comment***	Imported from UXNMR.			***Date***	05 Apr 2022 15:01:32
File Name	C:\DOCS\OUTPUT_301\2022\04.апрель\BM-2479-2.H_001001r					***Frequency (MHz)***	400.13
Nucleus	1H	***Number of Transients***	4	***Original Points Count***	32768	***Points Count***	131072
Pulse Sequence	zg30	***Solvent***	BENZENE-D6	***Sweep Width (Hz)***	8012.82	***Temperature (degree C)***	27.000

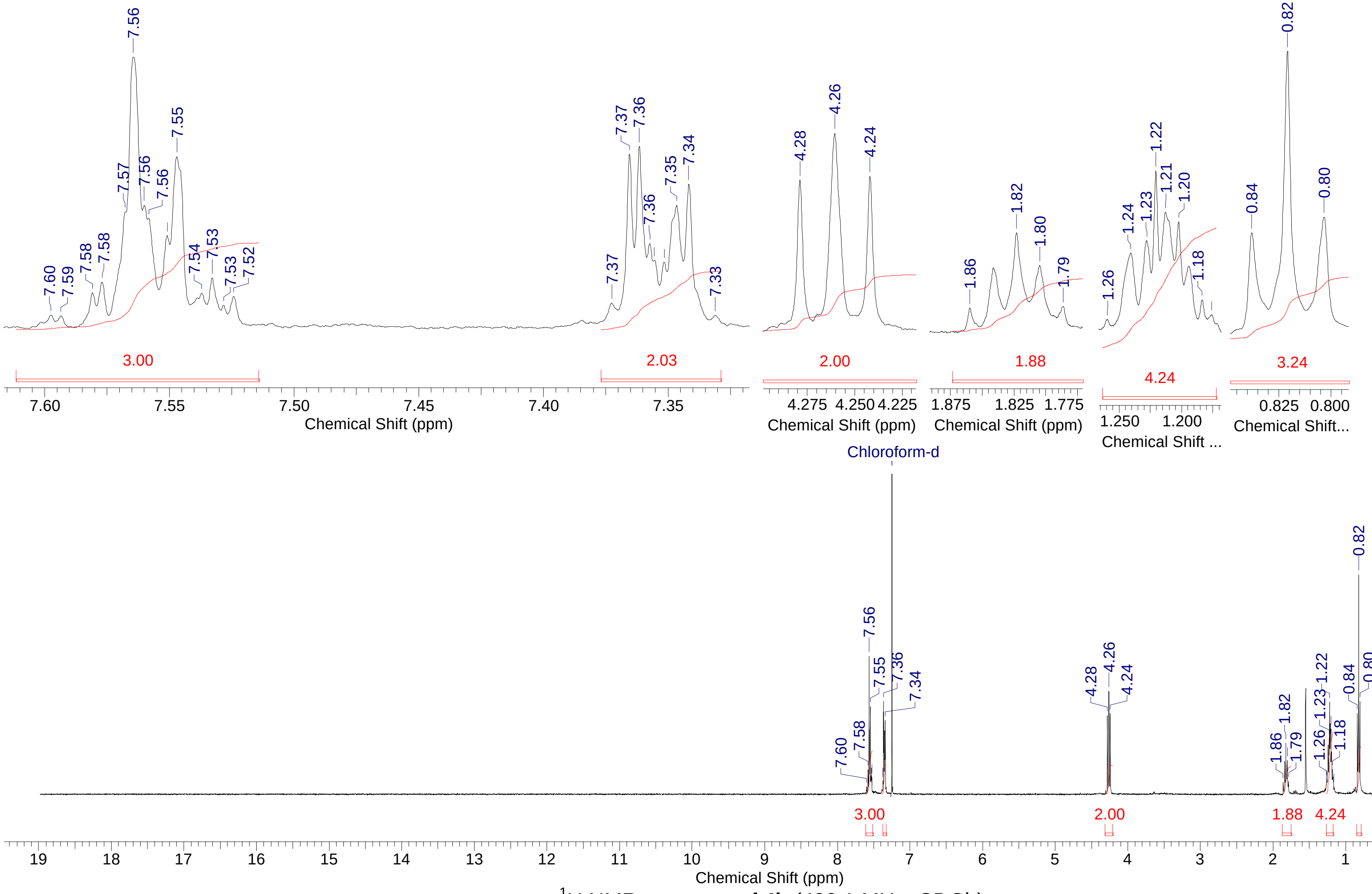

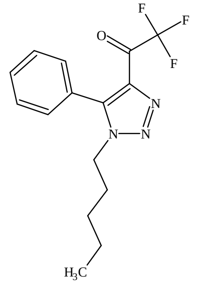

^{1}H NMR spectrum of **4h** (400.1 MHz, $CDCl_3$)

Acquisition Time (sec)	1.7433	***Comment***	Imported from UXNMR.			***Date***	05 Apr 2022 15:30:54
File Name	C:\DOCS\OUTPUT_301\2022\04.апрель\BM-2479-2.F_005001r					***Frequency (MHz)***	376.50
Nucleus	19F	***Number of Transients***	4	***Original Points Count***	131072	***Points Count***	262144
Pulse Sequence	zgflqn	***Solvent***	CHLOROFORM-D			***Sweep Width (Hz)***	75187.97
Temperature (degree C)	27.000						

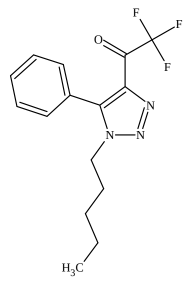

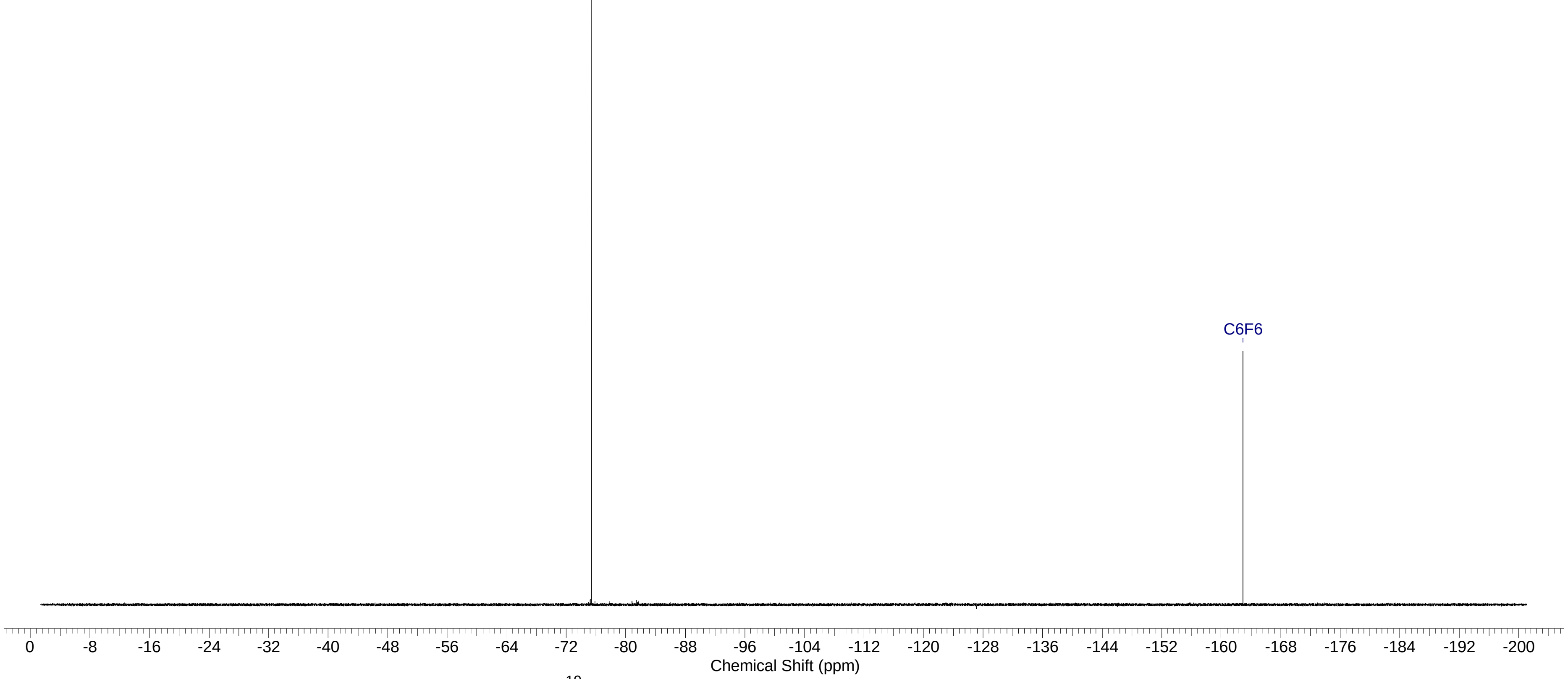

^{19}F NMR spectrum of **4h** (376.5 MHz, $CDCl_3$)

16 May 2022

Acquisition Time (sec)	0.6783	***Comment***	Imported from UXNMR.	***Date***	12 Apr 2022 16:03:14
File Name	C:\DOCS\OUTPUT_301\2022\04.апрель\BM-2479-2.C_002001r			***Frequency (MHz)***	100.61
Nucleus	13C	***Number of Transients***	307 ***Original Points Count*** 16384	***Points Count***	131072
Pulse Sequence	zgpg30	***Solvent***	CHLOROFORM-D	***Sweep Width (Hz)***	24154.59
Temperature (degree C)	27.000				

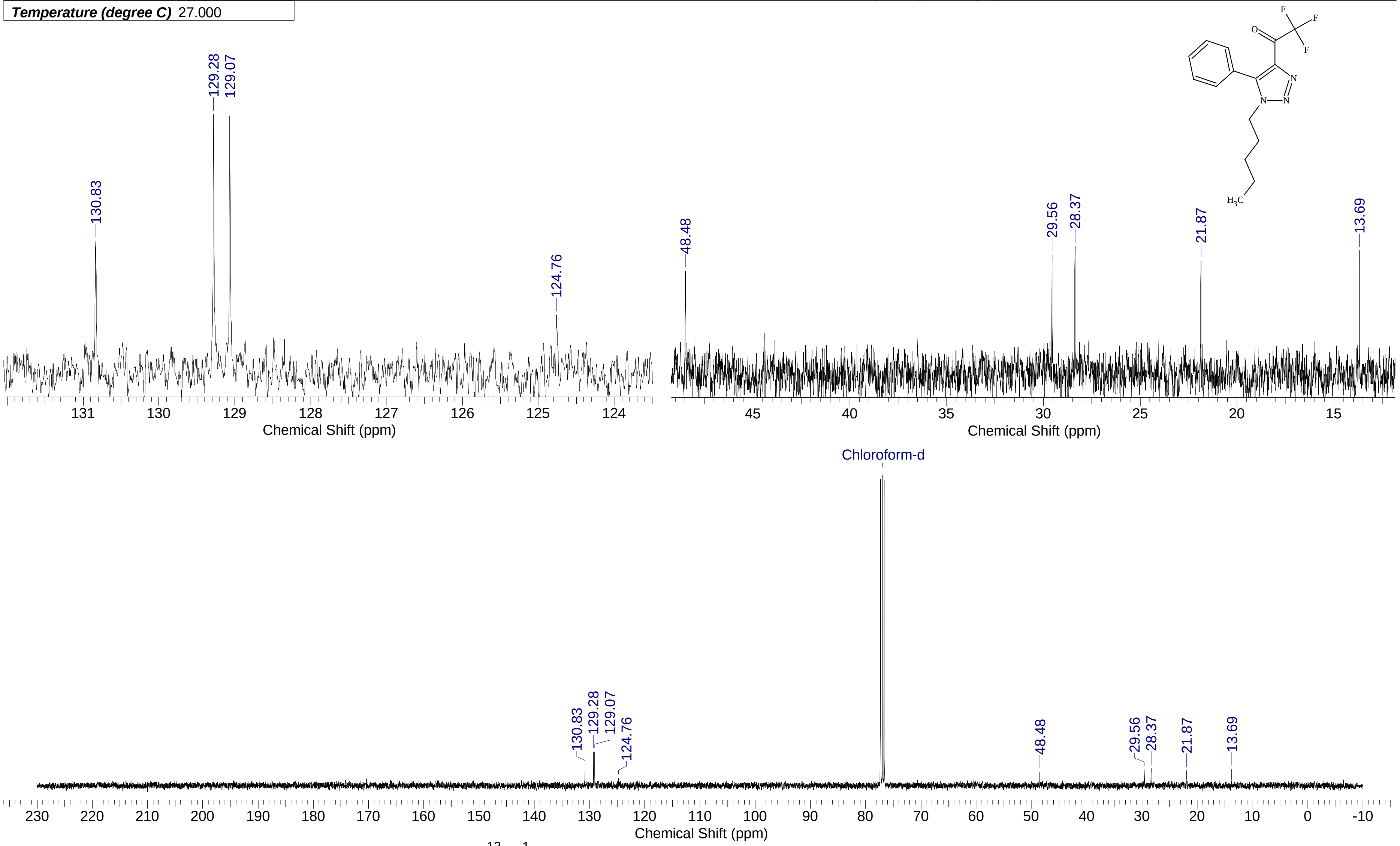

$^{13}C\{^1H\}$ NMR spectrum of **4h** (100.6 MHz, $CDCl_3$)

16 May 2022

Acquisition Time (sec)	4.0894	Comment	Imported from UXNMR.			Date	25 Mar 2022 15:48:10
File Name	C:\DOCS\OUTPUT_301\2022\03.март\BM-2464-2.H_001001r					Frequency (MHz)	400.13
Nucleus	1H	Number of Transients	4	Original Points Count	32768	Points Count	131072
Pulse Sequence	zg30	Solvent	CHLOROFORM-D			Sweep Width (Hz)	8012.82
Temperature (degree C)	27.000						

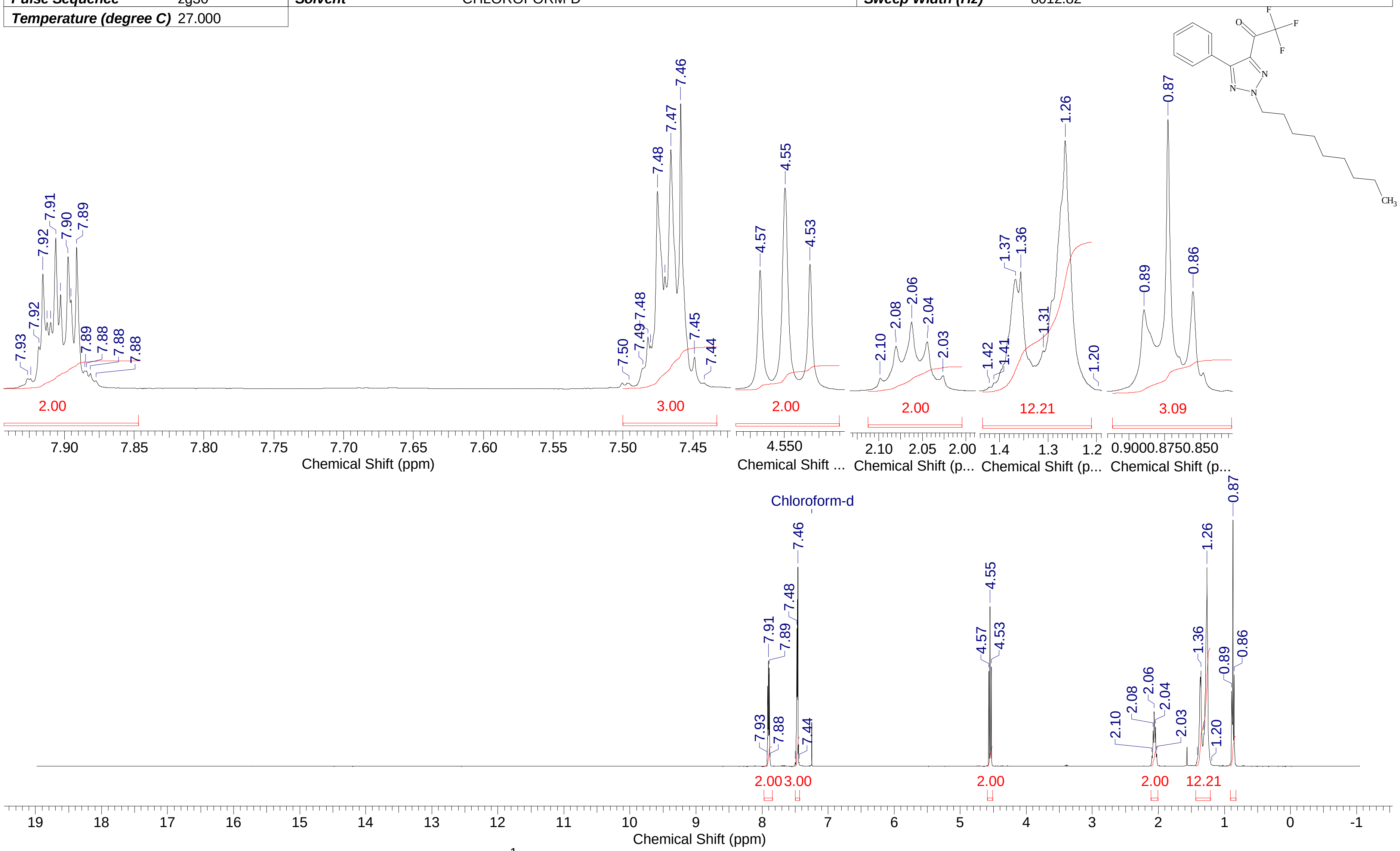

^{1}H NMR spectrum of **3i** (400.1 MHz, $CDCl_3$)

Acquisition Time (sec)	1.7433	Comment	Imported from UXNMR.			Date	25 Mar 2022 15:58:36
File Name	C:\DOCS\OUTPUT_301\2022\03.март\BM-2464-2.F_005001r			Frequency (MHz)	376.50	Nucleus	19F
Number of Transients	4	Original Points Count	131072	Points Count	262144	Pulse Sequence	zgflqn
Solvent	CHLOROFORM-D			Sweep Width (Hz)	75187.97	Temperature (degree C)	27.000

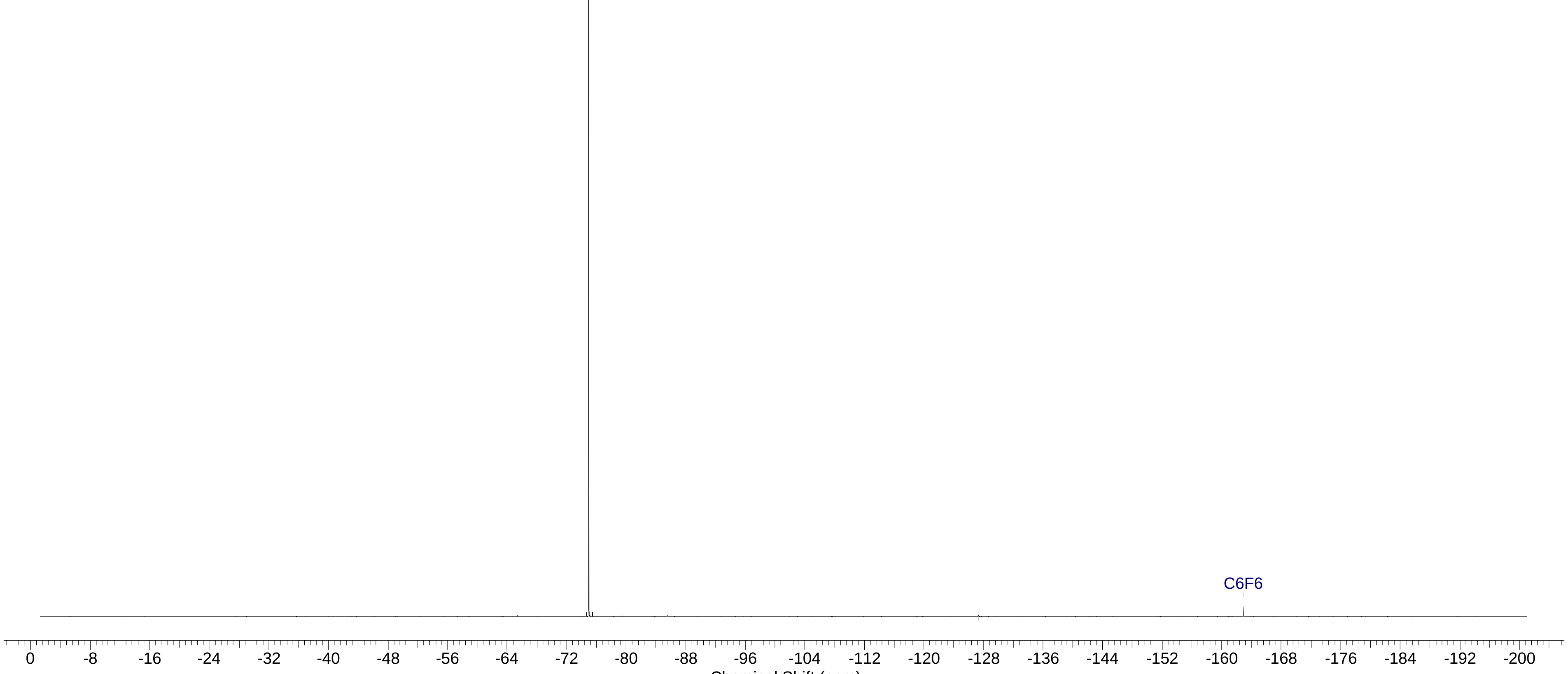

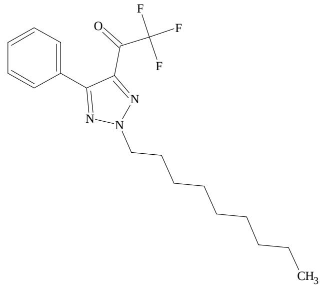

^{19}F NMR spectrum of **3i** (376.5 MHz, $CDCl_3$)

16 May 2022

Acquisition Time (sec)	0.6783	***Comment***	Imported from UXNMR.			***Date***	26 Mar 2022 13:44:02
File Name	C:\DOCS\BM\ЯМР_СОСЕДИ\2022\bm220326\BM-2464-2_002001r					***Frequency (MHz)***	100.61
Nucleus	13C	***Number of Transients***	160	***Original Points Count***	16384	***Points Count***	131072
Pulse Sequence	zgpg30	***Solvent***	CHLOROFORM-D			***Sweep Width (Hz)***	24154.59
Temperature (degree C)	27.000						

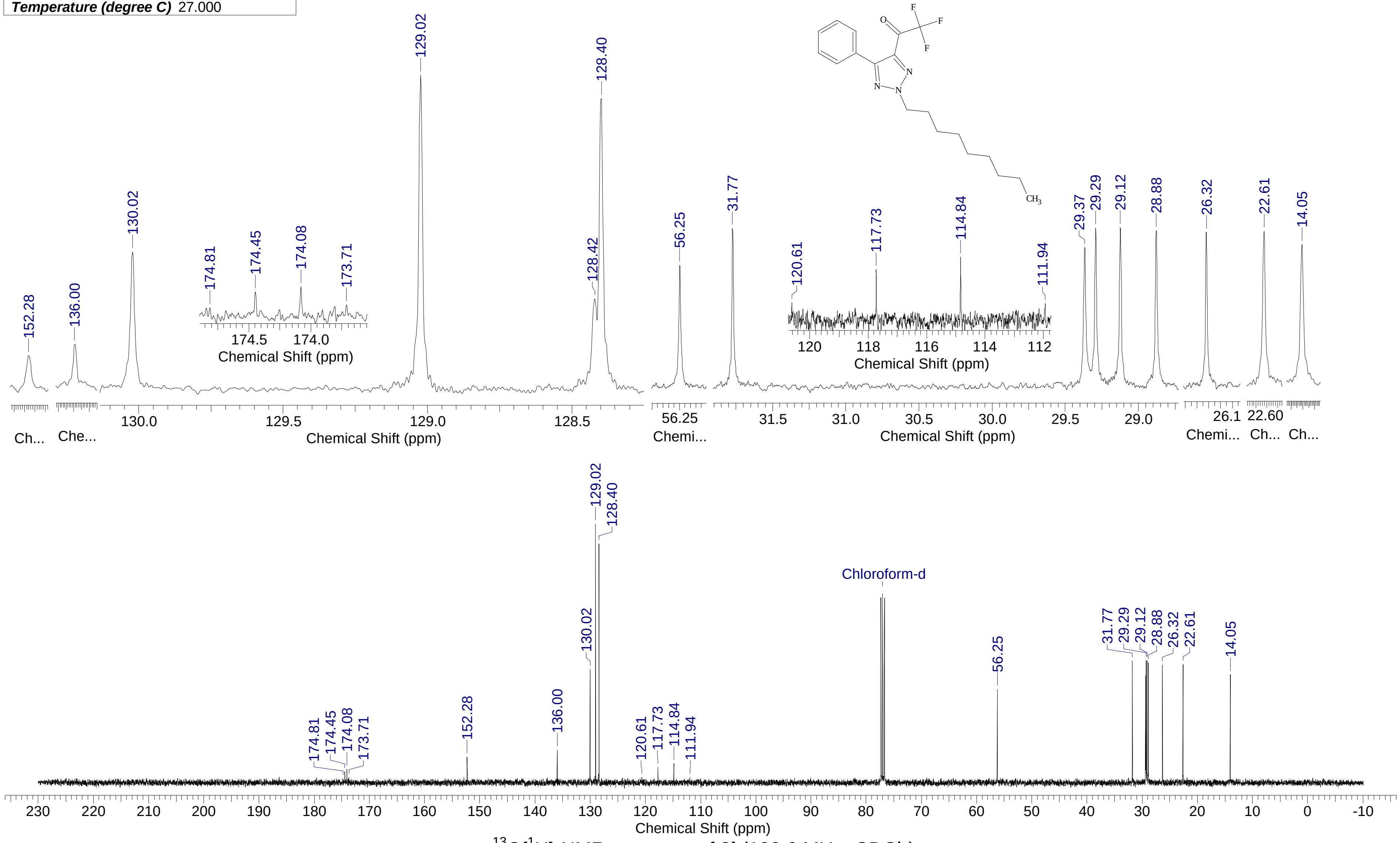

$^{13}C\{^1H\}$ NMR spectrum of **3i** (100.6 MHz, $CDCl_3$)

16 May 2022

**Acquisition Time (sec)**	4.0894	**Comment**	Imported from UXNMR.			**Date**	25 Mar 2022 15:50:02
**File Name**	C:\DOCS\OUTPUT_301\2022\03.март\BM-2464-3.H_001001r					**Frequency (MHz)**	400.13
**Nucleus**	1H	**Number of Transients**	4	**Original Points Count**	32768	**Points Count**	131072
**Pulse Sequence**	zg30	**Solvent**	CHLOROFORM-D			**Sweep Width (Hz)**	8012.82
**Temperature (degree C)**	27.000						

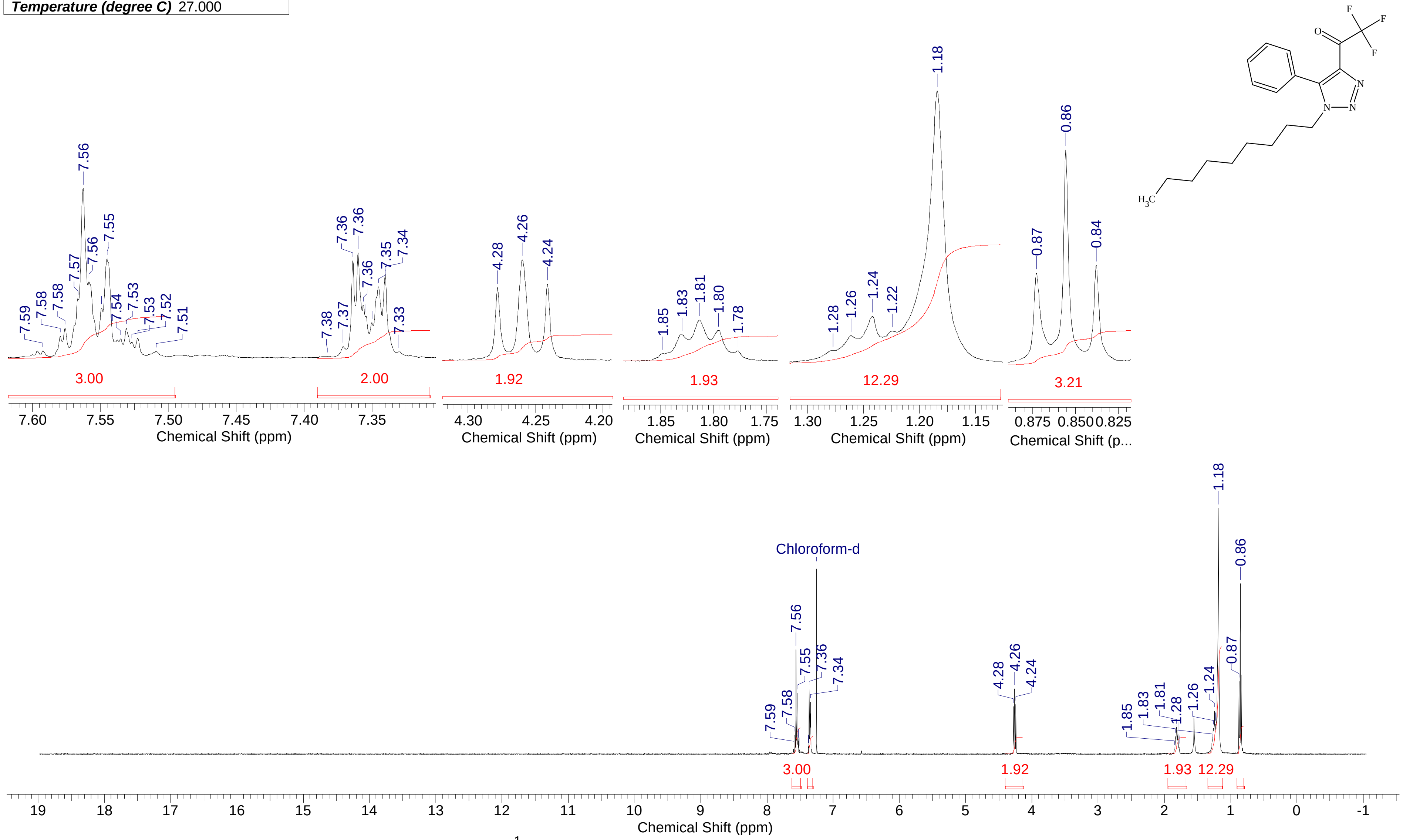

^{1}H NMR spectrum of **4i** (400.1 MHz, $CDCl_3$)

16 May 2022

Acquisition Time (sec)	1.7433	***Comment***	Imported from UXNMR.			***Date***	25 Mar 2022 15:57:02
File Name	C:\DOCS\OUTPUT_301\2022\03.март\BM-2464-3.F_005001r			***Frequency (MHz)***	376.50	***Nucleus***	19F
Number of Transients	4	***Original Points Count***	131072	***Points Count***	262144	***Pulse Sequence***	zgflqn
Solvent	CHLOROFORM-D			***Sweep Width (Hz)***	75187.97	***Temperature (degree C)***	27.000

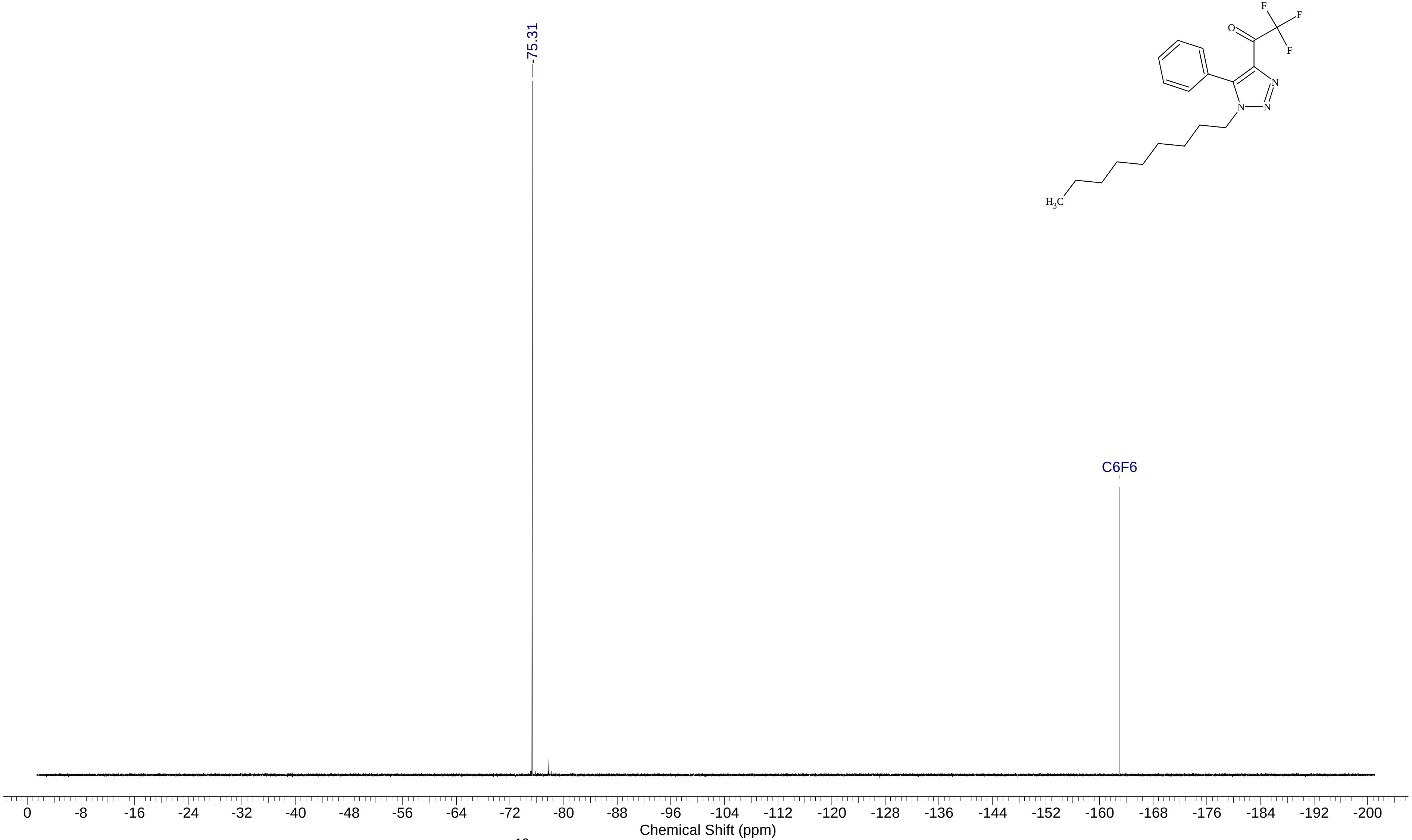

^{19}F NMR spectrum of **4i** (376.5 MHz, $CDCl_3$)

16 May 2022

Acquisition Time (sec)	0.6783	***Comment***	Imported from UXNMR.	***Date***	29 Mar 2022 16:07:40
File Name	C:\DOCS\OUTPUT_301\2022\03.март\BM-2464-3.C_002001r	***Frequency (MHz)***	100.61	***Nucleus***	13C
Number of Transients	175	***Original Points Count*** 16384	***Points Count*** 131072	***Pulse Sequence***	zgpg30
Solvent	CHLOROFORM-D	***Sweep Width (Hz)***	24154.59	***Temperature (degree C)***	27.000

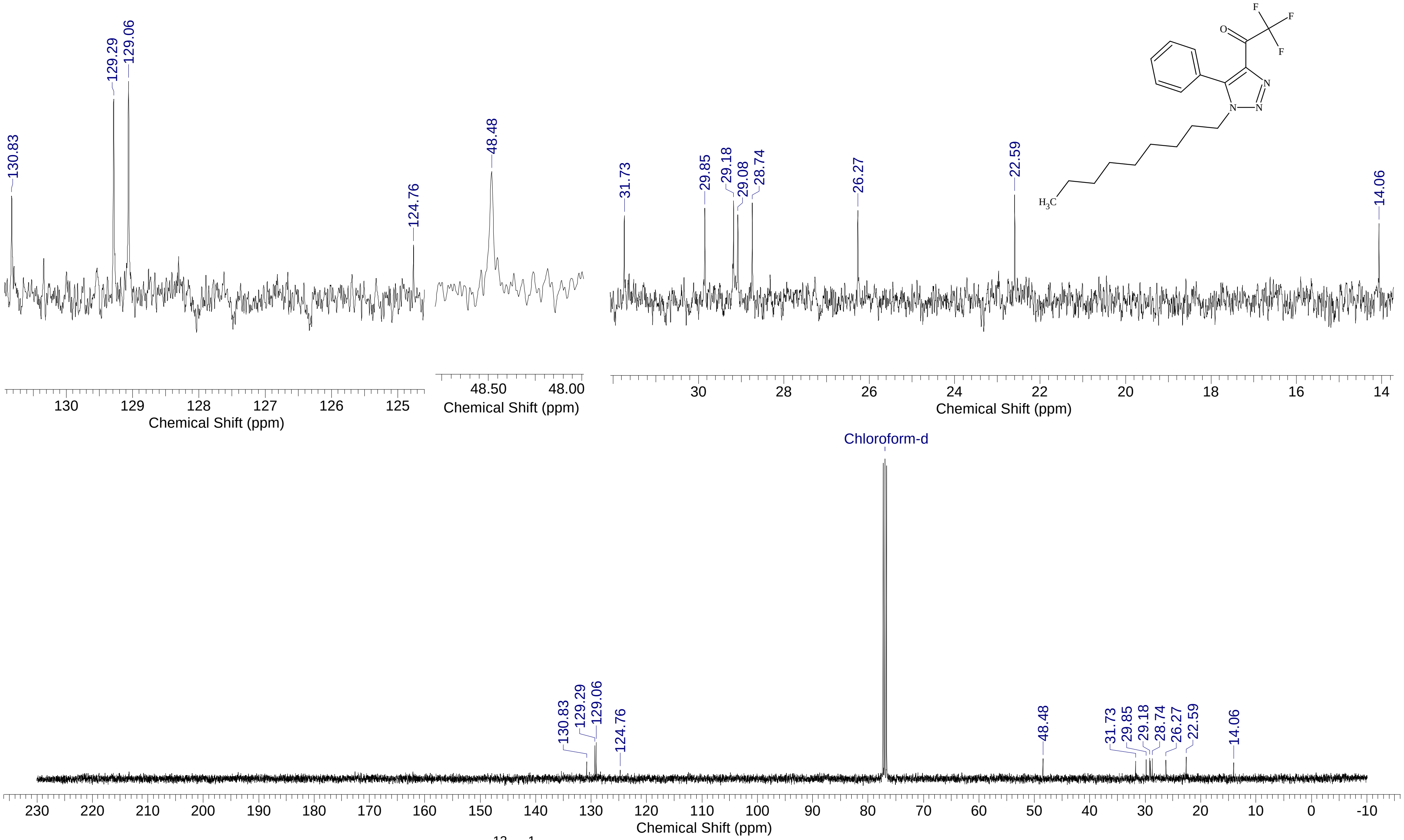

$^{13}C\{^{1}H\}$ NMR spectrum of **4i** (100.6 MHz, $CDCl_3$)

16 May 2022

Acquisition Time (sec)	4.0894	Comment	Imported from UXNMR.			Date	06 Apr 2022 17:11:40
File Name	C:\DOCS\OUTPUT_301\2022\04.апрель\SZA-BM-2481R.H_001001r					Frequency (MHz)	400.13
Nucleus	1H	Number of Transients	4	Original Points Count	32768	Points Count	131072
Pulse Sequence	zg30	Solvent	BENZENE-D6	Sweep Width (Hz)	8012.82	Temperature (degree C)	27.000

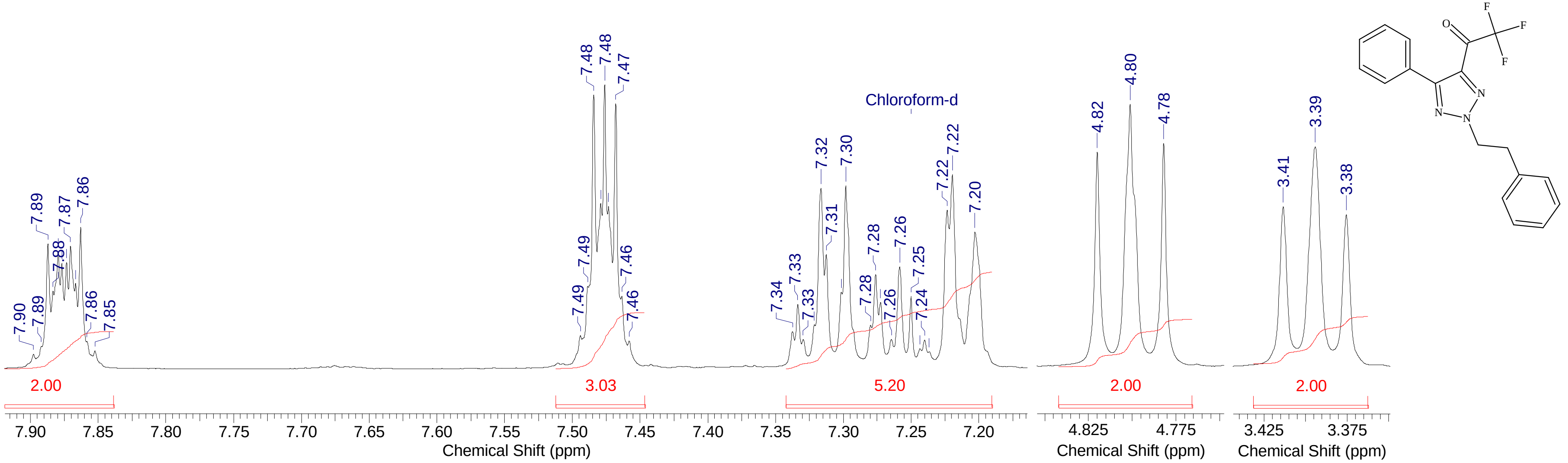

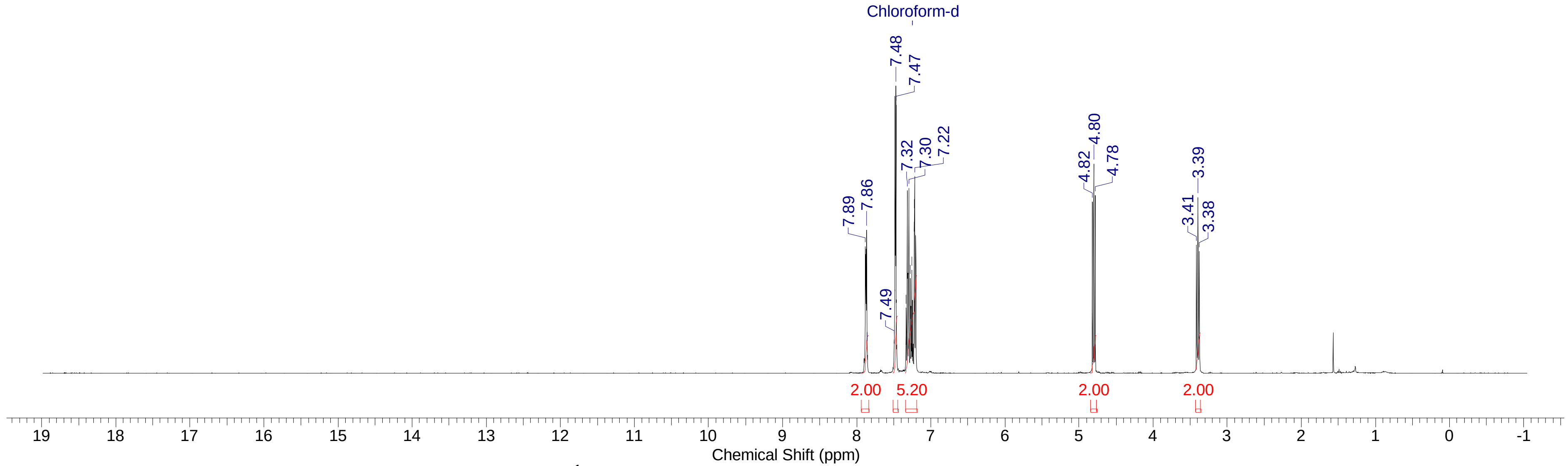

^{1}H NMR spectrum of **3j** (400.1 MHz, $CDCl_3$)

16 May 2022

Acquisition Time (sec)	1.7433	***Comment***	Imported from UXNMR.			***Date***	06 Apr 2022 17:37:22
File Name	C:\DOCS\OUTPUT_301\2022\04.апрель\SZA-BM-2481R.F_005001r					***Frequency (MHz)***	376.50
Nucleus	19F	***Number of Transients***	16	***Original Points Count***	131072	***Points Count***	262144
Pulse Sequence	zgflqn	***Solvent***	CHLOROFORM-D			***Sweep Width (Hz)***	75187.97
Temperature (degree C)	27.000						

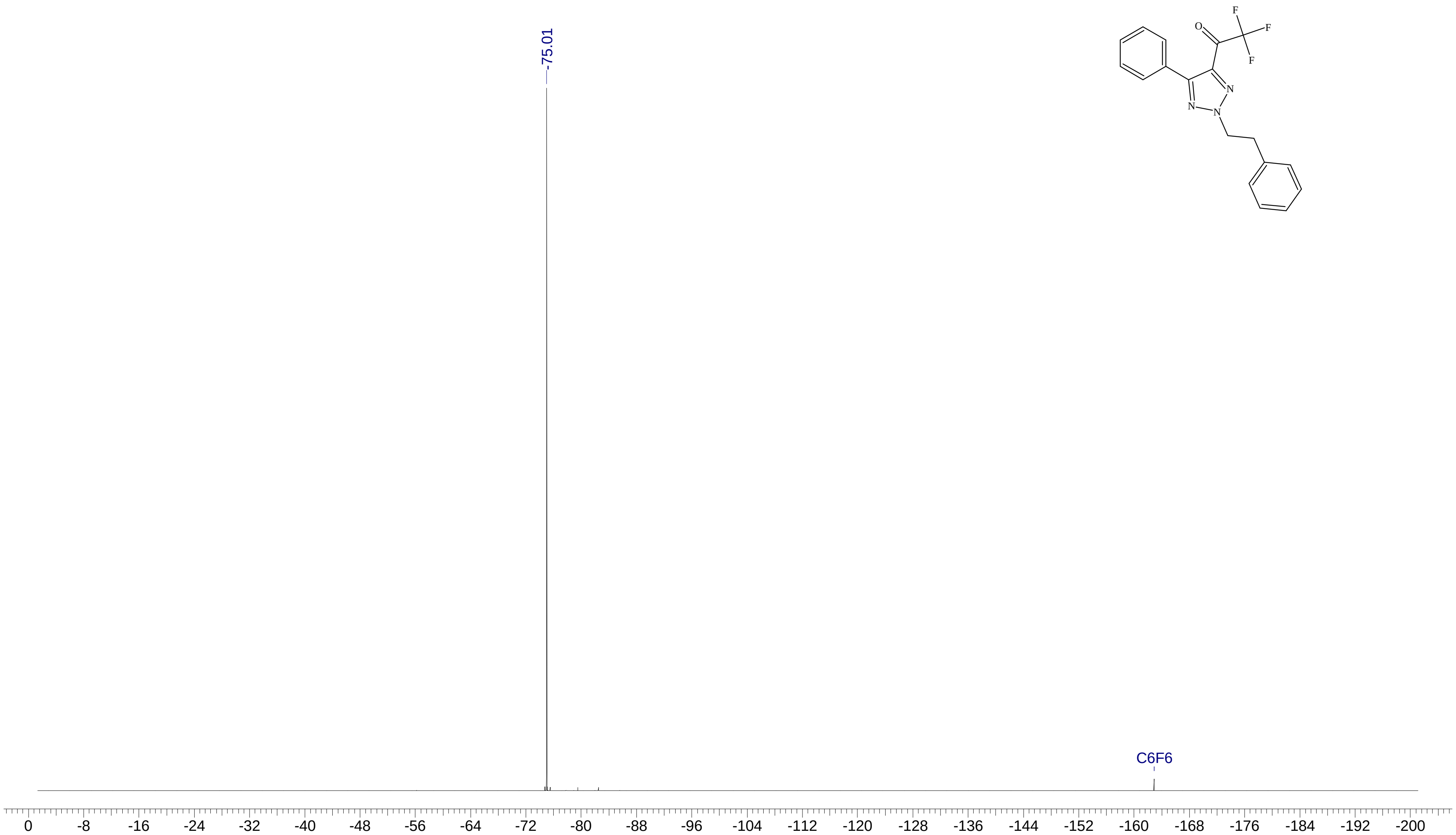

^{19}F NMR spectrum of **3j** (376.5 MHz, $CDCl_3$)

16 May 2022

Acquisition Time (sec)	0.6783	Comment	Imported from UXNMR.			Date	06 Apr 2022 17:33:28
File Name	C:\DOCS\OUTPUT_301\2022\04.апрель\SZA-BM-2481R.C_002001r					Frequency (MHz)	100.61
Nucleus	13C	Number of Transients	521	Original Points Count	16384	Points Count	131072
Pulse Sequence	zgpg30	Solvent	CHLOROFORM-D			Sweep Width (Hz)	24154.59
Temperature (degree C)	27.000						

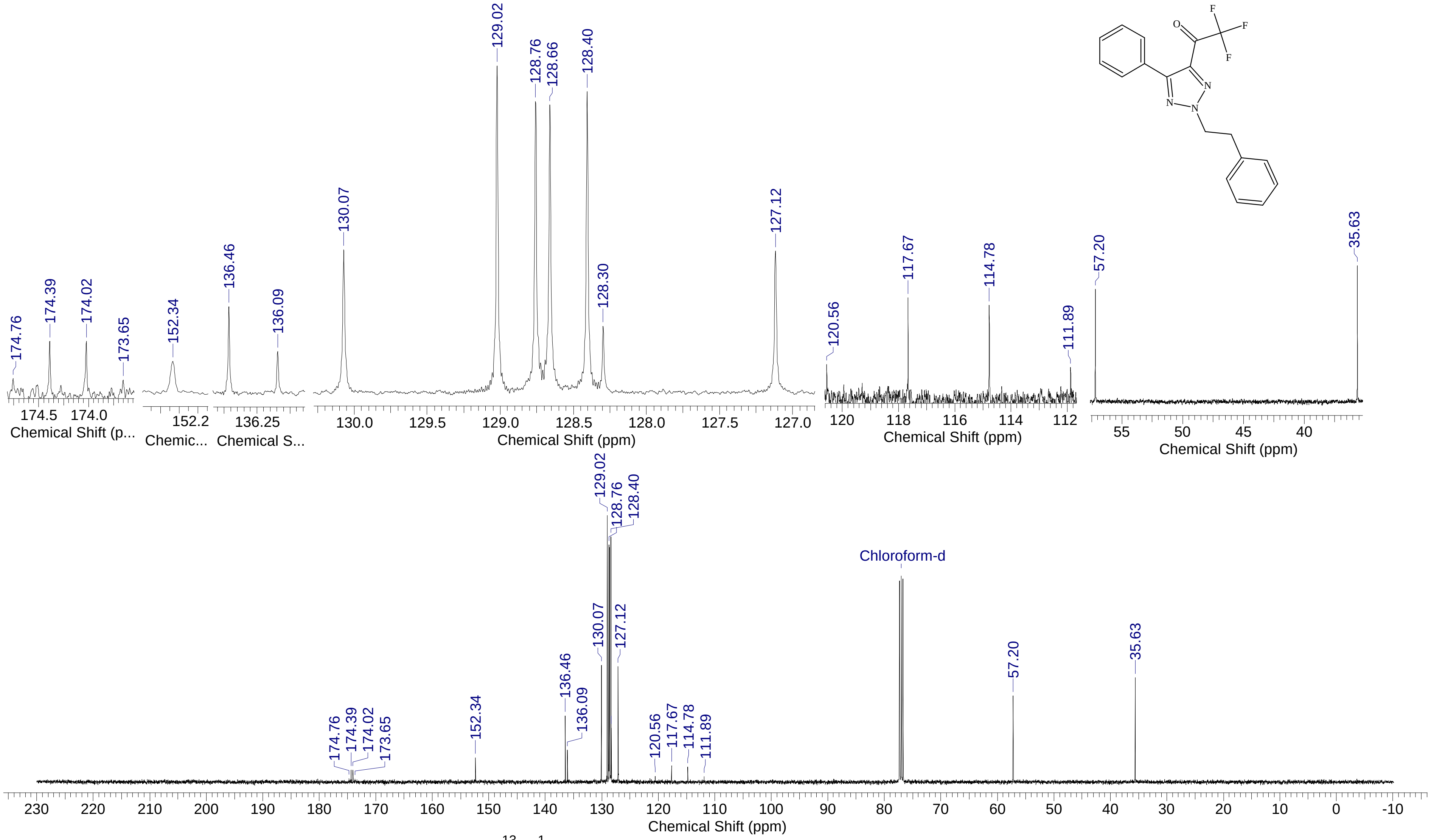

$^{13}C\{^{1}H\}$ NMR spectrum of **3j** (100.6 MHz, $CDCl_3$)

16 May 2022

Acquisition Time (sec)	4.0894	***Comment***	Imported from UXNMR.	***Date***	07 Apr 2022 11:31:38
File Name	C:\DOCS\OUTPUT_301\2022\04.апрель\SZA-BM-2481-11.H.H_001001r			***Frequency (MHz)***	400.13
Nucleus	1H	***Number of Transients***	8 ***Original Points Count*** 32768	***Points Count***	131072
Pulse Sequence	zg30	***Solvent***	CHLOROFORM-D	***Sweep Width (Hz)***	8012.82
Temperature (degree C)	27.000				

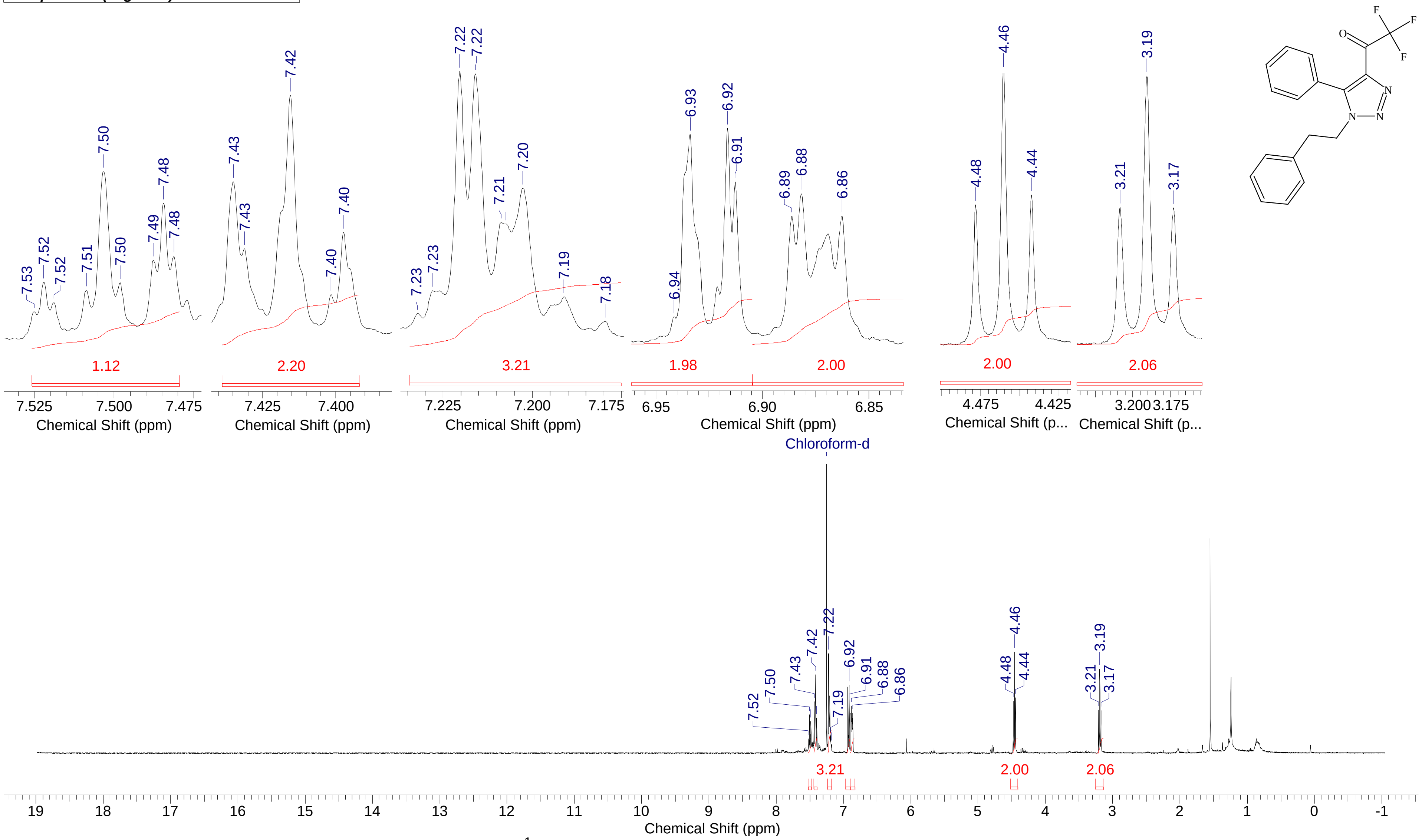

^{1}H NMR spectrum of **4j** (400.1 MHz, $CDCl_3$)

16 May 2022

Acquisition Time (sec)	1.7433	Comment	Imported from UXNMR.			Date	07 Apr 2022 12:32:14
File Name	C:\DOCS\OUTPUT_301\2022\04.апрель\SZA-BM-2481-11.F_005001r					Frequency (MHz)	376.50
Nucleus	19F	Number of Transients	16	Original Points Count	131072	Points Count	262144
Pulse Sequence	zgflqn	Solvent	CHLOROFORM-D			Sweep Width (Hz)	75187.97
Temperature (degree C)	27.000						

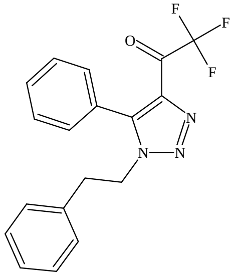

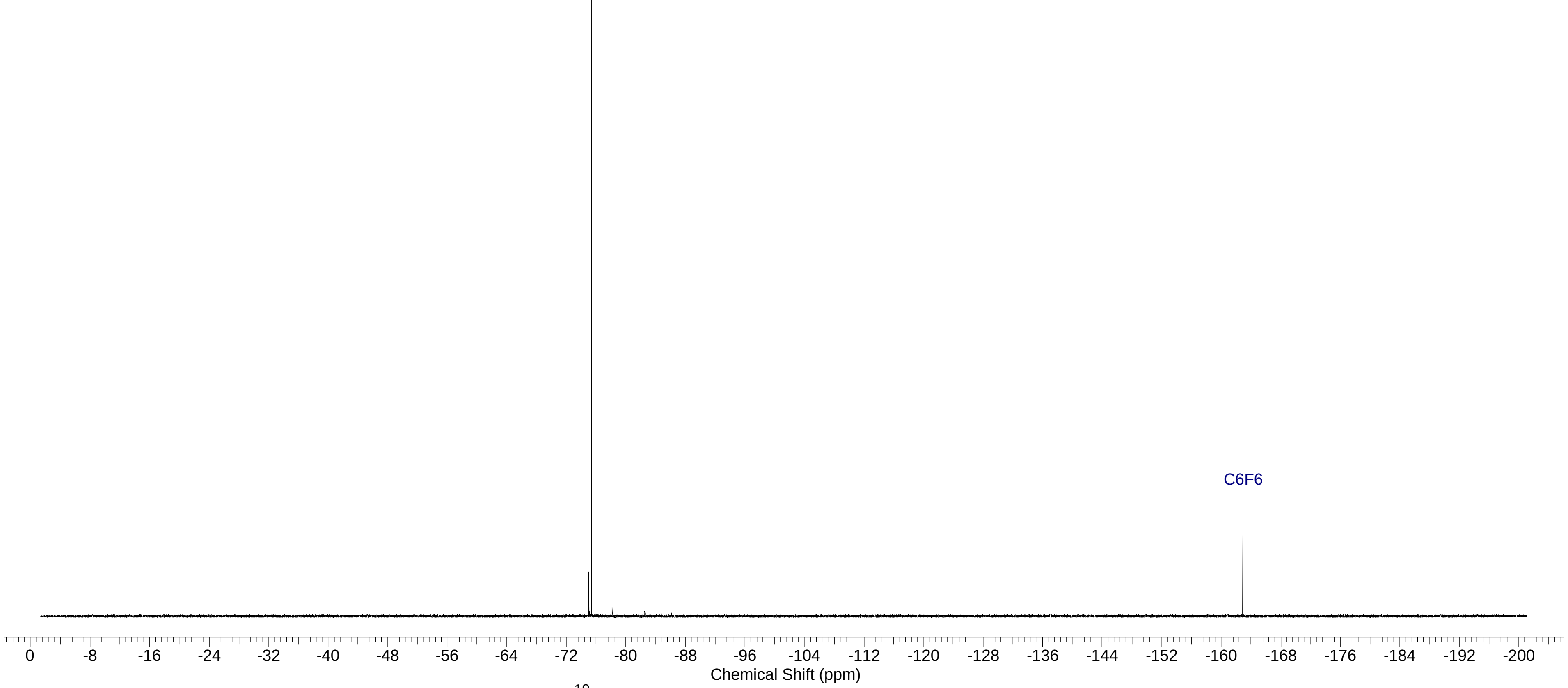

^{19}F NMR spectrum of **4j** (376.5 MHz, $CDCl_3$)

16 May 2022

Acquisition Time (sec)	4.0894	***Comment***	Imported from UXNMR.	***Date***	08 Apr 2022 15:21:08
File Name	C:\DOCS\OUTPUT_301\2022\04.апрель\BM-2480-1.H_001001r			***Frequency (MHz)***	400.13
Nucleus	1H	***Number of Transients***	4 ***Original Points Count*** 32768	***Points Count***	131072
Pulse Sequence	zg30	***Solvent***	CHLOROFORM-D	***Sweep Width (Hz)***	8012.82
Temperature (degree C)	27.000				

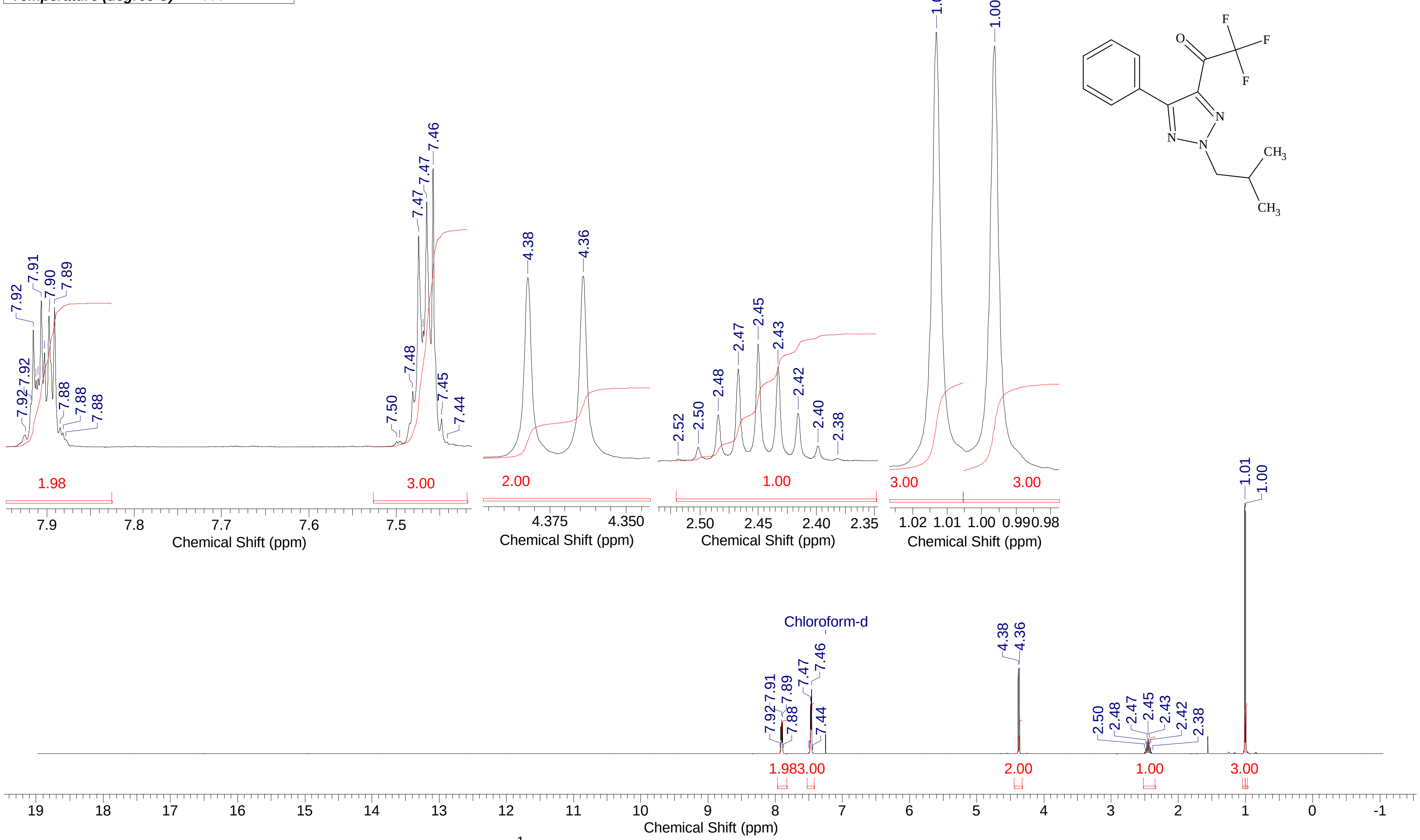

^{1}H NMR spectrum of **3k** (400.1 MHz, $CDCl_3$)

16 May 2022

Acquisition Time (sec)	1.7433	***Comment***	Imported from UXNMR.			***Date***	08 Apr 2022 15:39:14
File Name	C:\DOCS\OUTPUT_301\2022\04.апрель\BM-2480-1.F_005001r					***Frequency (MHz)***	376.50
Nucleus	19F	***Number of Transients***	8	***Original Points Count***	131072	***Points Count***	262144
Pulse Sequence	zgflqn	***Solvent***	CHLOROFORM-D			***Sweep Width (Hz)***	75187.97
Temperature (degree C)	27.000						

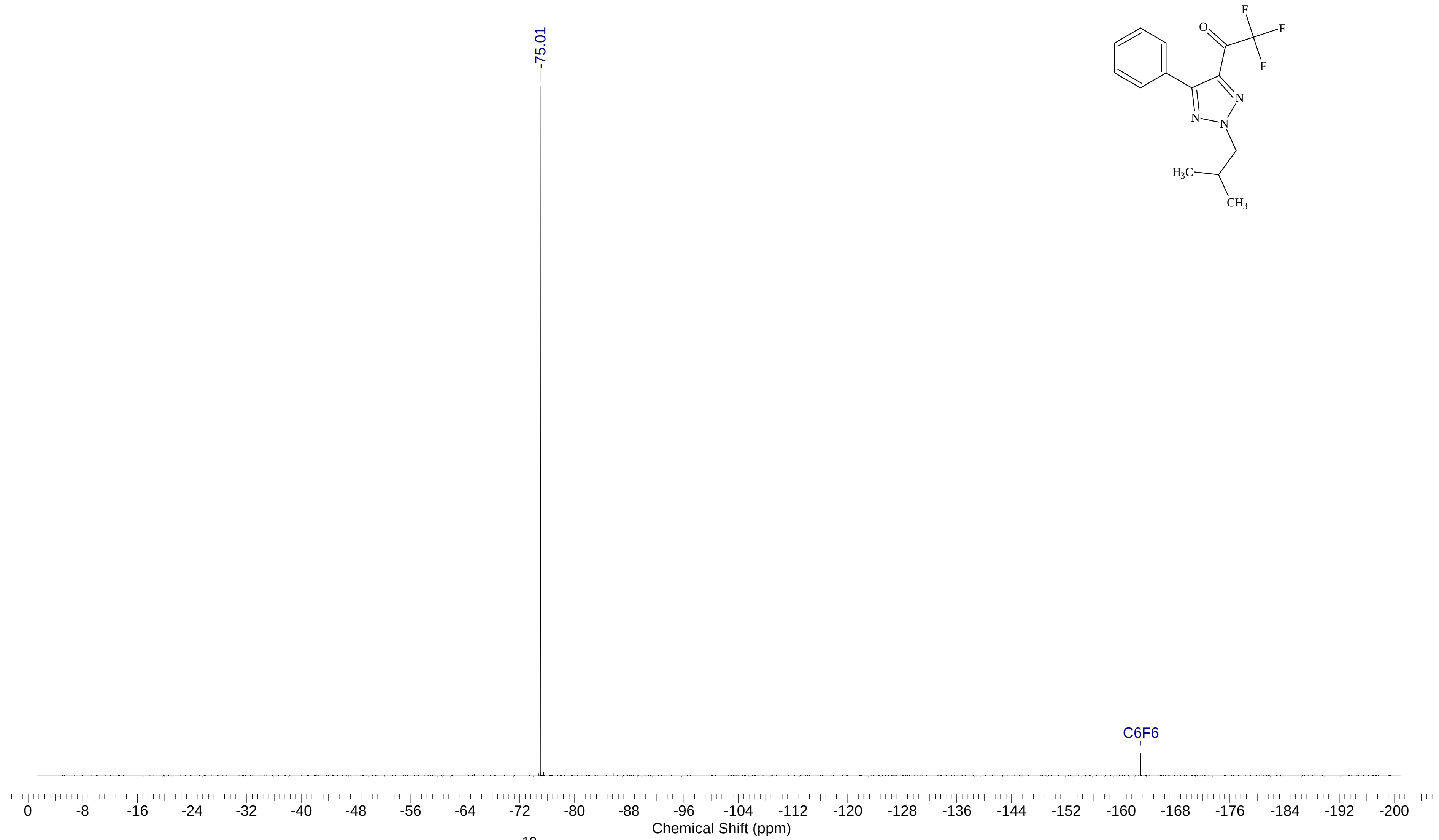

^{19}F NMR spectrum of **3k** (376.5 MHz, $CDCl_3$)

16 May 2022

Acquisition Time (sec)	0.6783	***Comment***	Imported from UXNMR.			***Date***	09 Apr 2022 13:45:48
File Name	C:\DOCS\BM\ЯМР_СОСЕДИ\2022\bm220409\BM-2480-1_002001r					***Frequency (MHz)***	100.61
Nucleus	13C	***Number of Transients***	192	***Original Points Count***	16384	***Points Count***	131072
Pulse Sequence	zgpg30	***Solvent***	CHLOROFORM-D			***Sweep Width (Hz)***	24154.59
Temperature (degree C)	27.000						

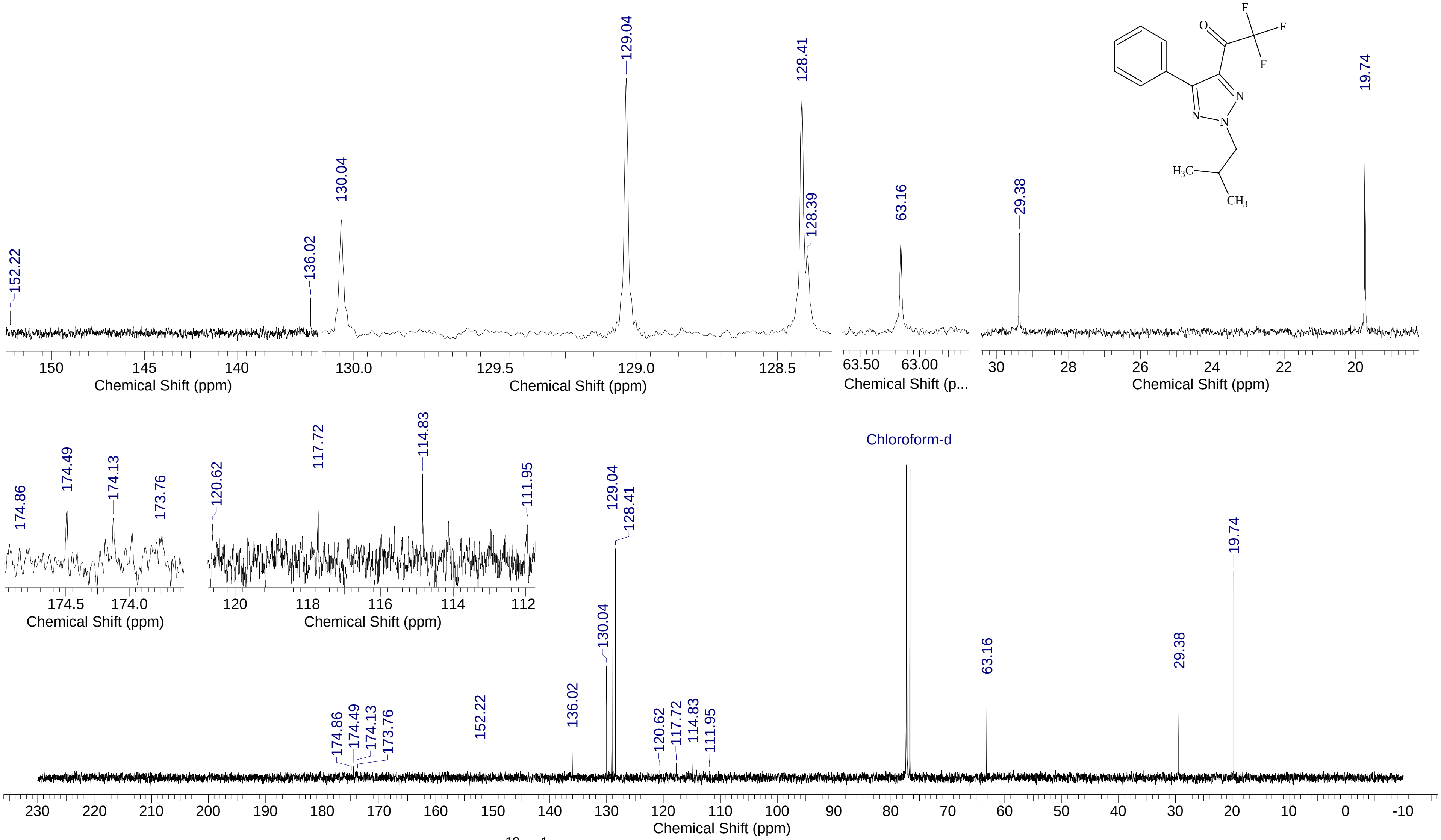

$^{13}C\{^{1}H\}$ NMR spectrum of **3k** (100.6 MHz, $CDCl_3$)

28 Mar 2023

Acquisition Time (sec)	4.0894	***Comment***	Imported from UXNMR.			***Date***	08 Apr 2022 15:22:48
File Name	C:\DOCS\OUTPUT_301\2022\04.апрель\BM-2480-3.H_001001r					***Frequency (MHz)***	400.13
Nucleus	1H	***Number of Transients***	4	***Original Points Count***	32768	***Points Count***	131072
Pulse Sequence	zg30	***Solvent***	CHLOROFORM-D			***Sweep Width (Hz)***	8012.82
Temperature (degree C)	27.000						

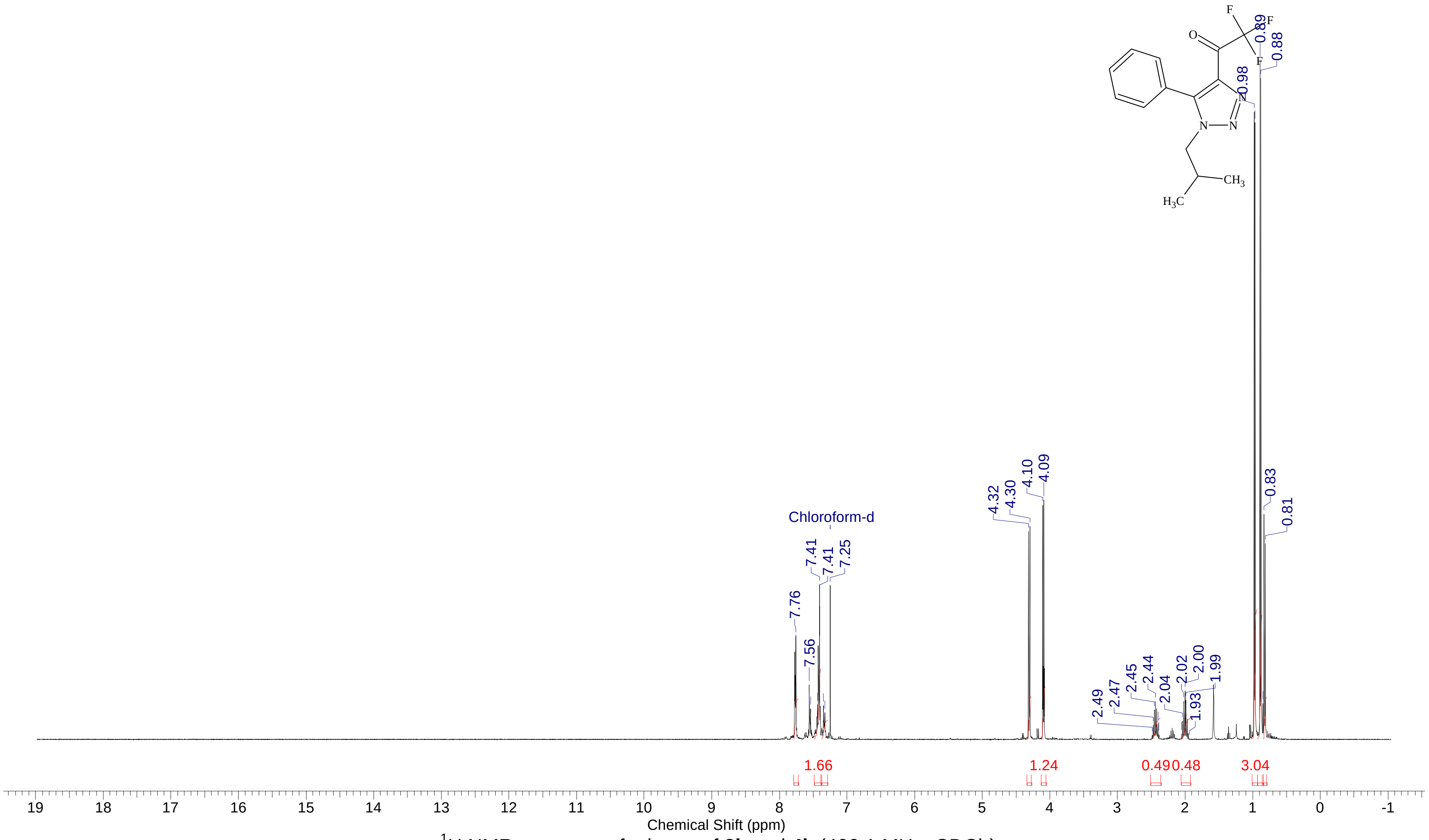

^{1}H NMR spectrum of mixture of **3k** and **4k** (400.1 MHz, $CDCl_3$)

16 May 2022

Acquisition Time (sec)	1.7433	***Comment***	Imported from UXNMR.			***Date***	08 Apr 2022 15:40:58
File Name	C:\DOCS\OUTPUT_301\2022\04.апрель\BM-2480-3.F_005001r					***Frequency (MHz)***	376.50
Nucleus	19F	***Number of Transients***	12	***Original Points Count***	131072	***Points Count***	262144
Pulse Sequence	zgflqn	***Solvent***	CHLOROFORM-D			***Sweep Width (Hz)***	75187.97
Temperature (degree C)	27.000						

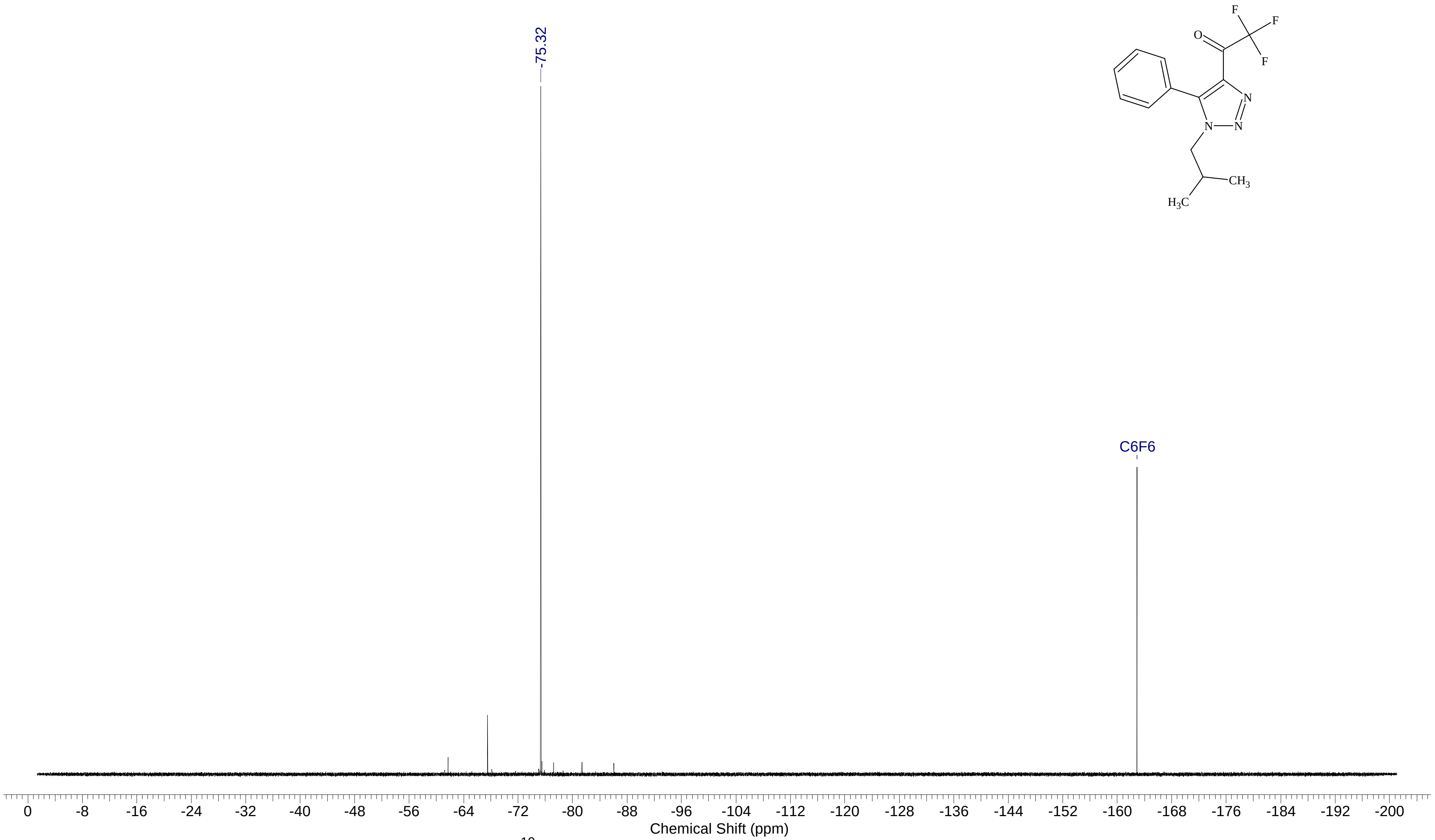

^{19}F NMR spectrum of **4k** (376.5 MHz, $CDCl_3$)

Acquisition Time (sec)	4.0894	***Comment***	Imported from UXNMR.	***Date***	07 Apr 2022 11:38:38
File Name	C:\DOCS\OUTPUT_301\2022\04.апрель\BM-2476-1.H_001001r			***Frequency (MHz)***	400.13
Nucleus	1H	***Number of Transients***	4 ***Original Points Count*** 32768	***Points Count***	131072
Pulse Sequence	zg30	***Solvent***	CHLOROFORM-D	***Sweep Width (Hz)***	8012.82
Temperature (degree C)	27.000				

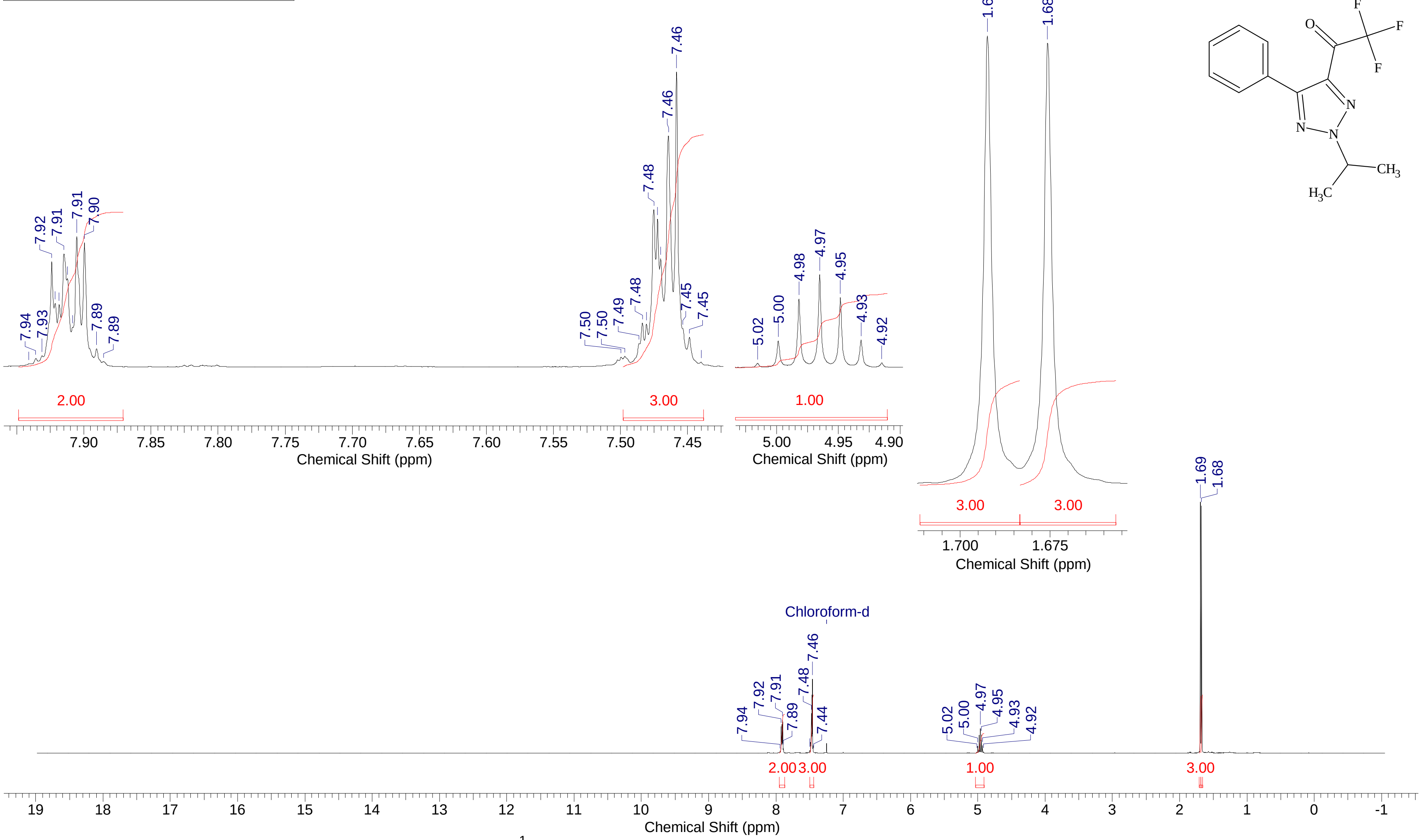

^{1}H NMR spectrum of **3l** (400.1 MHz, $CDCl_3$)

16 May 2022

Acquisition Time (sec)	1.7433	Comment	Imported from UXNMR.			Date	07 Apr 2022 12:13:14
File Name	C:\DOCS\OUTPUT_301\2022\04.апрель\BM-2476-1.F_005001r					Frequency (MHz)	376.50
Nucleus	19F	Number of Transients	16	Original Points Count	131072	Points Count	262144
Pulse Sequence	zgflqn	Solvent	CHLOROFORM-D			Sweep Width (Hz)	75187.97
Temperature (degree C)	27.000						

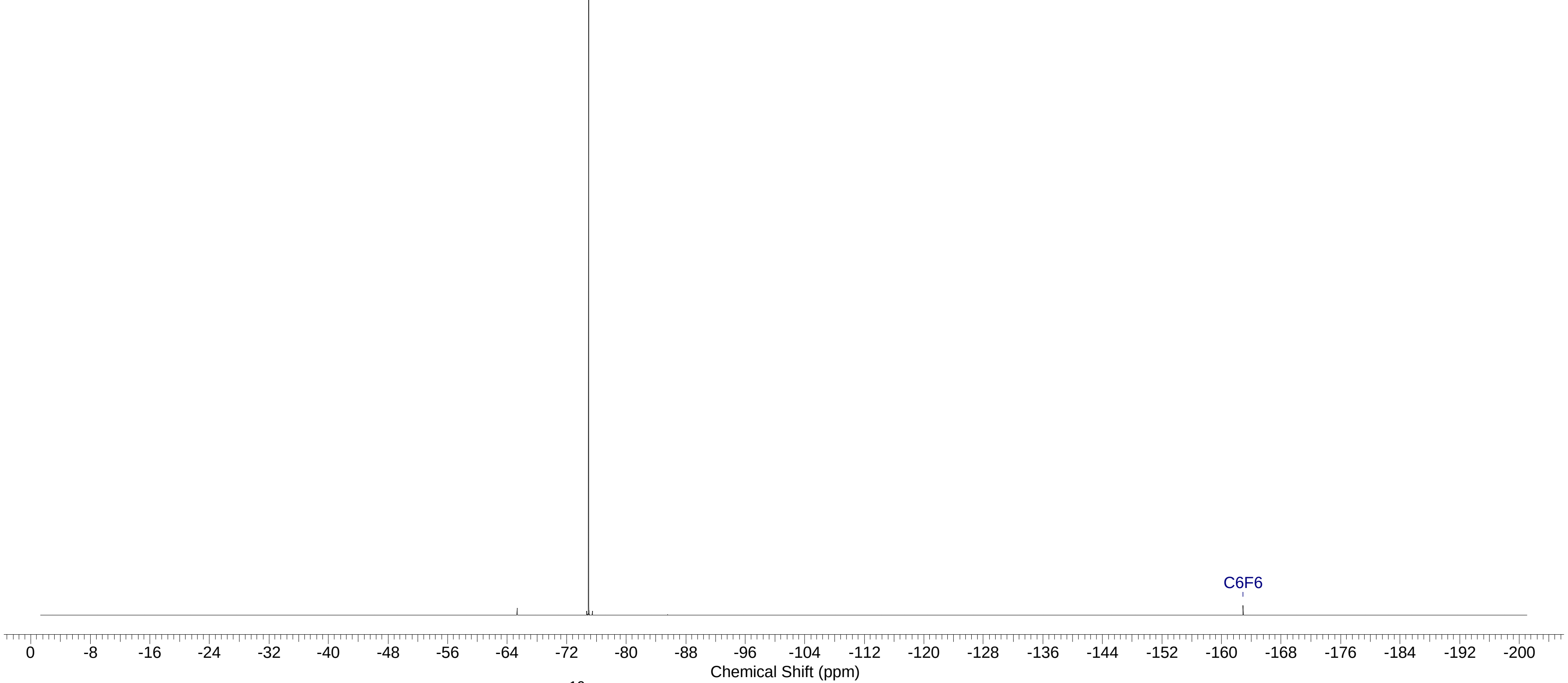

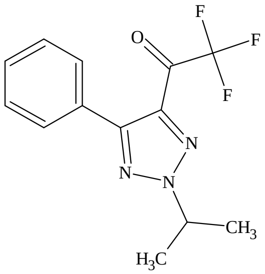

^{19}F NMR spectrum of **3l** (376.5 MHz, $CDCl_3$)

17 May 2022

Acquisition Time (sec)	0.6783	***Comment***	Imported from UXNMR.			***Date***	07 Apr 2022 11:54:10
File Name	C:\DOCS\OUTPUT_301\2022\04.апрель\BM-2476-1.C_002001r					***Frequency (MHz)***	100.61
Nucleus	13C	***Number of Transients***	361	***Original Points Count***	16384	***Points Count***	131072
Pulse Sequence	zgpg30	***Solvent***	CHLOROFORM-D			***Sweep Width (Hz)***	24154.59
Temperature (degree C)	27.000						

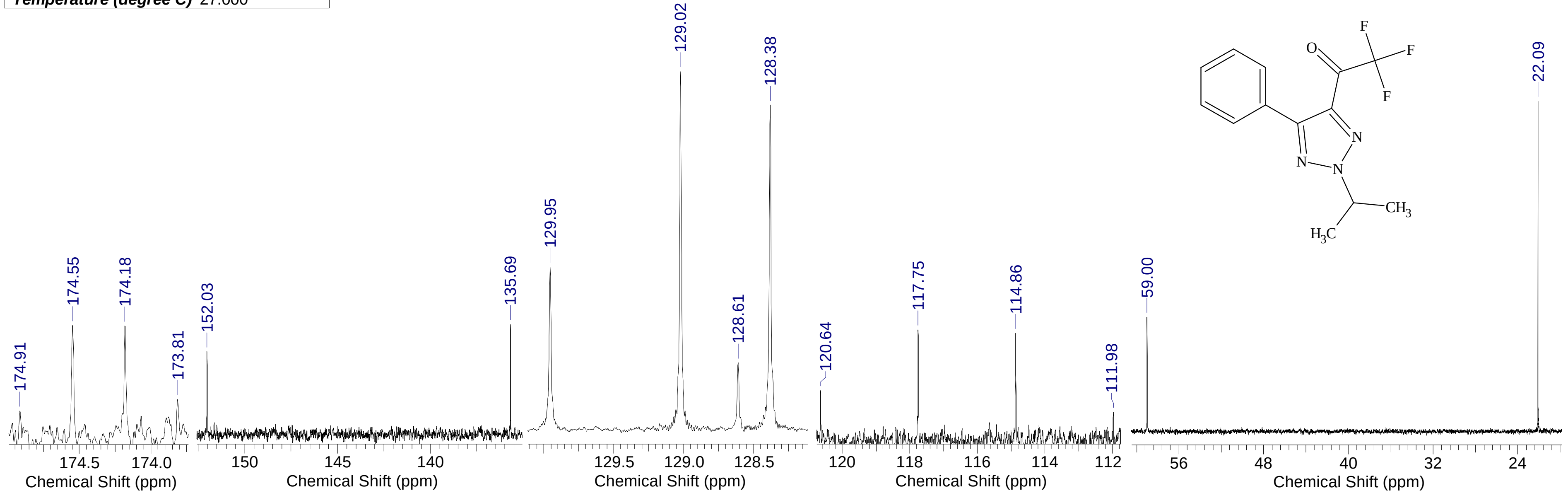

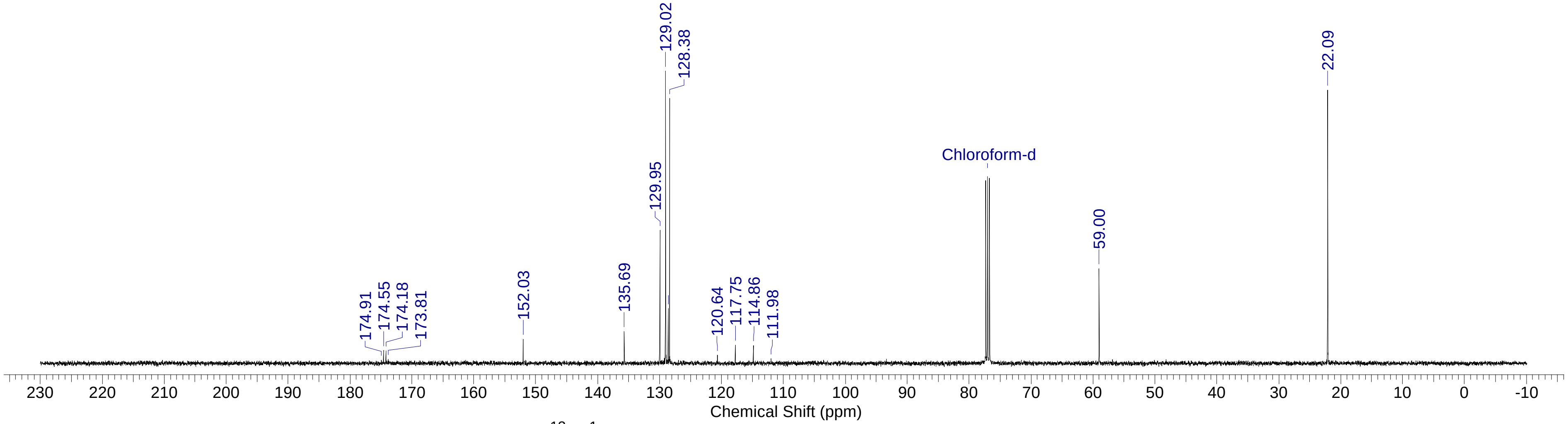

$^{13}C\{^1H\}$ NMR spectrum of **3l** (100.6 MHz, $CDCl_3$)

17 May 2022

Acquisition Time (sec)	4.0894	Comment	Imported from UXNMR.			Date	06 Apr 2022 17:08:52
File Name	C:\DOCS\OUTPUT_301\2022\04.апрель\BM-2476-2.H_001001r					Frequency (MHz)	400.13
Nucleus	1H	Number of Transients	4	Original Points Count	32768	Points Count	131072
Pulse Sequence	zg30	Solvent	CHLOROFORM-D			Sweep Width (Hz)	8012.82
Temperature (degree C)	27.000						

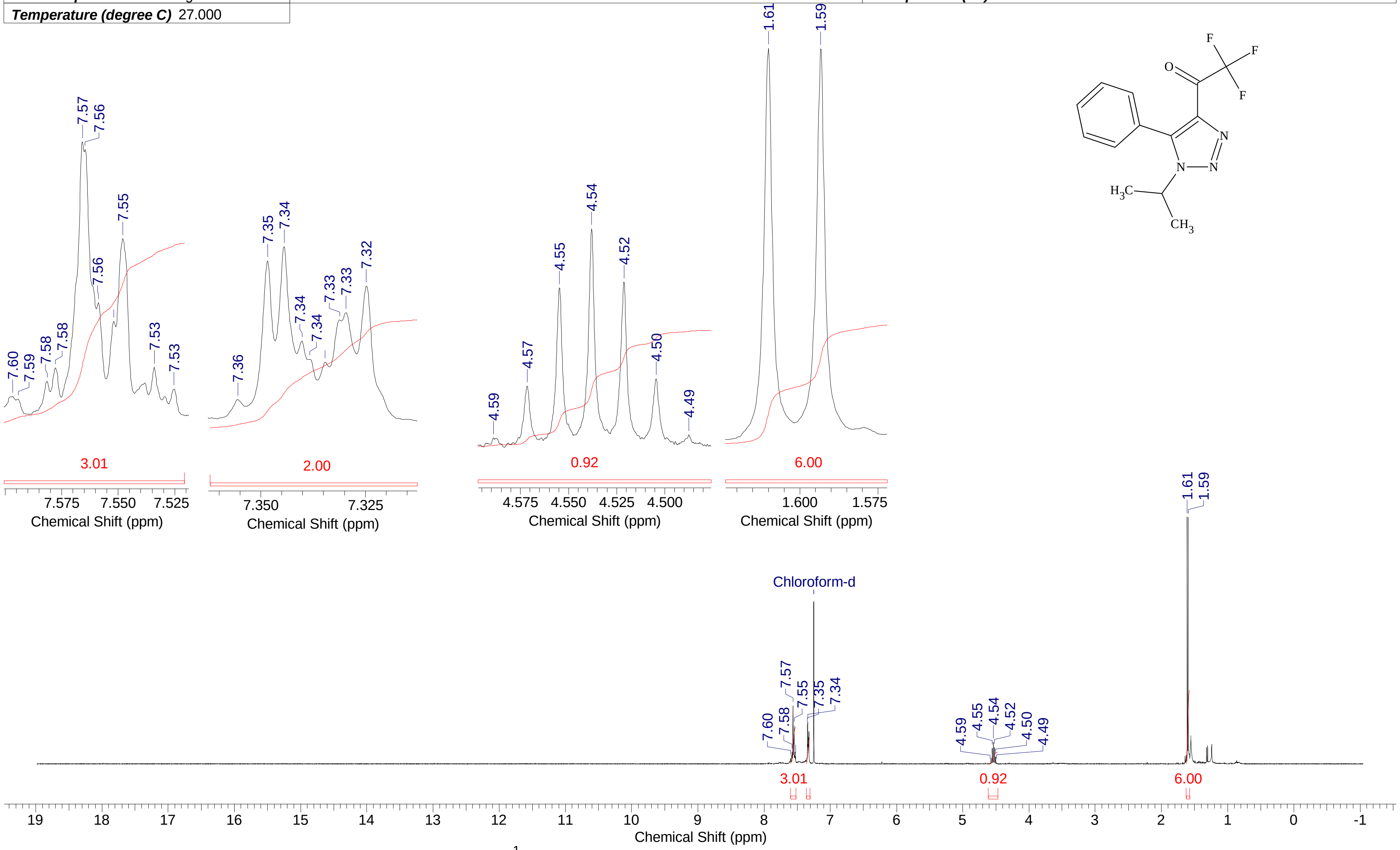

^{1}H NMR spectrum of **4l** (400.1 MHz, $CDCl_3$)

17 May 2022

Acquisition Time (sec)	1.7433	***Comment***	Imported from UXNMR.			***Date***	06 Apr 2022 17:40:20
File Name	C:\DOCS\OUTPUT_301\2022\04.апрель\BM-2476-2.F_005001r					***Frequency (MHz)***	376.50
Nucleus	19F	***Number of Transients***	16	***Original Points Count***	131072	***Points Count***	262144
Pulse Sequence	zgflqn	***Solvent***	CHLOROFORM-D			***Sweep Width (Hz)***	75187.97
Temperature (degree C)	27.000						

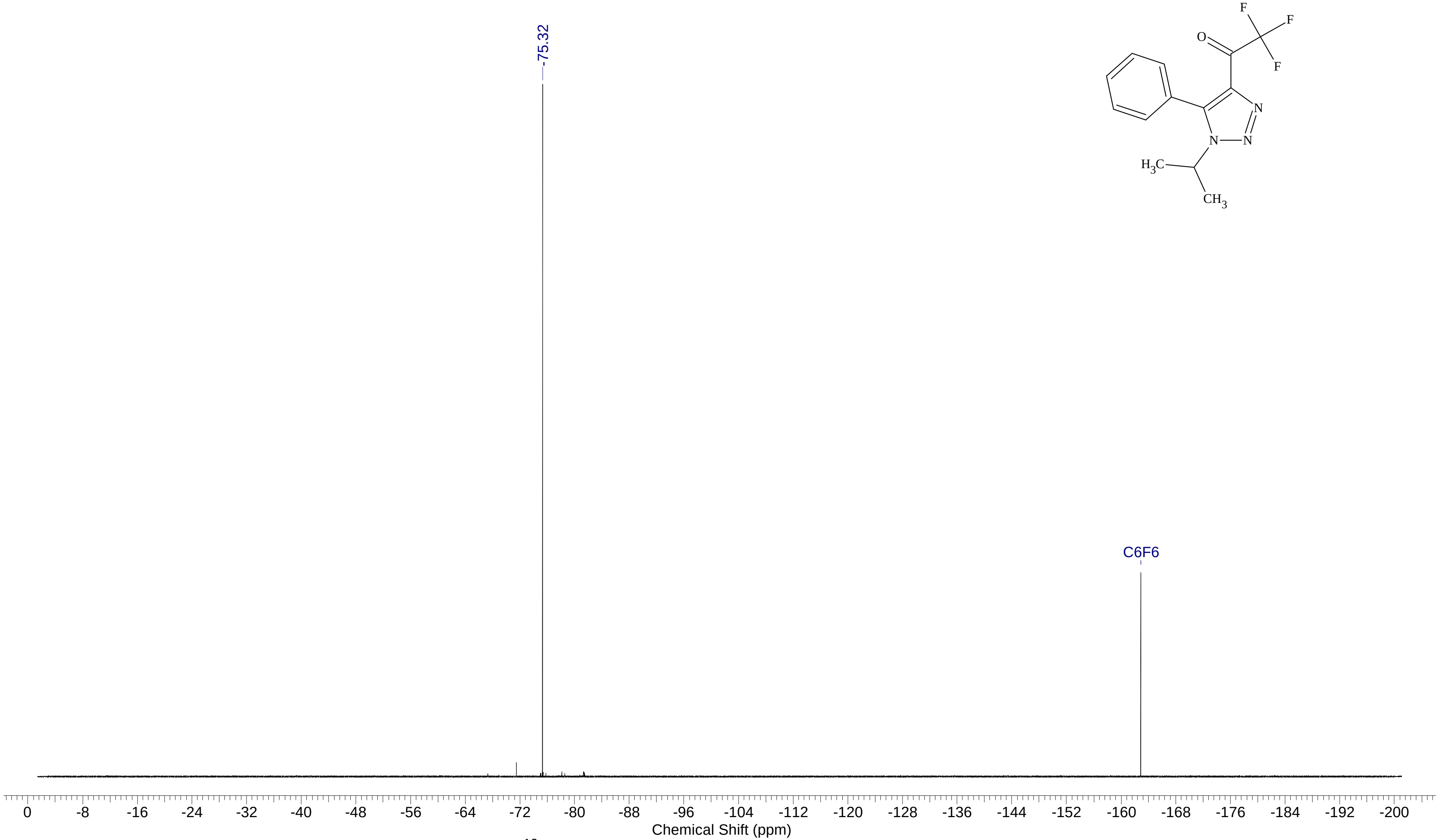

^{19}F NMR spectrum of **4l** (376.5 MHz, $CDCl_3$)

25 Nov 2022

**Acquisition Time (sec)**	4.0894	**Comment**	Imported from UXNMR.			**Date**	28 Jun 2022 14:29:08
**File Name**	C:\DOCS\OUTPUT_301\2022\06.июнь\SZA-BM-2538-4.H_001001r					**Frequency (MHz)**	400.13
**Nucleus**	1H	**Number of Transients**	4	**Original Points Count**	32768	**Points Count**	131072
**Pulse Sequence**	zg30	**Solvent**	CHLOROFORM-D			**Sweep Width (Hz)**	8012.82
**Temperature (degree C)**	27.000						

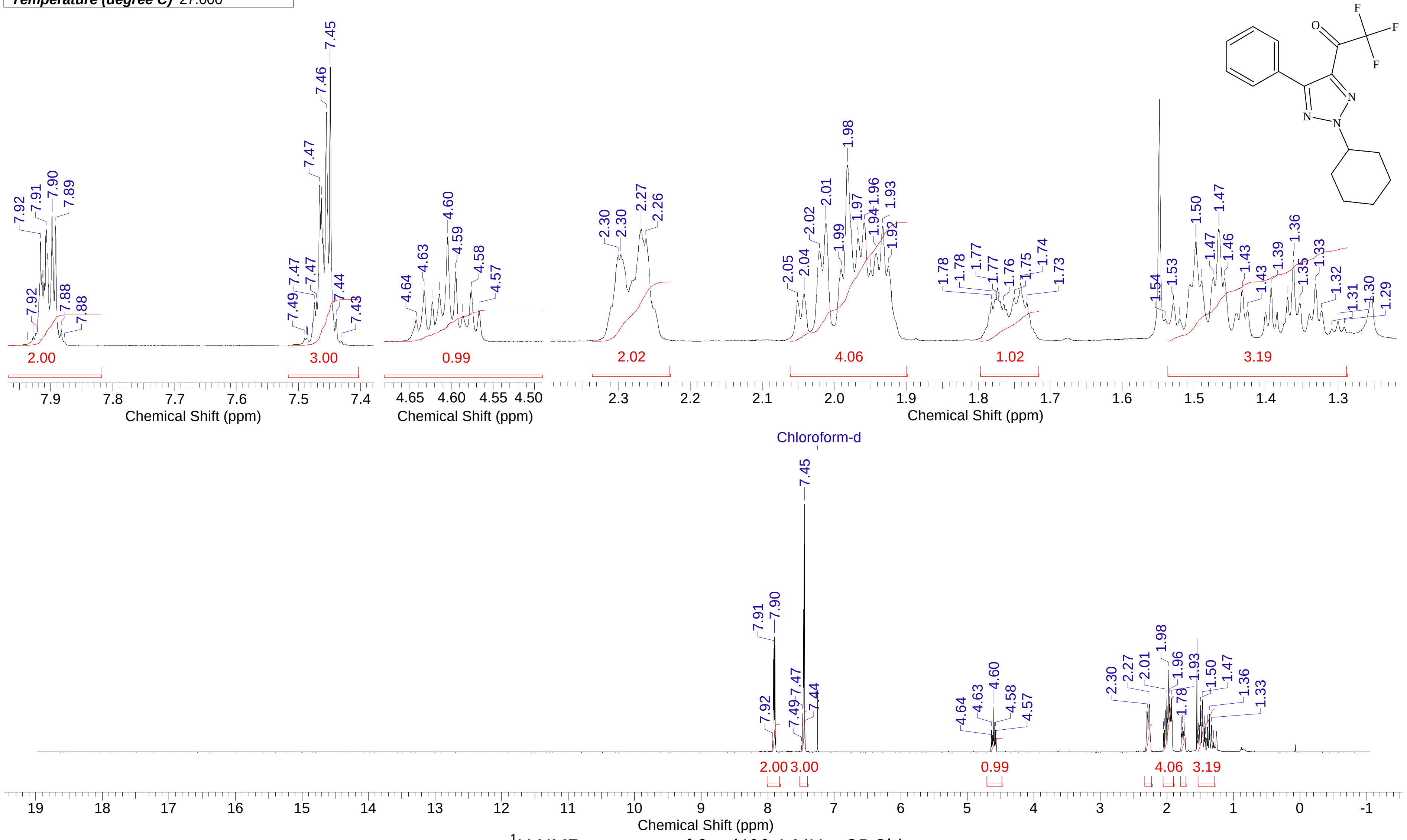

^{1}H NMR spectrum of **3m** (400.1 MHz, $CDCl_3$)

25 Nov 2022

**_Acquisition Time (sec)_**	1.7433	**_Comment_**	Imported from UXNMR.			**_Date_**	28 Jun 2022 14:47:14
**_File Name_**	C:\DOCS\OUTPUT_301\2022\06.июнь\SZA-BM-2538-4.F_005001r					**_Frequency (MHz)_**	376.50
**_Nucleus_**	19F	**_Number of Transients_**	16	**_Original Points Count_**	131072	**_Points Count_**	262144
**_Pulse Sequence_**	zgflqn	**_Solvent_**	CHLOROFORM-D			**_Sweep Width (Hz)_**	75187.97
**_Temperature (degree C)_**	27.000						

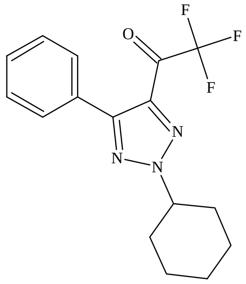

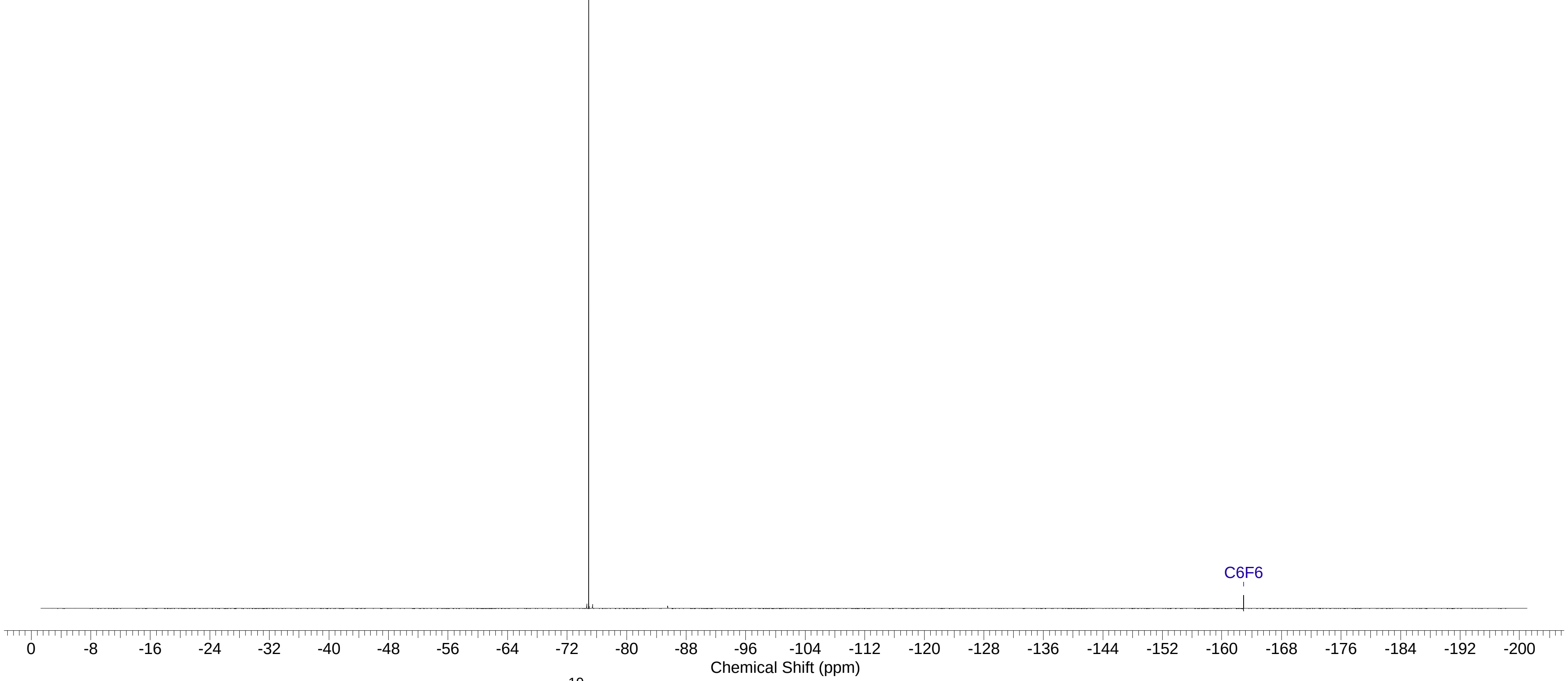

^{19}F NMR spectrum of **3m** (376.5 MHz, $CDCl_3$)

25 Nov 2022

Acquisition Time (sec)	0.6783	***Comment***	Imported from UXNMR.			***Date***	29 Jun 2022 17:47:08
File Name	C:\DOCS\OUTPUT_301\2022\06.июнь\SZA-BM-2538-4.C_002001r					***Frequency (MHz)***	100.61
Nucleus	13C	***Number of Transients***	401	***Original Points Count***	16384	***Points Count***	131072
Pulse Sequence	zgpg30	***Solvent***	CHLOROFORM-D			***Sweep Width (Hz)***	24154.59
Temperature (degree C)	27.000						

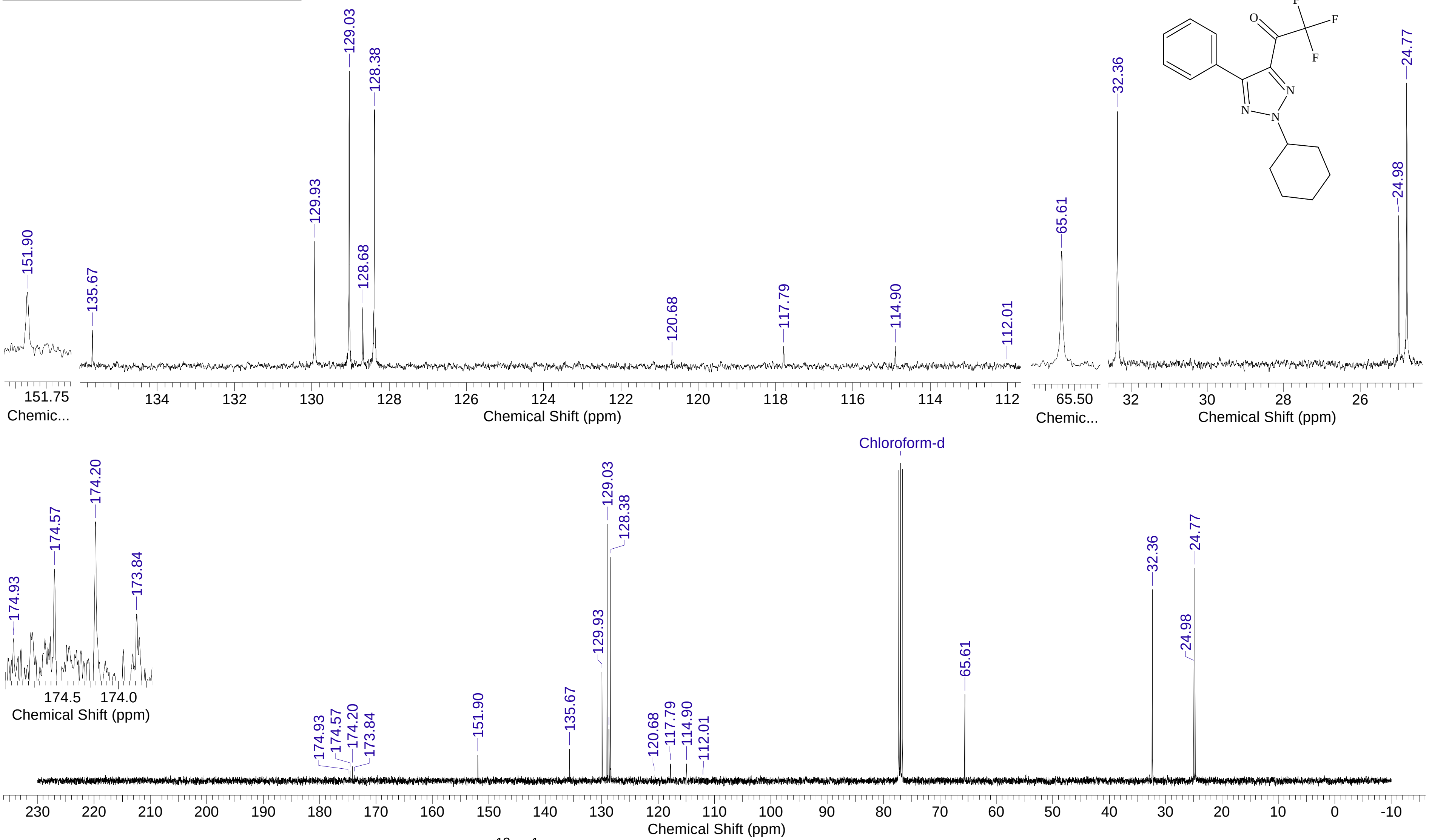

$^{13}C\{^1H\}$ NMR spectrum of **3m** (100.6 MHz, $CDCl_3$)

25 Nov 2022

Acquisition Time (sec)	4.0894	***Comment***	Imported from UXNMR.			***Date***	28 Jun 2022 14:24:24
File Name	C:\DOCS\OUTPUT_301\2022\06.июнь\SZA-BM-2538-15.H_001001r					***Frequency (MHz)***	400.13
Nucleus	1H	***Number of Transients***	4	***Original Points Count***	32768	***Points Count***	131072
Pulse Sequence	zg30	***Solvent***	CHLOROFORM-D			***Sweep Width (Hz)***	8012.82
Temperature (degree C)	27.000						

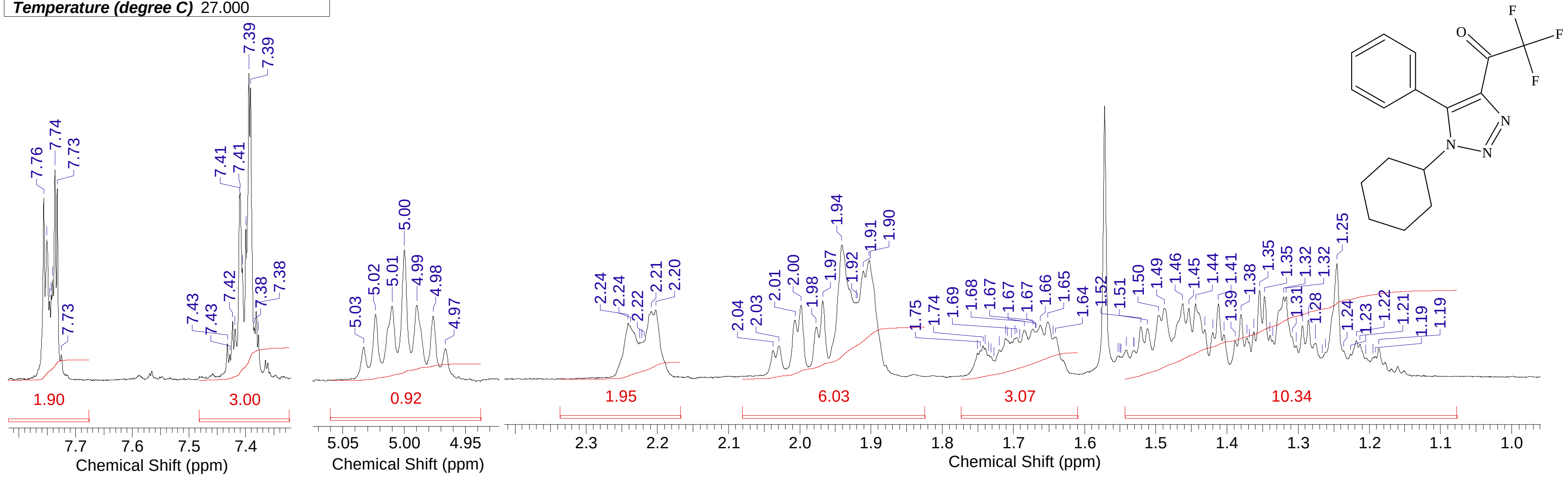

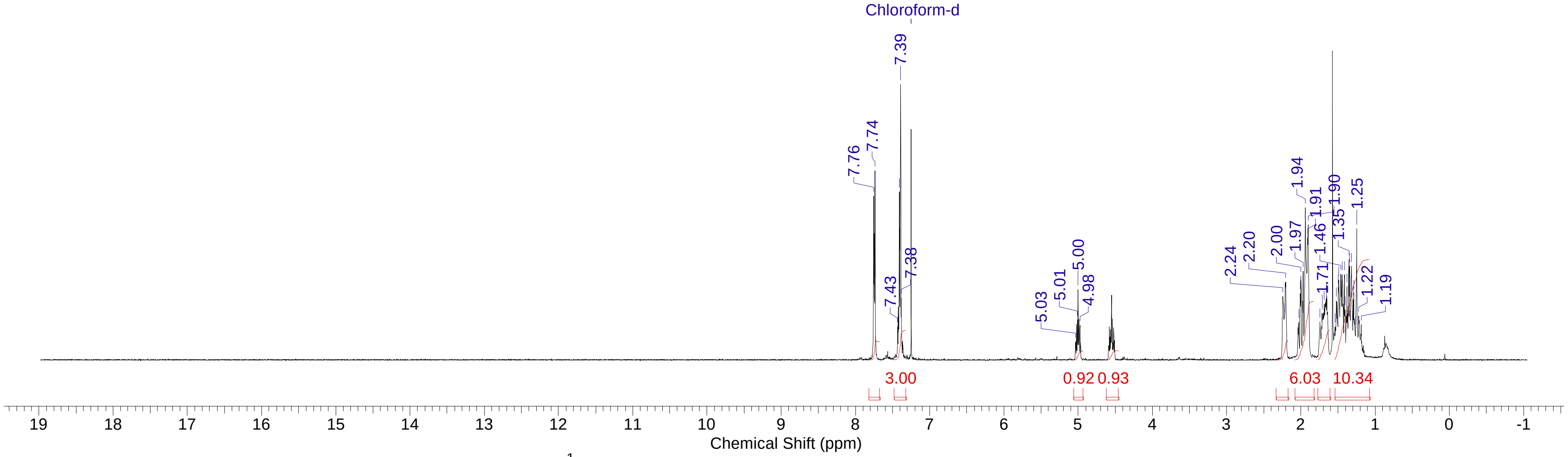

^{1}H NMR spectrum of **4m** (400.1 MHz, $CDCl_3$)

25 Nov 2022

Acquisition Time (sec)	1.7433	***Comment***	Imported from UXNMR.			***Date***	28 Jun 2022 14:44:30
File Name	C:\DOCS\OUTPUT_301\2022\06.июнь\SZA-BM-2538-15.F_005001r					***Frequency (MHz)***	376.50
Nucleus	19F	***Number of Transients***	32	***Original Points Count***	131072	***Points Count***	262144
Pulse Sequence	zgflqn	***Solvent***	CHLOROFORM-D			***Sweep Width (Hz)***	75187.97
Temperature (degree C)	27.000						

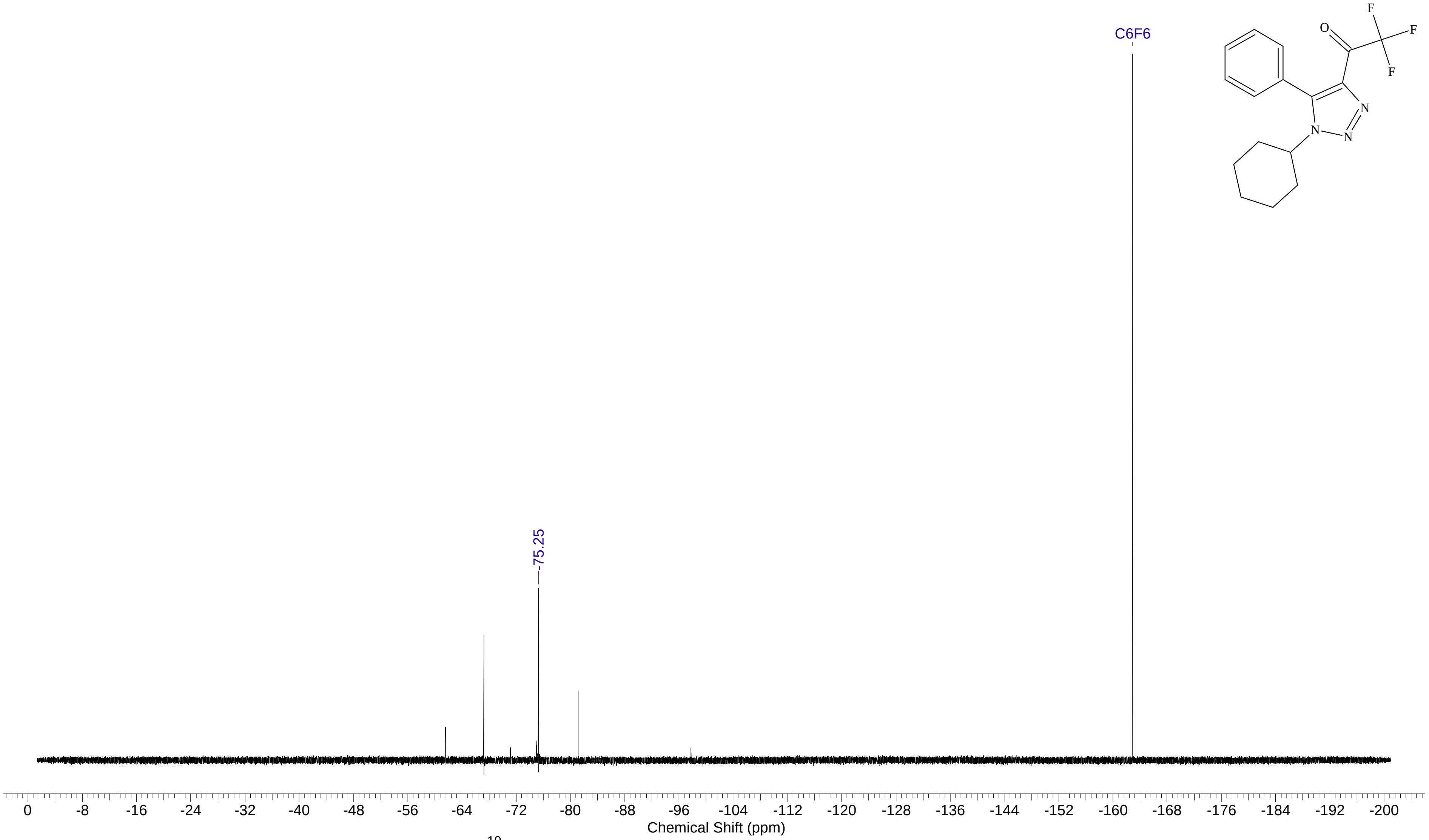

^{19}F NMR spectrum of crude **4m** (376.5 MHz, $CDCl_3$)

19 May 2022

Acquisition Time (sec)	4.0894	Comment	Imported from UXNMR.			Date	26 Apr 2022 14:01:10
File Name	C:\DOCS\OUTPUT_301\2022\04.апрель\SZA-BM-2504-R-18.H_001001r					Frequency (MHz)	400.13
Nucleus	1H	Number of Transients	7	Original Points Count	32768	Points Count	131072
Pulse Sequence	zg30	Solvent	BENZENE-D6	Sweep Width (Hz)	8012.82	Temperature (degree C)	27.000

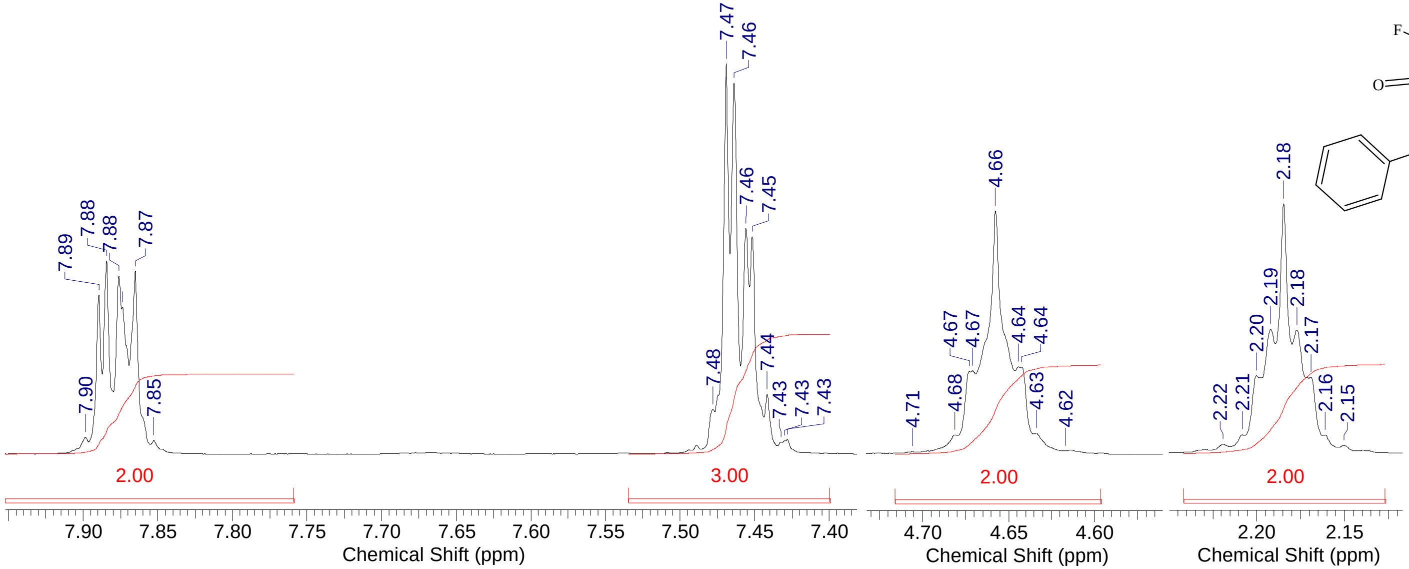

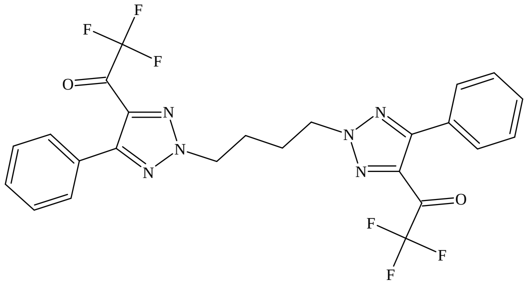

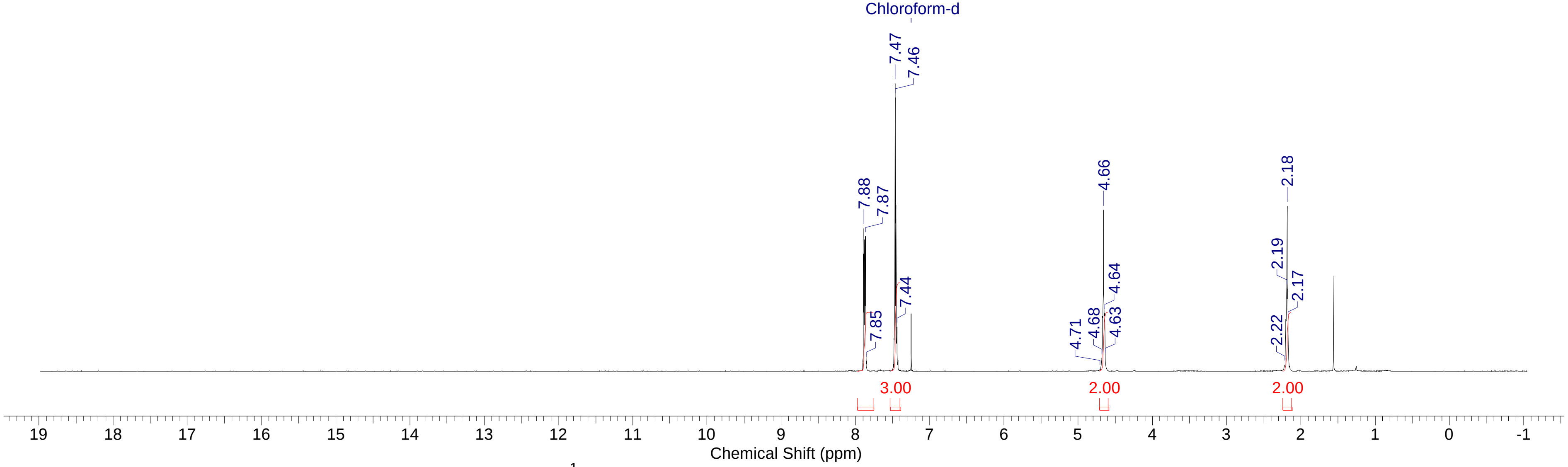

^{1}H NMR spectrum of **3n** (400.1 MHz, $CDCl_3$)

19 May 2022

Acquisition Time (sec)	1.7433	***Comment***	Imported from UXNMR.			***Date***	26 Apr 2022 13:58:04
File Name	C:\DOCS\OUTPUT_301\2022\04.апрель\SZA-BM-2504-R-18.F_005001r					***Frequency (MHz)***	376.50
Nucleus	19F	***Number of Transients***	16	***Original Points Count***	131072	***Points Count***	262144
Pulse Sequence	zgflqn	***Solvent***	CHLOROFORM-D			***Sweep Width (Hz)***	75187.97
Temperature (degree C)	27.000						

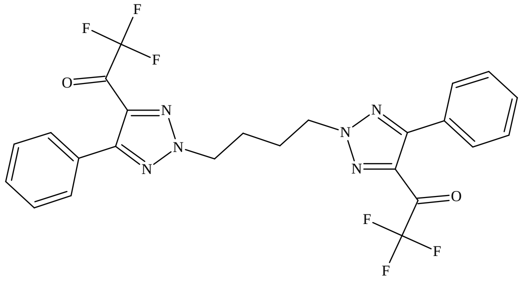

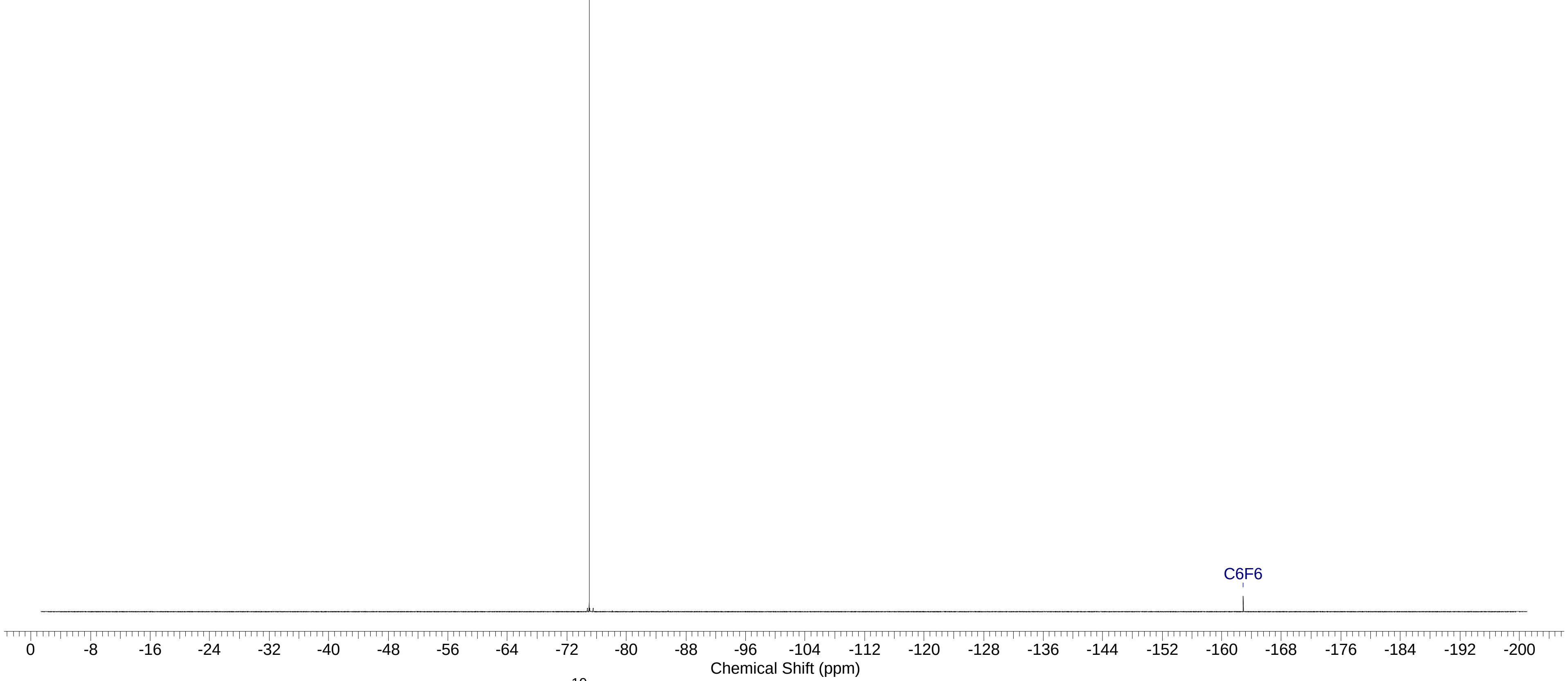

^{19}F NMR spectrum of **3n** (376.5 MHz, $CDCl_3$)

19 May 2022

Acquisition Time (sec)	0.6783	Comment	Imported from UXNMR.			Date	26 Apr 2022 14:26:04
File Name	C:\DOCS\OUTPUT_301\2022\04.апрель\SZA-BM-2504-R-18.C_002001r					Frequency (MHz)	100.61
Nucleus	13C	Number of Transients	546	Original Points Count	16384	Points Count	131072
Pulse Sequence	zgpg30	Solvent	CHLOROFORM-D			Sweep Width (Hz)	24154.59
Temperature (degree C)	27.000						

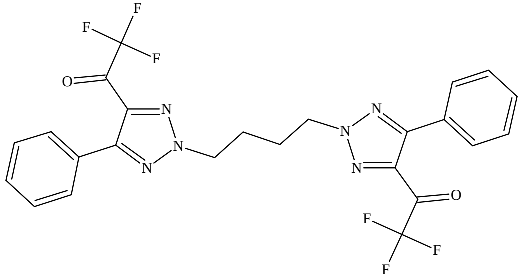

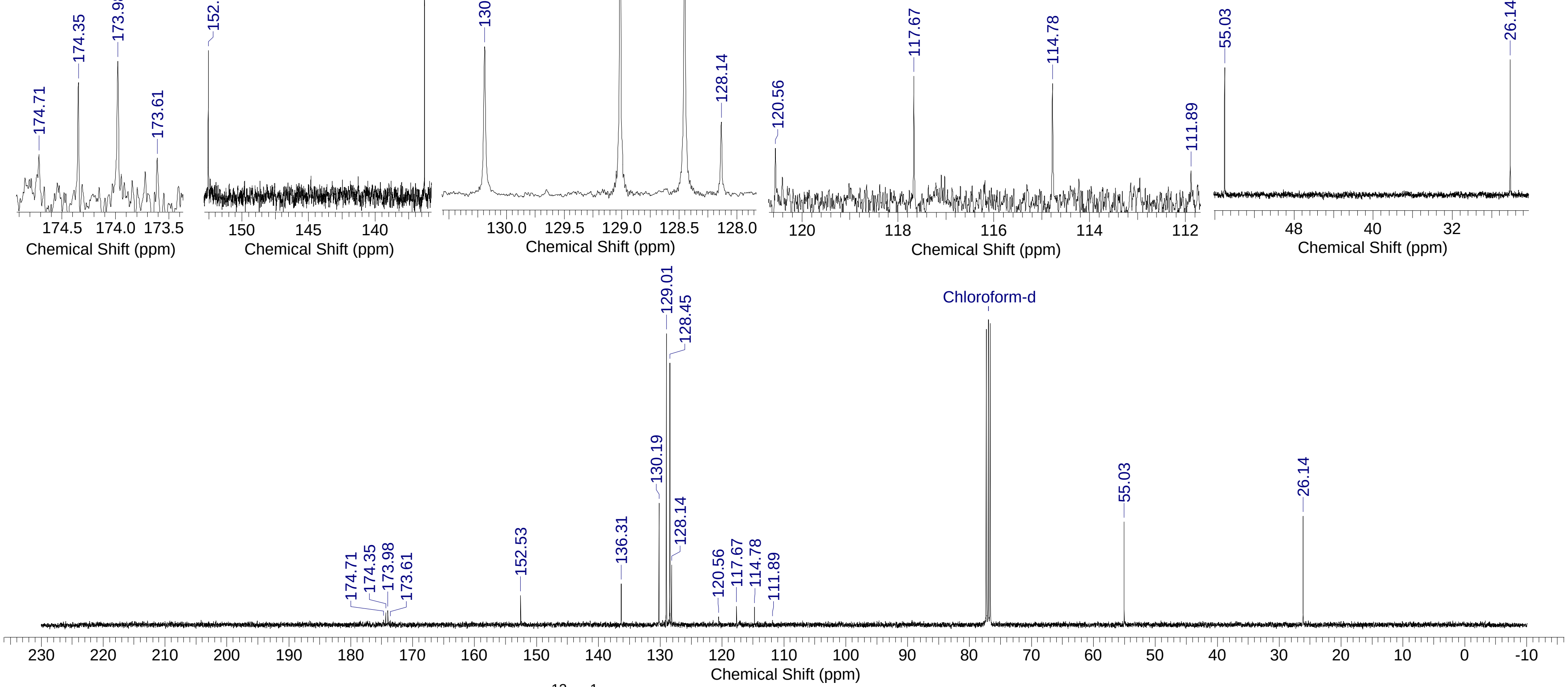

$^{13}C\{^{1}H\}$ NMR spectrum of **3n** (100.6 MHz, $CDCl_3$)

20 May 2022

Acquisition Time (sec)	4.0894	***Comment***	Imported from UXNMR.			***Date***	28 Apr 2022 11:23:52
File Name	C:\DOCS\OUTPUT_301\2022\04.апрель\SZA-BM-2504-25.H_001001r					***Frequency (MHz)***	400.13
Nucleus	1H	***Number of Transients***	8	***Original Points Count***	32768	***Points Count***	131072
Pulse Sequence	zg30	***Solvent***	CHLOROFORM-D			***Sweep Width (Hz)***	8012.82
Temperature (degree C)	27.000						

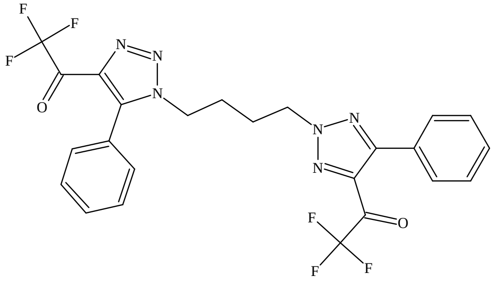

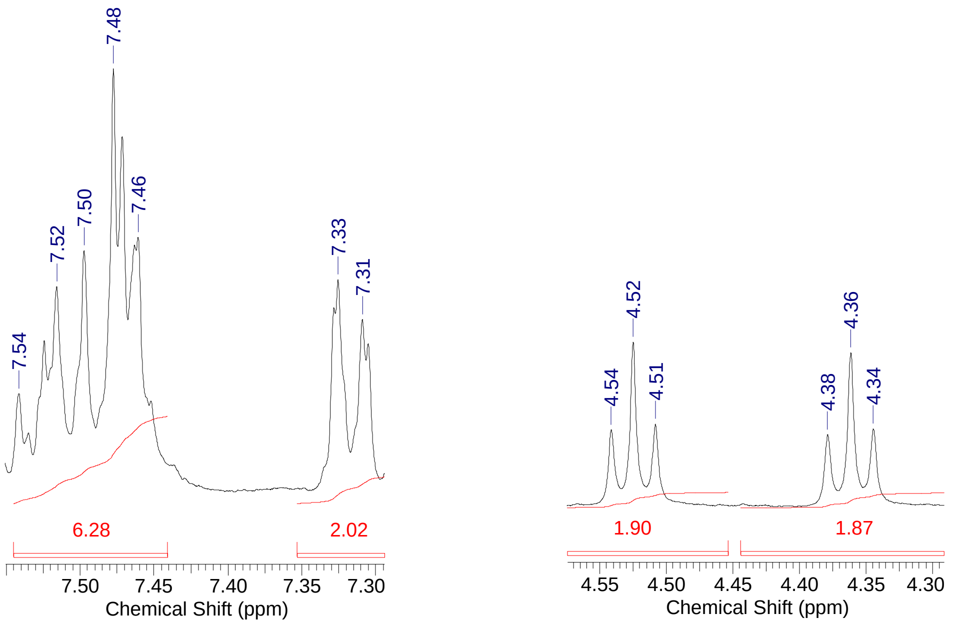

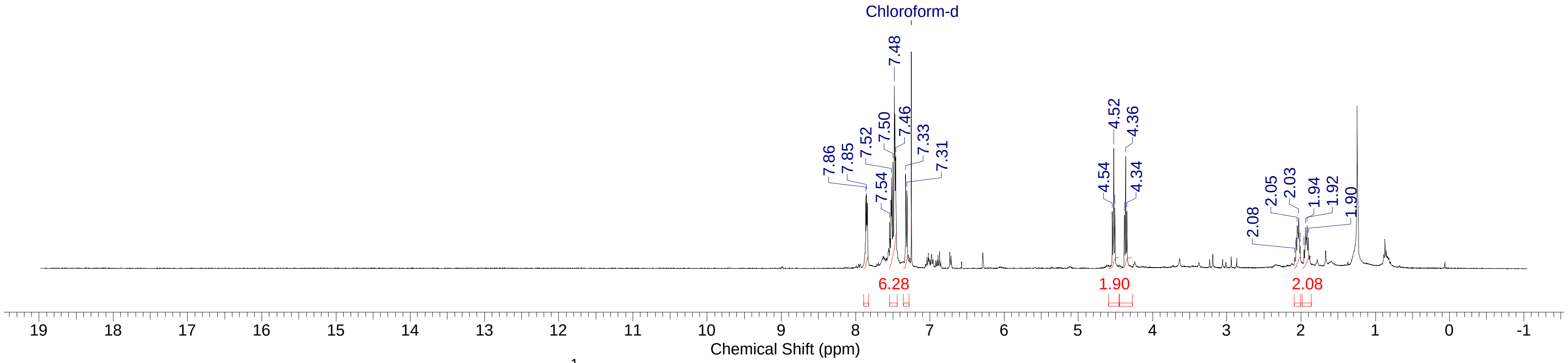

^{1}H NMR spectrum of **4n** (400.1 MHz, $CDCl_3$)

20 May 2022

Acquisition Time (sec)	1.7433	***Comment***	Imported from UXNMR.			***Date***	28 Apr 2022 12:03:50
File Name	C:\DOCS\OUTPUT_301\2022\04.апрель\SZA-BM-2504-25.F_005001r					***Frequency (MHz)***	376.50
Nucleus	19F	***Number of Transients***	16	***Original Points Count***	131072	***Points Count***	262144
Pulse Sequence	zgflqn	***Solvent***	CHLOROFORM-D			***Sweep Width (Hz)***	75187.97
Temperature (degree C)	27.000						

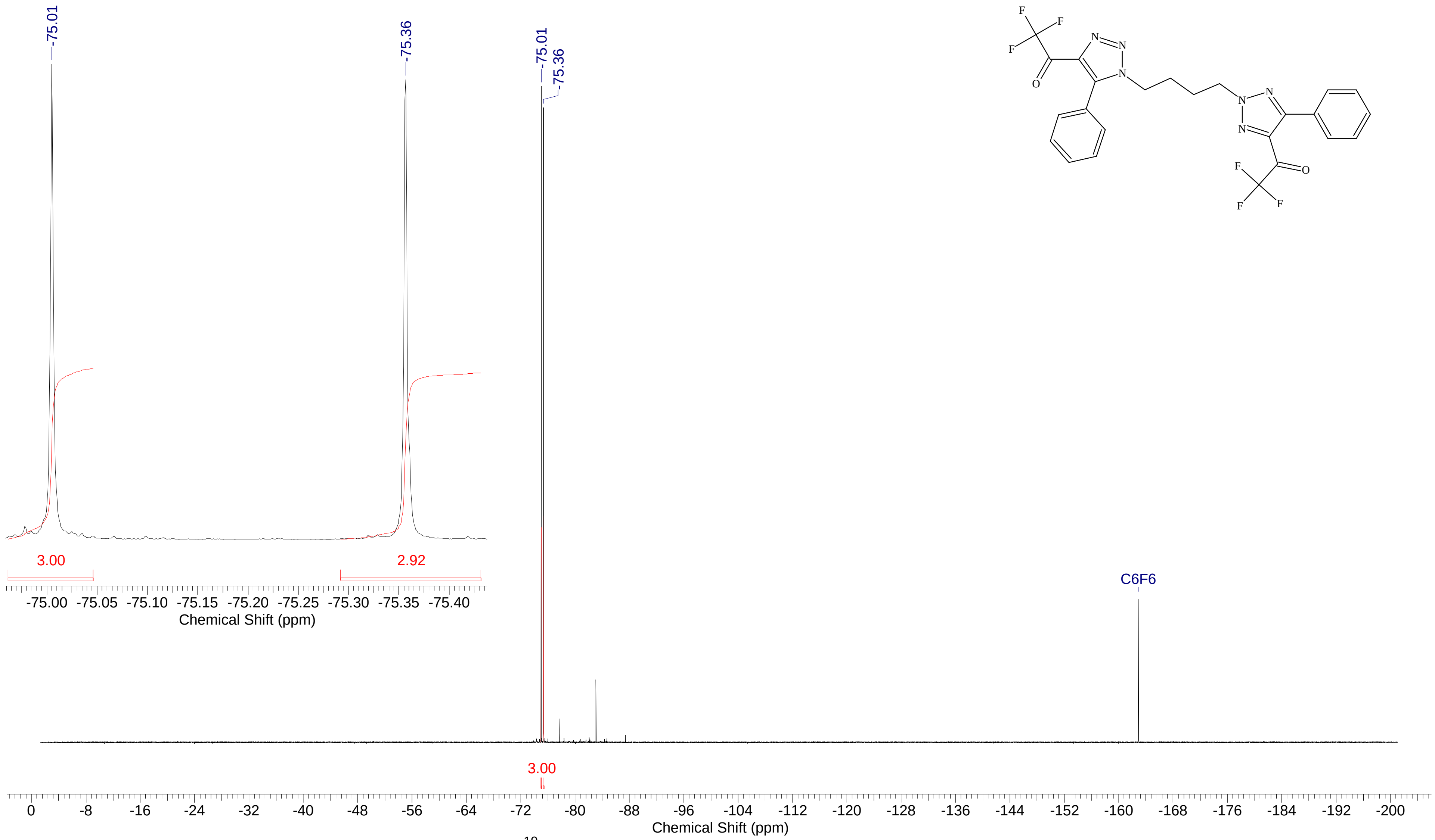

^{19}F NMR spectrum of **4n** (376.5 MHz, $CDCl_3$)

28 Mar 2023

Acquisition Time (sec)	0.6783	Comment	Imported from UXNMR.			Date	29 Apr 2022 12:48:00
File Name	C:\DOCS\OUTPUT_301\2022\05.май\SZA-BM-2504-25.C\SZA-BM-2504-25.C_002000fid					Frequency (MHz)	100.62
Nucleus	13C	Number of Transients	3207	Original Points Count	16384	Points Count	16384
Pulse Sequence	zgpg30	Solvent	CHLOROFORM-D	Sweep Width (Hz)	24154.59	Temperature (degree C)	27.000

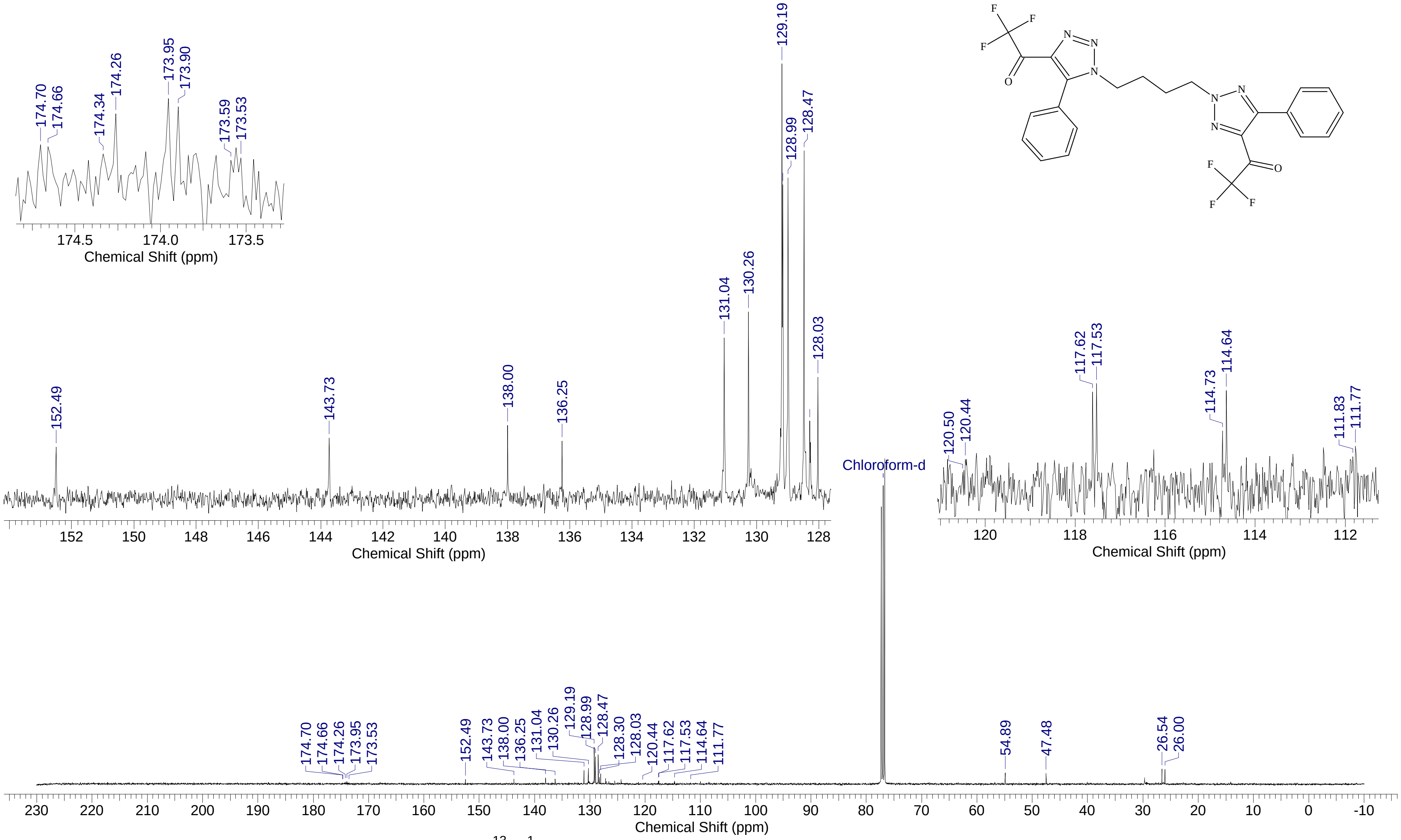

$^{13}C\{^{1}H\}$ NMR spectrum of **4n** (100.6 MHz, $CDCl_3$)

17 May 2022

Acquisition Time (sec)	4.0894	***Comment***	Imported from UXNMR.			***Date***	19 Mar 2022 23:08:00
File Name	C:\DOCS\BM\ЯМР_СОСЕДИ\2022\bm220319\BM-2459-R_001001r					***Frequency (MHz)***	400.13
Nucleus	1H	***Number of Transients***	8	***Original Points Count***	32768	***Points Count***	131072
Pulse Sequence	zg30	***Solvent***	CHLOROFORM-D			***Sweep Width (Hz)***	8012.82
Temperature (degree C)	27.000						

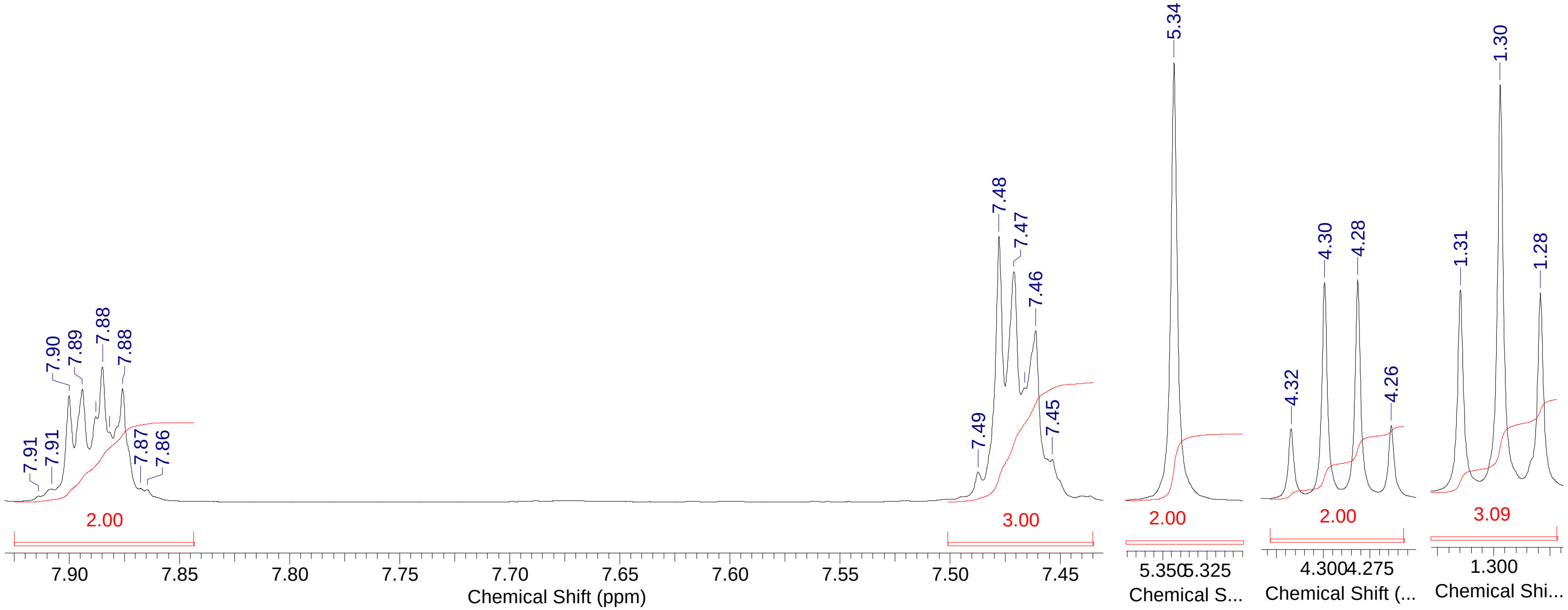

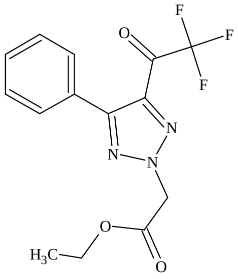

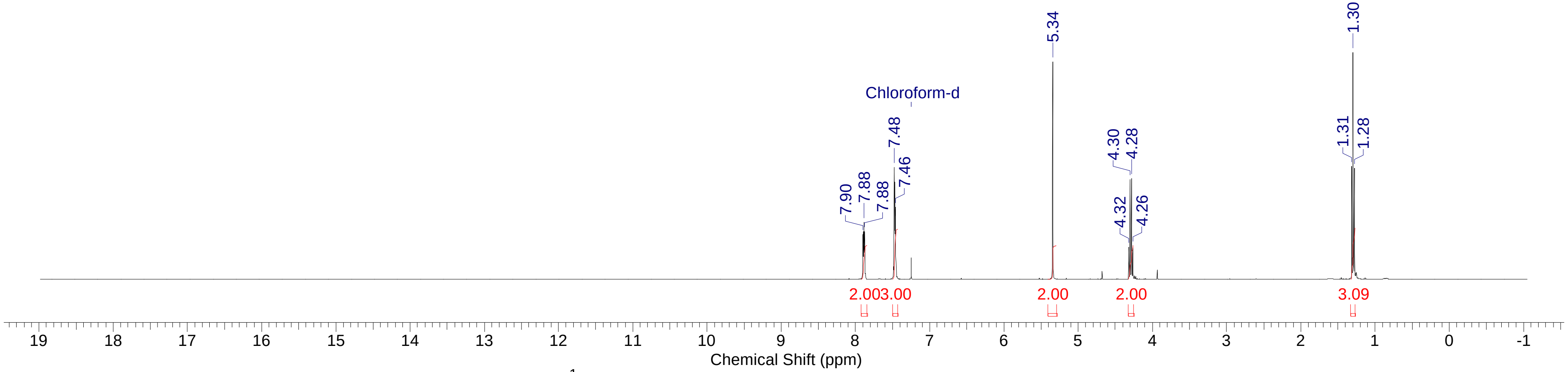

^{1}H NMR spectrum of **3o** (400.1 MHz, $CDCl_3$)

17 May 2022

Acquisition Time (sec)	1.7433	***Comment***	Imported from UXNMR.			***Date***	19 Mar 2022 23:11:04
File Name	C:\BM_DATA\DOCS\ЯМР_соседи\2022\bm220319\BM-2459-R_005001r					***Frequency (MHz)***	376.50
Nucleus	19F	***Number of Transients***	16	***Original Points Count***	131072	***Points Count***	262144
Pulse Sequence	zgflqn	***Solvent***	CHLOROFORM-D			***Sweep Width (Hz)***	75187.97
Temperature (degree C)	27.000						

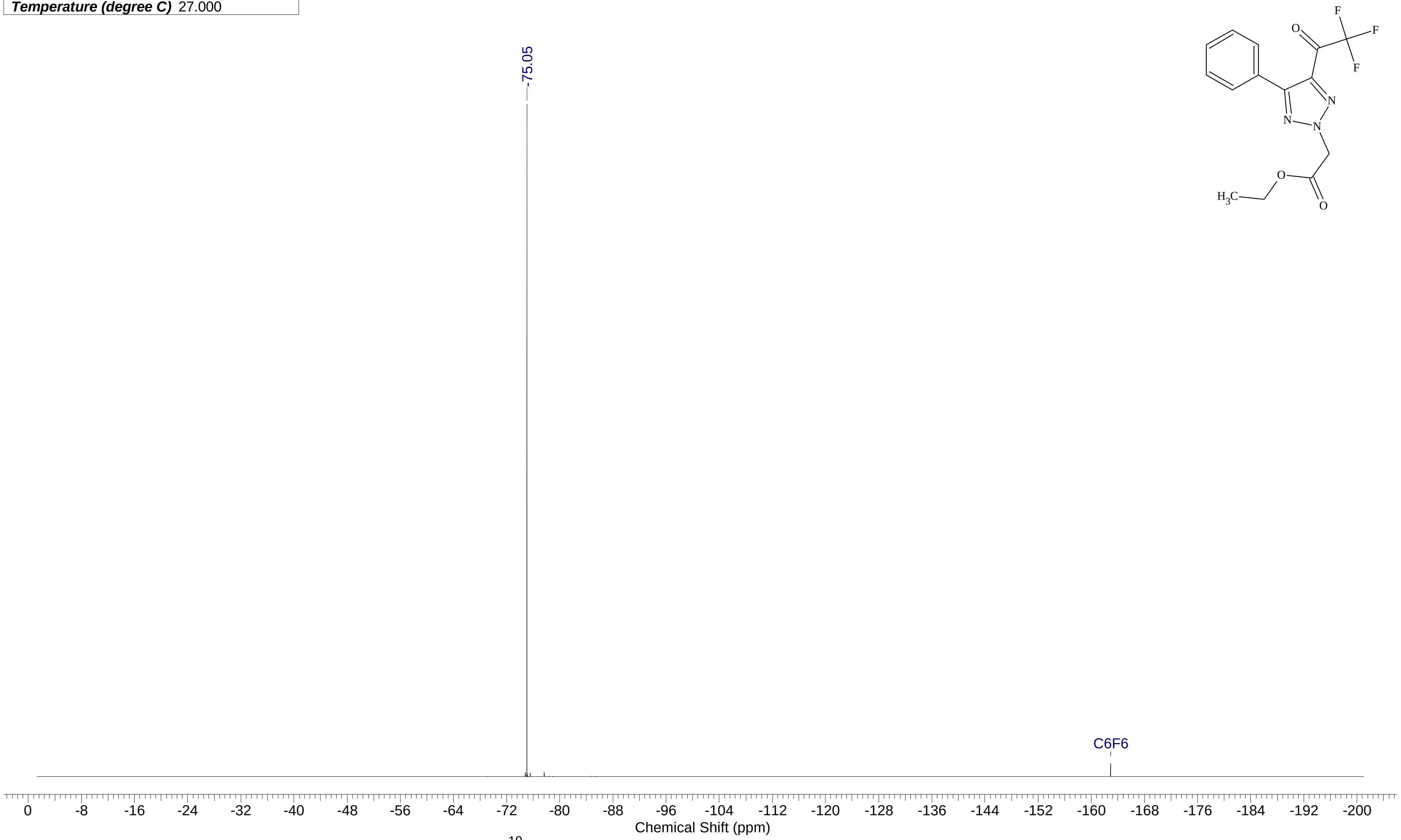

^{19}F NMR spectrum of **3o** (376.5 MHz, $CDCl_3$)

Acquisition Time (sec)	0.6783	***Comment***	Imported from UXNMR.			***Date***	21 Mar 2022 15:51:54
File Name	C:\DOCS\OUTPUT_301\2022\03.март\BM-2459-R.C_002001r					***Frequency (MHz)***	100.61
Nucleus	13C	***Number of Transients***	257	***Original Points Count***	16384	***Points Count***	131072
Pulse Sequence	zgpg30	***Solvent***	DMSO-D6	***Sweep Width (Hz)***	24154.59	***Temperature (degree C)***	27.000

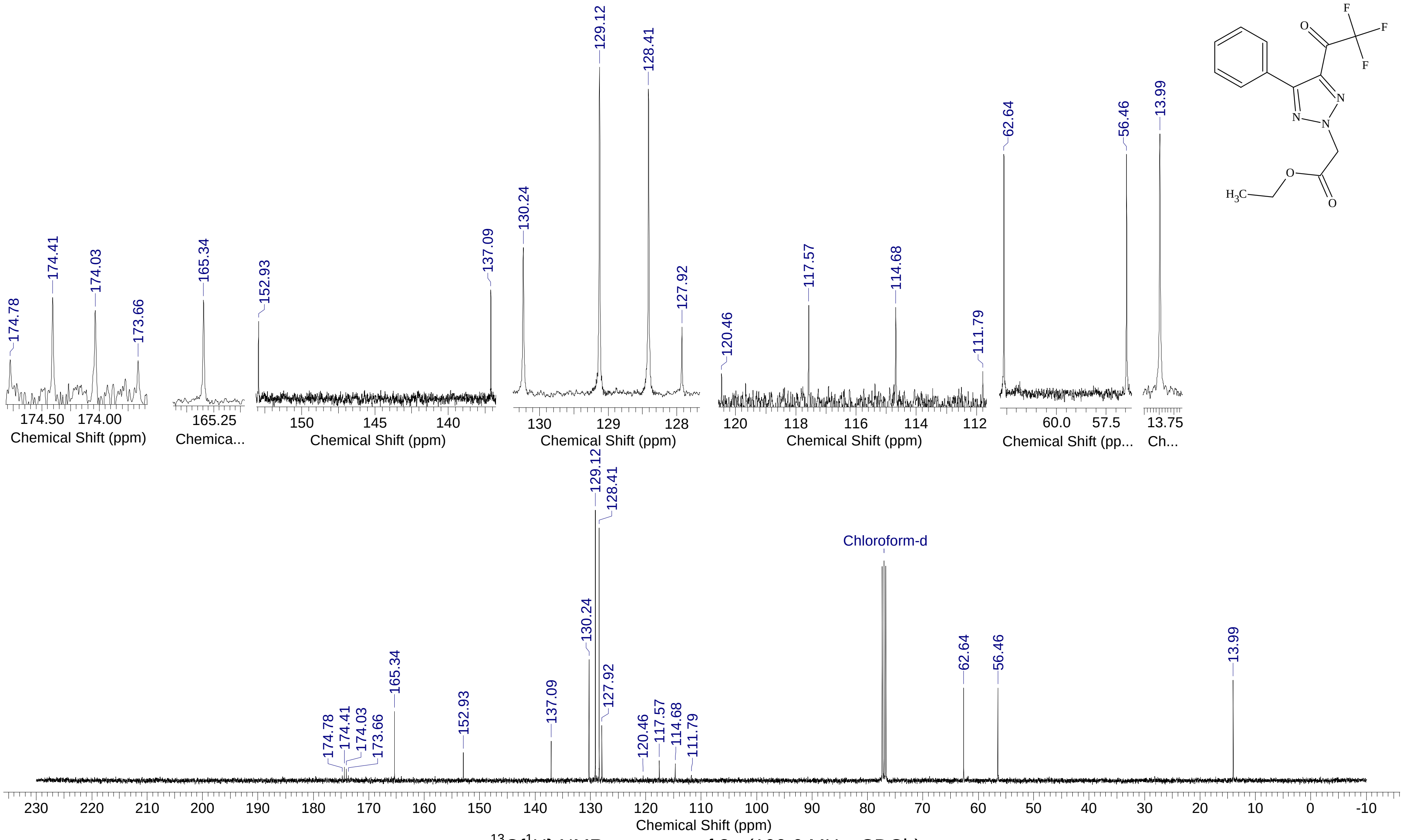

$^{13}C\{^{1}H\}$ NMR spectrum of **3o** (100.6 MHz, $CDCl_3$)

28 Mar 2023

**Acquisition Time (sec)**	4.0894	**Comment**	Imported from UXNMR.			**Date**	17 Mar 2022 22:53:52
**File Name**	C:\DOCS\BM\ЯМР_СОСЕДИ\2022\BM-2459-R\BM-2459-R_001001r					**Frequency (MHz)**	400.13
**Nucleus**	1H	**Number of Transients**	8	**Original Points Count**	32768	**Points Count**	131072
**Pulse Sequence**	zg30	**Solvent**	CHLOROFORM-D			**Sweep Width (Hz)**	8012.82
**Temperature (degree C)**	27.000						

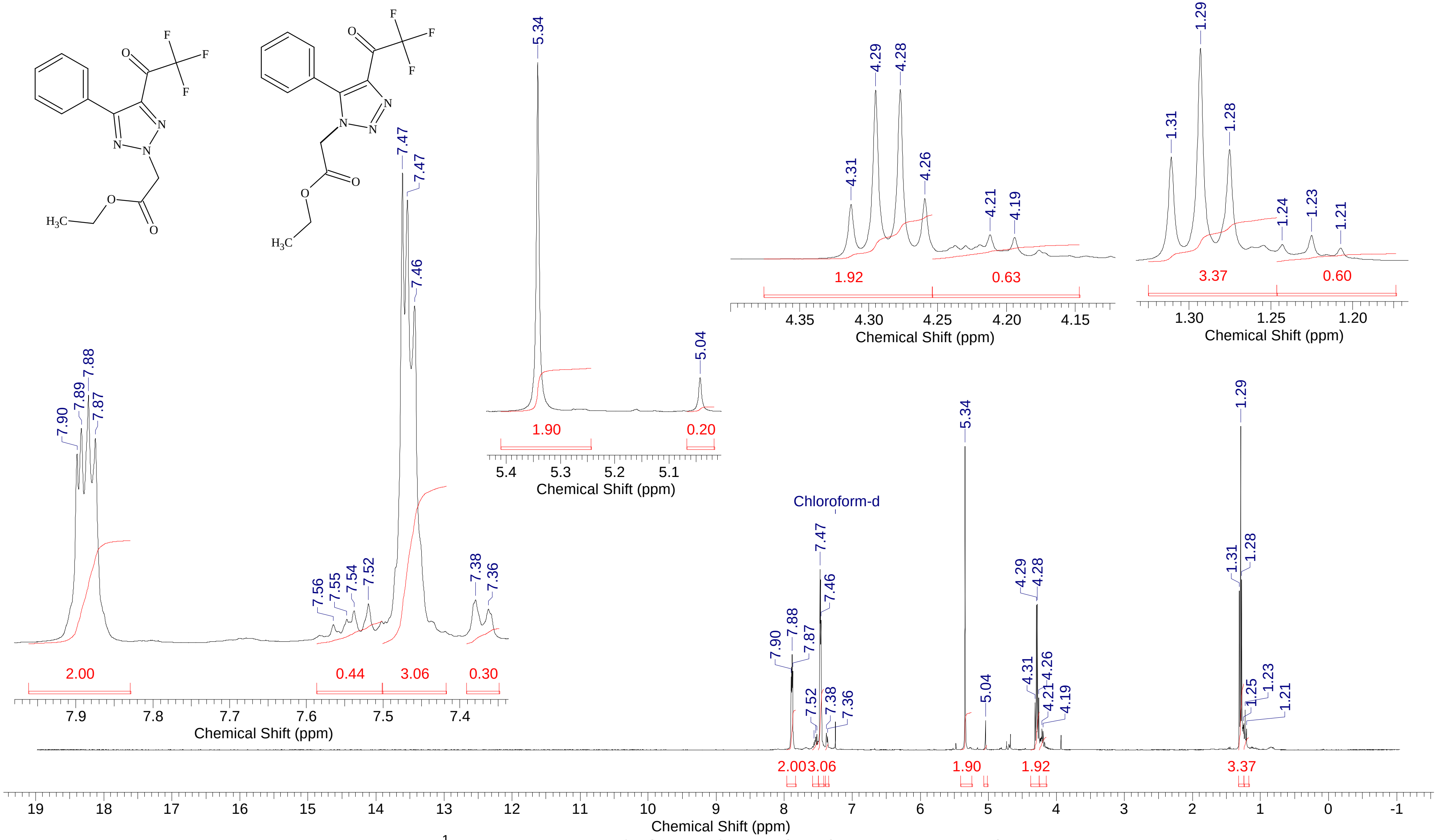

^{1}H NMR spectrum of mixture of **3o** and **4o** (400.1 MHz, $CDCl_3$)

28 Mar 2023

Acquisition Time (sec)	1.7433	***Comment***	Imported from UXNMR.	***Date***	23 Mar 2022 17:55:00
File Name	C:\DOCS\OUTPUT_301\2022\03.март\SZA-BM-2454.F_005001r			***Frequency (MHz)***	376.50
Nucleus	19F	***Number of Transients***	16 ***Original Points Count*** 131072	***Points Count***	262144
Pulse Sequence	zgflqn	***Solvent***	CHLOROFORM-D	***Sweep Width (Hz)***	75187.97
Temperature (degree C)	27.000				

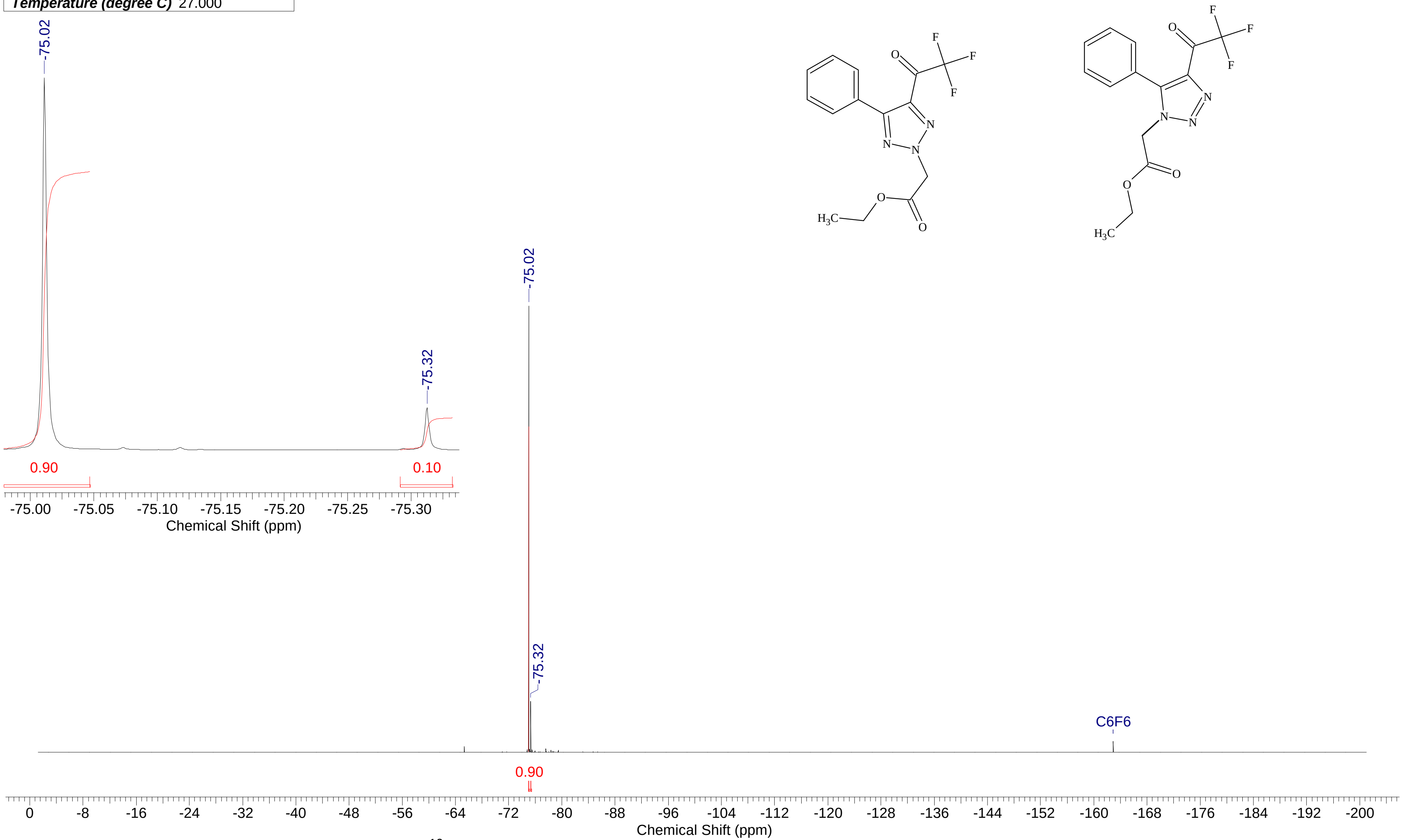

^{19}F NMR spectrum of mixture of **3o** and **4o** (376.5 MHz, $CDCl_3$)

18 May 2022

Acquisition Time (sec)	4.0894	***Comment***	Imported from UXNMR.			***Date***	18 May 2022 17:12:22
File Name	C:\DOCS\OUTPUT_301\2022\05.май\BM-2494-2p.H_001001r					***Frequency (MHz)***	400.13
Nucleus	1H	***Number of Transients***	4	***Original Points Count***	32768	***Points Count***	131072
Pulse Sequence	zg30	***Solvent***	BENZENE-D6	***Sweep Width (Hz)***	8012.82	***Temperature (degree C)***	27.000

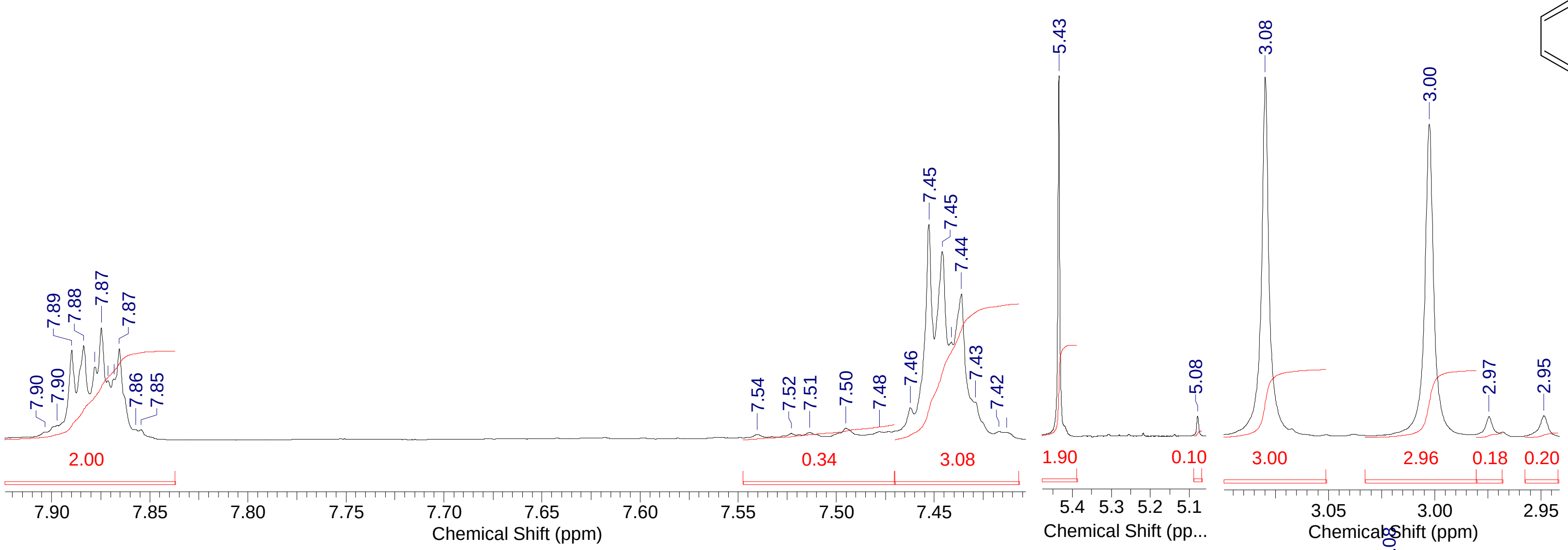

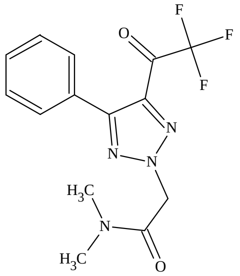

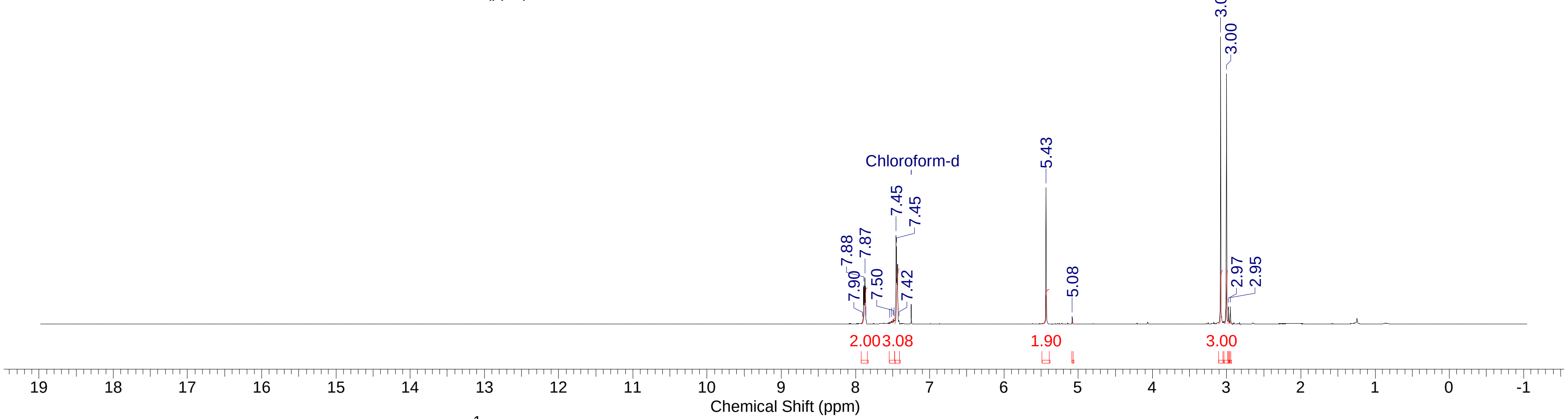

^{1}H NMR spectrum of mixture of **3p** and **4p** (400.1 MHz, $CDCl_3$)

18 May 2022

Acquisition Time (sec)	1.7433	***Comment***	Imported from UXNMR.			***Date***	18 Apr 2022 15:31:52
File Name	C:\DOCS\OUTPUT_301\2022\04.апрель\BM-2494-2c.F_005001r					***Frequency (MHz)***	376.50
Nucleus	19F	***Number of Transients***	16	***Original Points Count***	131072	***Points Count***	262144
Pulse Sequence	zgflqn	***Solvent***	CHLOROFORM-D			***Sweep Width (Hz)***	75187.97
Temperature (degree C)	27.000						

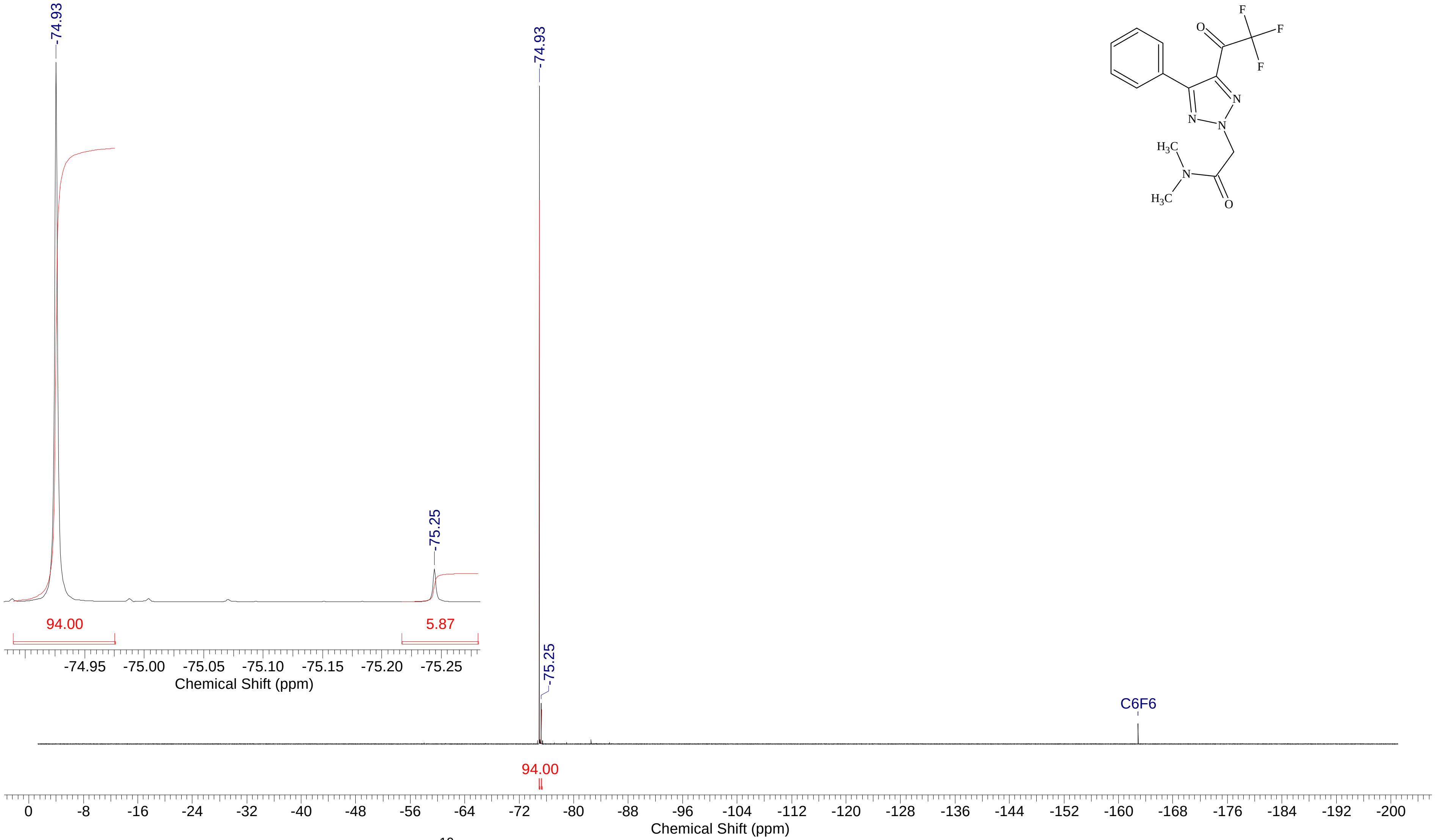

^{19}F NMR spectrum of mixture of **3p** and **4p** (376.5 MHz, $CDCl_3$)

18 May 2022

Acquisition Time (sec)	0.6783	***Comment***	Imported from UXNMR.			***Date***	16 Apr 2022 14:05:18
File Name	C:\DOCS\BM\ЯМР_СОСЕДИ\2022\16-04-2022_14-19-08\BM-2494-2c\BM-2494-2c_002001r					***Frequency (MHz)***	100.61
Nucleus	13C	***Number of Transients***	176	***Original Points Count***	16384	***Points Count***	131072
Pulse Sequence	zgpg30	***Solvent***	CHLOROFORM-D	***Sweep Width (Hz)***	24154.59	***Temperature (degree C)***	27.000

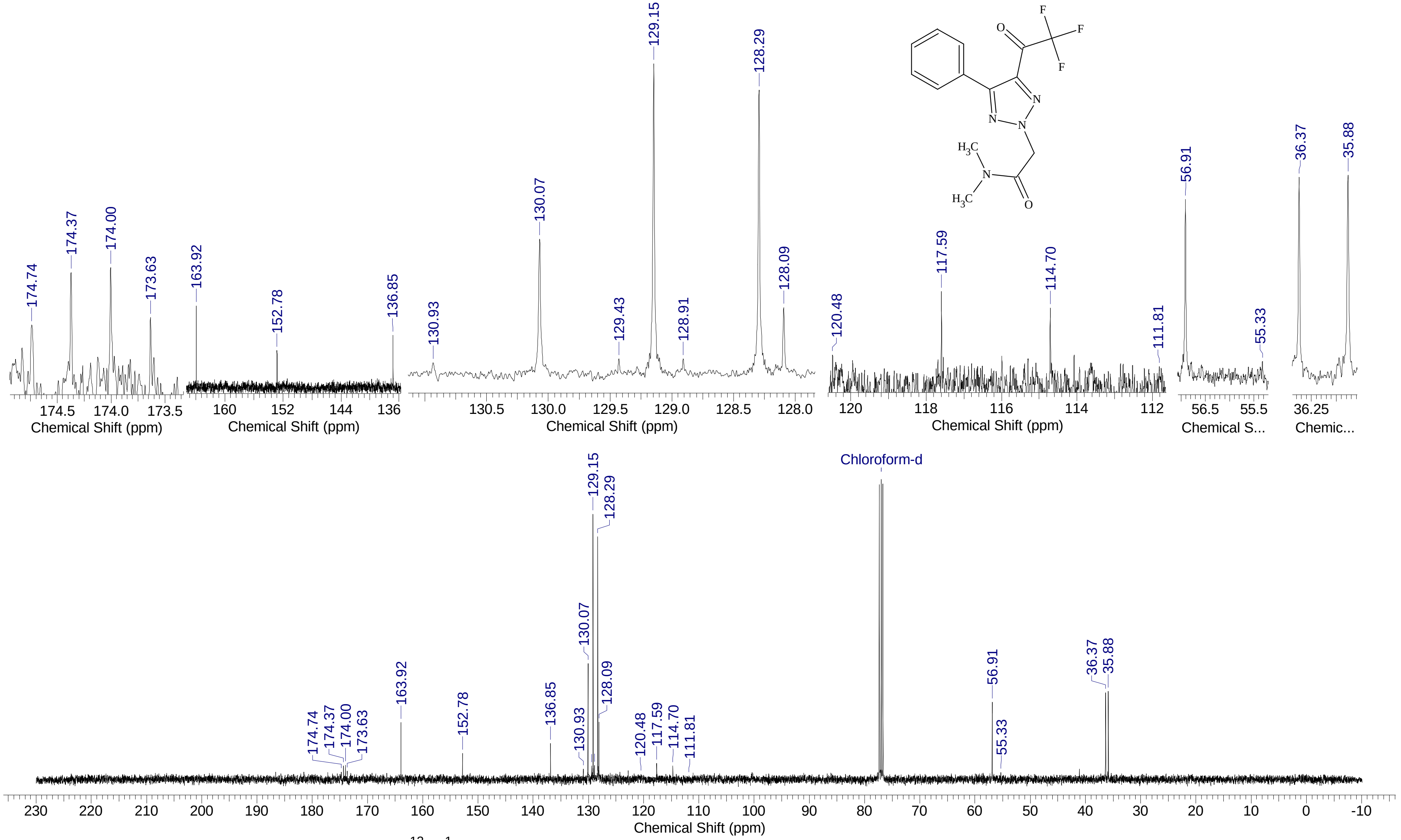

$^{13}C\{^1H\}$ NMR spectrum of mixture of **3p** and **4p** (100.6 MHz, $CDCl_3$)

18 May 2022

Acquisition Time (sec)	4.0894	Comment	Imported from UXNMR.			Date	14 Apr 2022 12:39:22
File Name	C:\DOCS\OUTPUT_301\2022\04.апрель\BM-2494-2.H_001001r					Frequency (MHz)	400.13
Nucleus	1H	Number of Transients	4	Original Points Count	32768	Points Count	131072
Pulse Sequence	zg30	Solvent	ACETONITRILE-D3			Sweep Width (Hz)	8012.82
Temperature (degree C)	27.000						

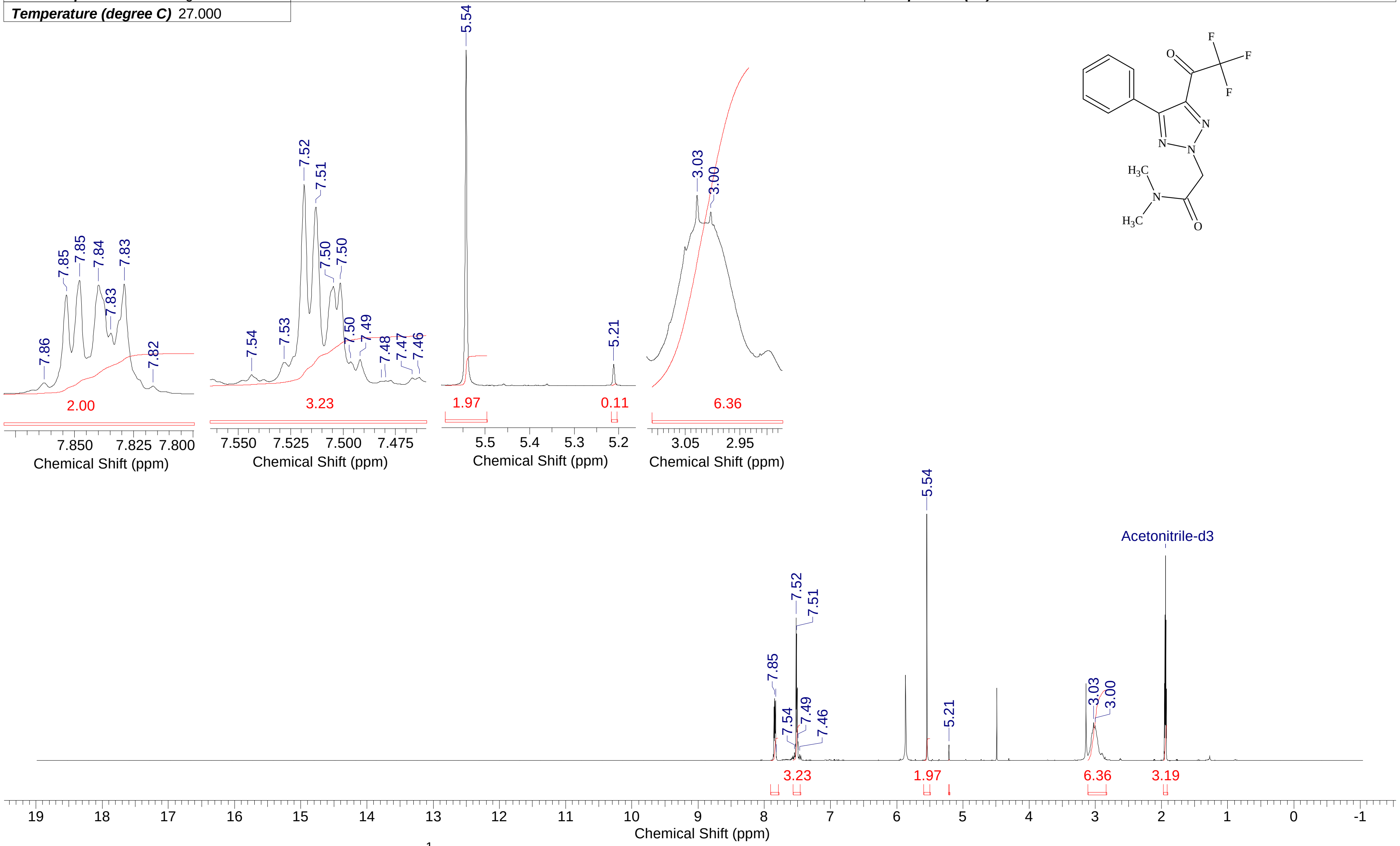

^{1}H NMR spectrum of mixture of **3p** and **4p** (400.1 MHz, CD_3CN)

18 May 2022

Acquisition Time (sec)	1.7433	***Comment***	Imported from UXNMR.			***Date***	14 Apr 2022 12:37:52
File Name	C:\DOCS\OUTPUT_301\2022\04.апрель\BM-2494-2.F_005001r					***Frequency (MHz)***	376.50
Nucleus	19F	***Number of Transients***	16	***Original Points Count***	131072	***Points Count***	262144
Pulse Sequence	zgflqn	***Solvent***	CHLOROFORM-D			***Sweep Width (Hz)***	75187.97
Temperature (degree C)	27.000						

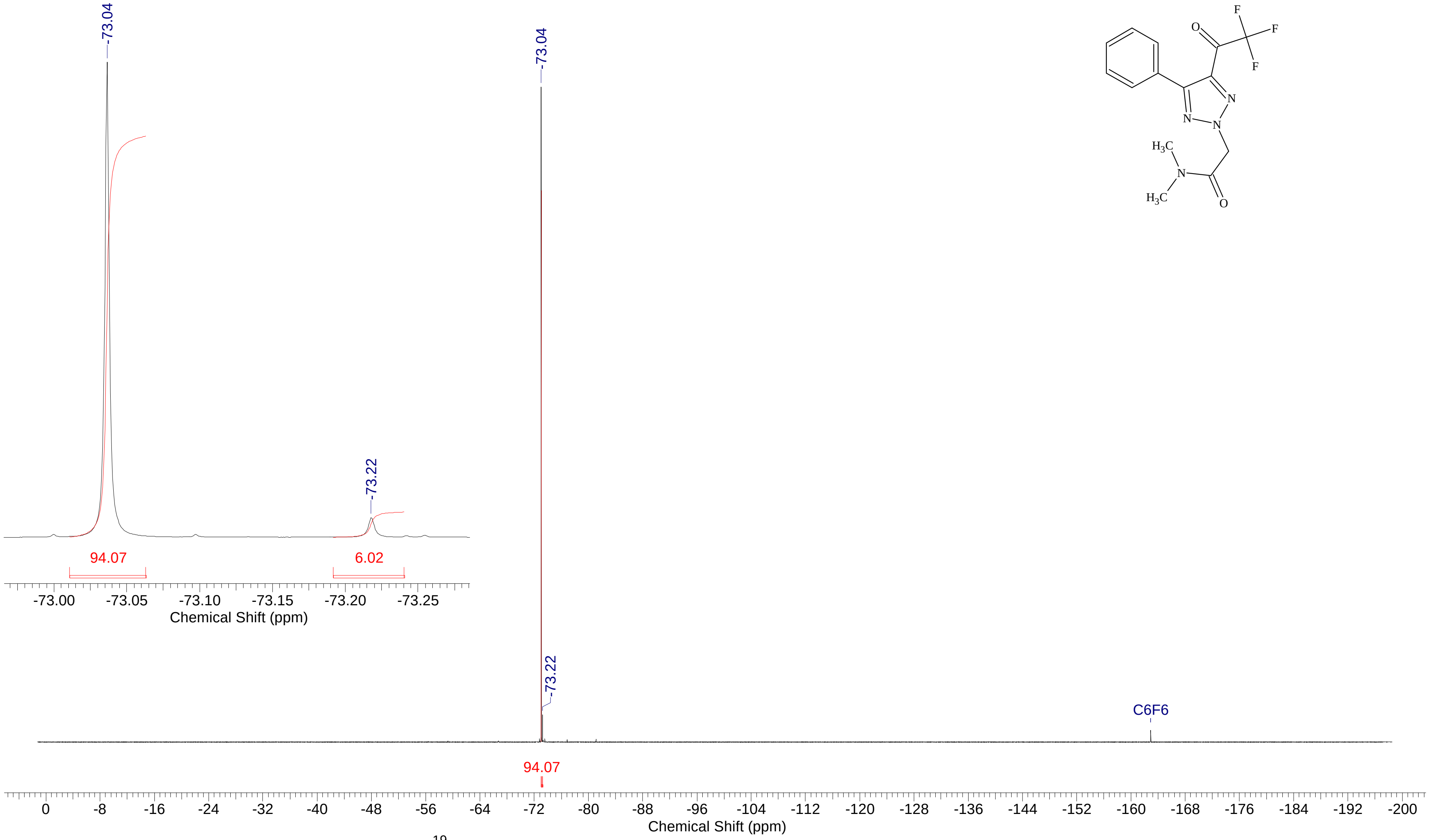

^{19}F NMR spectrum of mixture of **3p** and **4p** (376.5 MHz, CD_3CN)

18 May 2022

Acquisition Time (sec)	0.6783	***Comment***	Imported from UXNMR.	***Date***	14 Apr 2022 13:05:18
File Name	C:\DOCS\OUTPUT_301\2022\04.апрель\BM-2494-2.C_002001r			***Frequency (MHz)***	100.61
Nucleus	13C	***Number of Transients***	601 ***Original Points Count*** 16384	***Points Count***	131072
Pulse Sequence	zgpg30	***Solvent***	CHLOROFORM-D	***Sweep Width (Hz)***	24154.59
Temperature (degree C)	27.000				

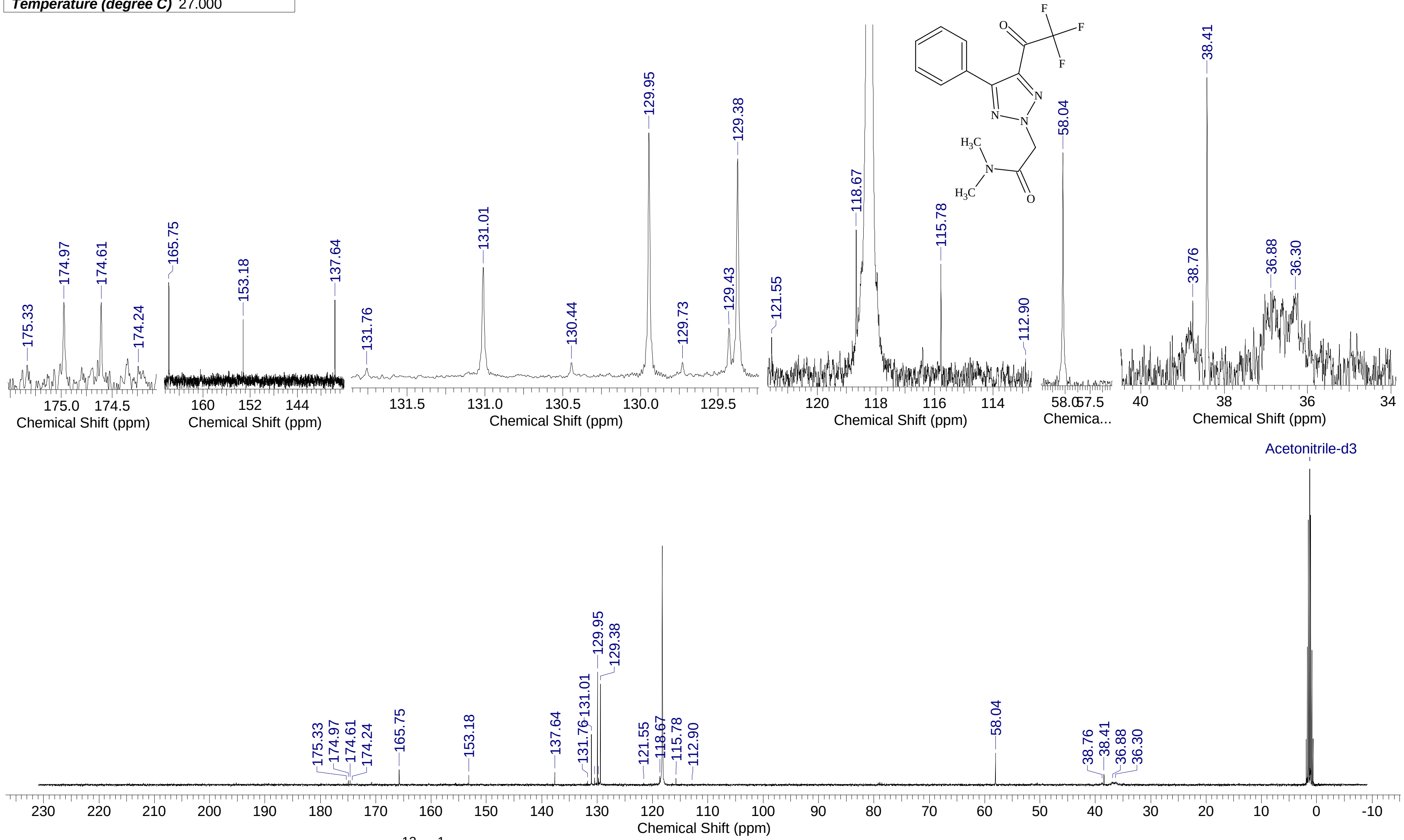

$^{13}C\{^1H\}$ NMR spectrum of mixture of **3p** and **4p** (100.6 MHz, CD_3CN)

Acquisition Time (sec)	4.0894	***Comment***	Imported from UXNMR.	***Date***	30 Mar 2022 17:23:40
File Name	C:\DOCS\OUTPUT_301\2022\03.март\SZA-BM-2470.H_001001r			***Frequency (MHz)***	400.13
Nucleus	1H	***Number of Transients***	4 ***Original Points Count*** 32768	***Points Count***	131072
Pulse Sequence	zg30	***Solvent***	CHLOROFORM-D	***Sweep Width (Hz)***	8012.82
Temperature (degree C)	27.000				

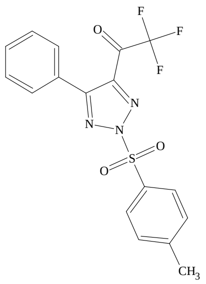

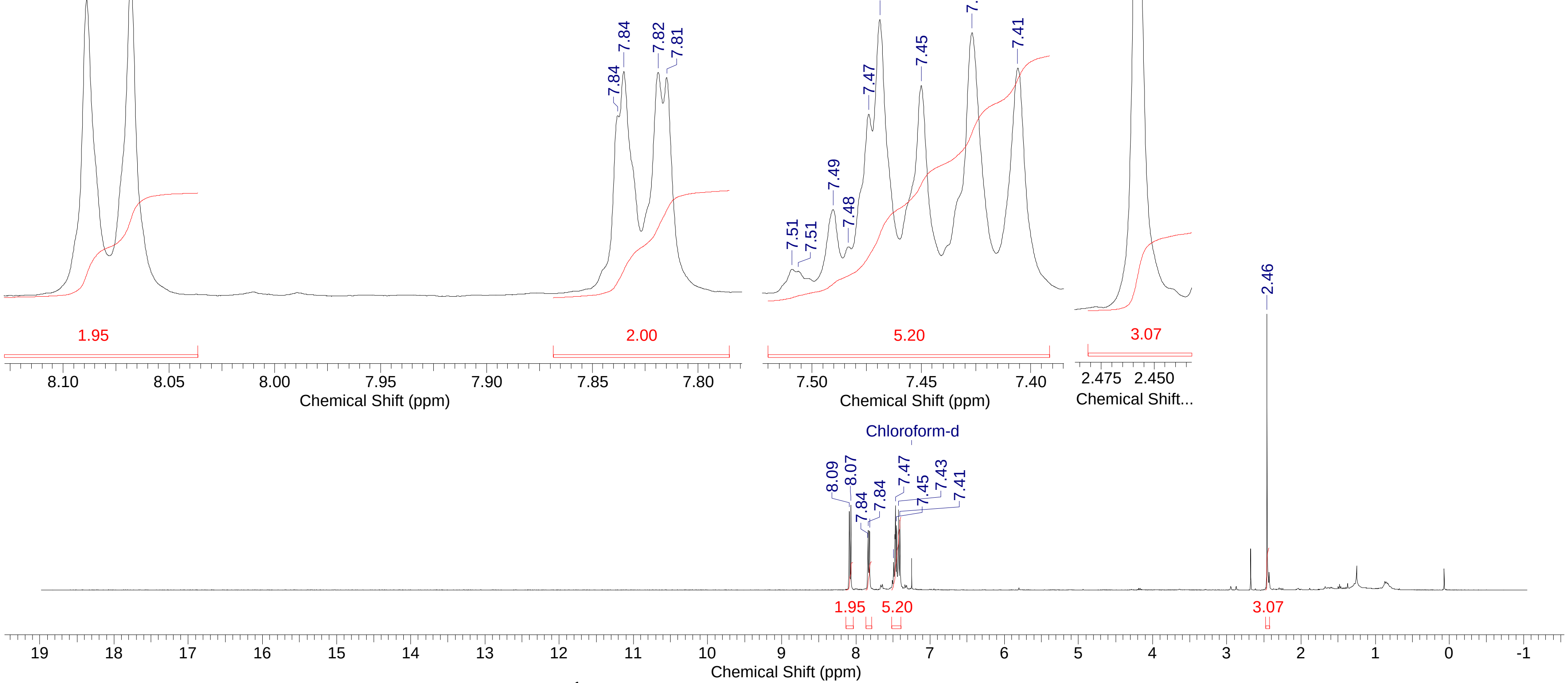

^{1}H NMR spectrum of **3q** (400.1 MHz, $CDCl_3$)

19 May 2022

Acquisition Time (sec)	1.7433	***Comment***	Imported from UXNMR.			***Date***	30 Mar 2022 17:49:38
File Name	C:\DOCS\OUTPUT_301\2022\03.март\SZA-BM-2470.F_005001r					***Frequency (MHz)***	376.50
Nucleus	19F	***Number of Transients***	16	***Original Points Count***	131072	***Points Count***	262144
Pulse Sequence	zgflqn	***Solvent***	CHLOROFORM-D			***Sweep Width (Hz)***	75187.97
Temperature (degree C)	27.000						

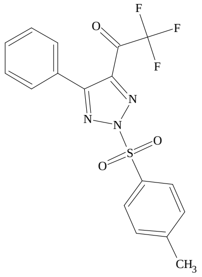

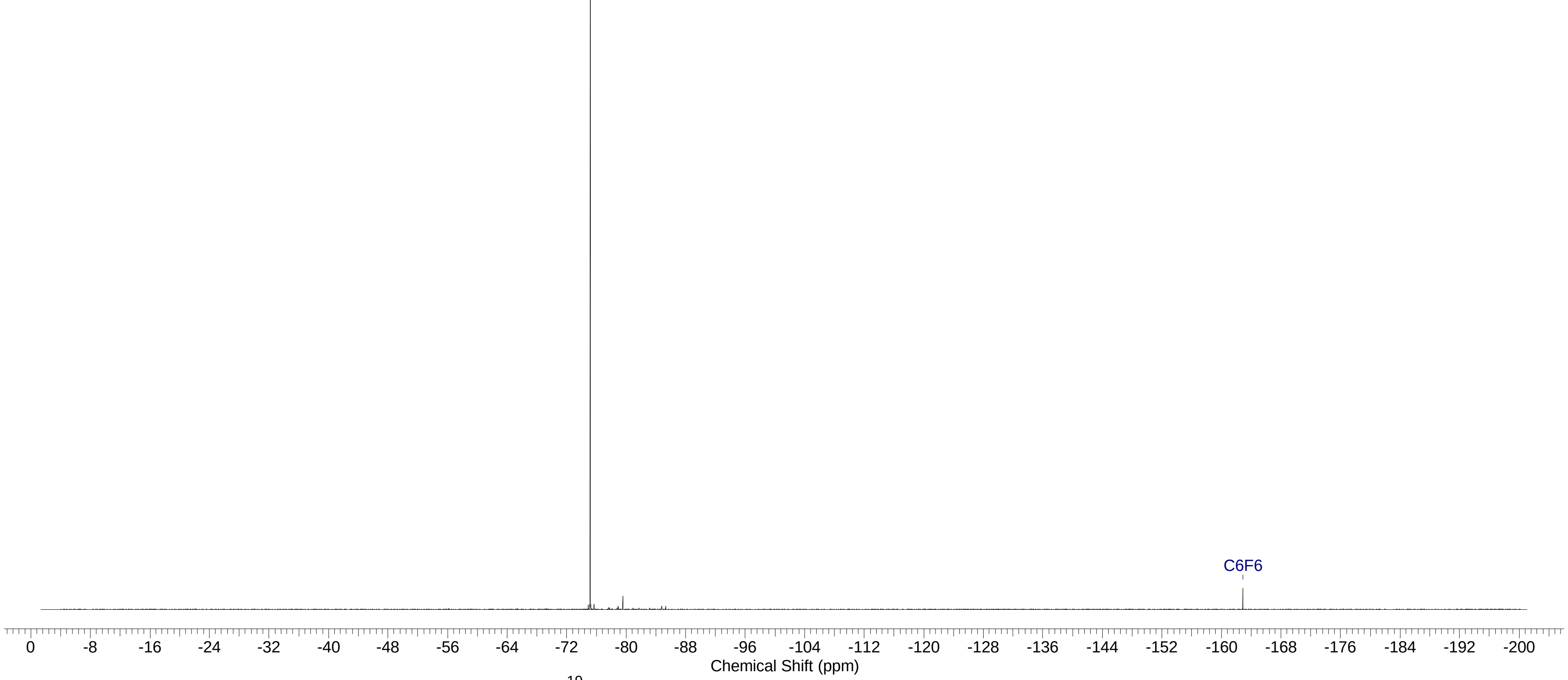

^{19}F NMR spectrum of **3q** (376.5 MHz, $CDCl_3$)

19 May 2022

**Acquisition Time (sec)**	0.6783	**Comment**	Imported from UXNMR.			**Date**	31 Mar 2022 12:54:26
**File Name**	C:\DOCS\OUTPUT_301\2022\03.март\SZA-BM-2470.C_002001r					**Frequency (MHz)**	100.61
**Nucleus**	13C	**Number of Transients**	305	**Original Points Count**	16384	**Points Count**	131072
**Pulse Sequence**	zgpg30	**Solvent**	DMSO-D6	**Sweep Width (Hz)**	24154.59	**Temperature (degree C)**	27.000

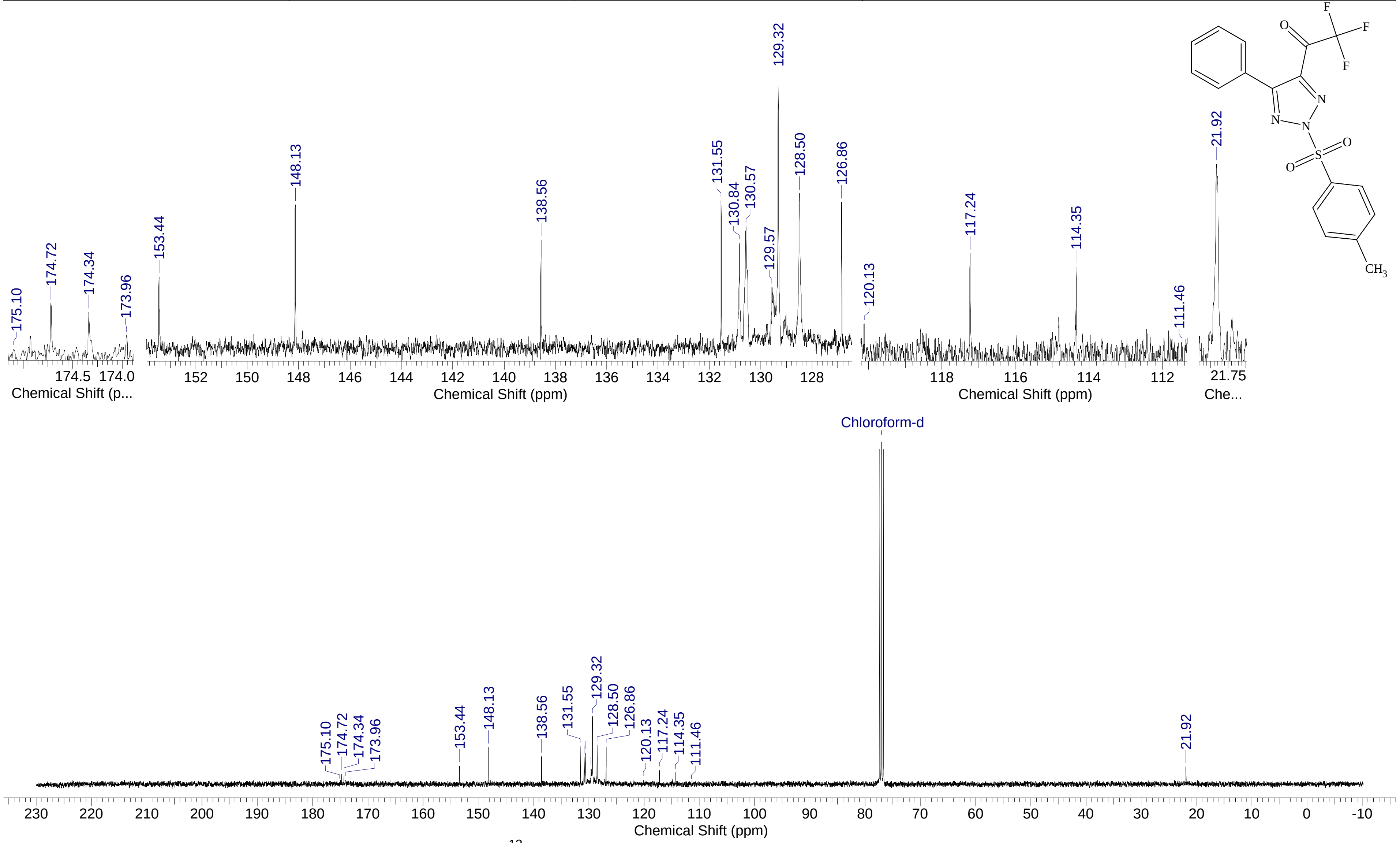

^{13}C NMR spectrum of **3q** (100.6 MHz, $CDCl_3$)

**Acquisition Time (sec)**	4.0894	**Comment**	Imported from UXNMR.			**Date**	14 Apr 2022 11:55:20
**File Name**	C:\DOCS\OUTPUT_301\2022\04.апрель\SZA-BM-2498.H_001001r					**Frequency (MHz)**	400.13
**Nucleus**	1H	**Number of Transients**	4	**Original Points Count**	32768	**Points Count**	131072
**Pulse Sequence**	zg30	**Solvent**	BENZENE-D6	**Sweep Width (Hz)**	8012.82	**Temperature (degree C)**	27.000

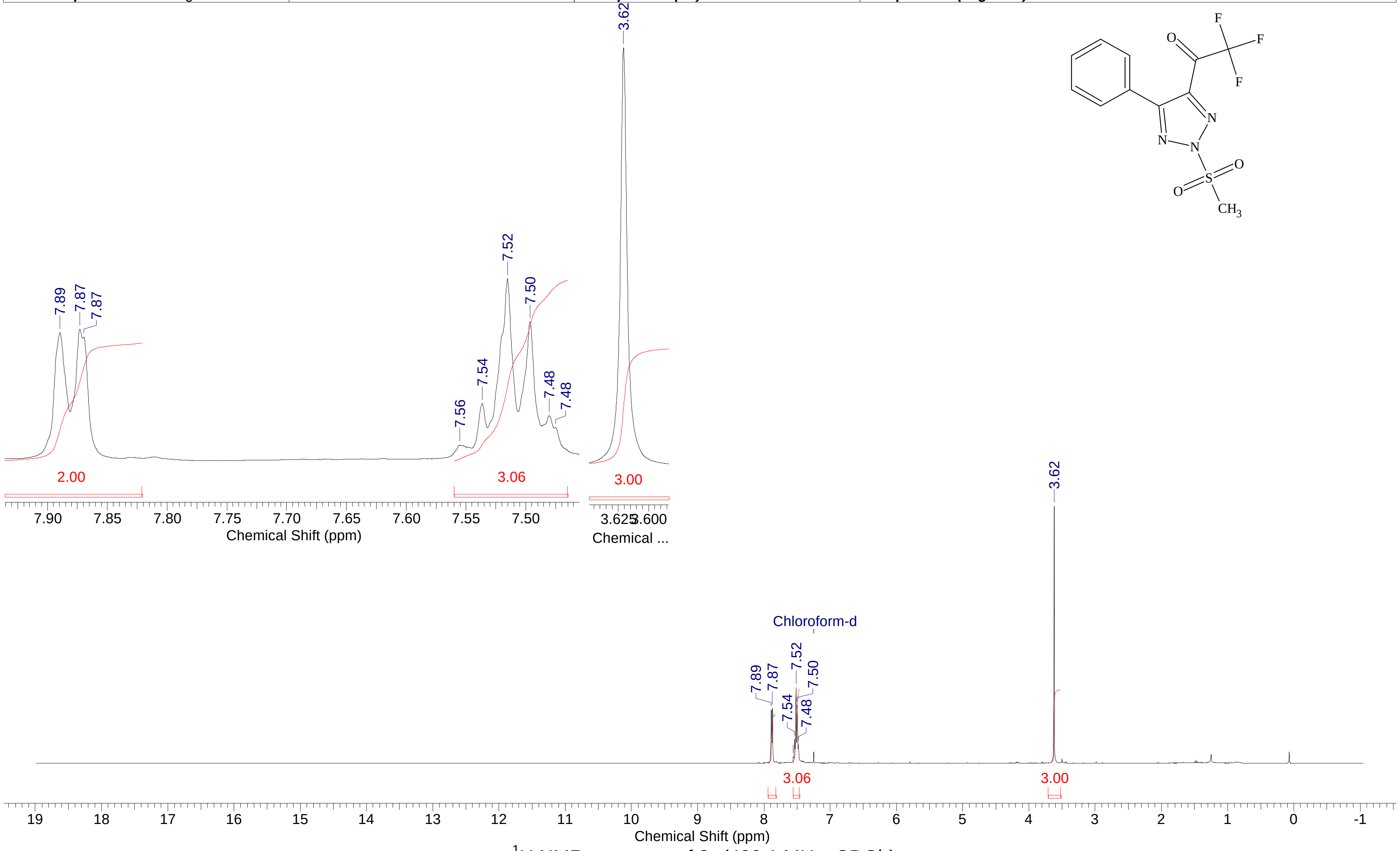

^{1}H NMR spectrum of **3r** (400.1 MHz, $CDCl_3$)

19 May 2022

Acquisition Time (sec)	1.7433	Comment	Imported from UXNMR.			Date	14 Apr 2022 13:21:22
File Name	C:\DOCS\OUTPUT_301\2022\04.апрель\SZA-BM-2498.F_005001r					Frequency (MHz)	376.50
Nucleus	19F	Number of Transients	16	Original Points Count	131072	Points Count	262144
Pulse Sequence	zgflqn	Solvent	CHLOROFORM-D			Sweep Width (Hz)	75187.97
Temperature (degree C)	27.000						

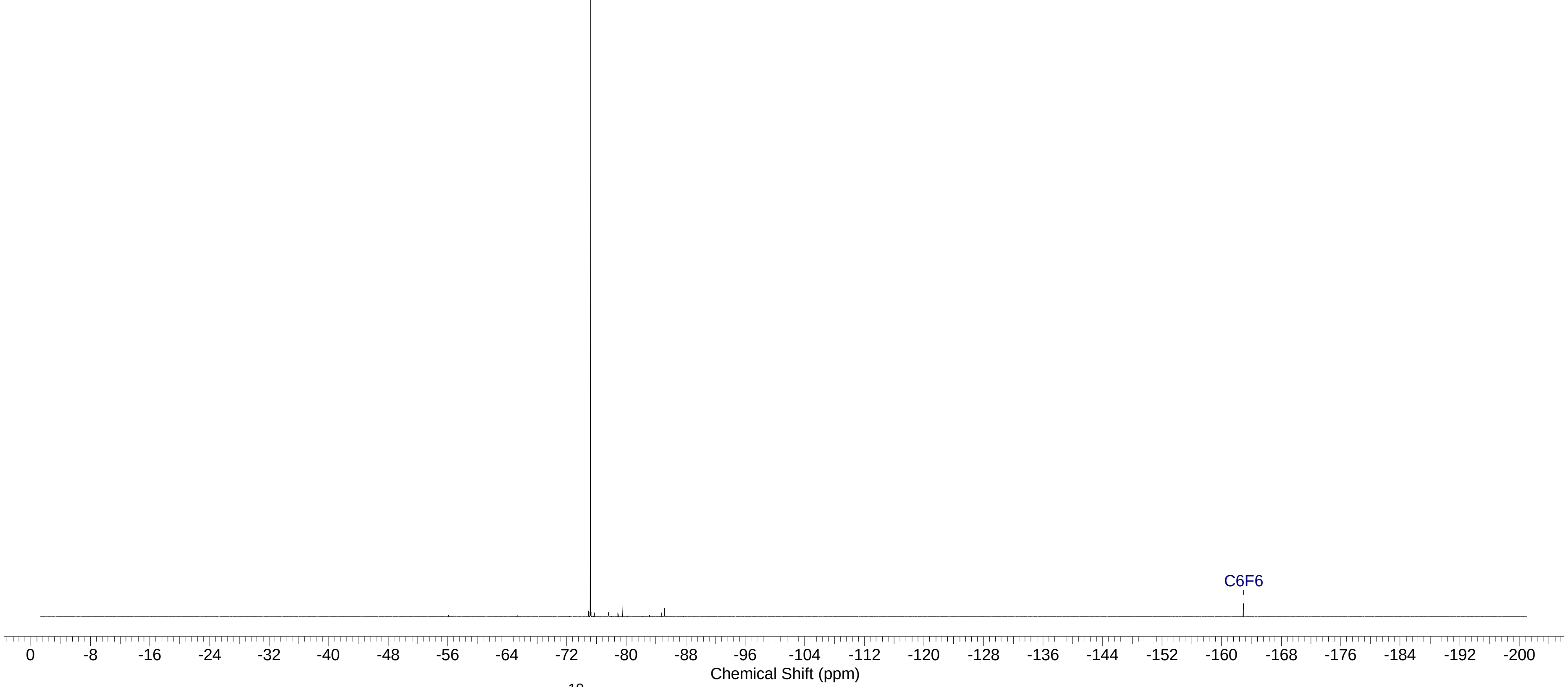

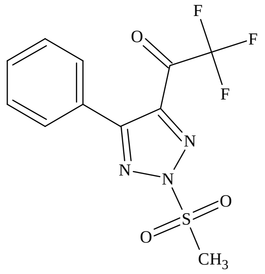

^{19}F NMR spectrum of **3r** (376.5 MHz, $CDCl_3$)

19 May 2022

Acquisition Time (sec)	0.6783	***Comment***	Imported from UXNMR.			***Date***	15 Apr 2022 16:00:22
File Name	C:\DOCS\OUTPUT_301\2022\04.апрель\SZA-BM-2498.C_002001r					***Frequency (MHz)***	100.61
Nucleus	13C	***Number of Transients***	201	***Original Points Count***	16384	***Points Count***	131072
Pulse Sequence	zgpg30	***Solvent***	CHLOROFORM-D			***Sweep Width (Hz)***	24154.59
Temperature (degree C)	27.000						

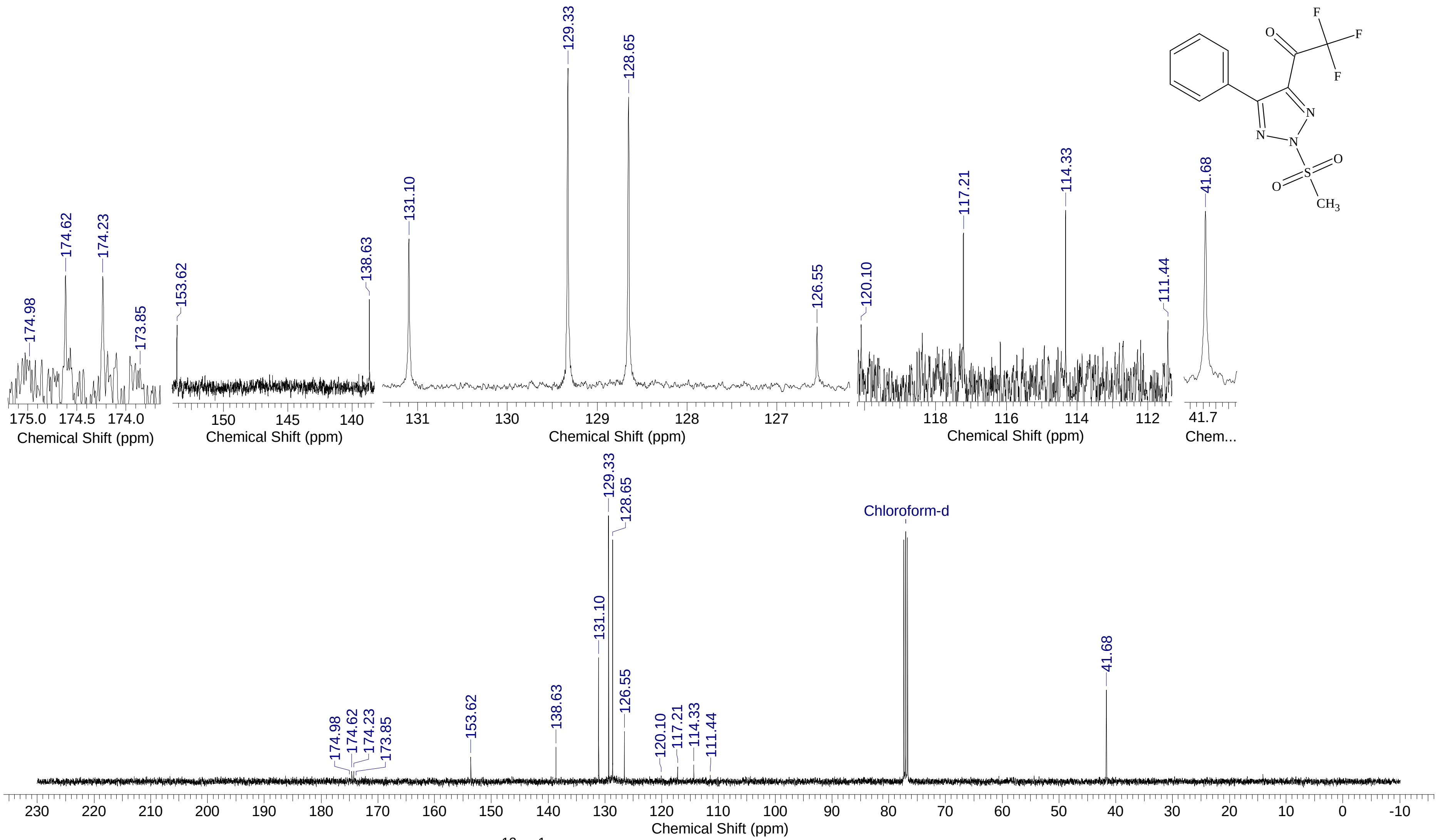

$^{13}C\{^{1}H\}$ NMR spectrum of **3r** (100.6 MHz, $CDCl_3$)

19 May 2022

Acquisition Time (sec)	4.0894	***Comment***	Imported from UXNMR.			***Date***	22 Mar 2022 15:43:50
File Name	C:\DOCS\OUTPUT_301\2022\03.март\BM-2461-1p.H_001001r					***Frequency (MHz)***	400.13
Nucleus	1H	***Number of Transients***	4	***Original Points Count***	32768	***Points Count***	131072
Pulse Sequence	zg30	***Solvent***	CHLOROFORM-D			***Sweep Width (Hz)***	8012.82
Temperature (degree C)	27.000						

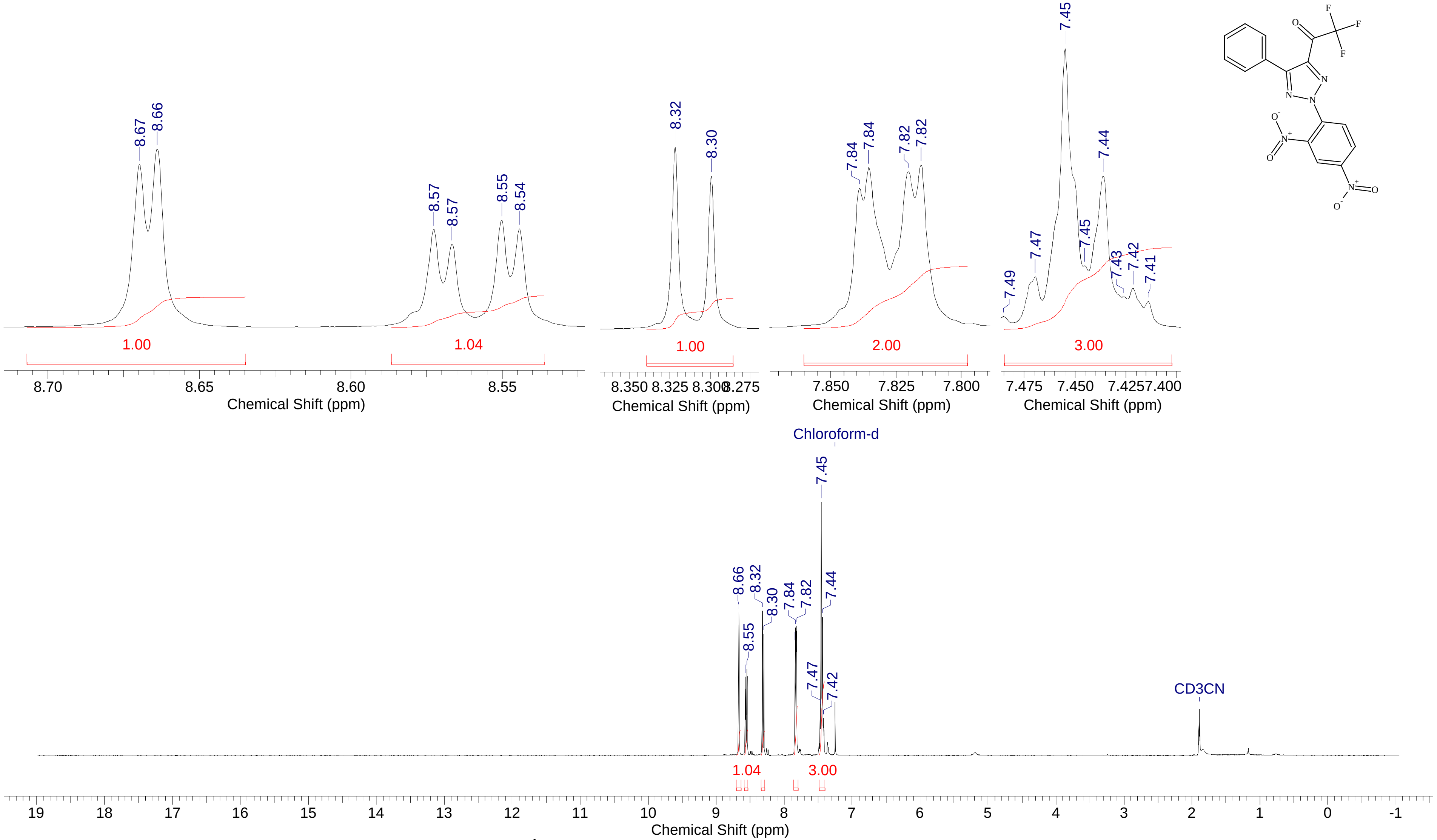

^{1}H NMR spectrum of **5** (400.1 MHz, $CDCl_3$)

19 May 2022

Acquisition Time (sec)	1.7433	***Comment***	Imported from UXNMR.			***Date***	22 Mar 2022 15:28:42
File Name	C:\DOCS\OUTPUT_301\2022\03.март\BM-2461-1p.F_005001r					***Frequency (MHz)***	376.50
Nucleus	19F	***Number of Transients***	4	***Original Points Count***	131072	***Points Count***	262144
Pulse Sequence	zgflqn	***Solvent***	CHLOROFORM-D			***Sweep Width (Hz)***	75187.97
Temperature (degree C)	27.000						

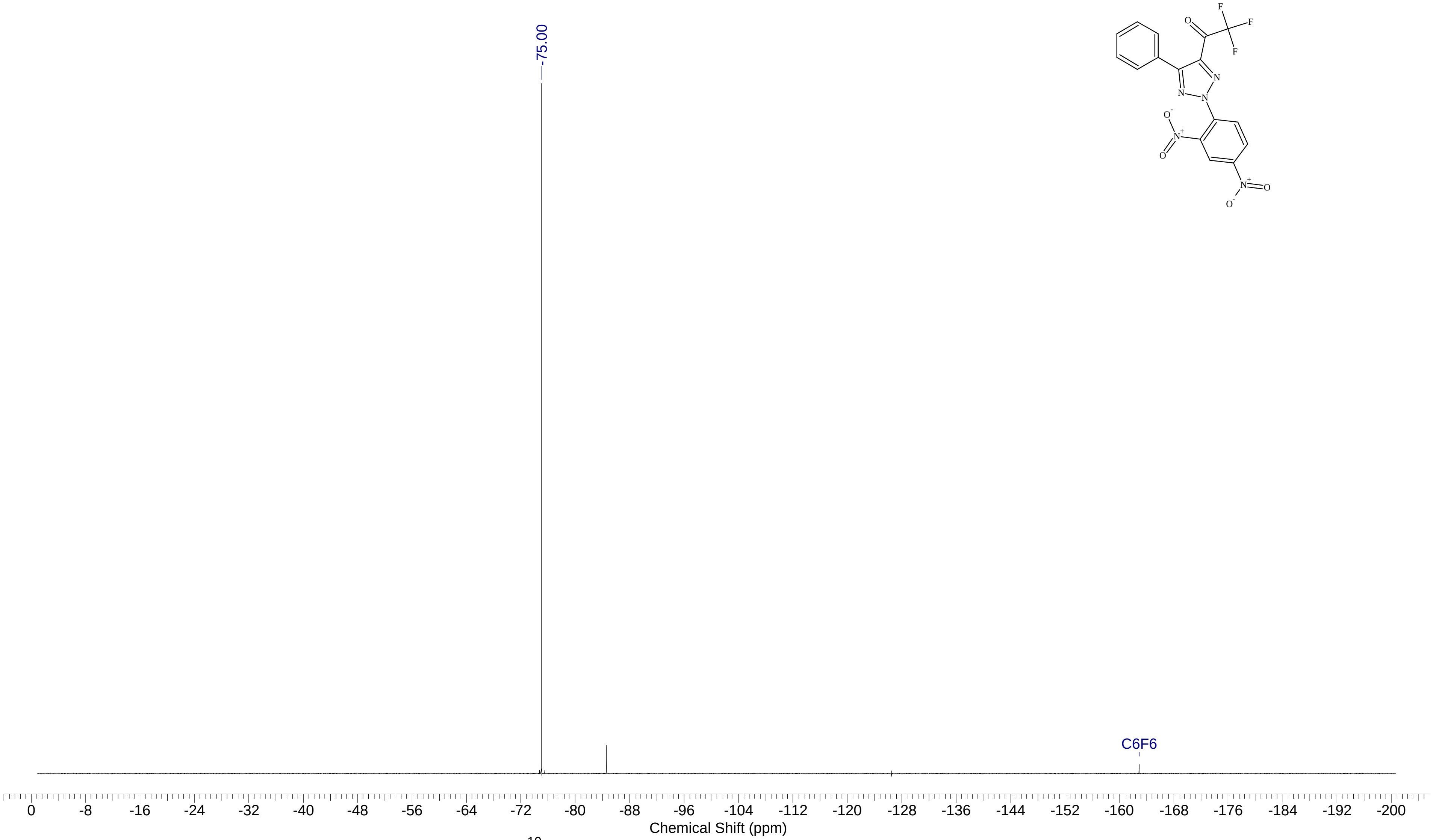

^{19}F NMR spectrum of **5** (376.5 MHz, $CDCl_3$)

19 May 2022

Acquisition Time (sec)	0.6783	***Comment***	Imported from UXNMR.			***Date***	22 Mar 2022 15:54:02
File Name	C:\DOCS\OUTPUT_301\2022\03.март\BM-2461-1p.C_002001r					***Frequency (MHz)***	100.61
Nucleus	13C	***Number of Transients***	266	***Original Points Count***	16384	***Points Count***	131072
Pulse Sequence	zgpg30	***Solvent***	DMSO-D6	***Sweep Width (Hz)***	24154.59	***Temperature (degree C)***	27.000

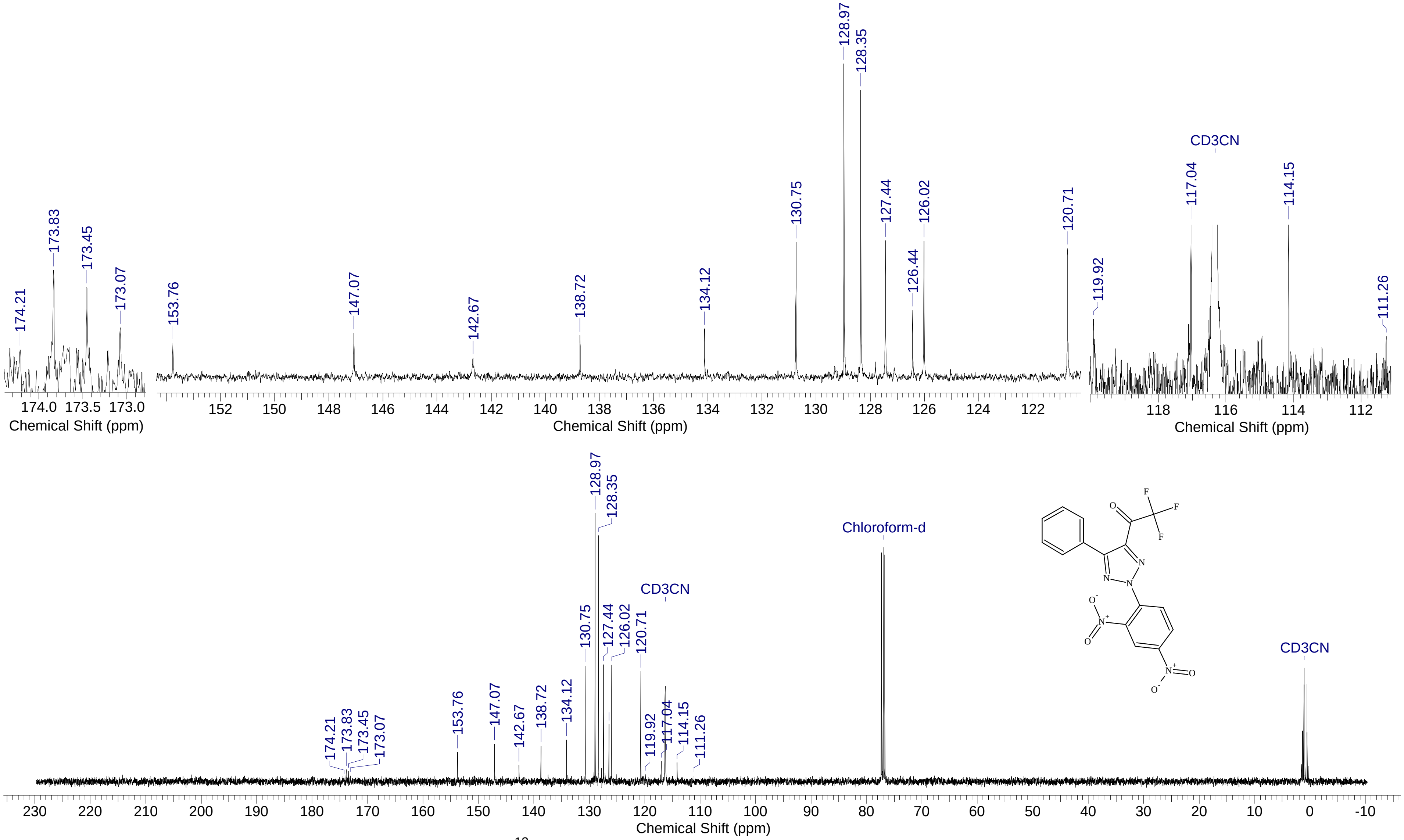

^{13}C NMR spectrum of **5** (100.6 MHz, $CDCl_3$)

25 Nov 2022

Acquisition Time (sec)	4.0894	***Comment***	Imported from UXNMR.	***Date***	01 Nov 2022 14:16:34
File Name	C:\DOCS\OUTPUT_301\2022\11.ноябрь\SZA-BM-2671-9.H_001001r			***Frequency (MHz)***	400.13
Nucleus	1H	***Number of Transients***	4	***Points Count***	131072
		Original Points Count	32768		
Pulse Sequence	zg30	***Solvent***	CHLOROFORM-D	***Sweep Width (Hz)***	8012.82
Temperature (degree C)	27.000				

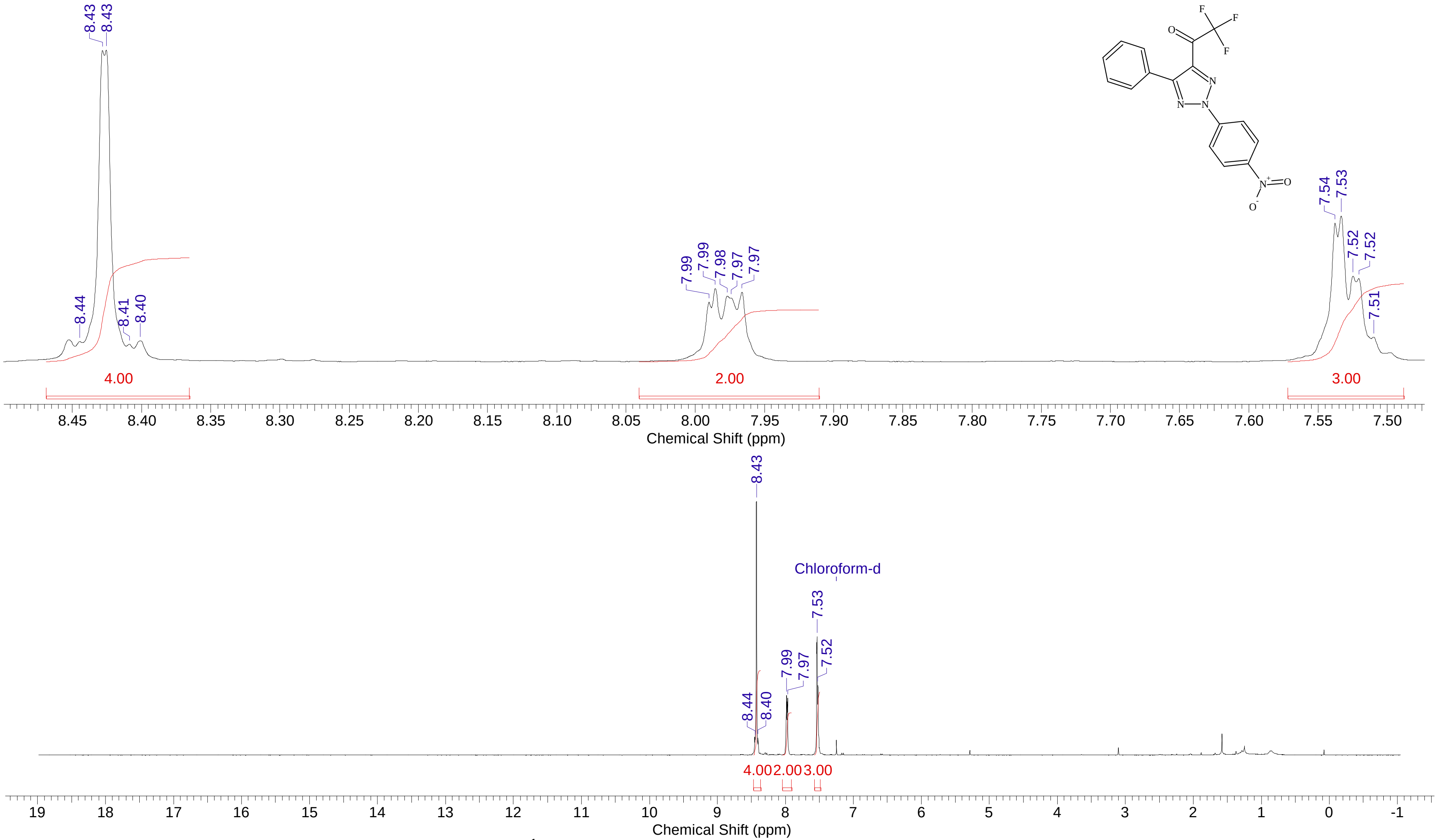

^{1}H NMR spectrum of **6** (400.1 MHz, $CDCl_3$)

25 Nov 2022

Acquisition Time (sec)	1.7433	Comment	Imported from UXNMR.			Date	01 Nov 2022 15:46:26
File Name	C:\DOCS\OUTPUT_301\2022\11.ноябрь\SZA-BM-2671-9.F_005001r					Frequency (MHz)	376.50
Nucleus	19F	Number of Transients	16	Original Points Count	131072	Points Count	262144
Pulse Sequence	zgflqn	Solvent	ACETONITRILE-D3			Sweep Width (Hz)	75187.97
Temperature (degree C)	27.000						

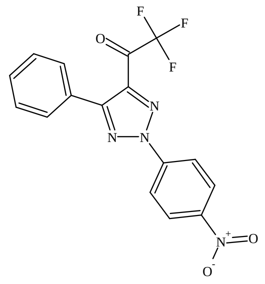

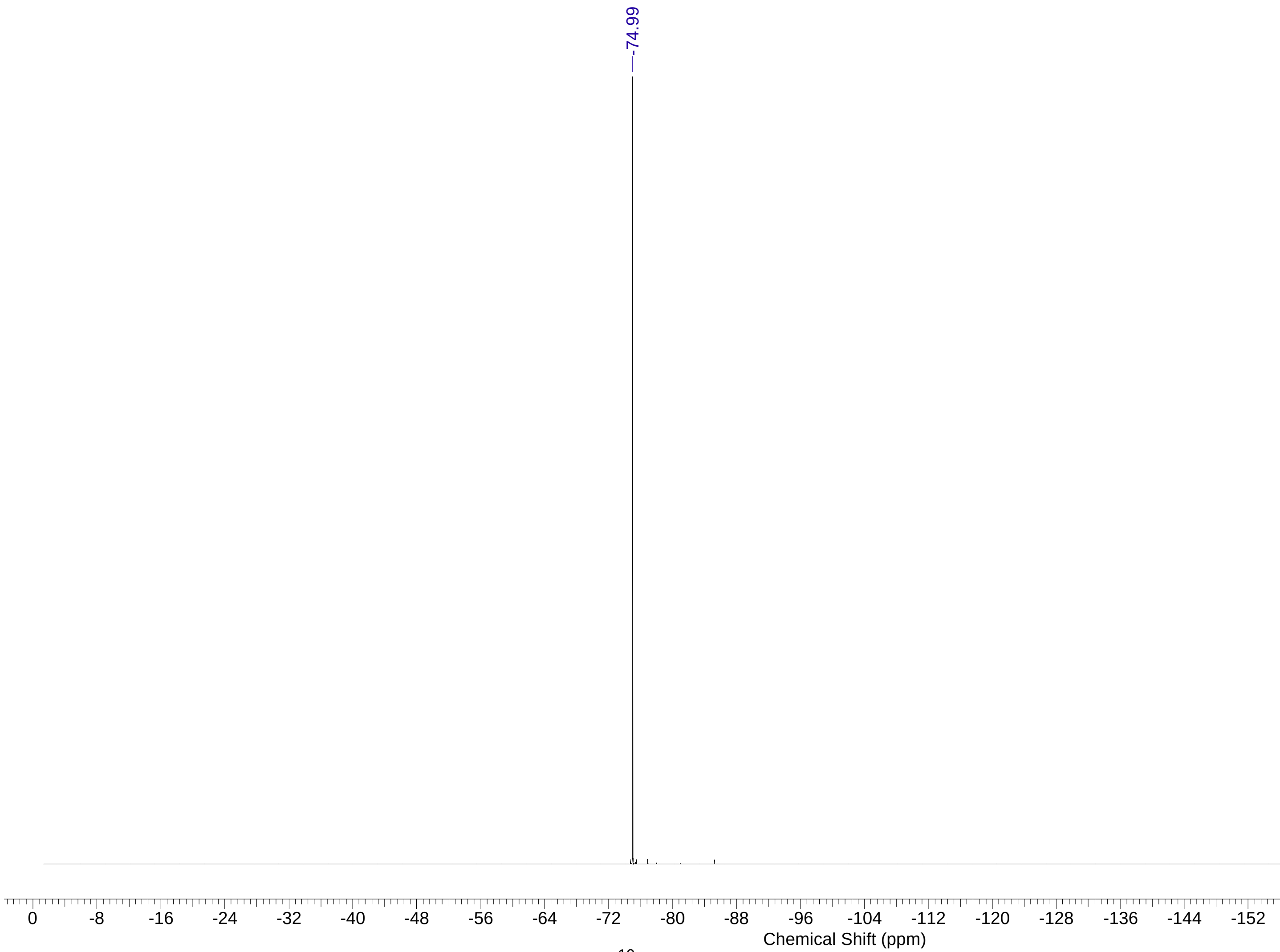

^{19}F NMR spectrum of **6** (376.5 MHz, $CDCl_3$)

25 Nov 2022

Acquisition Time (sec)	0.6783	Comment	Imported from UXNMR.			Date	01 Nov 2022 14:31:16
File Name	C:\DOCS\OUTPUT_301\2022\11.ноябрь\SZA-BM-2671-9.C_002001r					Frequency (MHz)	100.61
Nucleus	13C	Number of Transients	305	Original Points Count	16384	Points Count	131072
Pulse Sequence	zgpg30	Solvent	CHLOROFORM-D			Sweep Width (Hz)	24154.59
Temperature (degree C)	27.000						

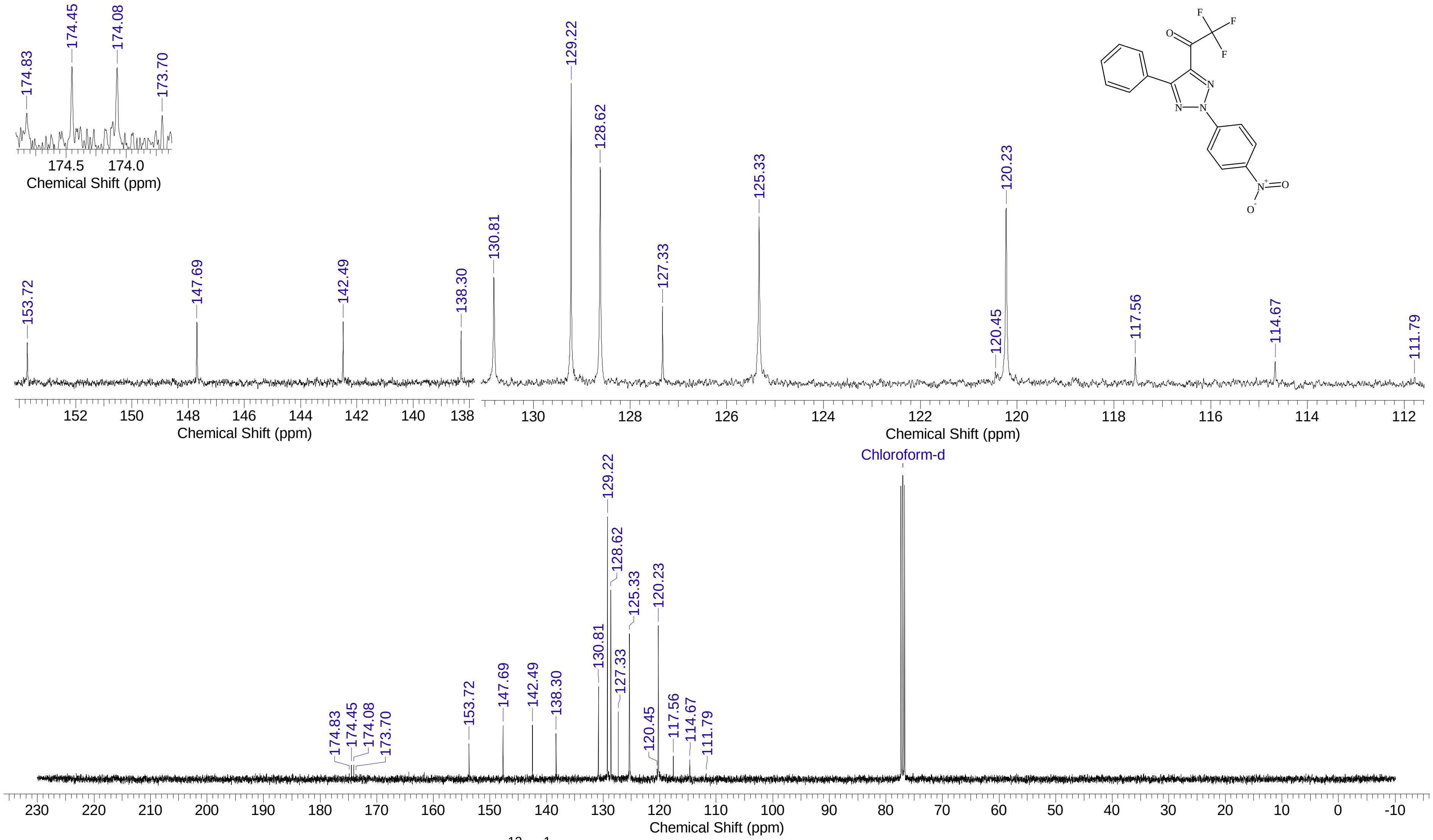

$^{13}C\{^1H\}$ NMR spectrum of **6** (100.6 MHz, $CDCl_3$)

19 May 2022

Acquisition Time (sec)	4.0894	***Comment***	Imported from UXNMR.			***Date***	08 Apr 2022 15:46:58
File Name	C:\DOCS\OUTPUT_301\2022\04.апрель\SZA-BM-2488-7.H_001001r					***Frequency (MHz)***	400.13
Nucleus	1H	***Number of Transients***	4	***Original Points Count***	32768	***Points Count***	131072
Pulse Sequence	zg30	***Solvent***	CHLOROFORM-D			***Sweep Width (Hz)***	8012.82
Temperature (degree C)	27.000						

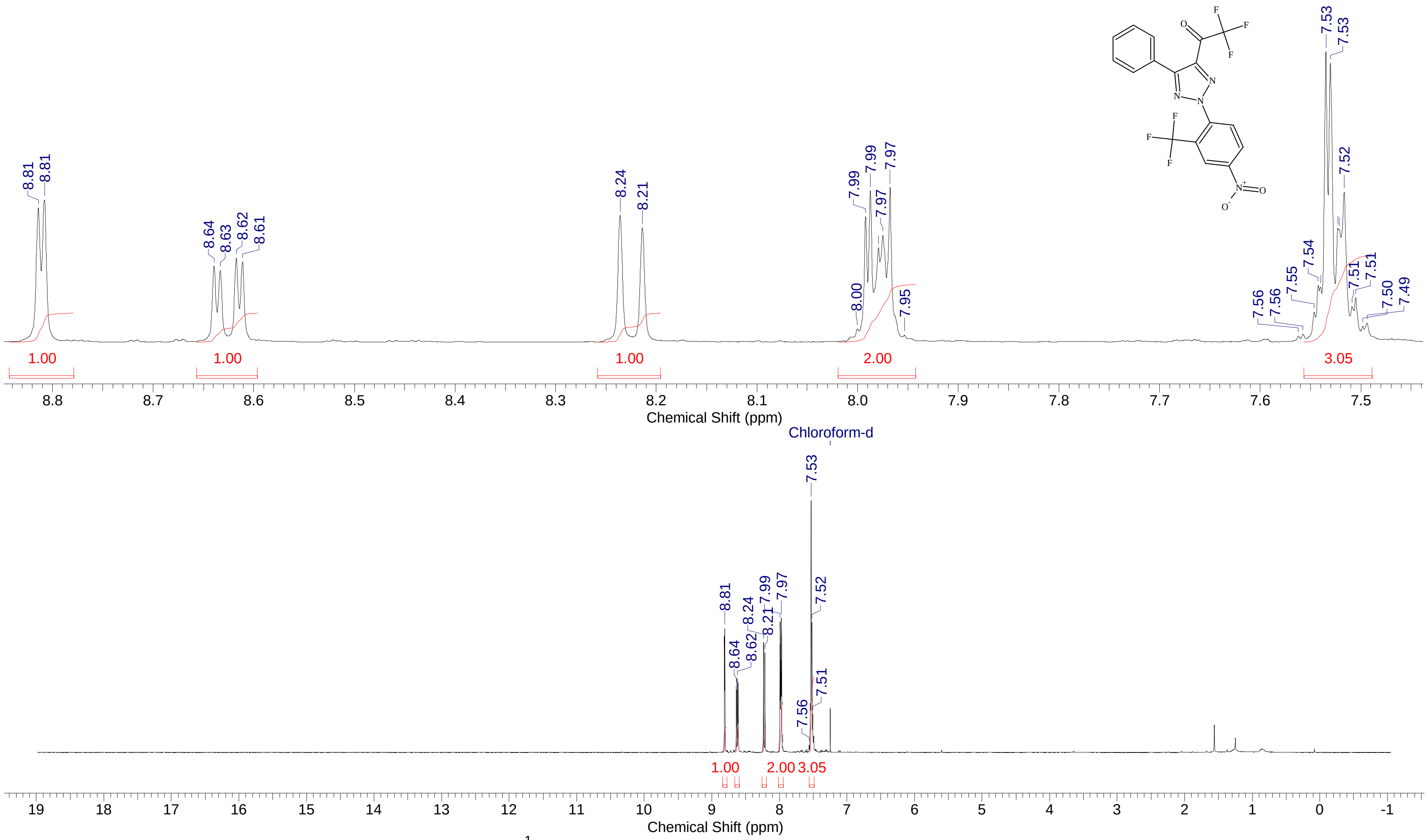

^{1}H NMR spectrum of **7** (400.1 MHz, $CDCl_3$)

19 May 2022

Acquisition Time (sec)	1.7433	***Comment***	Imported from UXNMR.	***Date***	08 Apr 2022 15:45:36
File Name	C:\DOCS\OUTPUT_301\2022\04.апрель\SZA-BM-2488-7.F_005001r			***Frequency (MHz)***	376.50
Nucleus	19F	***Number of Transients***	10 ***Original Points Count*** 131072	***Points Count***	262144
Pulse Sequence	zgflqn	***Solvent***	CHLOROFORM-D	***Sweep Width (Hz)***	75187.97
Temperature (degree C)	27.000				

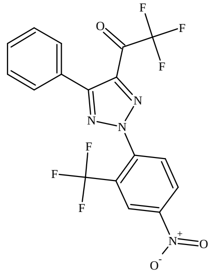

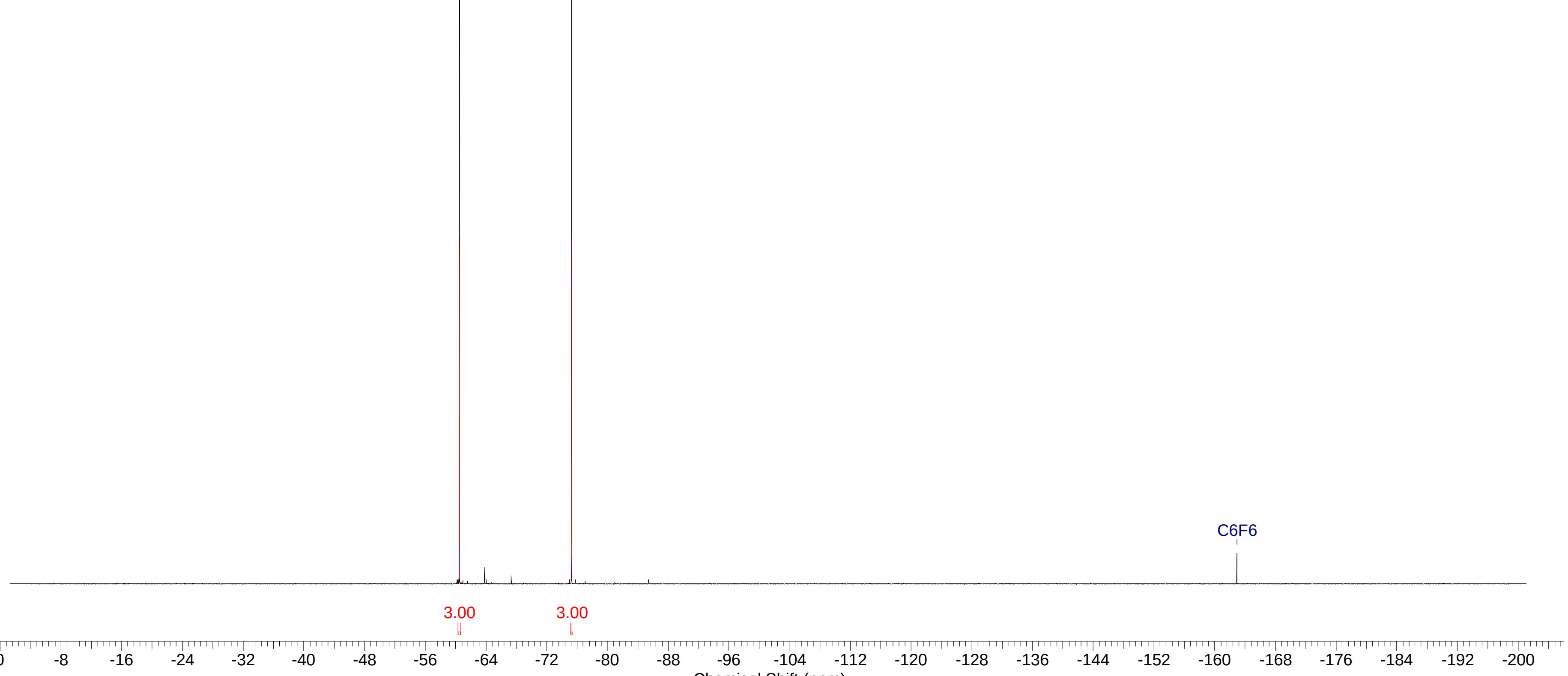

^{19}F NMR spectrum of **7** (376.5 MHz, $CDCl_3$)

19 May 2022

Acquisition Time (sec)	0.6783	***Comment***	Imported from UXNMR.			***Date***	08 Apr 2022 15:54:04
File Name	C:\DOCS\OUTPUT_301\2022\04.апрель\SZA-BM-2488-7.C_002001r					***Frequency (MHz)***	100.61
Nucleus	13C	***Number of Transients***	123	***Original Points Count***	16384	***Points Count***	131072
Pulse Sequence	zgpg30	***Solvent***	CHLOROFORM-D			***Sweep Width (Hz)***	24154.59
Temperature (degree C)	27.000						

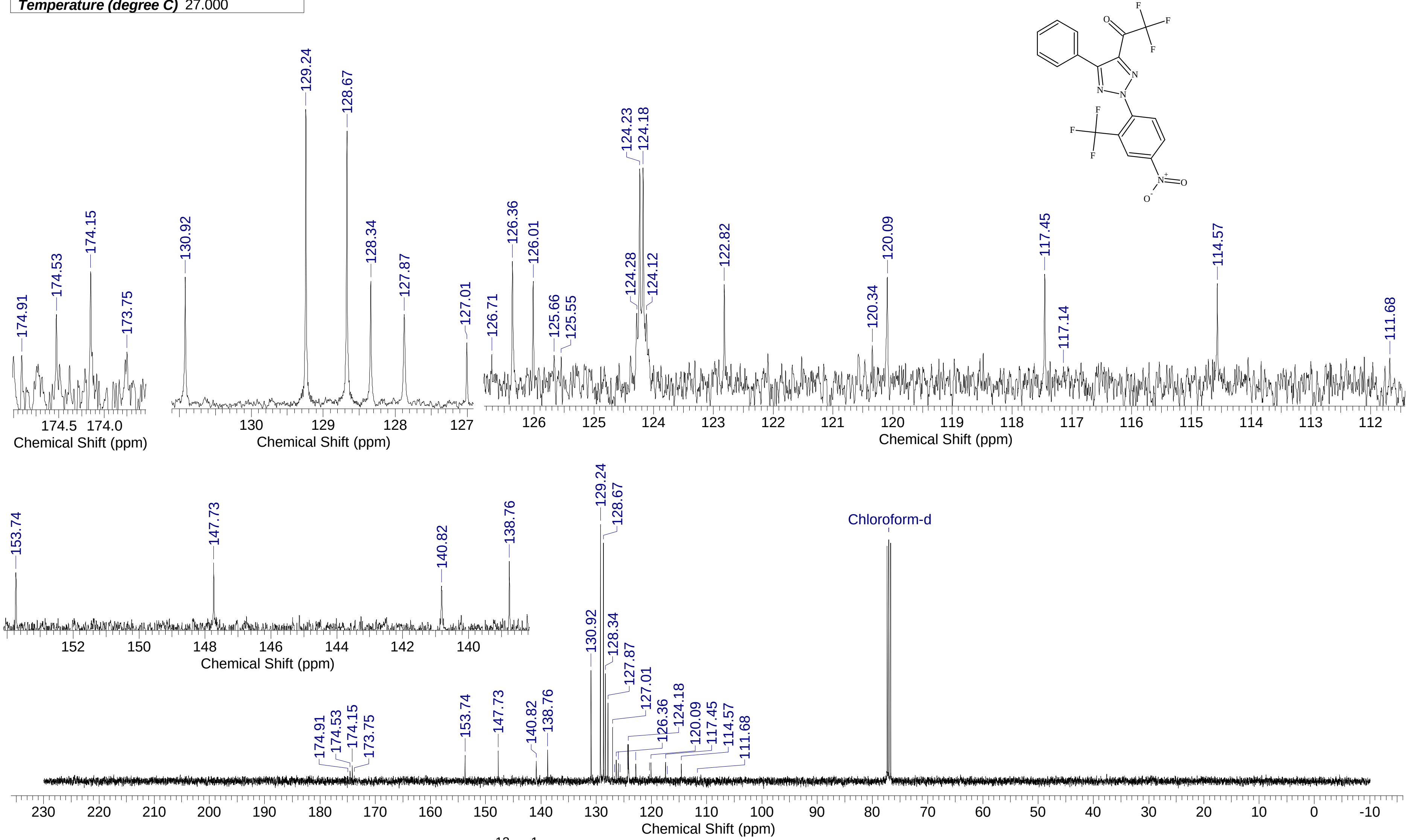

$^{13}C\{^{1}H\}$ NMR spectrum of **7** (100.6 MHz, $CDCl_3$)

19 May 2022

Acquisition Time (sec)	4.0894	***Comment***	Imported from UXNMR.			***Date***	13 Apr 2022 17:07:34
File Name	C:\DOCS\OUTPUT_301\2022\04.апрель\SZA-BM-2492-5.H_001001r					***Frequency (MHz)***	400.13
Nucleus	1H	***Number of Transients***	4	***Original Points Count***	32768	***Points Count***	131072
Pulse Sequence	zg30	***Solvent***	CHLOROFORM-D			***Sweep Width (Hz)***	8012.82
Temperature (degree C)	27.000						

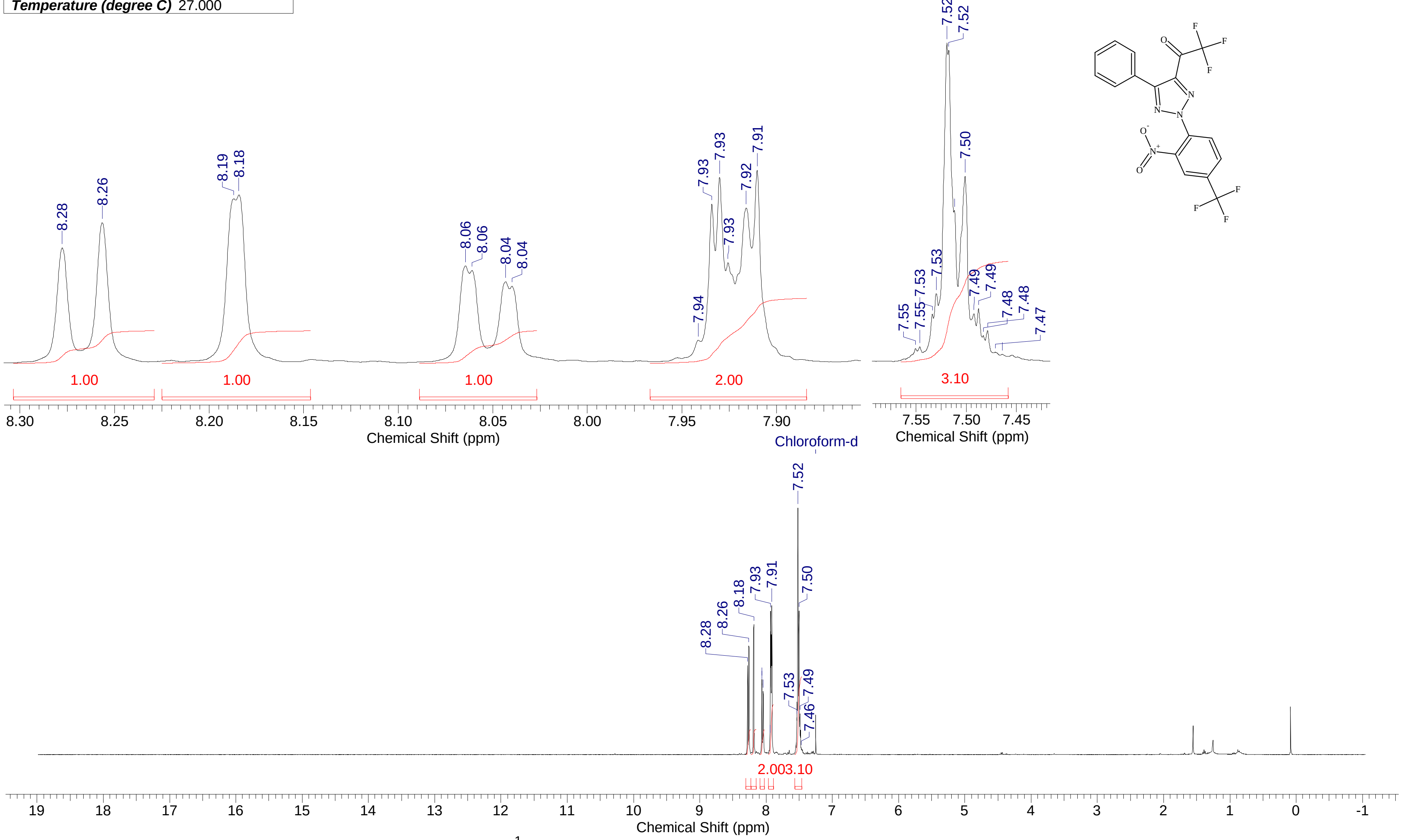

^{1}H NMR spectrum of **8** (400.1 MHz, $CDCl_3$)

19 May 2022

Acquisition Time (sec)	1.7433	***Comment***	Imported from UXNMR.			***Date***	13 Apr 2022 17:11:06
File Name	C:\DOCS\OUTPUT_301\2022\04.апрель\SZA-BM-2492-5.F_005001r					***Frequency (MHz)***	376.50
Nucleus	19F	***Number of Transients***	16	***Original Points Count***	131072	***Points Count***	262144
Pulse Sequence	zgflqn	***Solvent***	CHLOROFORM-D			***Sweep Width (Hz)***	75187.97
Temperature (degree C)	27.000						

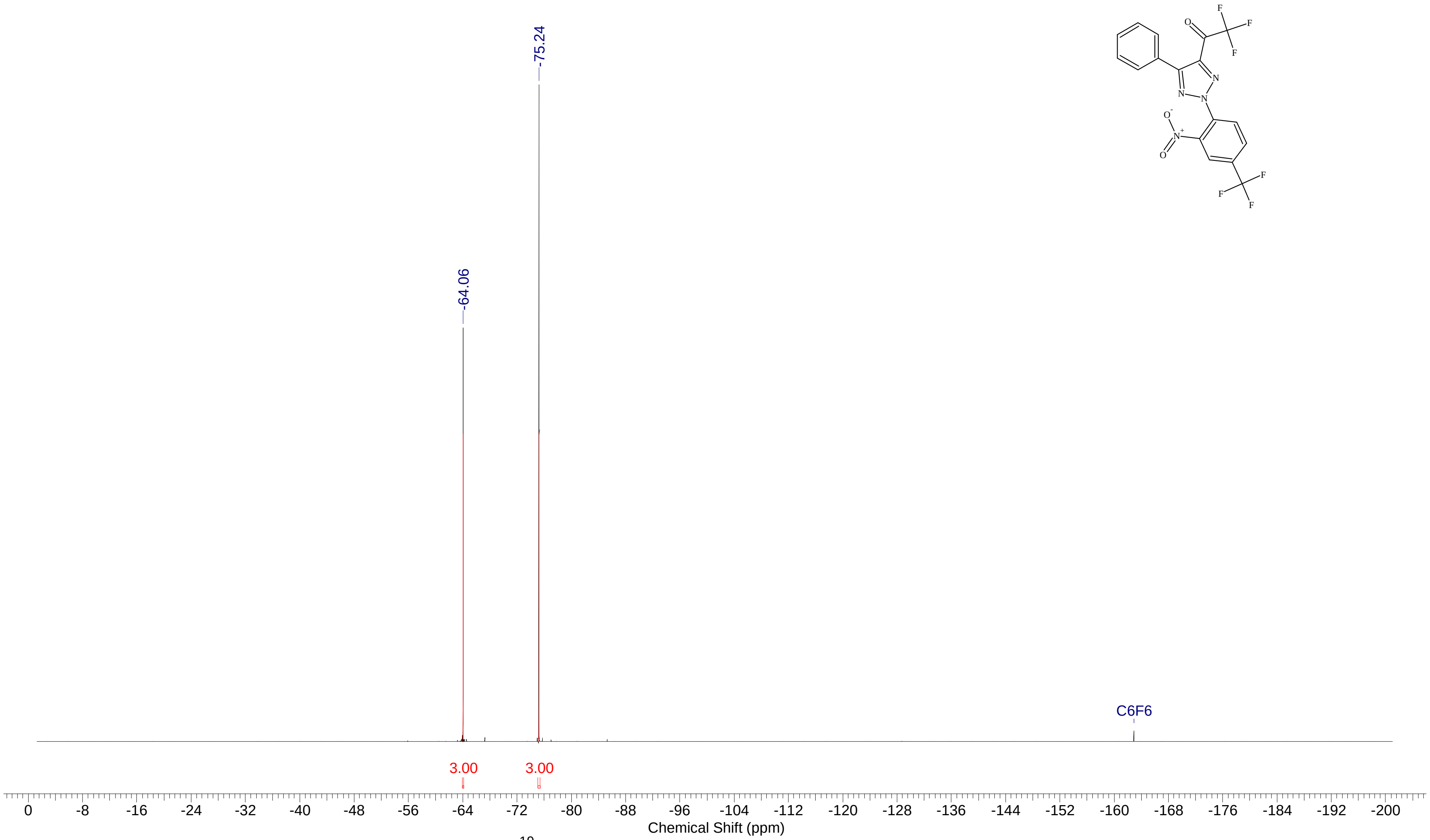

^{19}F NMR spectrum of **8** (376.5 MHz, $CDCl_3$)

19 May 2022

Acquisition Time (sec)	0.6783	***Comment***	Imported from UXNMR.	***Date***	14 Apr 2022 12:08:54
File Name	C:\DOCS\OUTPUT_301\2022\04.апрель\SZA-BM-2492-5.C_002001r			***Frequency (MHz)***	100.61
Nucleus	13C	***Number of Transients***	298	***Points Count***	131072
Pulse Sequence	zgpg30	***Solvent***	CHLOROFORM-D	***Sweep Width (Hz)***	24154.59
Temperature (degree C)	27.000				

Original Points Count 16384

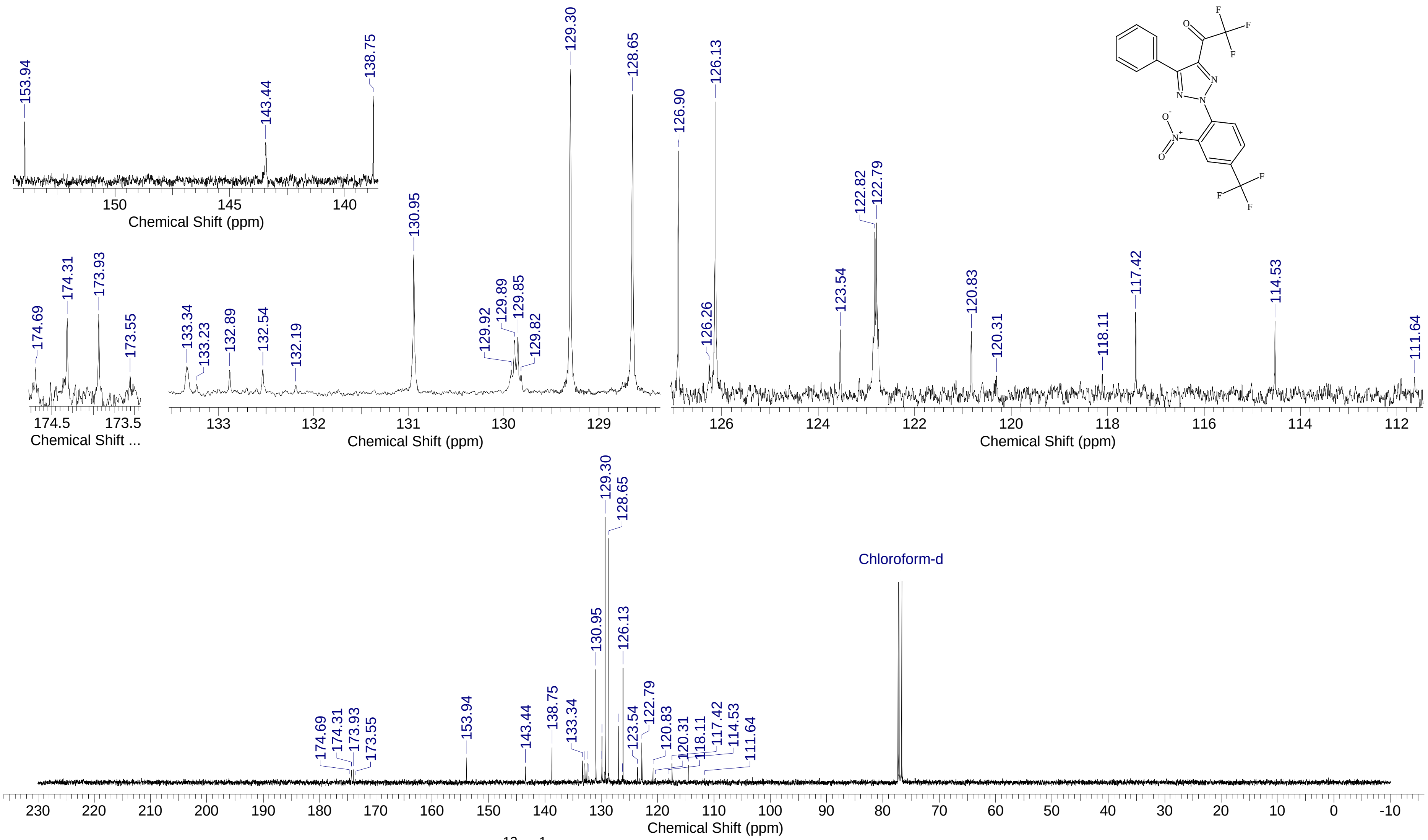

$^{13}C\{^1H\}$ NMR spectrum of **8** (100.6 MHz, $CDCl_3$)

19 May 2022

Acquisition Time (sec)	4.0894	***Comment***	Imported from UXNMR.	***Date***	18 Apr 2022 15:15:14
File Name	C:\DOCS\OUTPUT_301\2022\04.апрель\BM-2501-2.H_001001r			***Frequency (MHz)***	400.13
Nucleus	1H	***Number of Transients***	4 ***Original Points Count*** 32768	***Points Count***	131072
Pulse Sequence	zg30	***Solvent***	CHLOROFORM-D	***Sweep Width (Hz)***	8012.82
Temperature (degree C)	27.000				

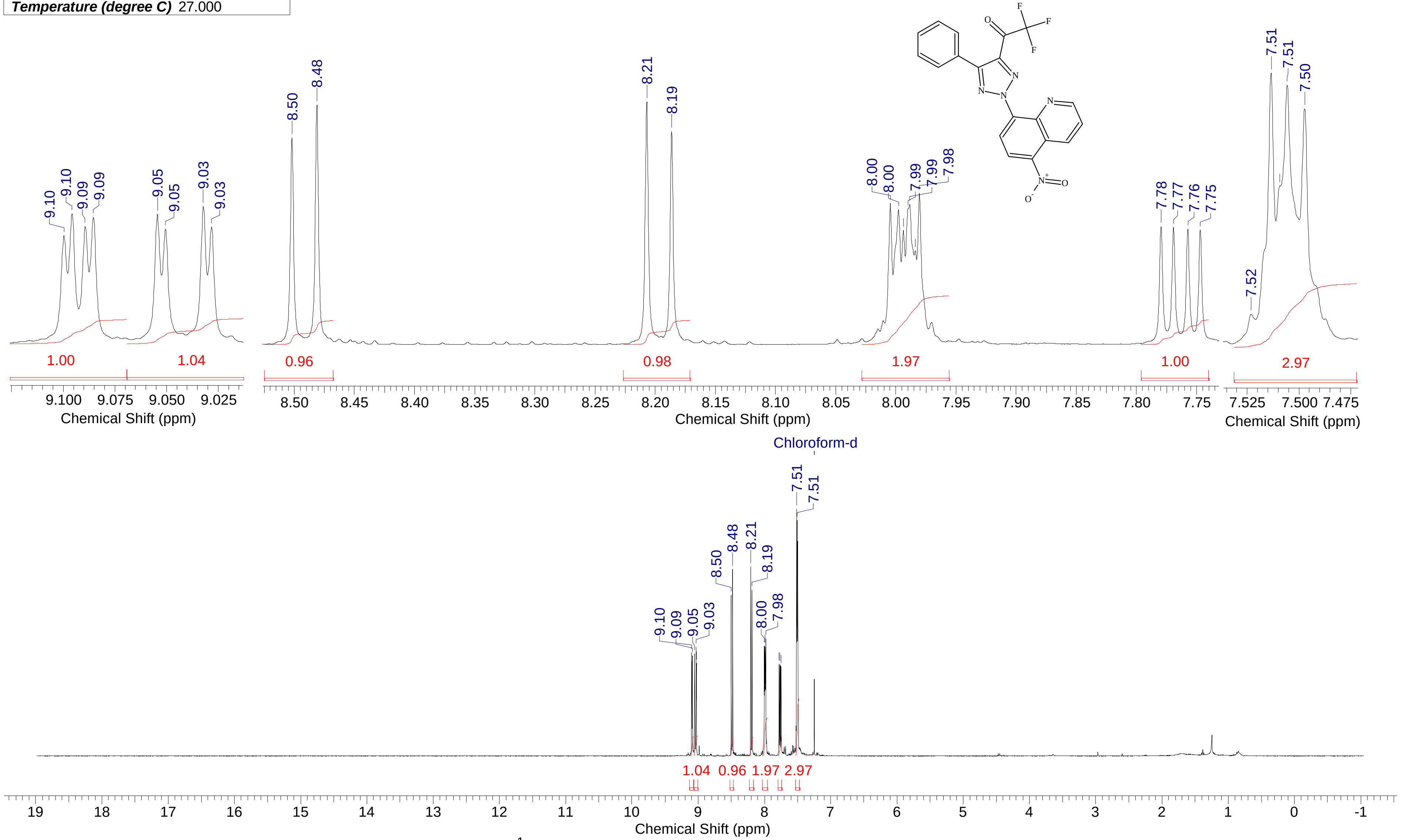

^{1}H NMR spectrum of **9** (400.1 MHz, $CDCl_3$)

19 May 2022

Acquisition Time (sec)	1.7433	Comment	Imported from UXNMR.			Date	16 Apr 2022 13:50:10
File Name	C:\DOCS\BM\ЯМР_СОСЕДИ\2022\16-04-2022_14-19-08\BM-2501-R\BM-2501-R_005001r					Frequency (MHz)	376.50
Nucleus	19F	Number of Transients	16	Original Points Count	131072	Points Count	262144
Pulse Sequence	zgflqn	Solvent	CHLOROFORM-D	Sweep Width (Hz)	75187.97	Temperature (degree C)	27.000

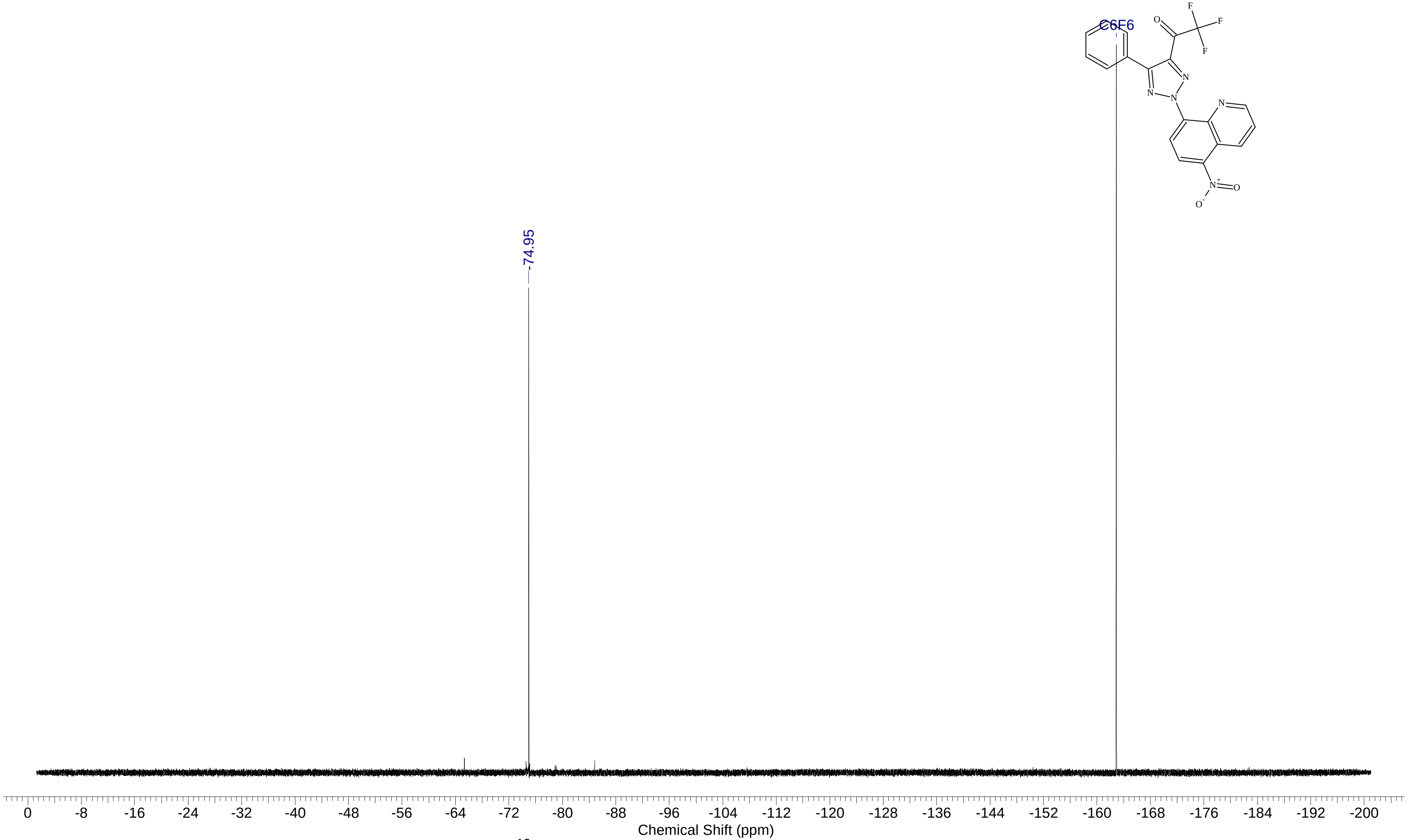

^{19}F NMR spectrum of **9** (376.5 MHz, $CDCl_3$)

19 May 2022

Acquisition Time (sec)	0.6783	***Comment***	Imported from UXNMR.			***Date***	19 Apr 2022 15:40:42
File Name	H:\ЯMP\19.04.2022\BM-2501-2.C_002001r			***Frequency (MHz)***	100.61	***Nucleus***	13C
Original Points Count	16384	***Points Count***	131072	***Pulse Sequence***	zgpg30	***Solvent***	CHLOROFORM-D
Sweep Width (Hz)	24154.59	***Temperature (degree C)***	27.000				

Number of Transients 314

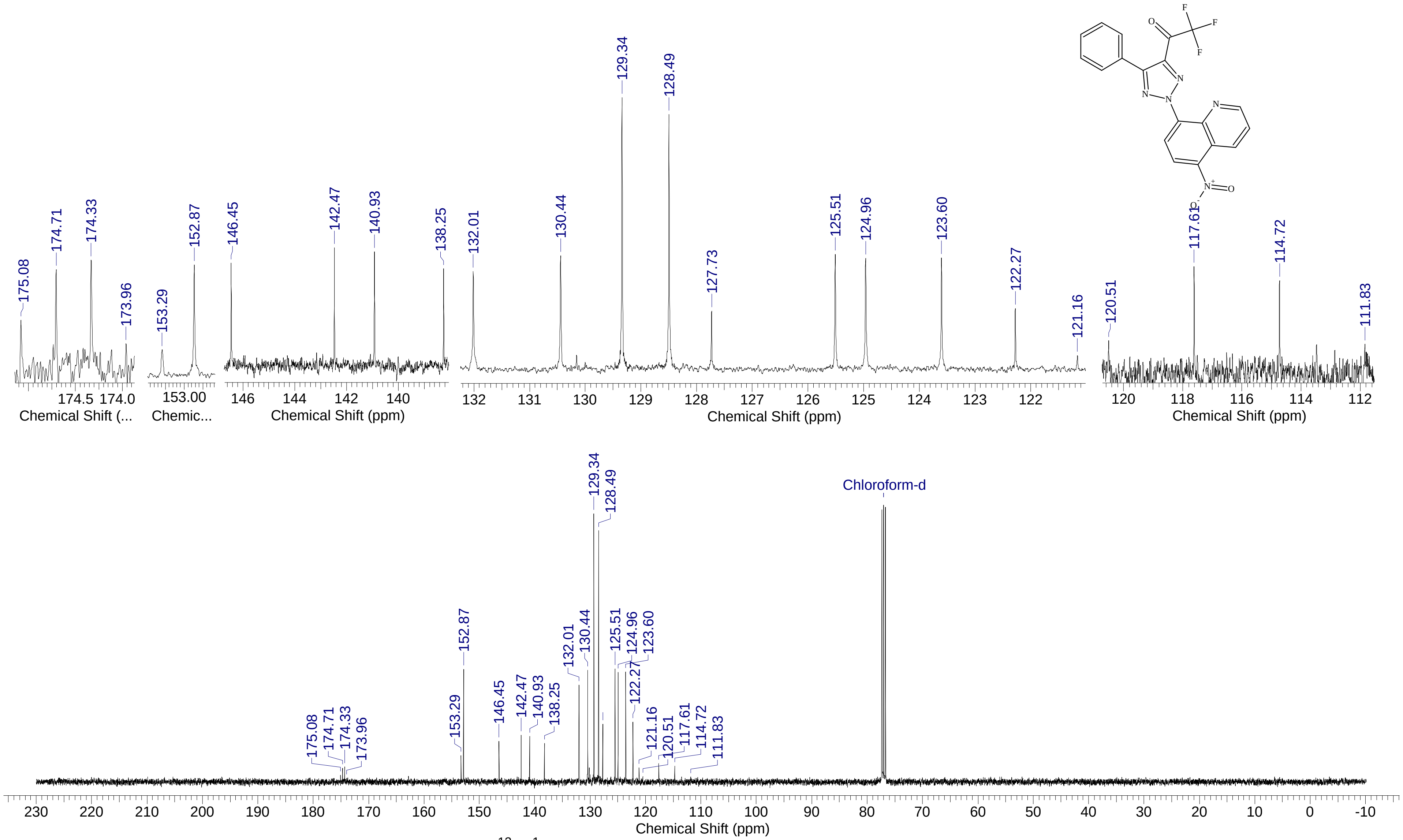

$^{13}C\{^1H\}$ NMR spectrum of **9** (100.6 MHz, $CDCl_3$)

19 May 2022

Acquisition Time (sec)	4.0894	***Comment***	Imported from UXNMR.			***Date***	09 Apr 2022 13:48:12
File Name	C:\DOCS\BM\ЯМР_СОСЕДИ\2022\bm220409\BM-2489-2_001001r					***Frequency (MHz)***	400.13
Nucleus	1H	***Number of Transients***	8	***Original Points Count***	32768	***Points Count***	131072
Pulse Sequence	zg30	***Solvent***	CHLOROFORM-D			***Sweep Width (Hz)***	8012.82
Temperature (degree C)	27.000						

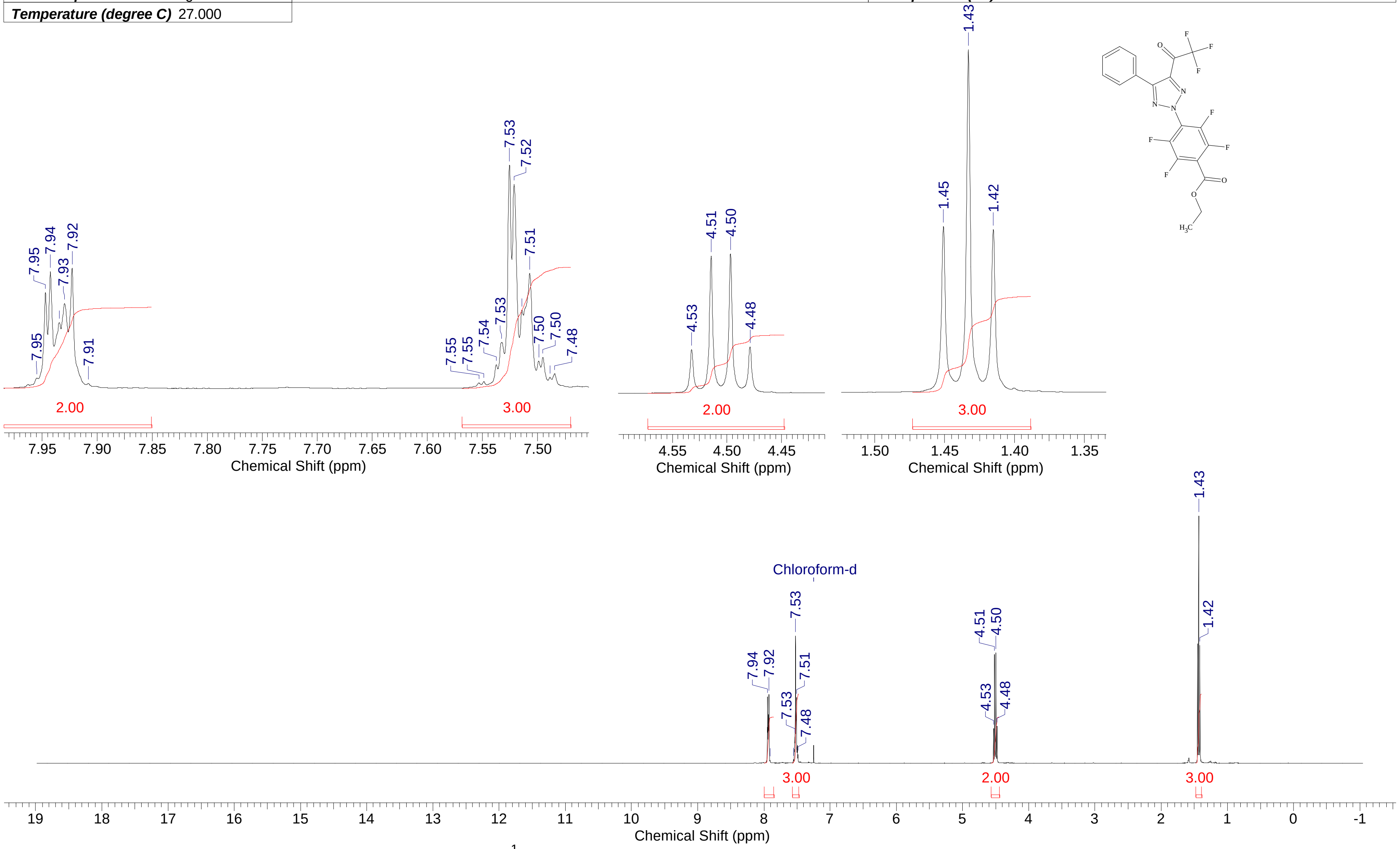

^{1}H NMR spectrum of **10** (400.1 MHz, $CDCl_3$)

19 May 2022

Acquisition Time (sec)	1.7433	***Comment***	Imported from UXNMR.	***Date***	09 Apr 2022 13:59:12
File Name	C:\DOCS\BM\ЯМР_СОСЕДИ\2022\bm220409\BM-2489-2_005001r			***Frequency (MHz)***	376.50
Nucleus	19F	***Number of Transients***	16 ***Original Points Count*** 131072	***Points Count***	262144
Pulse Sequence	zgflqn	***Solvent***	CHLOROFORM-D	***Sweep Width (Hz)***	75187.97
Temperature (degree C)	27.000				

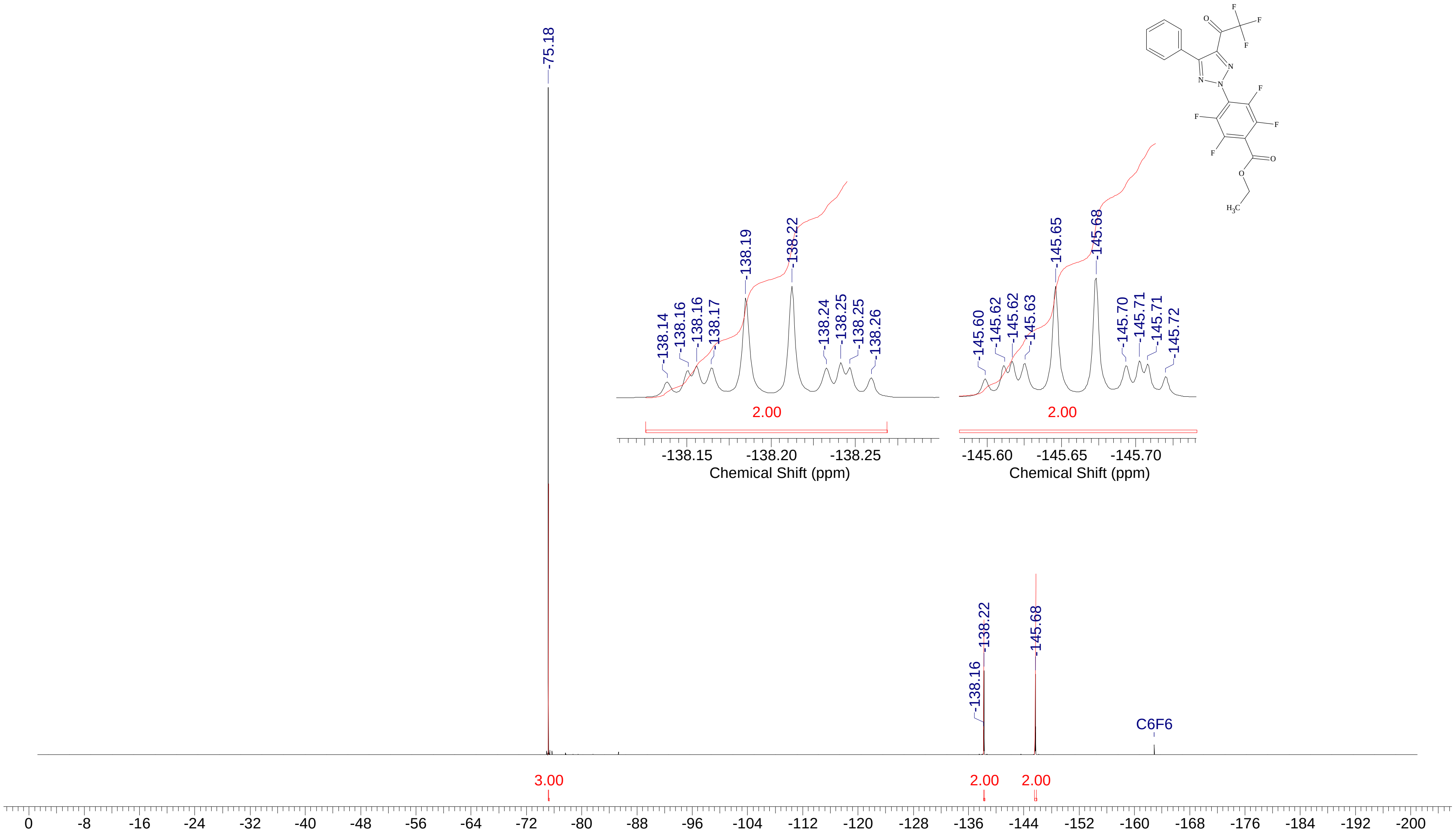

^{19}F NMR spectrum of **10** (376.5 MHz, $CDCl_3$)

19 May 2022

Acquisition Time (sec)	0.6783	Comment	Imported from UXNMR.			Date	12 Apr 2022 14:16:50
File Name	C:\DOCS\OUTPUT_301\2022\04.апрель\BM-2489-2.C_002001r					Frequency (MHz)	100.61
Nucleus	13C	Number of Transients	922	Original Points Count	16384	Points Count	131072
Pulse Sequence	zgpg30	Solvent	CHLOROFORM-D			Sweep Width (Hz)	24154.59
Temperature (degree C)	27.000						

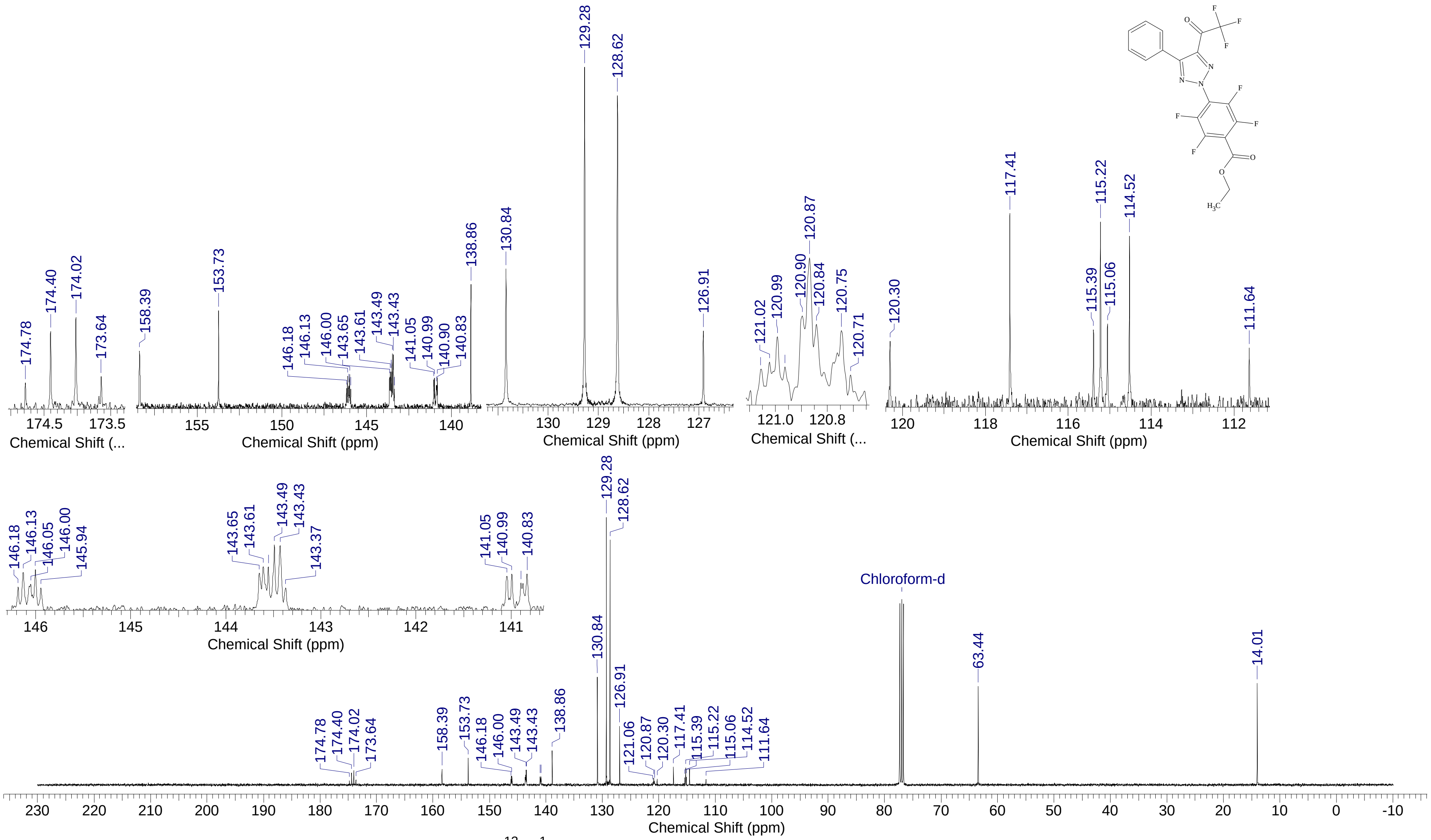

$^{13}C\{^{1}H\}$ NMR spectrum of **10** (100.6 MHz, $CDCl_3$)

25 Nov 2022

Acquisition Time (sec)	4.0894	***Comment***	Imported from UXNMR.	***Date***	31 Oct 2022 15:59:38
File Name	C:\DOCS\OUTPUT_301\2022\10.октябрь\SZA-BM-2672.H_001001r			***Frequency (MHz)***	400.13
Nucleus	1H	***Number of Transients***	4 ***Original Points Count*** 32768	***Points Count***	131072
Pulse Sequence	zg30	***Solvent***	CHLOROFORM-D	***Sweep Width (Hz)***	8012.82
Temperature (degree C)	27.000				

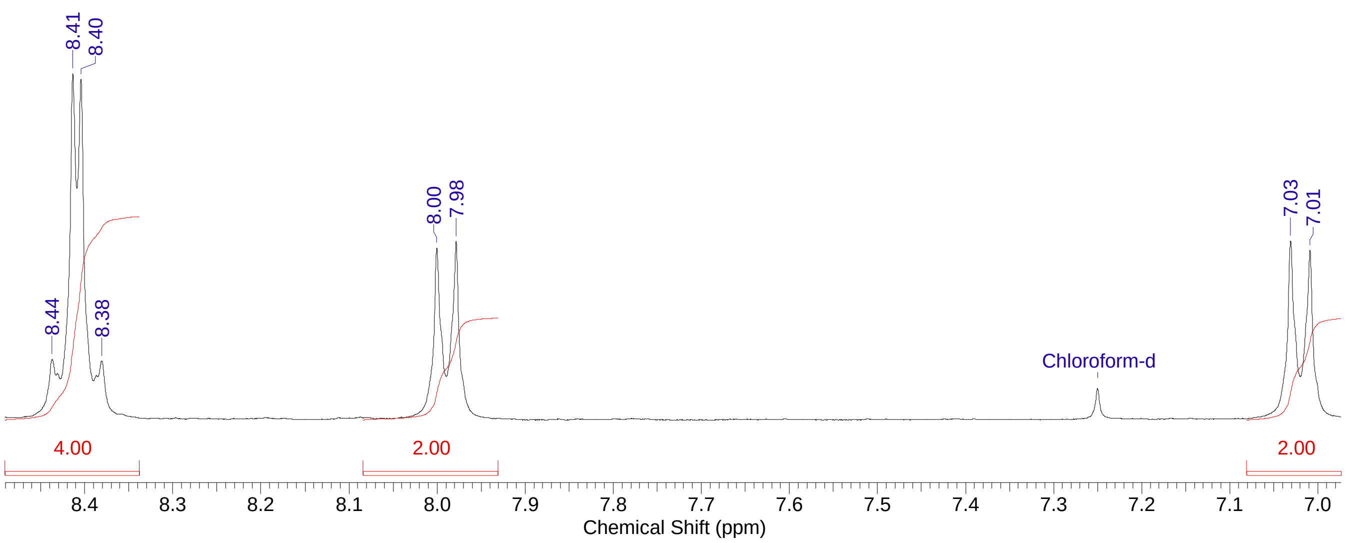

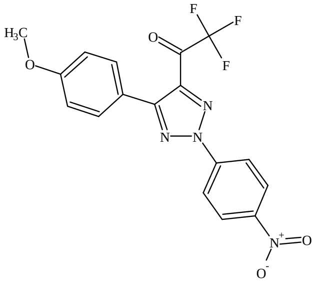

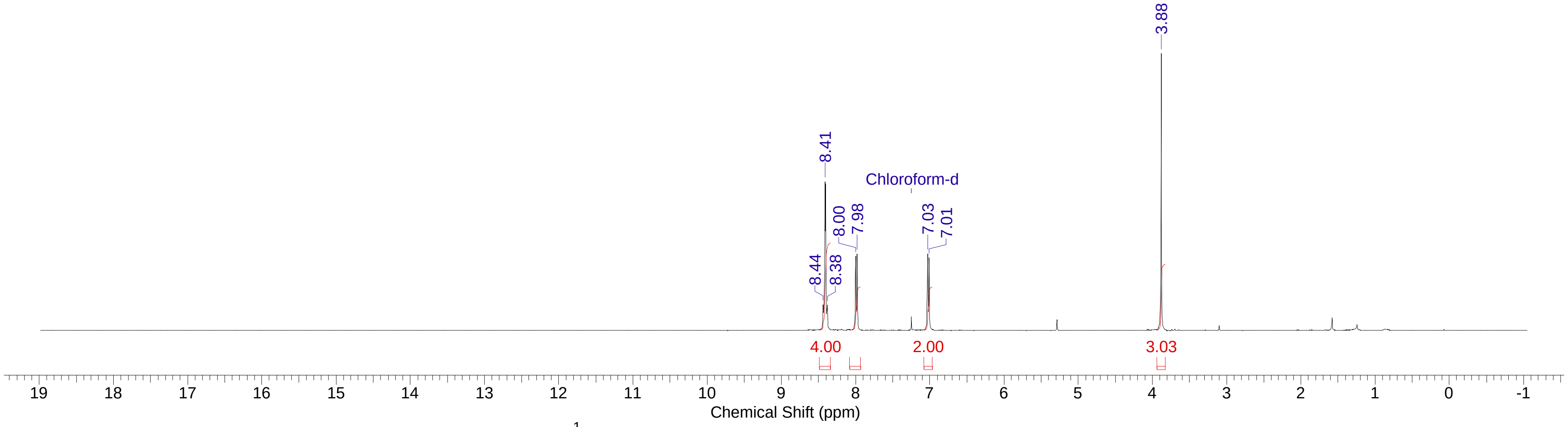

^{1}H NMR spectrum of **11** (400.1 MHz, $CDCl_3$)

**_Acquisition Time (sec)_**	1.7433	**_Comment_**	Imported from UXNMR.			**_Date_**	31 Oct 2022 15:49:56
**_File Name_**	C:\DOCS\OUTPUT_301\2022\10.октябрь\SZA-BM-2672.F_005001r					**_Frequency (MHz)_**	376.50
**_Nucleus_**	19F	**_Number of Transients_**	16	**_Original Points Count_**	131072	**_Points Count_**	262144
**_Pulse Sequence_**	zgflqn	**_Solvent_**	CHLOROFORM-D			**_Sweep Width (Hz)_**	75187.97
**_Temperature (degree C)_**	27.000						

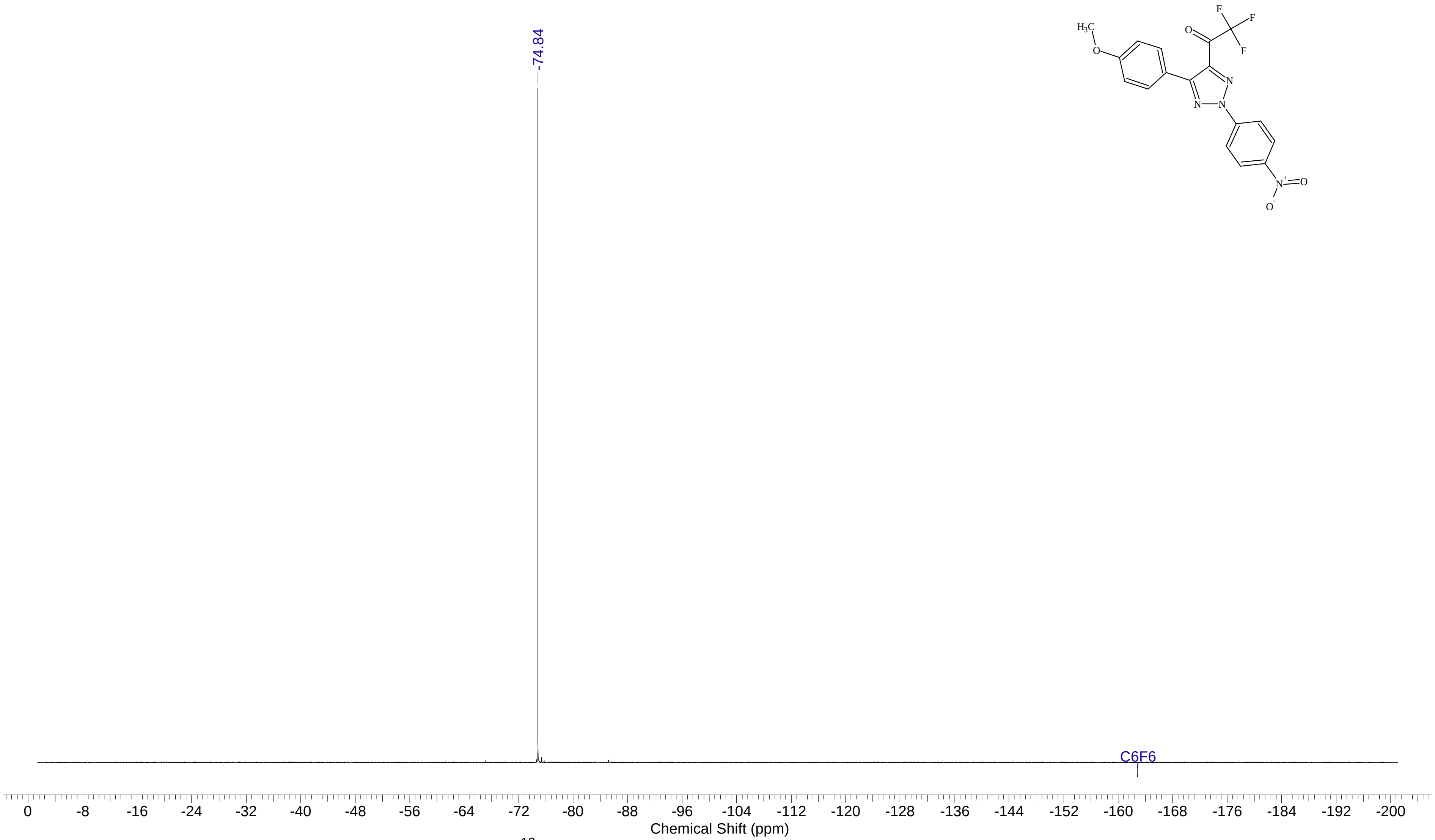

^{19}F NMR spectrum of **11** (376.5 MHz, $CDCl_3$)

25 Nov 2022

Acquisition Time (sec)	0.6783	***Comment***	Imported from UXNMR.			***Date***	01 Nov 2022 14:13:08
File Name	C:\DOCS\OUTPUT_301\2022\11.ноябрь\SZA-BM-2672.C_002001r					***Frequency (MHz)***	100.61
Nucleus	13C	***Number of Transients***	356	***Original Points Count***	16384	***Points Count***	131072
Pulse Sequence	zgpg30	***Solvent***	CHLOROFORM-D			***Sweep Width (Hz)***	24154.59
Temperature (degree C)	27.000						

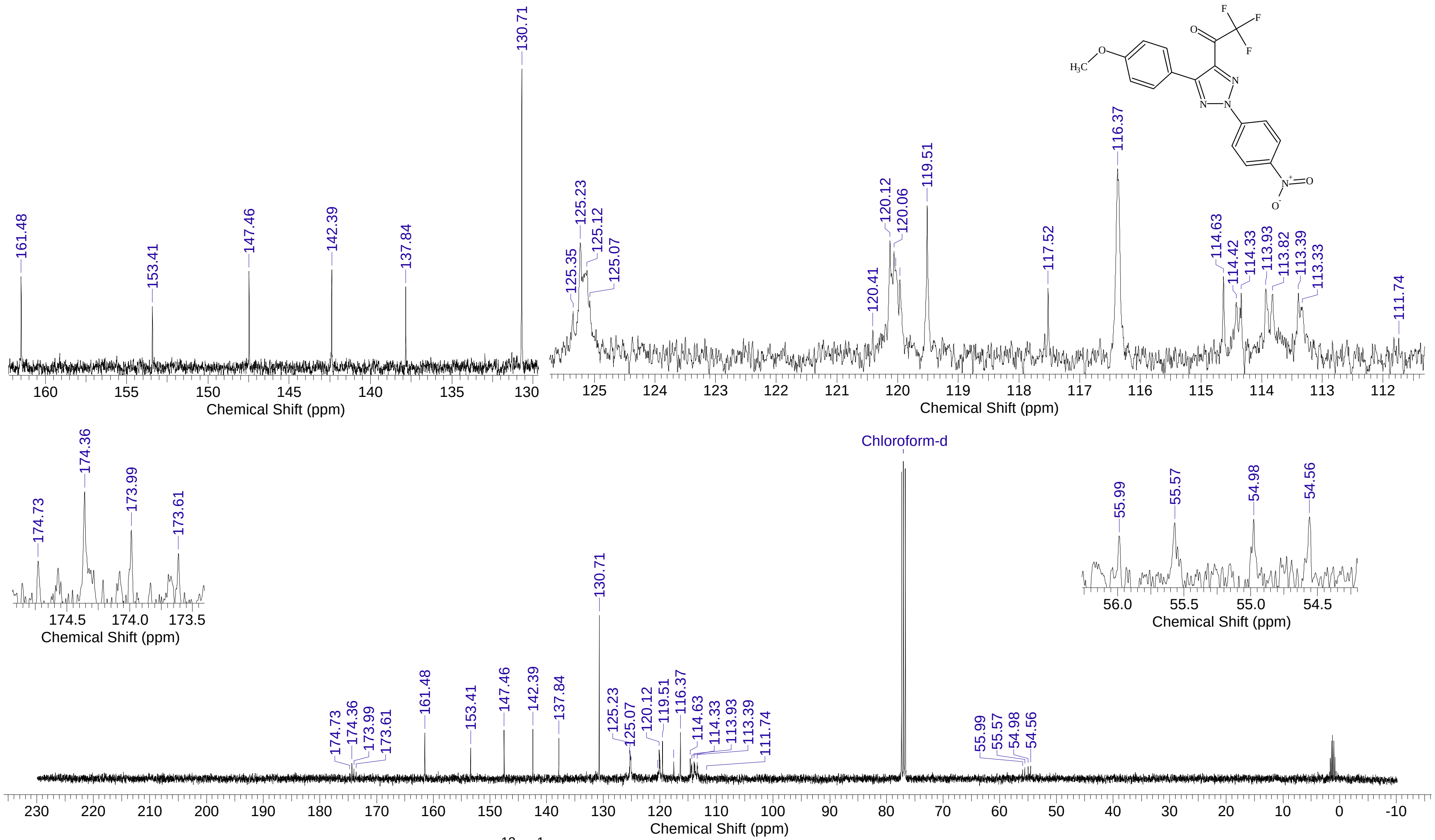

$^{13}C\{^1H\}$ NMR spectrum of **11** (100.6 MHz, $CDCl_3$)

28 Mar 2023

Acquisition Time (sec)	4.0894	Comment	Imported from UXNMR.			Date	13 Dec 2022 15:36:00
File Name	C:\DOCS\OUTPUT_301\2022\12.декабрь\BM-2697-2.H_001001r					Frequency (MHz)	400.13
Nucleus	1H	Number of Transients	4	Original Points Count	32768	Points Count	131072
Pulse Sequence	zg30	Solvent	CHLOROFORM-D			Sweep Width (Hz)	8012.82
Temperature (degree C)	27.000						

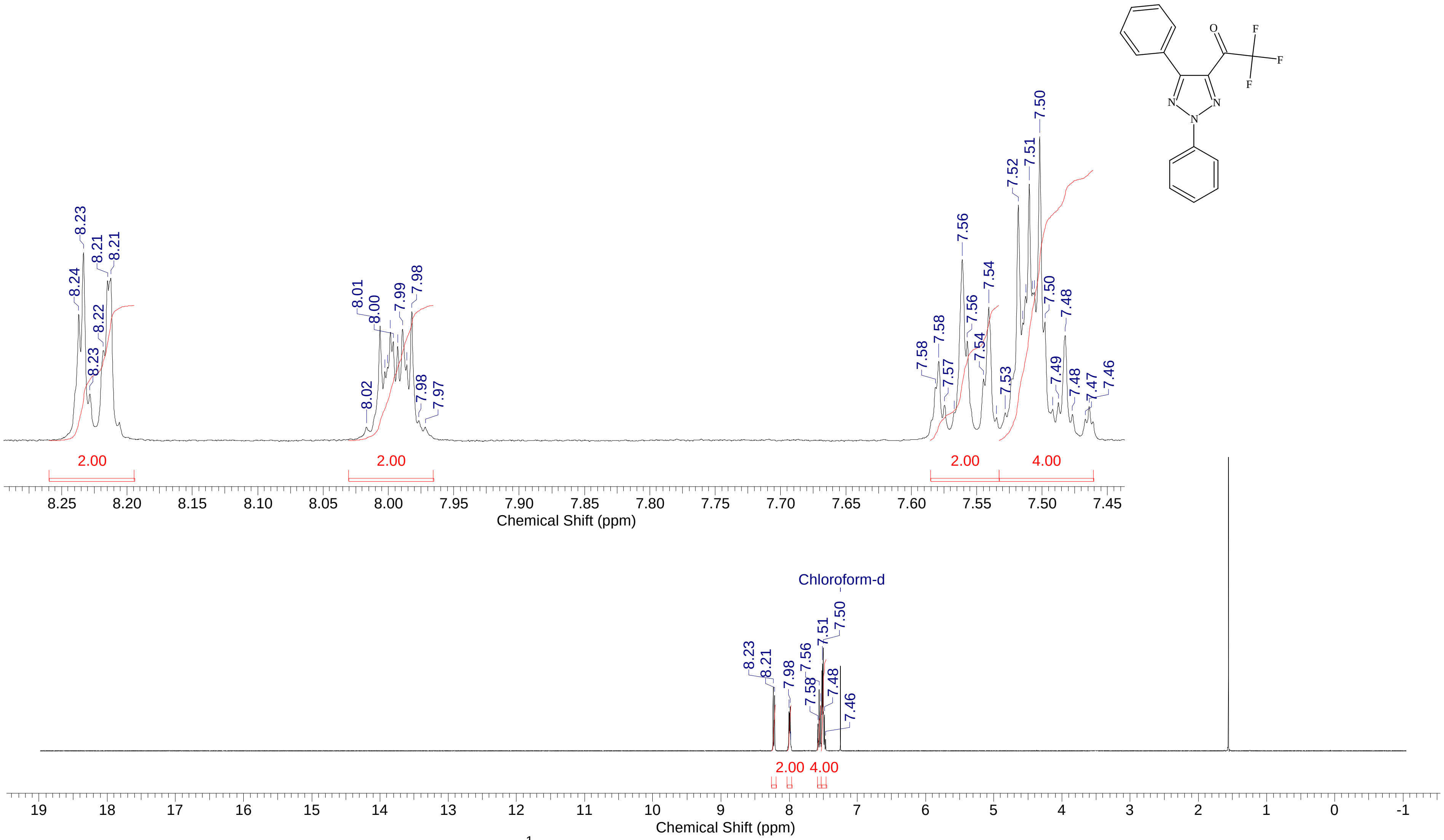

^{1}H NMR spectrum of **12** (400.1 MHz, $CDCl_3$)

28 Mar 2023

Acquisition Time (sec)	1.7433	***Comment***	Imported from UXNMR.			***Date***	13 Dec 2022 15:37:54
File Name	C:\DOCS\OUTPUT_301\2022\12.декабрь\BM-2697-2.F_005001r					***Frequency (MHz)***	376.50
Nucleus	19F	***Number of Transients***	14	***Original Points Count***	131072	***Points Count***	262144
Pulse Sequence	zgflqn	***Solvent***	CHLOROFORM-D			***Sweep Width (Hz)***	75187.97
Temperature (degree C)	27.000						

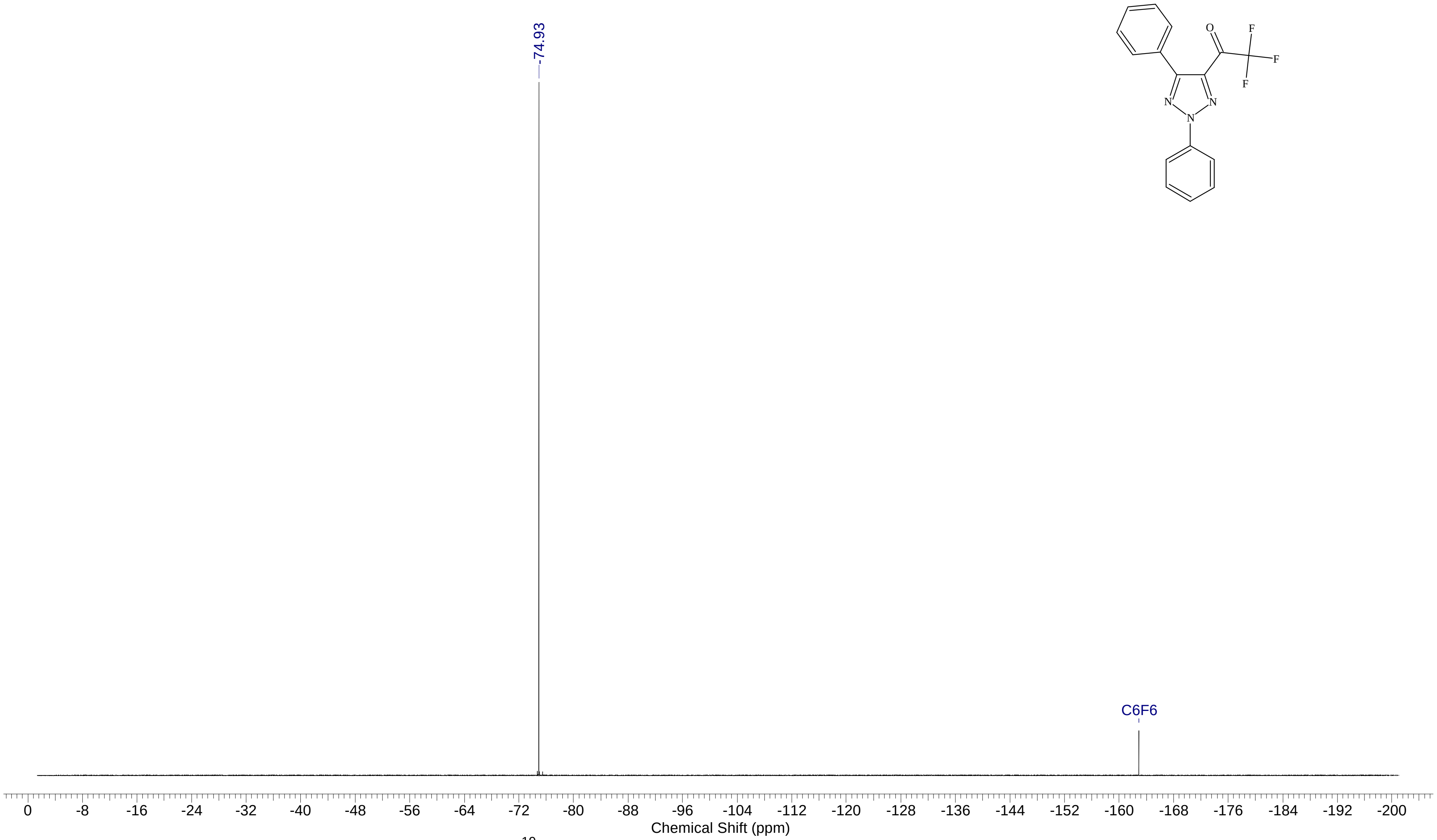

^{19}F NMR spectrum of **12** (376.5 MHz, $CDCl_3$)

19 May 2022

Acquisition Time (sec)	0.6783	***Comment***	Imported from UXNMR.			***Date***	21 Apr 2022 13:09:40
File Name	C:\DOCS\OUTPUT_301\2022\04.апрель\BM-2505-2.C_002001r					***Frequency (MHz)***	100.61
Nucleus	13C	***Number of Transients***	763	***Original Points Count***	16384	***Points Count***	131072
Pulse Sequence	zgpg30	***Solvent***	CHLOROFORM-D			***Sweep Width (Hz)***	24154.59
Temperature (degree C)	27.000						

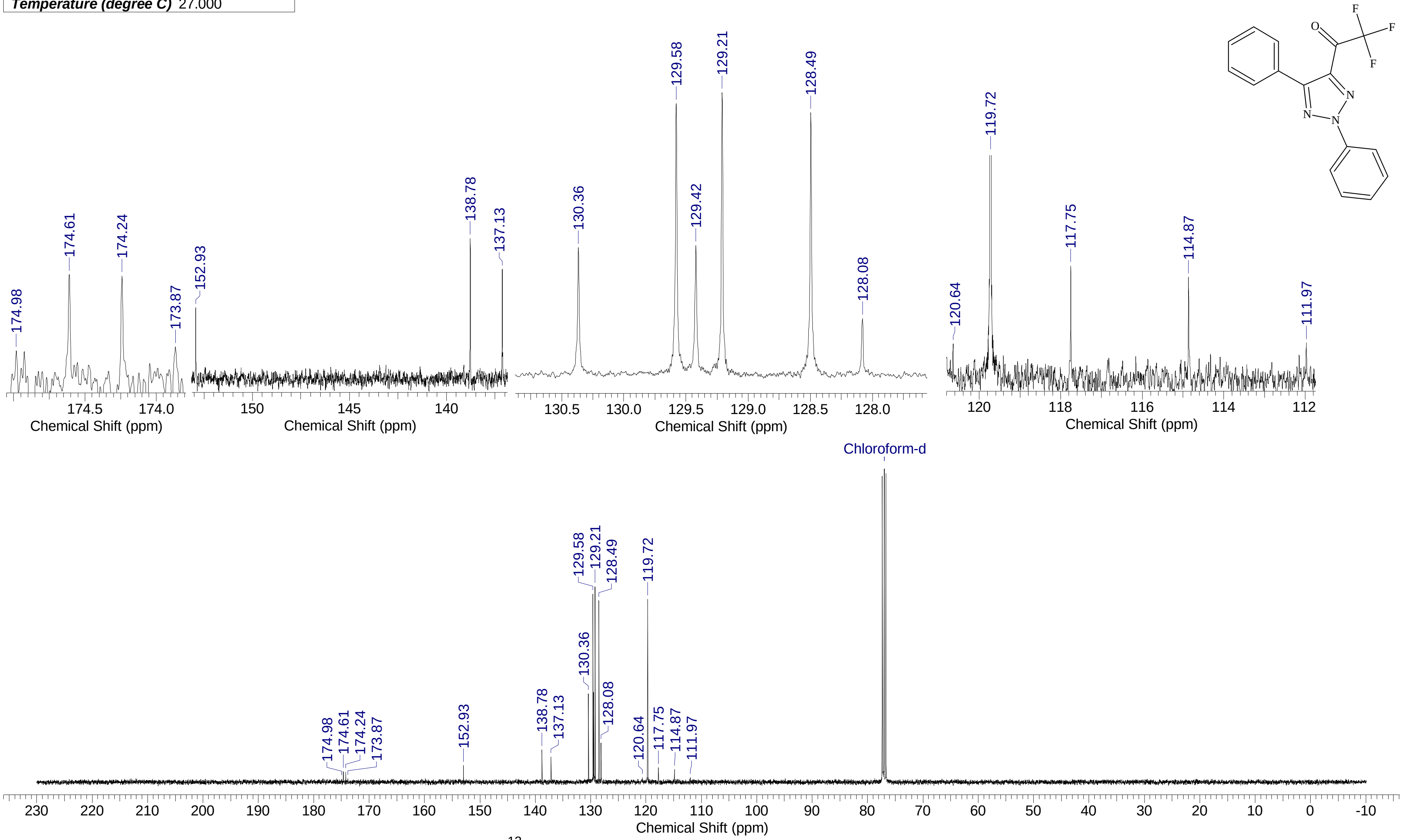

^{13}C NMR spectrum of **12** (100.6 MHz, $CDCl_3$)

25 Nov 2022

Acquisition Time (sec)	4.0894	***Comment***	Imported from UXNMR.			***Date***	15 Oct 2022 22:41:10
File Name	C:\DOCS\BM\ЯМР_СОСЕДИ\2022\221015\BM-2650_001001r					***Frequency (MHz)***	400.13
Nucleus	1H	***Number of Transients***	8	***Original Points Count***	32768	***Points Count***	131072
Pulse Sequence	zg30	***Solvent***	CHLOROFORM-D			***Sweep Width (Hz)***	8012.82
Temperature (degree C)	27.000						

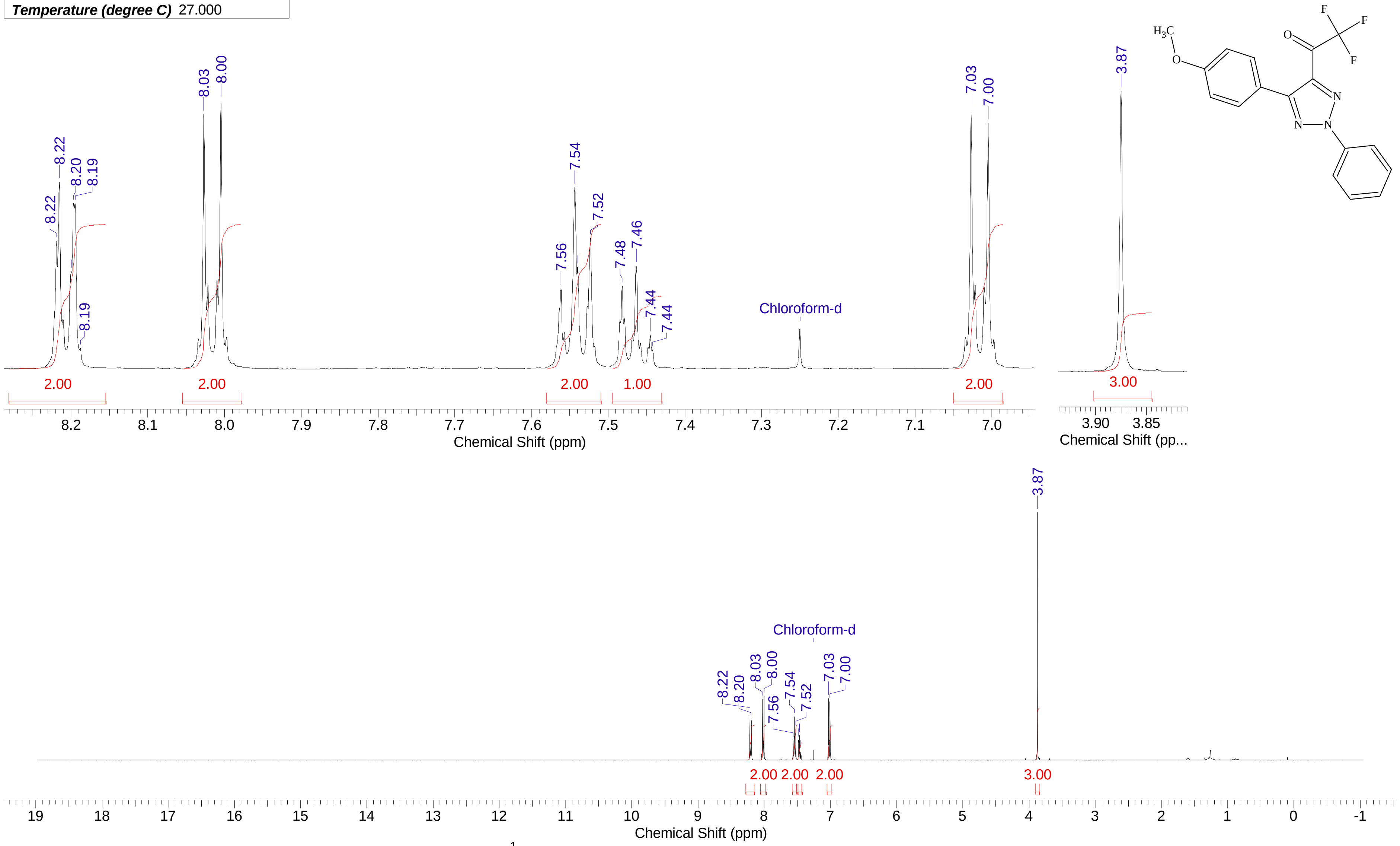

^{1}H NMR spectrum of **13** (400.1 MHz, $CDCl_3$)

Acquisition Time (sec)	1.7433	***Comment***	Imported from UXNMR.			***Date***	15 Oct 2022 22:44:04
File Name	C:\DOCS\BM\ЯМР_СОСЕДИ\2022\221015\BM-2650_005001r					***Frequency (MHz)***	376.50
Nucleus	19F	***Number of Transients***	16	***Original Points Count***	131072	***Points Count***	262144
Pulse Sequence	zgflqn	***Solvent***	CHLOROFORM-D			***Sweep Width (Hz)***	75187.97
Temperature (degree C)	27.000						

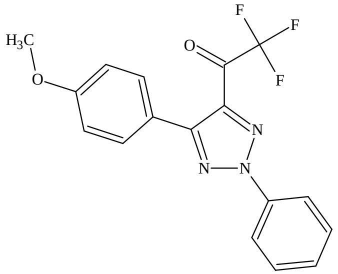

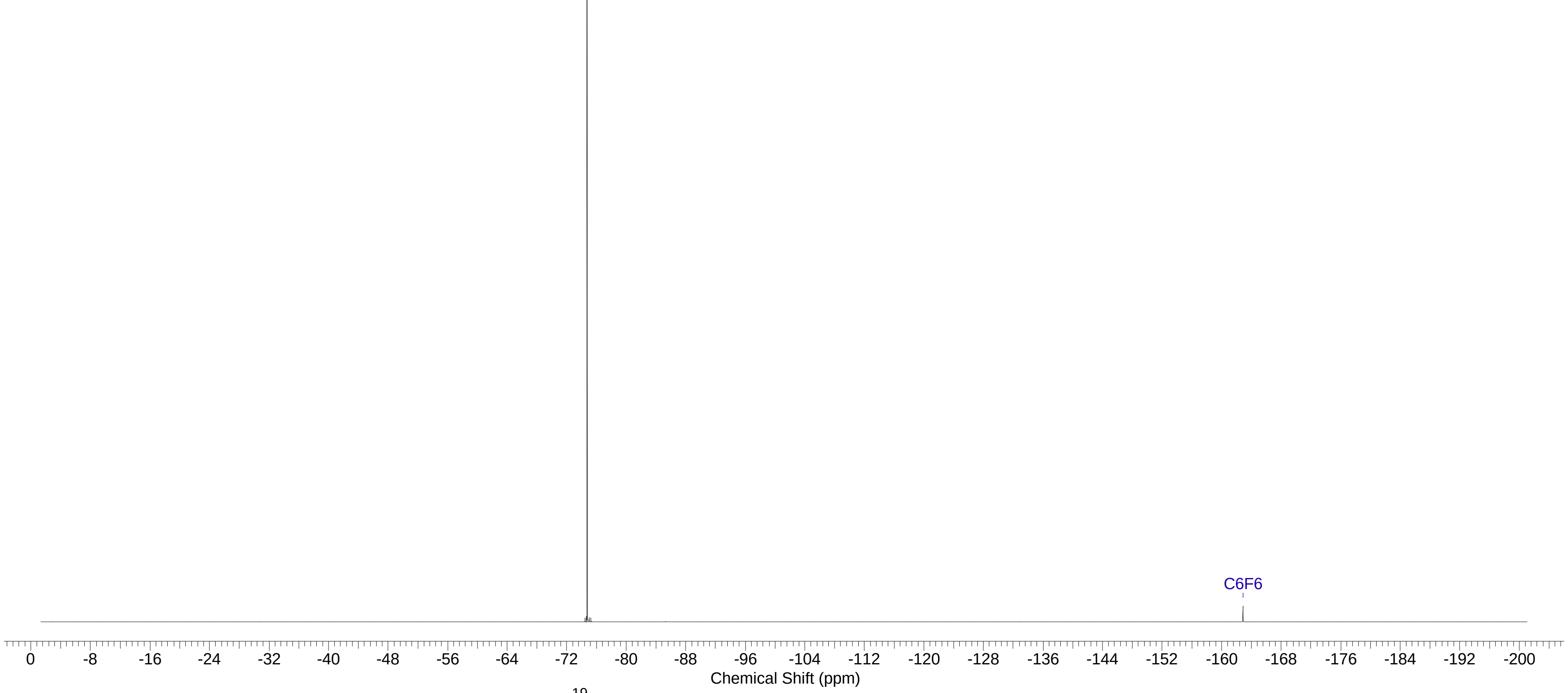

^{19}F NMR spectrum of **13** (376.5 MHz, $CDCl_3$)

25 Nov 2022

Acquisition Time (sec)	0.6783	Comment	Imported from UXNMR.			Date	18 Oct 2022 14:48:06
File Name	C:\DOCS\OUTPUT_301\2022\10.октябрь\BM-2650.C_002001r					Frequency (MHz)	100.61
Nucleus	13C	Number of Transients	273	Original Points Count	16384	Points Count	131072
Pulse Sequence	zgpg30	Solvent	CHLOROFORM-D			Sweep Width (Hz)	24154.59
Temperature (degree C)	27.000						

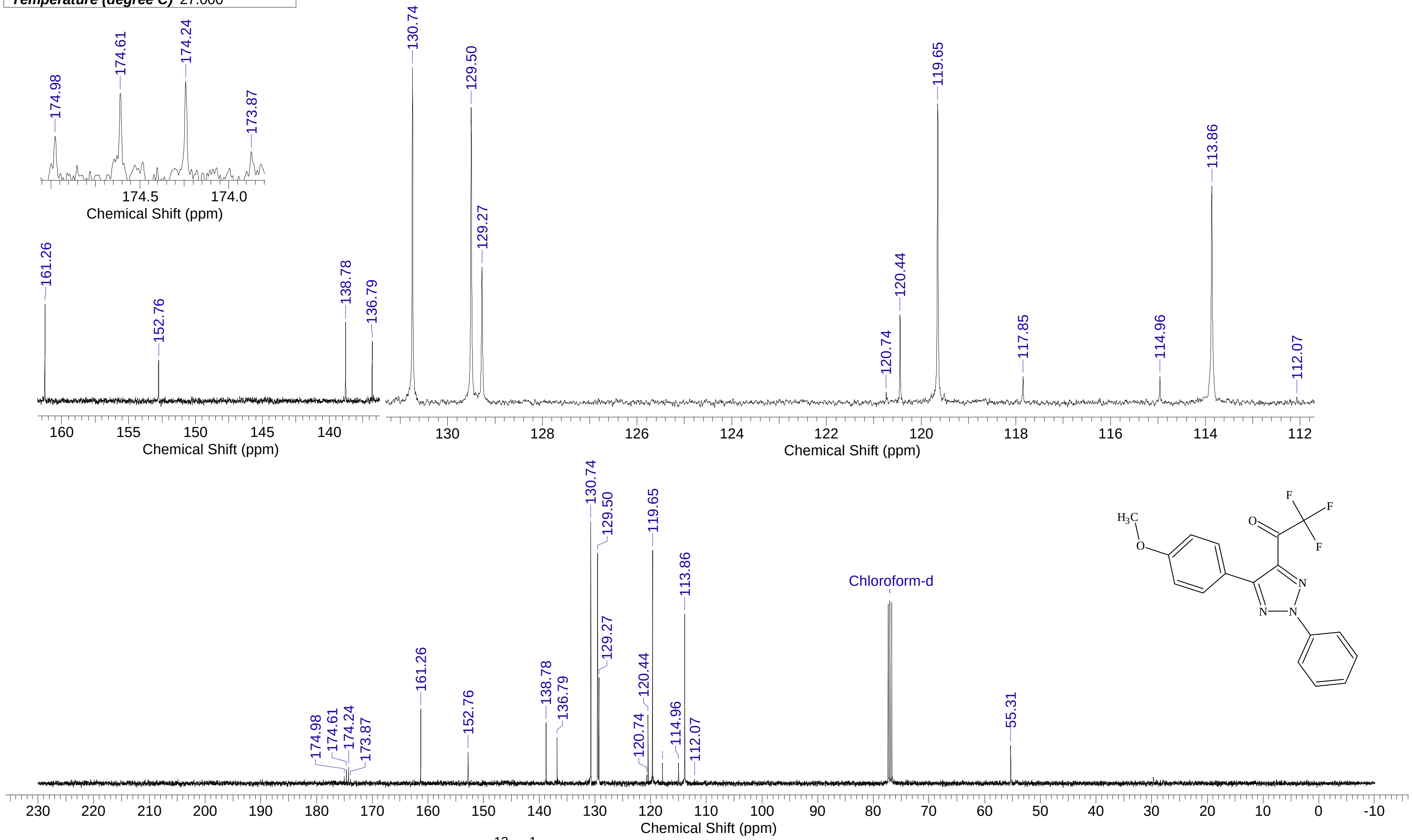

$^{13}C\{^{1}H\}$ NMR spectrum of **13** (100.6 MHz, $CDCl_3$)

25 Nov 2022

Acquisition Time (sec) 4.0894	***Comment***	Imported from UXNMR.		***Date***	14 Nov 2022 15:31:06
File Name	C:\BM_DATA\DOCS\SZA-BM-2676-5\SZA-BM-2676-5\SZA-BM-2676-5.H_001001r			***Frequency (MHz)***	400.13
Nucleus 1H	***Number of Transients***	4	***Original Points Count*** 32768	***Points Count***	131072
Pulse Sequence zg30	***Solvent***	CHLOROFORM-D		***Sweep Width (Hz)***	8012.82
Temperature (degree C) 27.000					

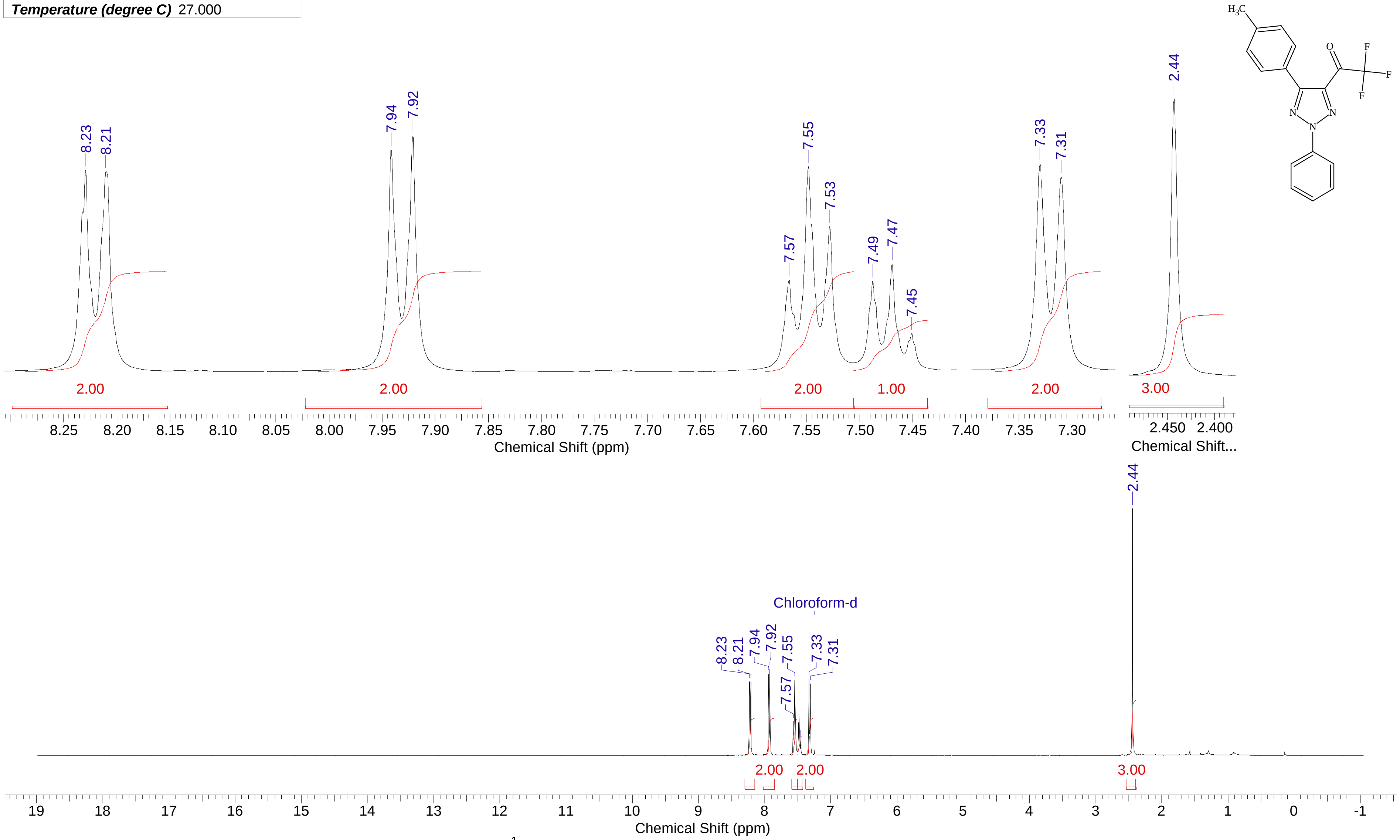

^{1}H NMR spectrum of **14** (400.1 MHz, $CDCl_3$)

Acquisition Time (sec)	1.7433	***Comment***	Imported from UXNMR.			***Date***	14 Nov 2022 15:44:06
File Name	C:\BM_DATA\DOCS\SZA-BM-2676-5\SZA-BM-2676-5\SZA-BM-2676-5.F_005001r					***Frequency (MHz)***	376.50
Nucleus	19F	***Number of Transients***	16	***Original Points Count***	131072	***Points Count***	262144
Pulse Sequence	zgflqn	***Solvent***	CHLOROFORM-D			***Sweep Width (Hz)***	75187.97
Temperature (degree C)	27.000						

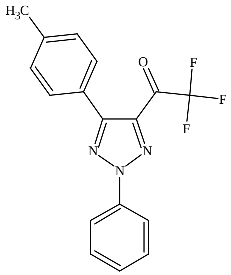

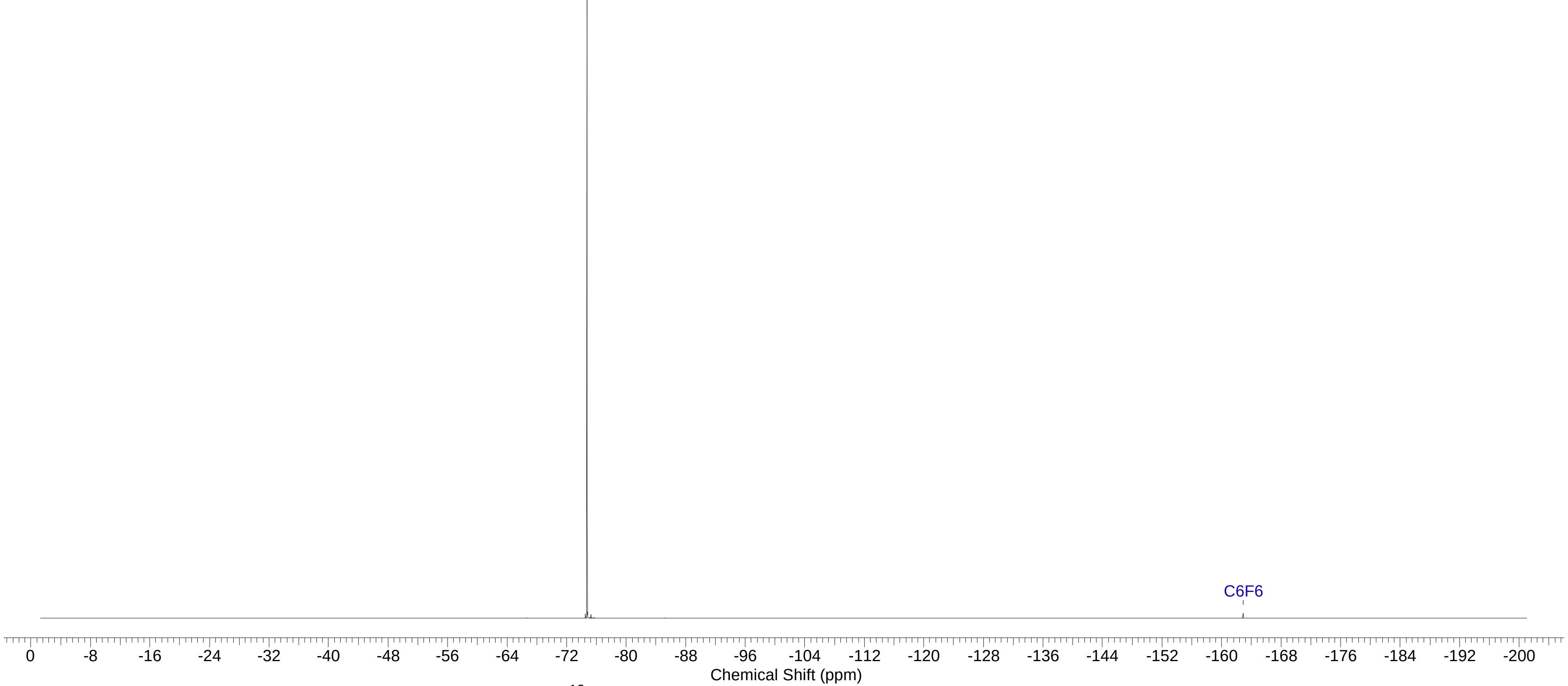

^{19}F NMR spectrum of **14** (376.5 MHz, $CDCl_3$)

25 Nov 2022

Acquisition Time (sec)	0.6783	***Comment***	Imported from UXNMR.			***Date***	14 Nov 2022 15:40:02
File Name	C:\BM_DATA\DOCS\SZA-BM-2676-5\SZA-BM-2676-5\SZA-BM-2676-5.C_002001r					***Frequency (MHz)***	100.61
Nucleus	13C	***Number of Transients***	180	***Original Points Count***	16384	***Points Count***	131072
Pulse Sequence	zgpg30	***Solvent***	DEUTERIUM OXIDE			***Sweep Width (Hz)***	24154.59
Temperature (degree C)	27.000						

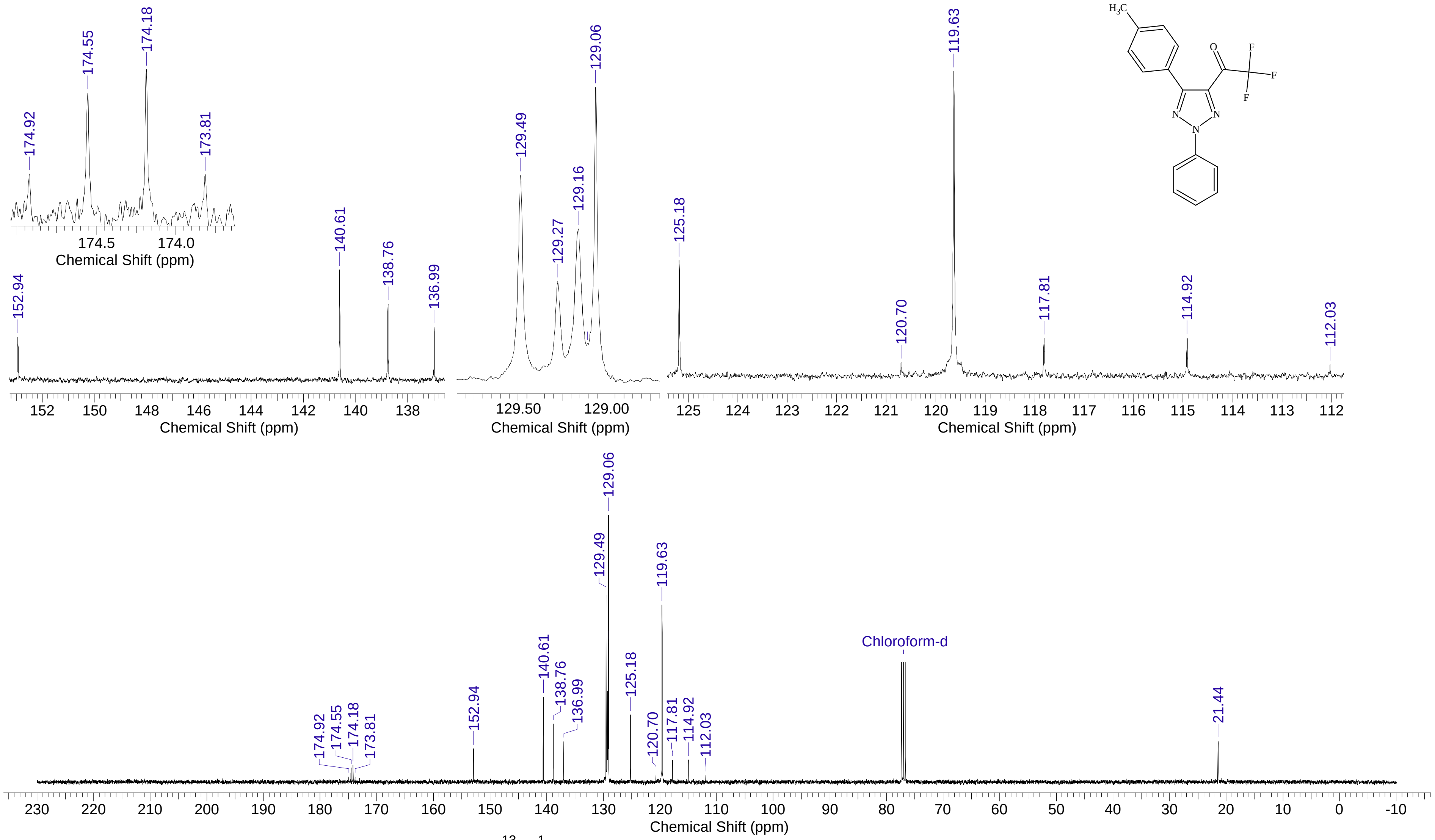

$^{13}C\{^1H\}$ NMR spectrum of **14** (100.6 MHz, $CDCl_3$)

29 Nov 2022

**Acquisition Time (sec)**	4.0894	**Comment**	Imported from UXNMR.			**Date**	15 Nov 2022 14:46:50
**File Name**	G:\DISK_ULTRA\SPEC_H,C_2022\11.ноябрь\SZA-BM-2677-9.H_001001r					**Frequency (MHz)**	400.13
**Nucleus**	1H	**Number of Transients**	4	**Original Points Count**	32768	**Points Count**	131072
**Pulse Sequence**	zg30	**Solvent**	CHLOROFORM-D			**Sweep Width (Hz)**	8012.82
**Temperature (degree C)**	27.000						

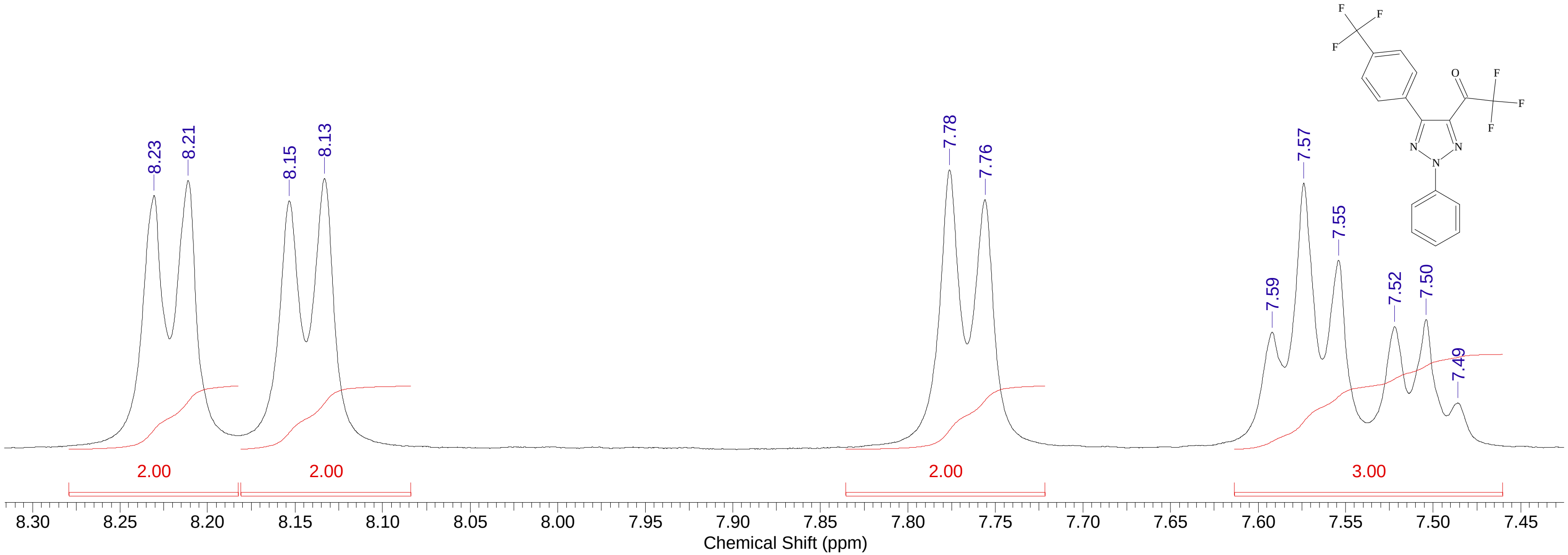

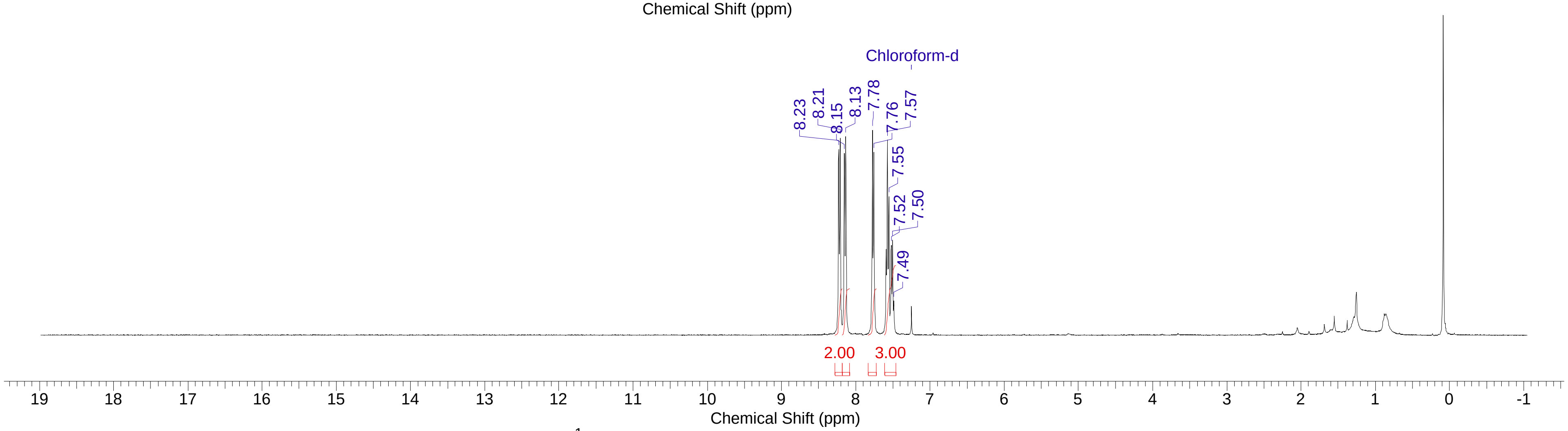

^{1}H NMR spectrum of **15** (400.1 MHz, $CDCl_3$)

29 Nov 2022

Acquisition Time (sec)	1.7433	***Comment***	Imported from UXNMR.			***Date***	15 Nov 2022 15:01:52
File Name	G:\DISK_ULTRA\SPEC_H,C_2022\11.ноябрь\SZA-BM-2677-9.F_005001r					***Frequency (MHz)***	376.50
Nucleus	19F	***Number of Transients***	16	***Original Points Count***	131072	***Points Count***	262144
Pulse Sequence	zgflqn	***Solvent***	CHLOROFORM-D			***Sweep Width (Hz)***	75187.97
Temperature (degree C)	27.000						

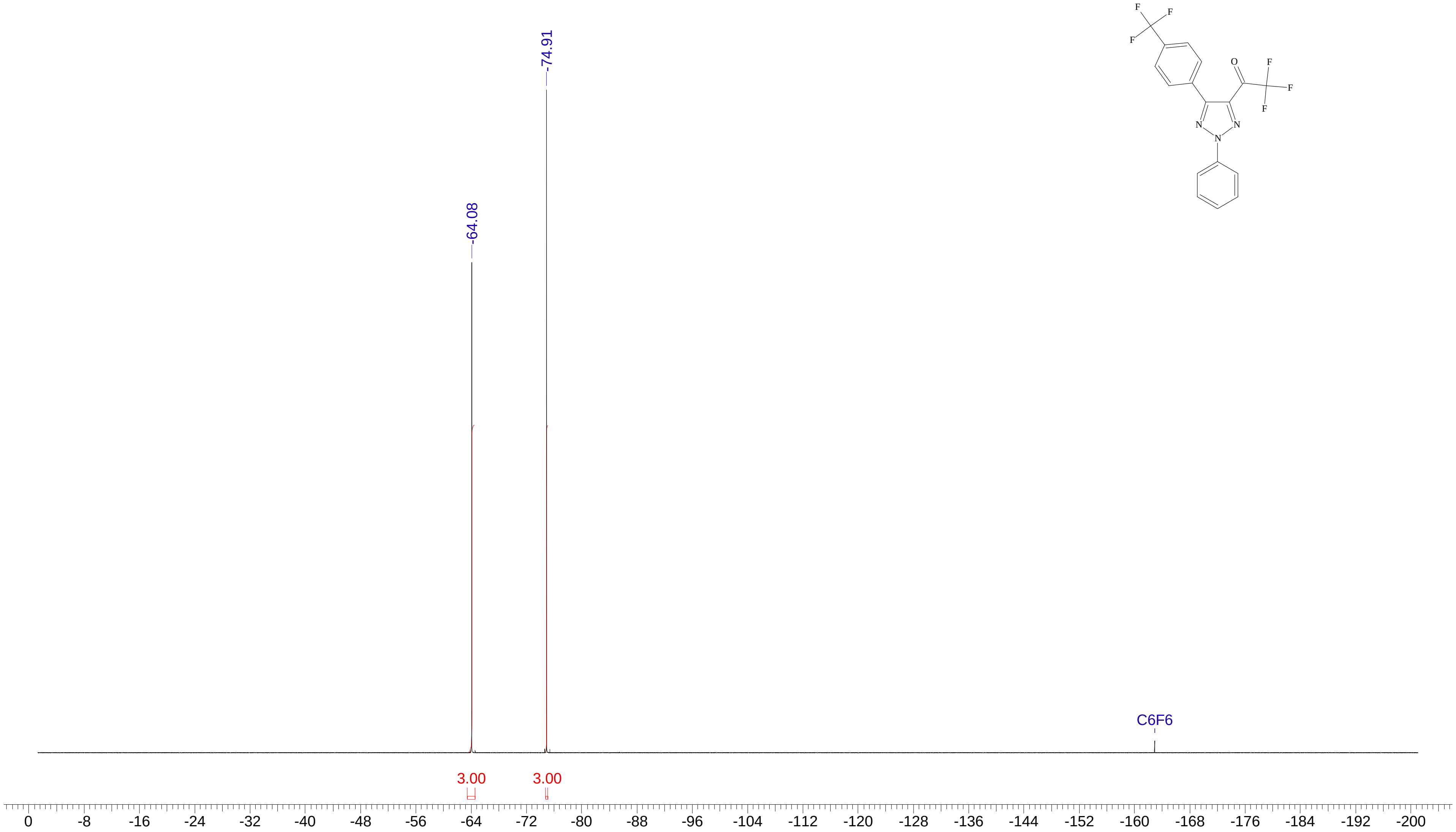

^{19}F NMR spectrum of **15** (376.5 MHz, $CDCl_3$)

29 Nov 2022

**Acquisition Time (sec)**	0.6783	**Comment**	Imported from UXNMR.			**Date**	15 Nov 2022 14:57:30
**File Name**	G:\DISK_ULTRA\SPEC_H,C_2022\11.ноябрь\SZA-BM-2677-9.C_002001r					**Frequency (MHz)**	100.61
**Nucleus**	13C	**Number of Transients**	257	**Original Points Count**	16384	**Points Count**	131072
**Pulse Sequence**	zgpg30	**Solvent**	DEUTERIUM OXIDE			**Sweep Width (Hz)**	24154.59
**Temperature (degree C)**	27.000						

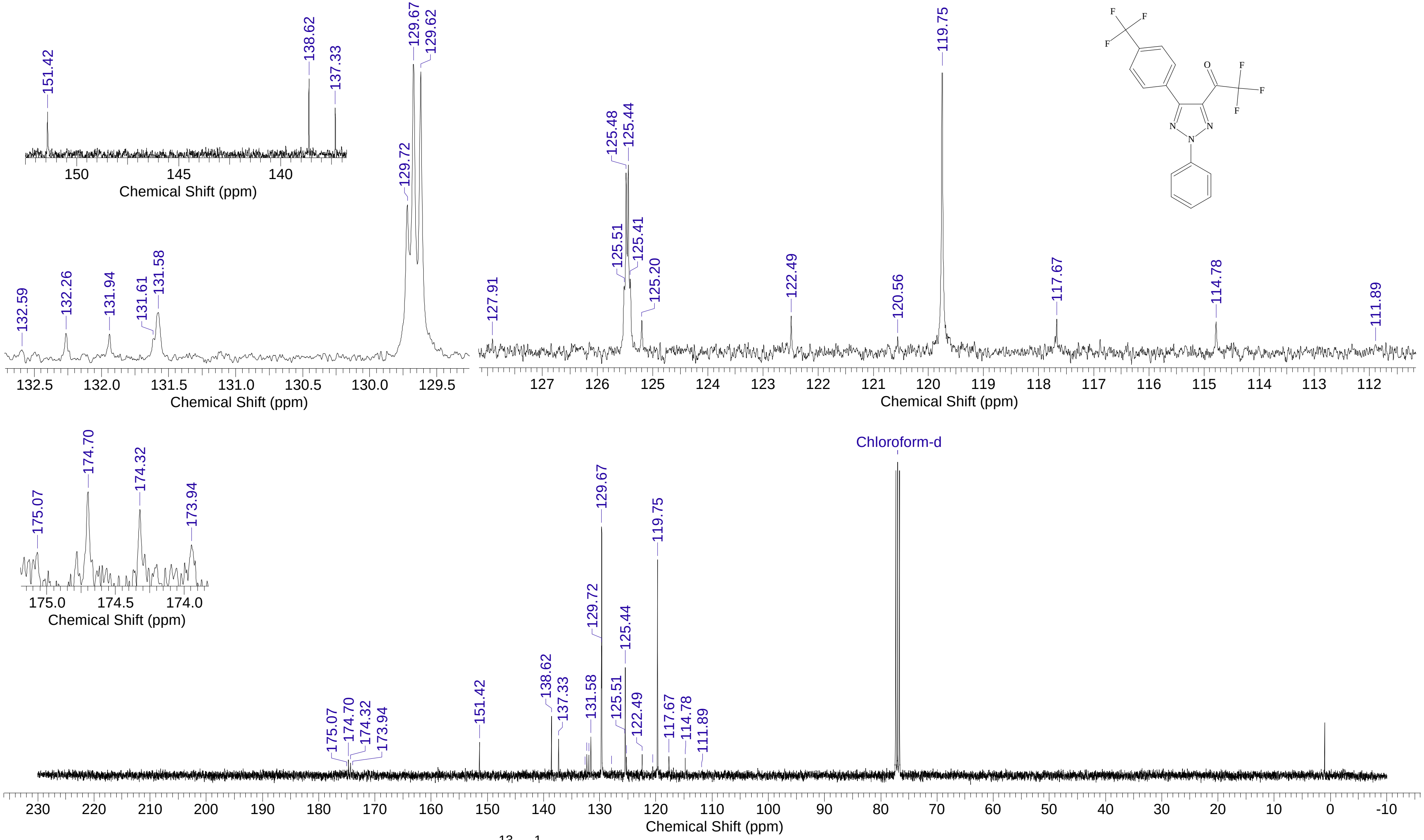

$^{13}C\{^{1}H\}$ NMR spectrum of **15** (100.6 MHz, $CDCl_3$)

29 Nov 2022

Acquisition Time (sec)	4.0894	***Comment***	Imported from UXNMR.	***Date***	20 Oct 2022 12:11:06
File Name	C:\DOCS\OUTPUT_301\2022\10.октябрь\BM-2655.H_001001r			***Frequency (MHz)***	400.13
Nucleus	1H	***Number of Transients***	4	***Points Count***	131072
		Original Points Count	32768		
Pulse Sequence	zg30	***Solvent***	DEUTERIUM OXIDE	***Sweep Width (Hz)***	8012.82
Temperature (degree C)	27.000				

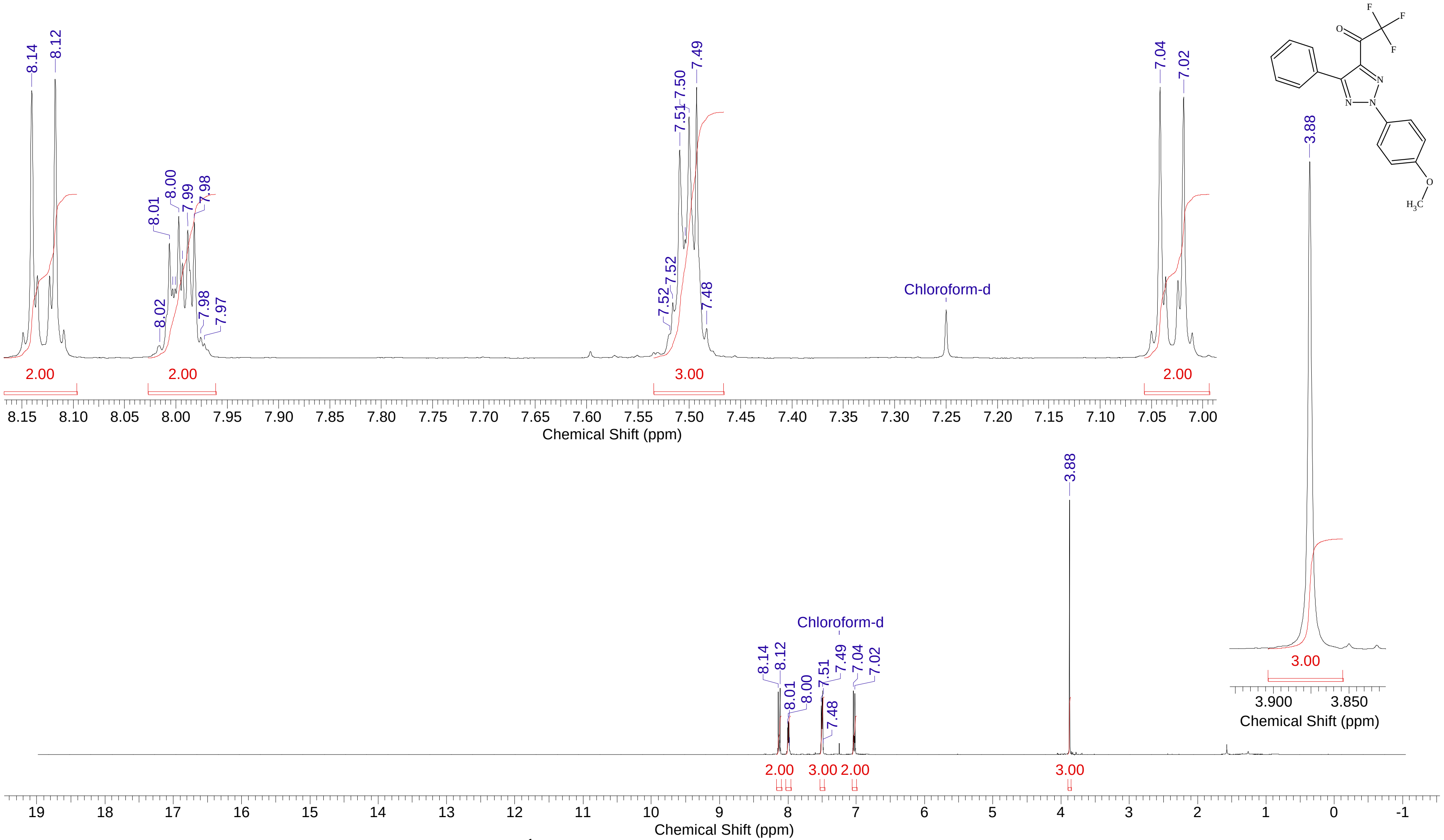

^{1}H NMR spectrum of **16** (400.1 MHz, $CDCl_3$)

29 Nov 2022

Acquisition Time (sec)	1.7433	***Comment***	Imported from UXNMR.			***Date***	20 Oct 2022 12:15:18
File Name	C:\DOCS\OUTPUT_301\2022\10.октябрь\BM-2655.F_005001r					***Frequency (MHz)***	376.50
Nucleus	19F	***Number of Transients***	16	***Original Points Count***	131072	***Points Count***	262144
Pulse Sequence	zgflqn	***Solvent***	BENZENE-D6	***Sweep Width (Hz)***	75187.97	***Temperature (degree C)***	27.000

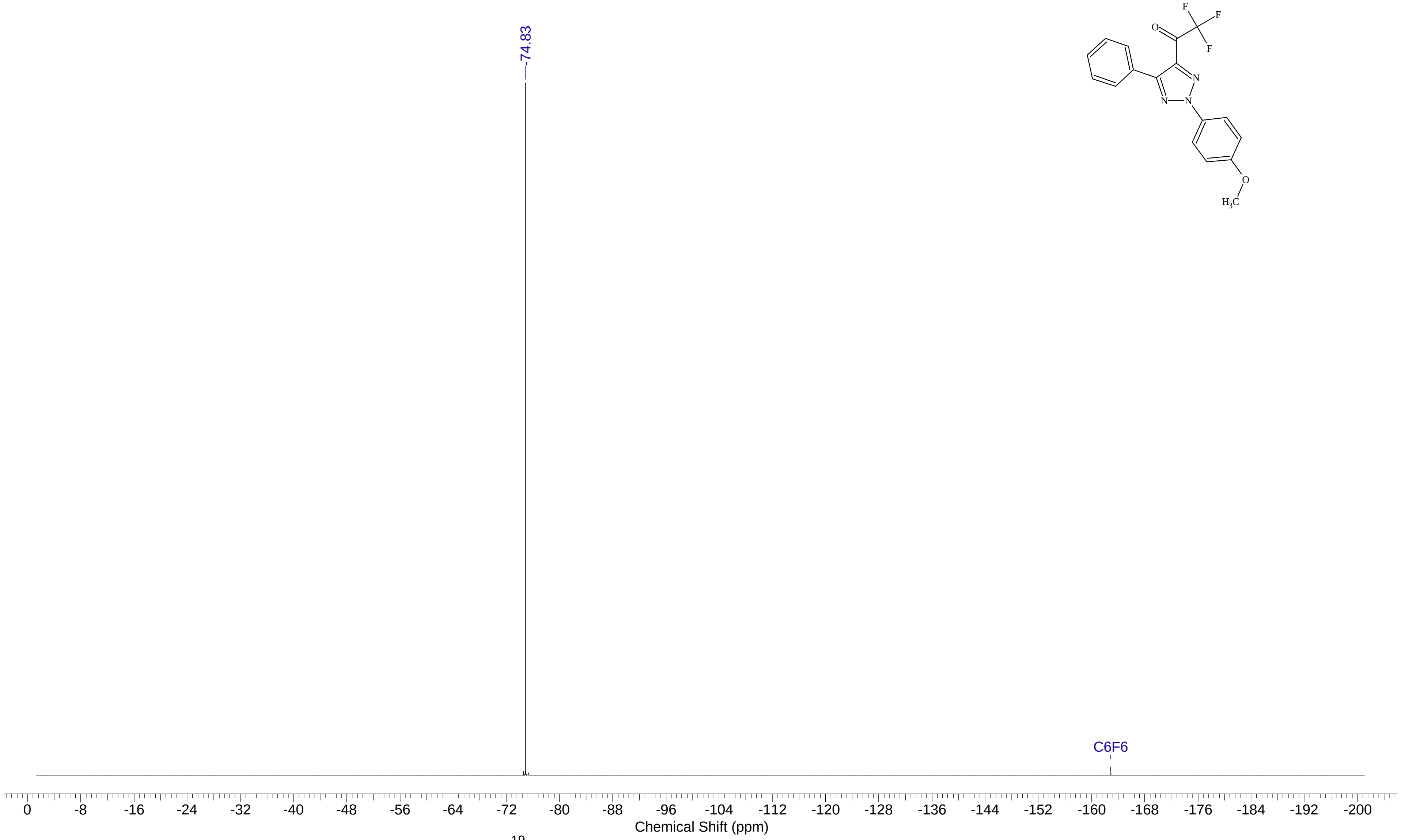

^{19}F NMR spectrum of **16** (376.5 MHz, $CDCl_3$)

29 Nov 2022

Acquisition Time (sec)	0.6783	***Comment***	Imported from UXNMR.			***Date***	20 Oct 2022 12:09:26
File Name	C:\DOCS\OUTPUT_301\2022\10.октябрь\BM-2655.C_002001r					***Frequency (MHz)***	100.61
Nucleus	13C	***Number of Transients***	190	***Original Points Count***	16384	***Points Count***	131072
Pulse Sequence	zgpg30	***Solvent***	DMSO-D6	***Sweep Width (Hz)***	24154.59	***Temperature (degree C)***	27.000

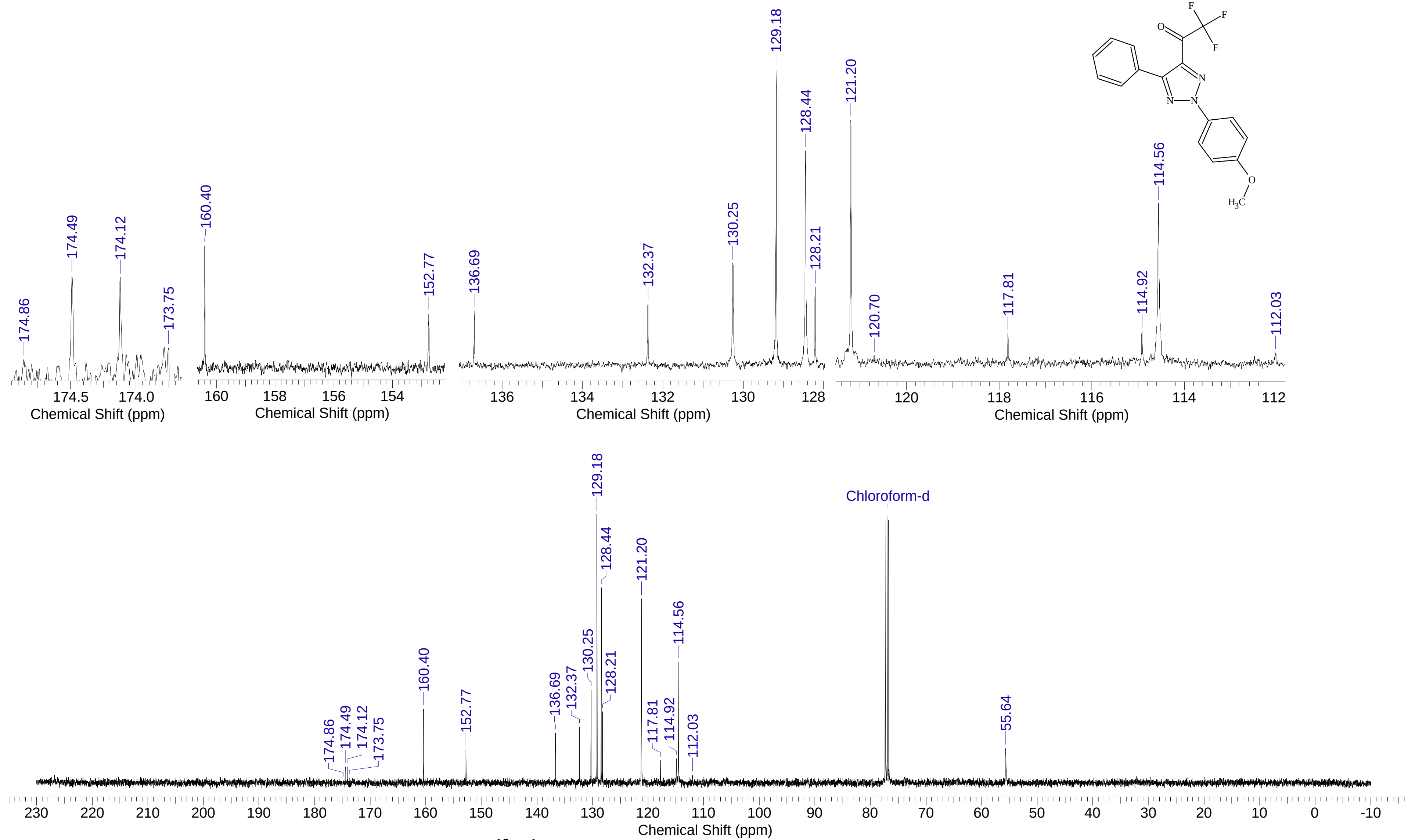

$^{13}C\{^1H\}$ NMR spectrum of **16** (100.6 MHz, $CDCl_3$)

29 Nov 2022

Acquisition Time (sec)	4.0894	***Comment***	Imported from UXNMR.	***Date***	20 Oct 2022 23:52:48
File Name	C:\DOCS\BM\ЯМР_СОСЕДИ\2022\bm221020\BM-2656-2p_001001r			***Frequency (MHz)***	400.13
Nucleus	1H	***Number of Transients***	8	***Points Count***	131072
Pulse Sequence	zg30	***Solvent***	CHLOROFORM-D	***Sweep Width (Hz)***	8012.82
Temperature (degree C)	27.000				

Original Points Count: 32768

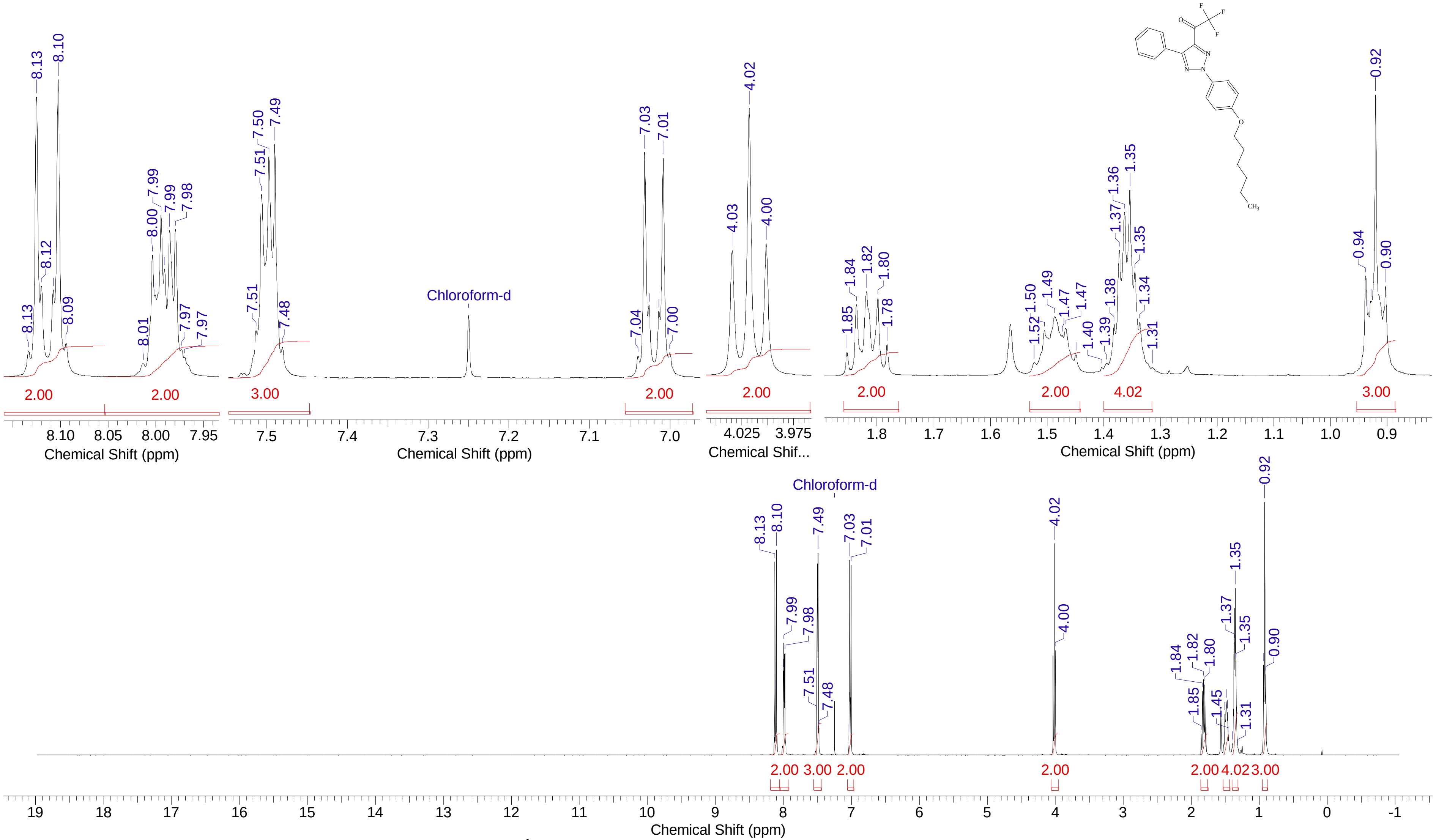

^{1}H NMR spectrum of **17** (400.1 MHz, $CDCl_3$)

29 Nov 2022

**Acquisition Time (sec)**	1.7433	**Comment**	Imported from UXNMR.			**Date**	21 Oct 2022 15:57:16
**File Name**	C:\DOCS\OUTPUT_301\2022\10.октябрь\BM-2656-2p.F_005001r					**Frequency (MHz)**	376.50
**Nucleus**	19F	**Number of Transients**	16	**Original Points Count**	131072	**Points Count**	262144
**Pulse Sequence**	zgflqn	**Solvent**	BENZENE-D6	**Sweep Width (Hz)**	75187.97	**Temperature (degree C)**	27.000

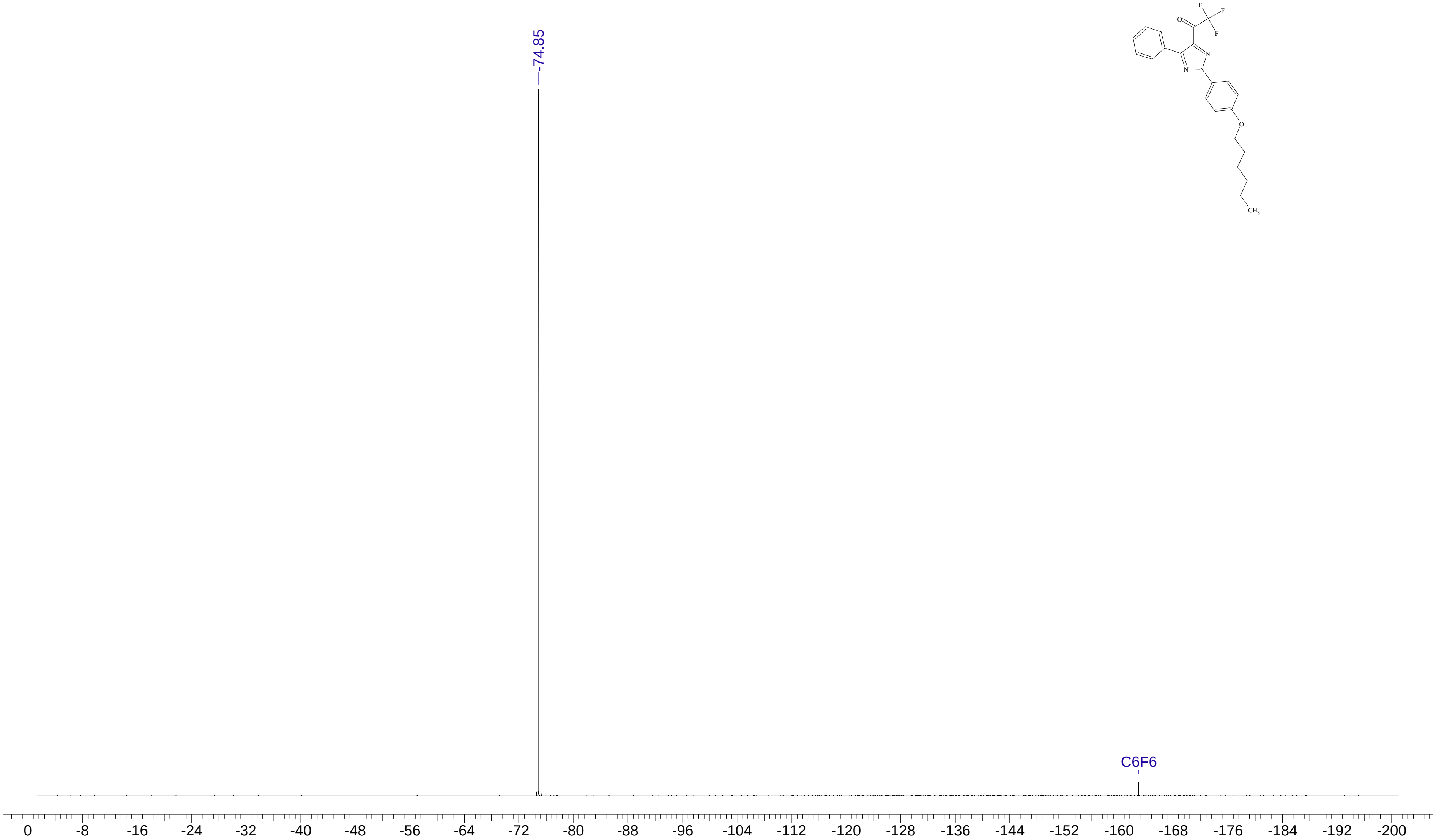

^{19}F NMR spectrum of **17** (376.5 MHz, $CDCl_3$)

29 Nov 2022

Acquisition Time (sec)	0.6783	Comment	Imported from UXNMR.			Date	21 Oct 2022 16:30:44
File Name	C:\DOCS\OUTPUT_301\2022\10.октябрь\BM-2656-2p.C_002001r					Frequency (MHz)	100.61
Nucleus	13C	Number of Transients	386	Original Points Count	16384	Points Count	131072
Pulse Sequence	zgpg30	Solvent	CHLOROFORM-D			Sweep Width (Hz)	24154.59
Temperature (degree C)	27.000						

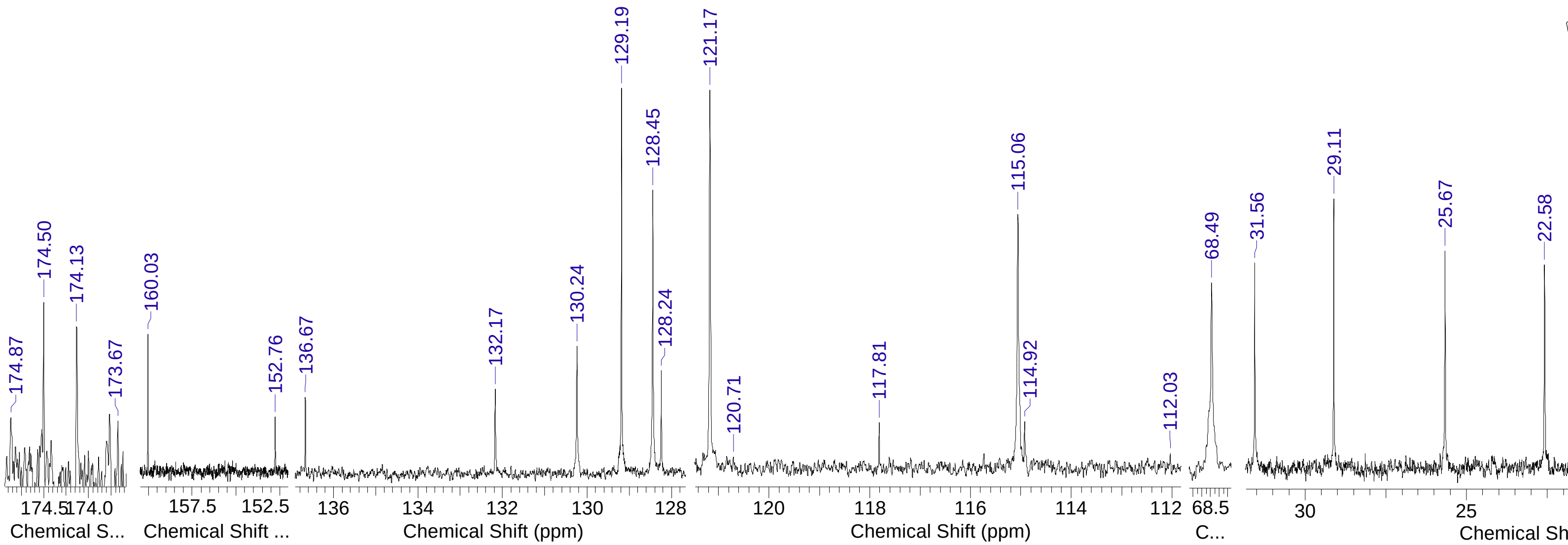

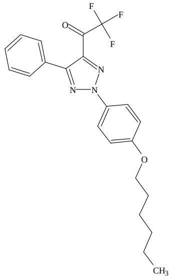

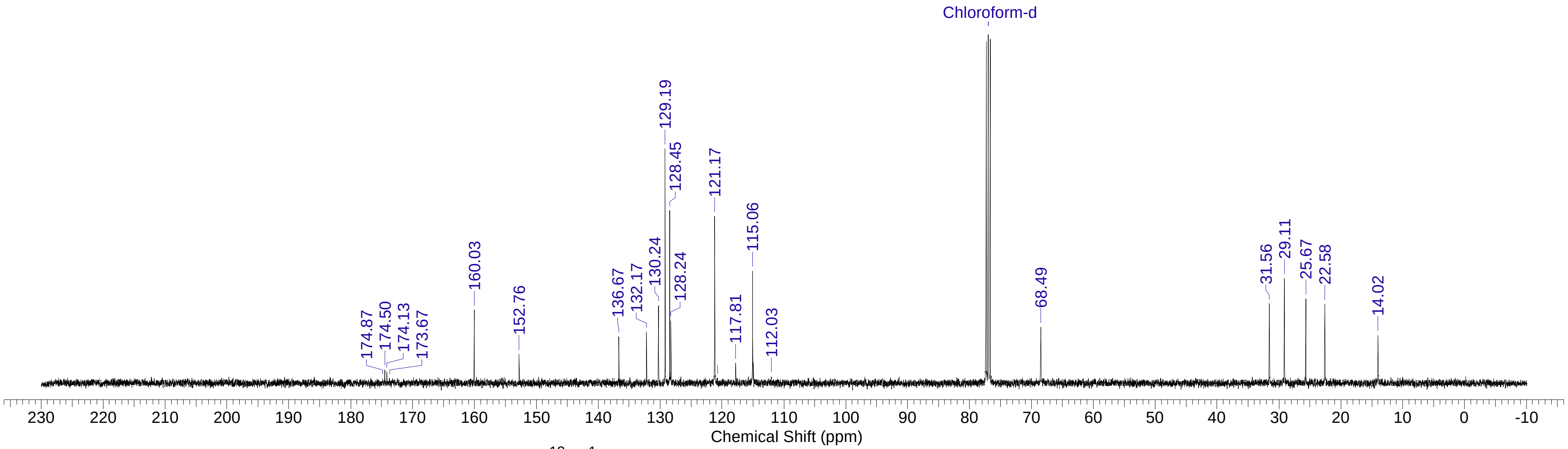

$^{13}C\{^{1}H\}$ NMR spectrum of **17** (100.6 MHz, $CDCl_3$)

29 Nov 2022

Acquisition Time (sec)	4.0894	***Comment***	Imported from UXNMR.			***Date***	11 Nov 2022 15:50:04
File Name	C:\BM_DATA\DOCS\SZA-BM-11.11.2022\SZA-BM-2674(2).H_001001r					***Frequency (MHz)***	400.13
Nucleus	1H	***Number of Transients***	4	***Original Points Count***	32768	***Points Count***	131072
Pulse Sequence	zg30	***Solvent***	DMSO-D6	***Sweep Width (Hz)***	8012.82	***Temperature (degree C)***	27.000

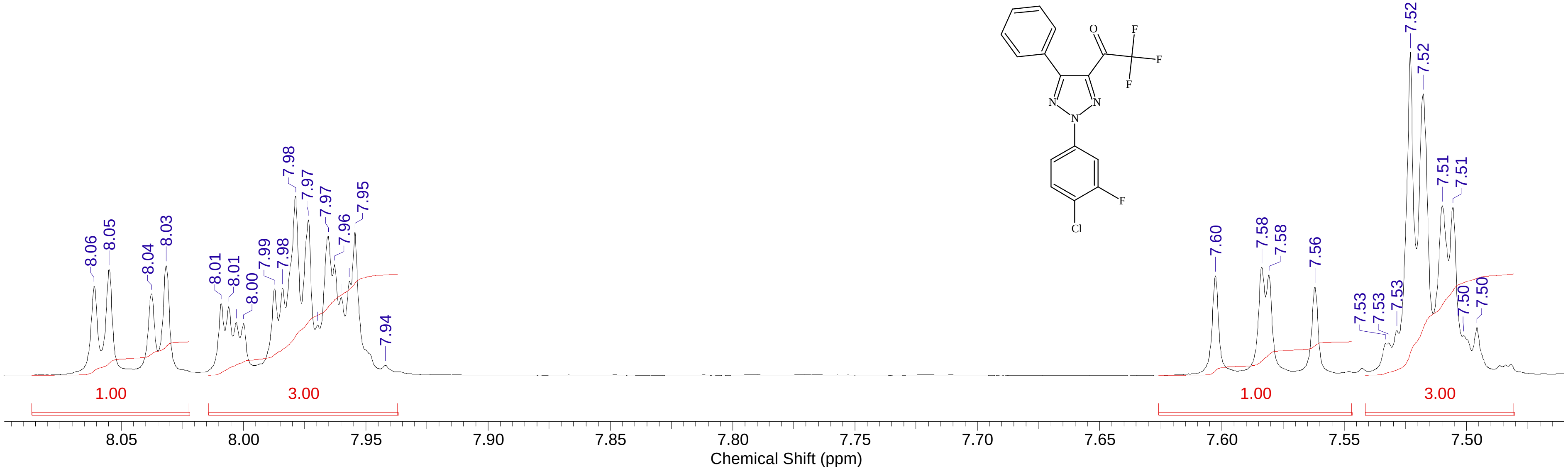

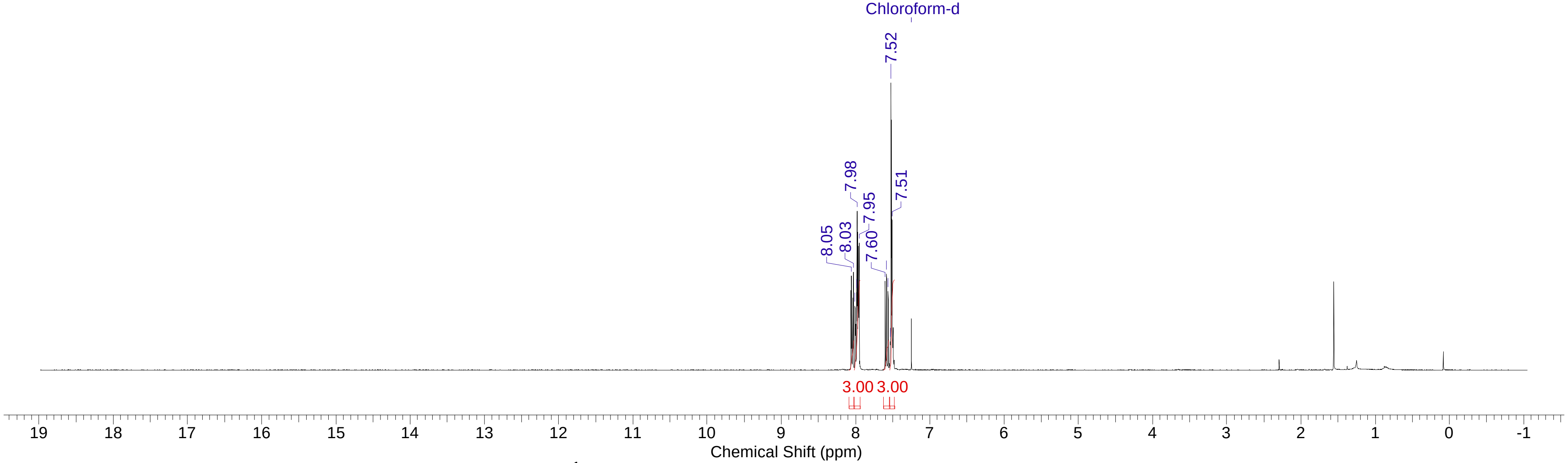

^{1}H NMR spectrum of **18** (400.1 MHz, $CDCl_3$)

29 Nov 2022

Acquisition Time (sec)	1.7433	***Comment***	Imported from UXNMR.			***Date***	11 Nov 2022 15:48:12
File Name	C:\BM_DATA\DOCS\SZA-BM-11.11.2022\SZA-BM-2674(2).F_005001r					***Frequency (MHz)***	376.50
Nucleus	19F	***Number of Transients***	9	***Original Points Count***	131072	***Points Count***	262144
Pulse Sequence	zgflqn	***Solvent***	CHLOROFORM-D			***Sweep Width (Hz)***	75187.97
Temperature (degree C)	27.000						

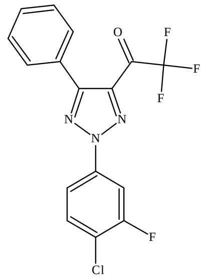

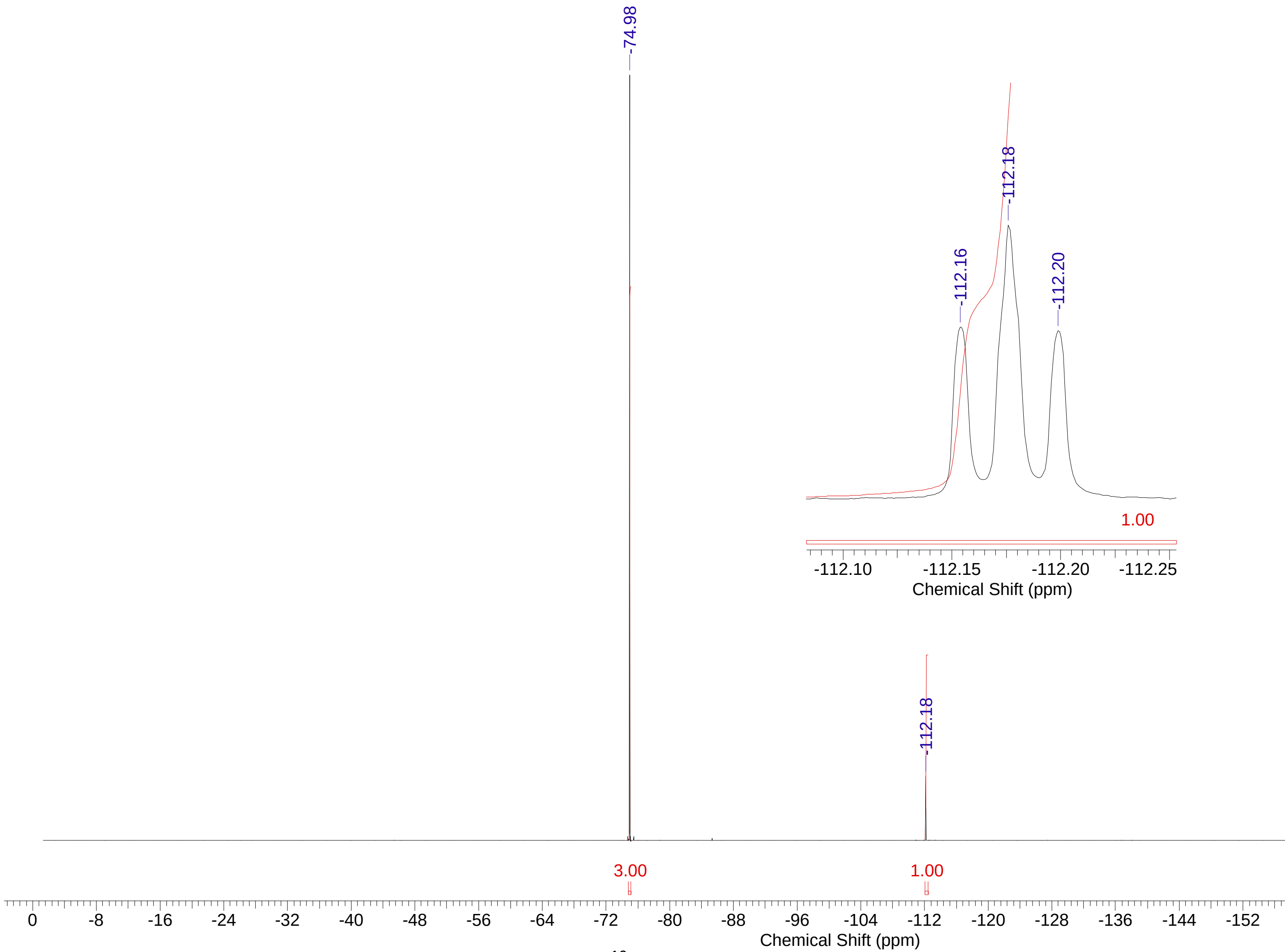

^{19}F NMR spectrum of **18** (376.5 MHz, $CDCl_3$)

29 Nov 2022

Acquisition Time (sec)	0.6783	Comment	Imported from UXNMR.			Date	11 Nov 2022 15:59:10
File Name	C:\BM_DATA\DOCS\SZA-BM-11.11.2022\SZA-BM-2674.C_002001r					Frequency (MHz)	100.61
Nucleus	13C	Number of Transients	193	Original Points Count	16384	Points Count	131072
Pulse Sequence	zgpg30	Solvent	CHLOROFORM-D			Sweep Width (Hz)	24154.59
Temperature (degree C)	27.000						

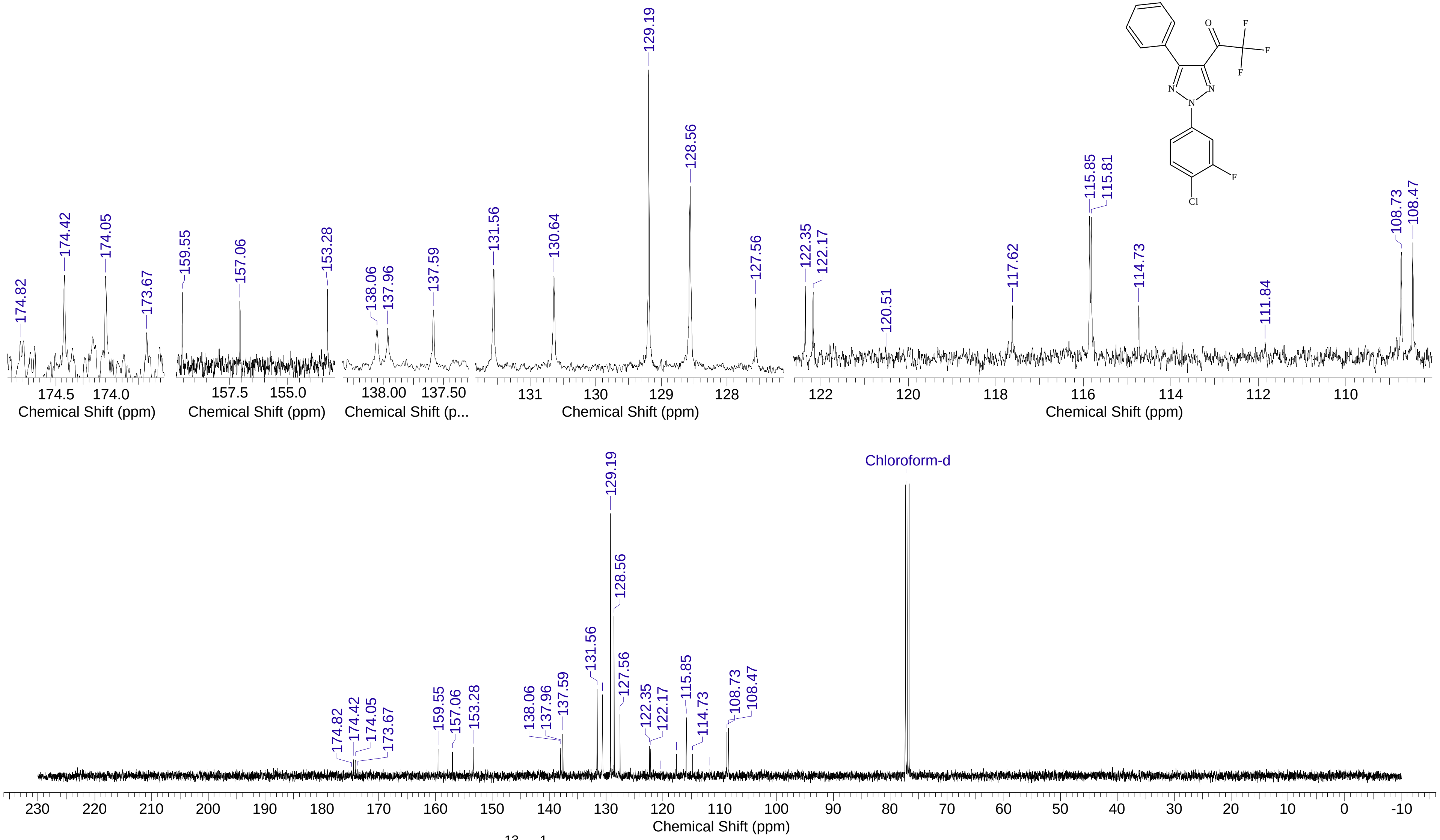

$^{13}C\{^1H\}$ NMR spectrum of **18** (100.6 MHz, $CDCl_3$)

29 Nov 2022

Acquisition Time (sec)	4.0894	***Comment***	Imported from UXNMR.			***Date***	11 Nov 2022 15:36:22
File Name	C:\BM_DATA\DOCS\SZA-BM-11.11.2022\SZA-BM-2675-8.H_001001r					***Frequency (MHz)***	400.13
Nucleus	1H	***Number of Transients***	4	***Original Points Count***	32768	***Points Count***	131072
Pulse Sequence	zg30	***Solvent***	DEUTERIUM OXIDE			***Sweep Width (Hz)***	8012.82
Temperature (degree C)	27.000						

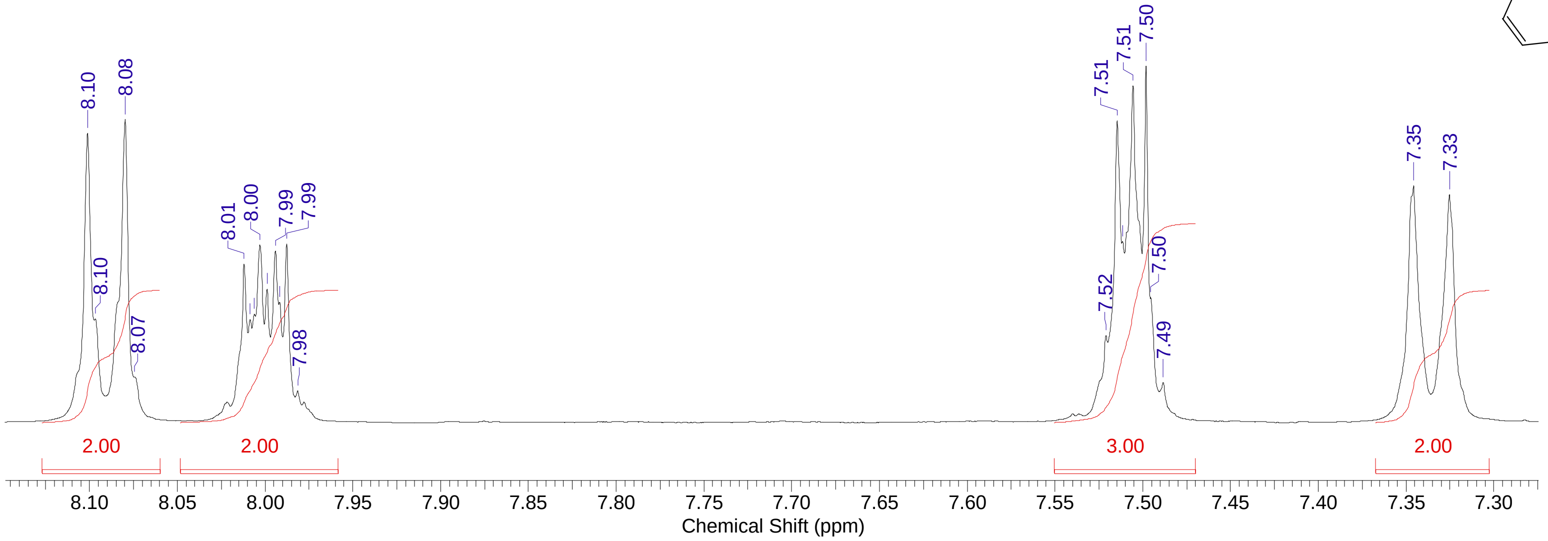

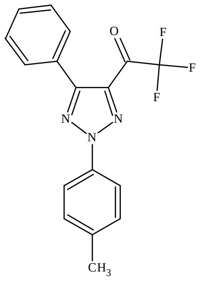

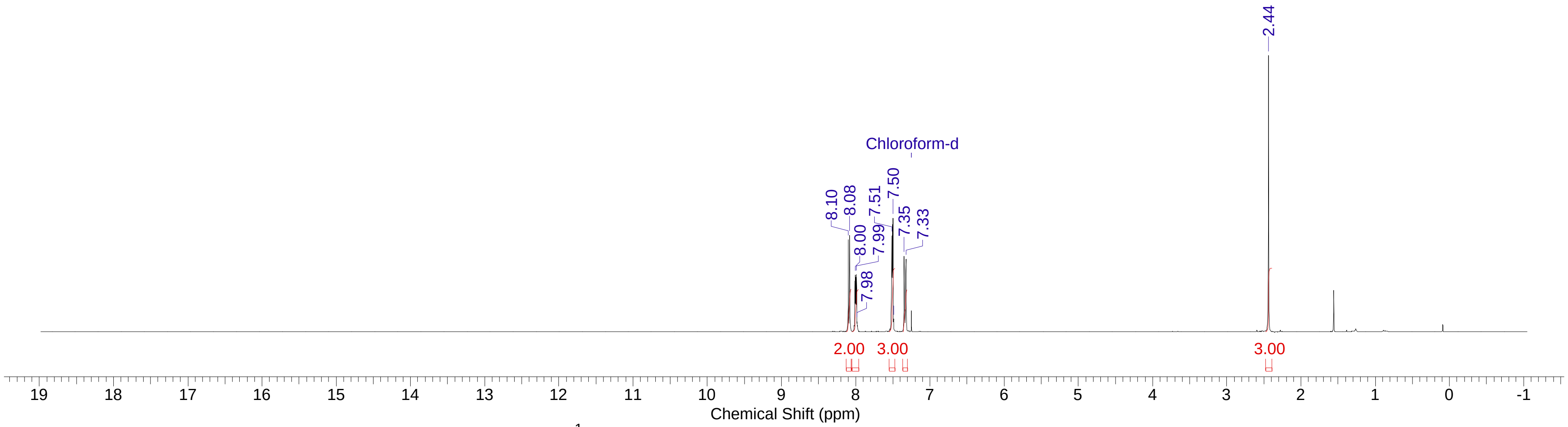

^{1}H NMR spectrum of **19** (400.1 MHz, $CDCl_3$)

29 Nov 2022

Acquisition Time (sec)	1.7433	***Comment***	Imported from UXNMR.	***Date***	11 Nov 2022 15:39:46
File Name	C:\BM_DATA\DOCS\SZA-BM-11.11.2022\SZA-BM-2675-8.F_005001r			***Frequency (MHz)***	376.50
Nucleus	19F	***Number of Transients***	13 ***Original Points Count*** 131072	***Points Count***	262144
Pulse Sequence	zgflqn	***Solvent***	CHLOROFORM-D	***Sweep Width (Hz)***	75187.97
Temperature (degree C)	27.000				

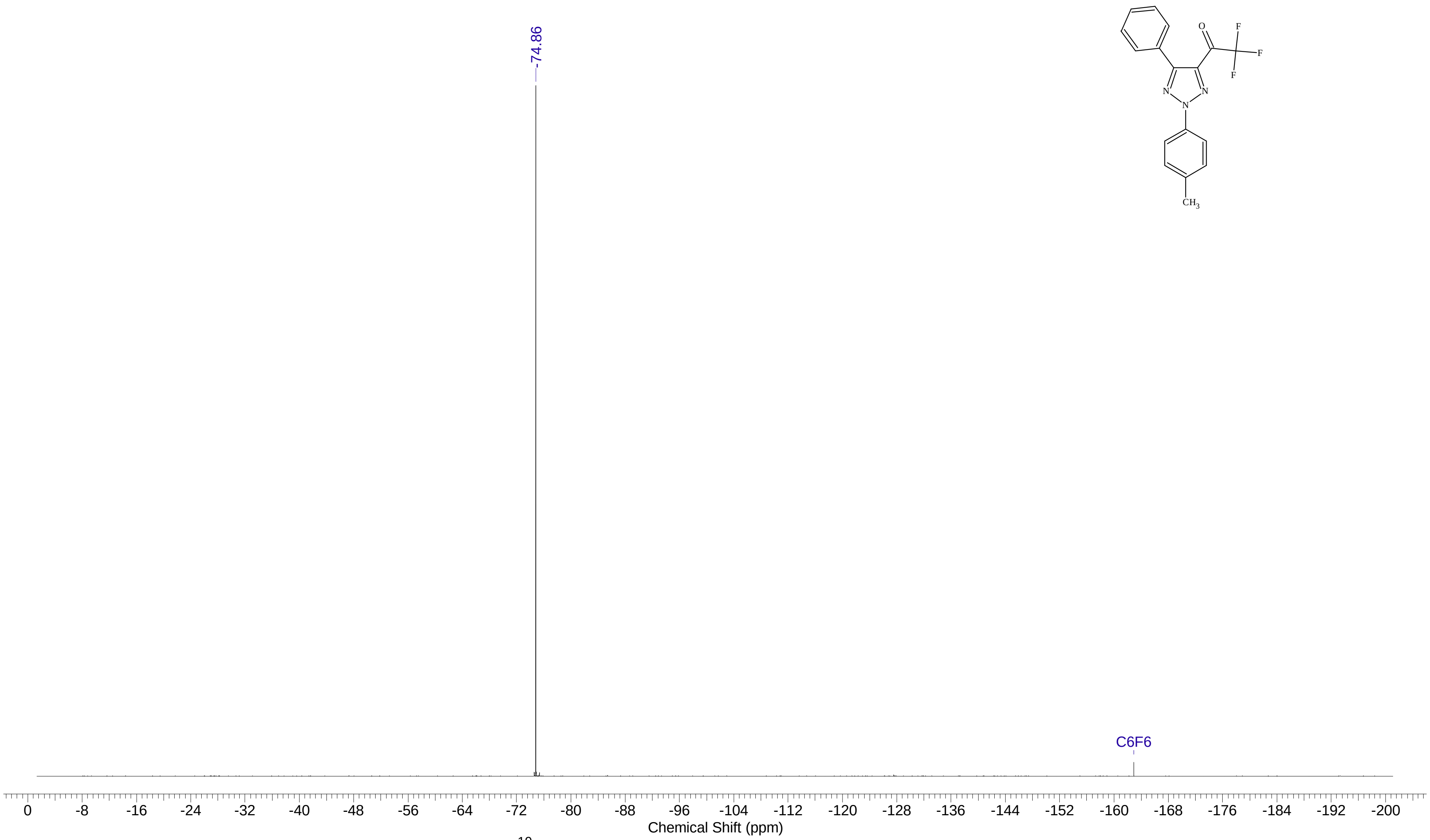

^{19}F NMR spectrum of **19** (376.5 MHz, $CDCl_3$)

29 Nov 2022

Acquisition Time (sec)	0.6783	Comment	Imported from UXNMR.			Date	11 Nov 2022 16:04:30
**File Name**	C:\BM_DATA\DOCS\SZA-BM-11.11.2022\SZA-BM-2675-8.C_002001r					**Frequency (MHz)**	100.61
**Nucleus**	13C	**Number of Transients**	71	**Original Points Count**	16384	**Points Count**	131072
**Pulse Sequence**	zgpg30	**Solvent**	CHLOROFORM-D			**Sweep Width (Hz)**	24154.59
**Temperature (degree C)**	27.000						

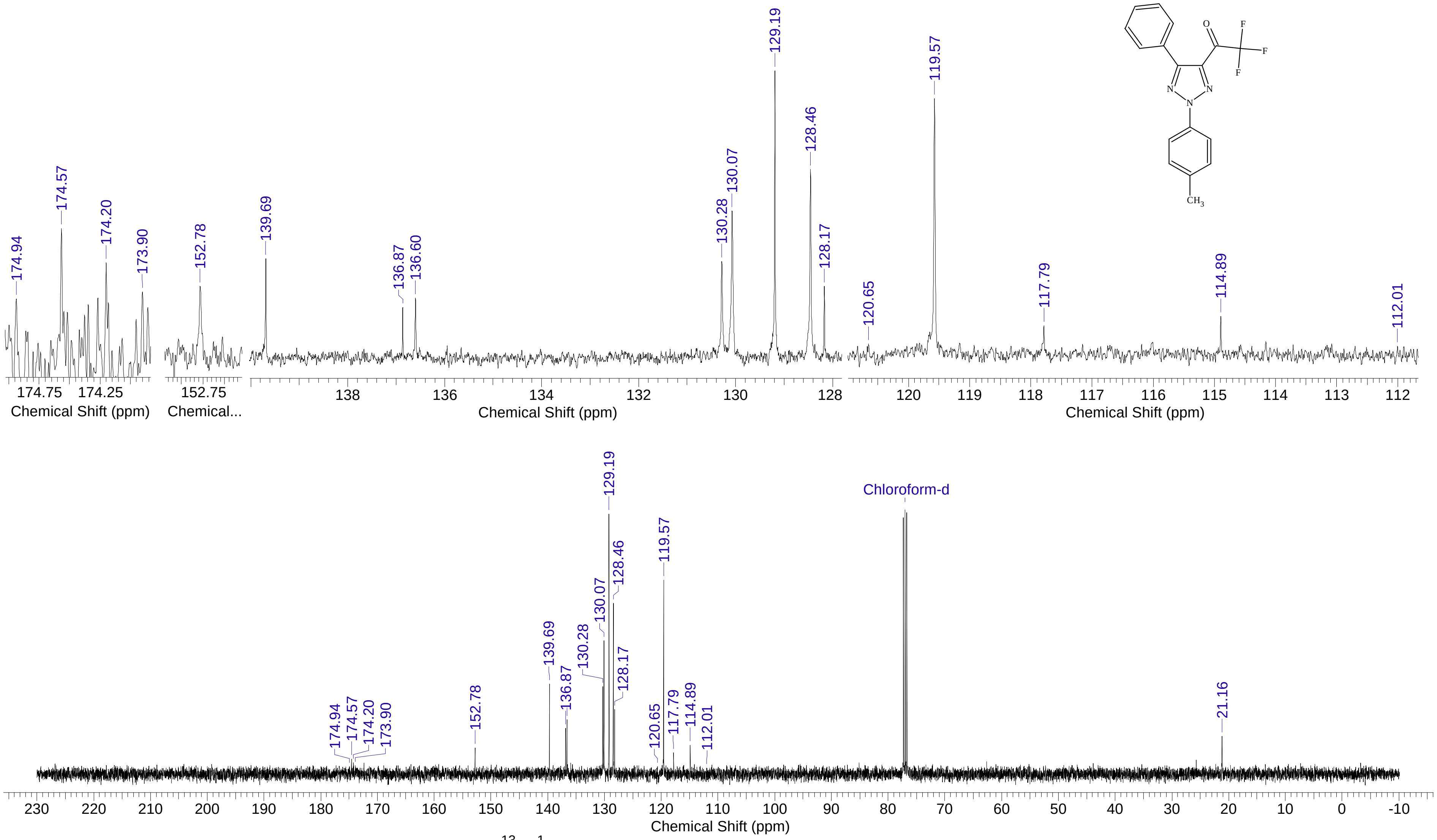

$^{13}C\{^1H\}$ NMR spectrum of **19** (100.6 MHz, $CDCl_3$)

29 Nov 2022

**Acquisition Time (sec)**	4.0894	**Comment**	Imported from UXNMR.			**Date**	10 Nov 2022 14:03:54
**File Name**	C:\BM_DATA\DOCS\10.11.22\10.11.22\BM-2673.H_001001r					**Frequency (MHz)**	400.13
**Nucleus**	1H	**Number of Transients**	4	**Original Points Count**	32768	**Points Count**	131072
**Pulse Sequence**	zg30	**Solvent**	DMSO-D6	**Sweep Width (Hz)**	8012.82	**Temperature (degree C)**	27.000

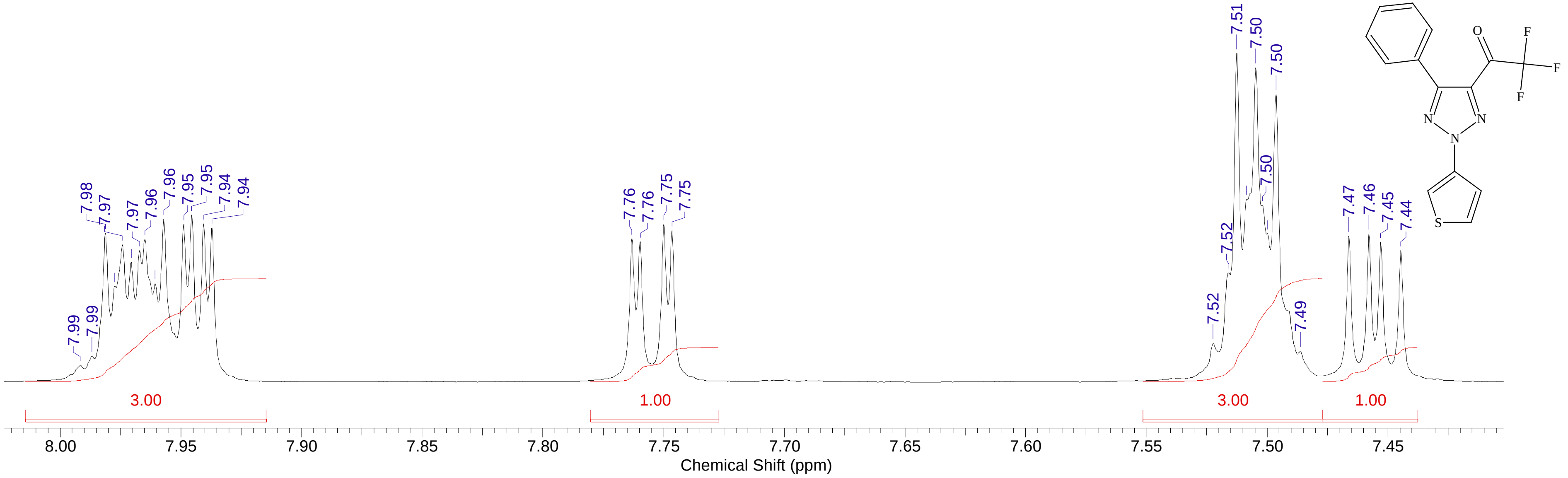

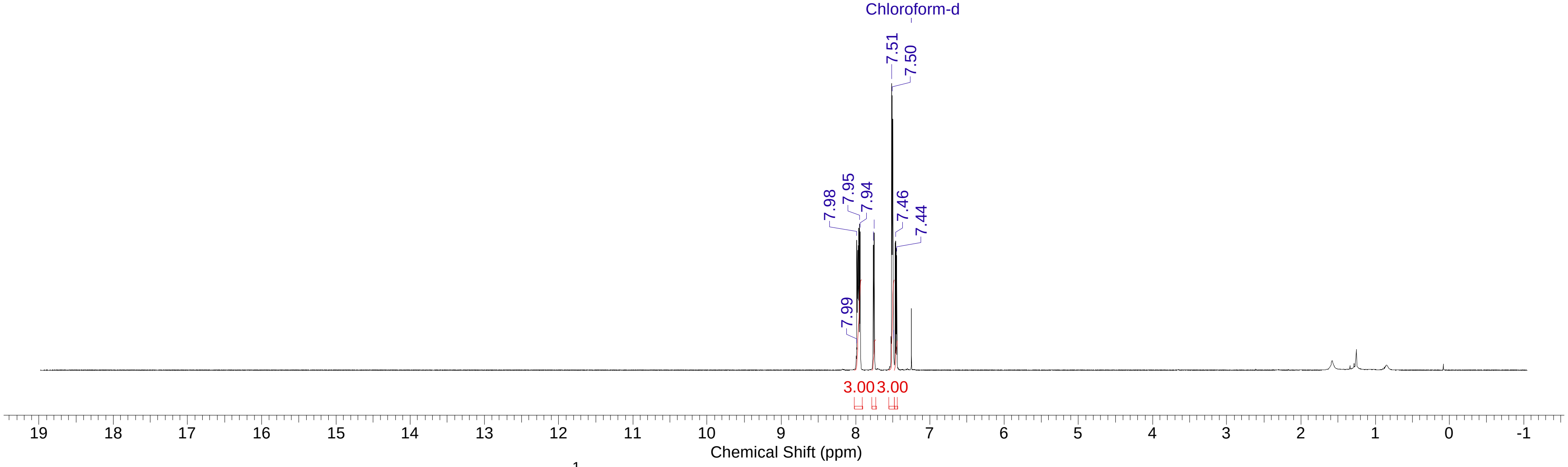

^{1}H NMR spectrum of **20** (400.1 MHz, $CDCl_3$)

Acquisition Time (sec)	1.7433	Comment	Imported from UXNMR.	Date	10 Nov 2022 14:01:52
File Name	C:\BM_DATA\DOCS\10.11.22\10.11.22\BM-2673.F_005001r	Frequency (MHz)	376.50	Nucleus	19F
Number of Transients	16	Original Points Count 131072	Points Count 262144	Pulse Sequence	zgflqn
Solvent	CHLOROFORM-D	Sweep Width (Hz)	75187.97	Temperature (degree C)	27.000

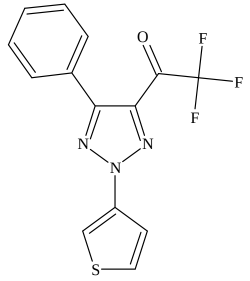

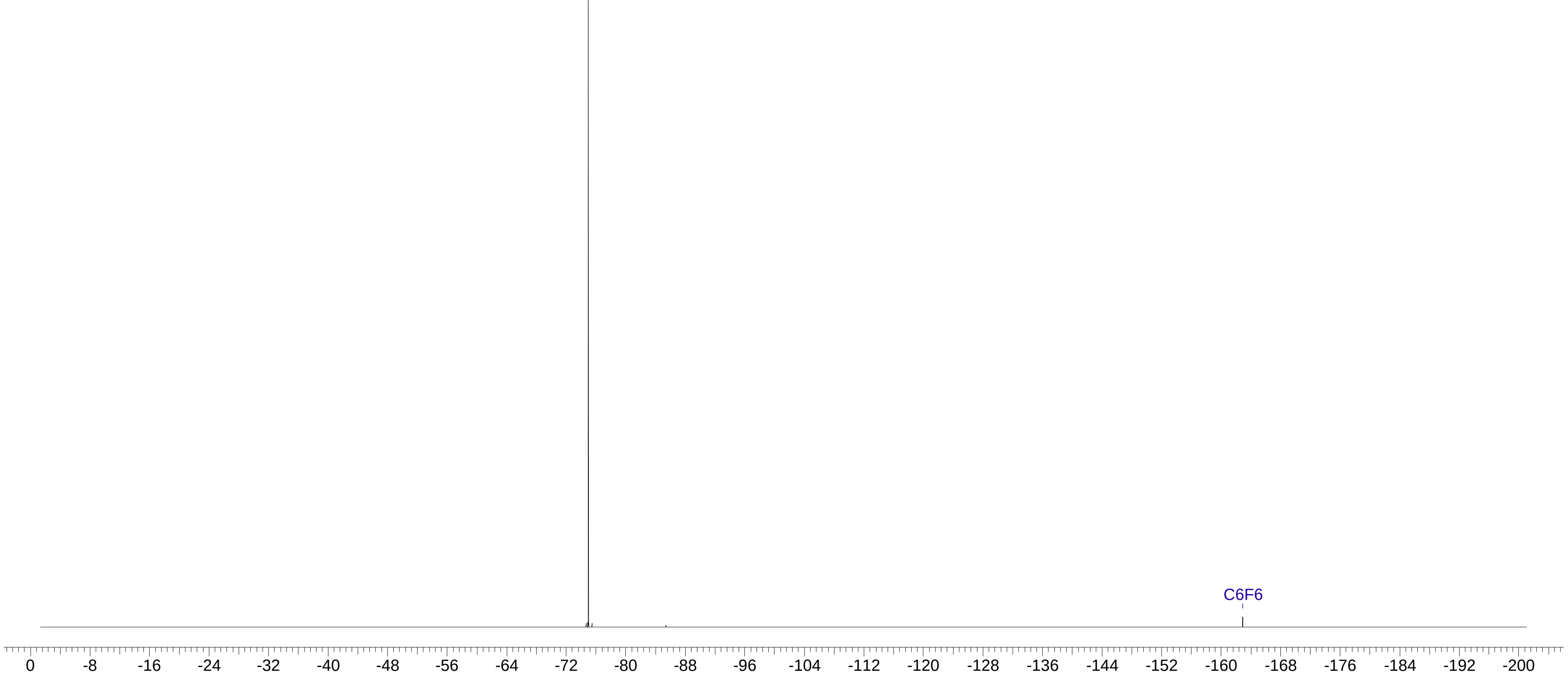

^{19}F NMR spectrum of **20** (376.5 MHz, $CDCl_3$)

29 Nov 2022

Acquisition Time (sec)	0.6783	***Comment***	Imported from UXNMR.			***Date***	10 Nov 2022 14:21:18
File Name	C:\BM_DATA\DOCS\10.11.22\10.11.22\BM-2673.C_002001r			***Frequency (MHz)***	100.61	***Nucleus***	13C
Number of Transients	386	***Original Points Count***	16384	***Points Count***	131072	***Pulse Sequence***	zgpg30
Solvent	CHLOROFORM-D			***Sweep Width (Hz)***	24154.59	***Temperature (degree C)***	27.000

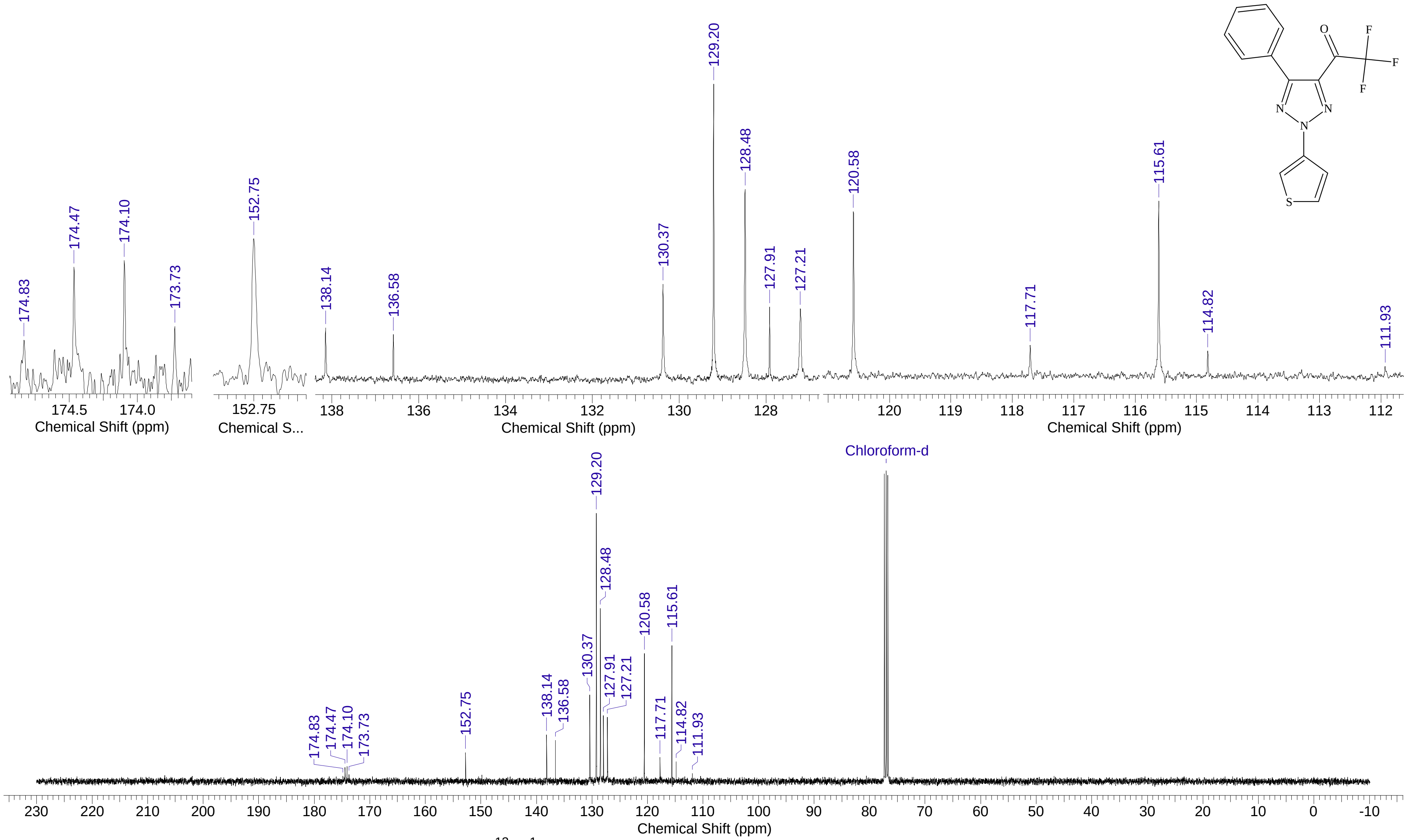

$^{13}C\{^{1}H\}$ NMR spectrum of **20** (100.6 MHz, $CDCl_3$)

4 Jan 2023

Acquisition Time (sec)	4.0894	***Comment***	Imported from UXNMR.			***Date***	17 Dec 2022 13:36:08
File Name	C:\DOCS\OUTPUT_301\2022\12.декабрь\BM-2702.H_001001r					***Frequency (MHz)***	400.13
Nucleus	1H	***Number of Transients***	4	***Original Points Count***	32768	***Points Count***	131072
Pulse Sequence	zg30	***Solvent***	CHLOROFORM-D			***Sweep Width (Hz)***	8012.82
Temperature (degree C)	27.000						

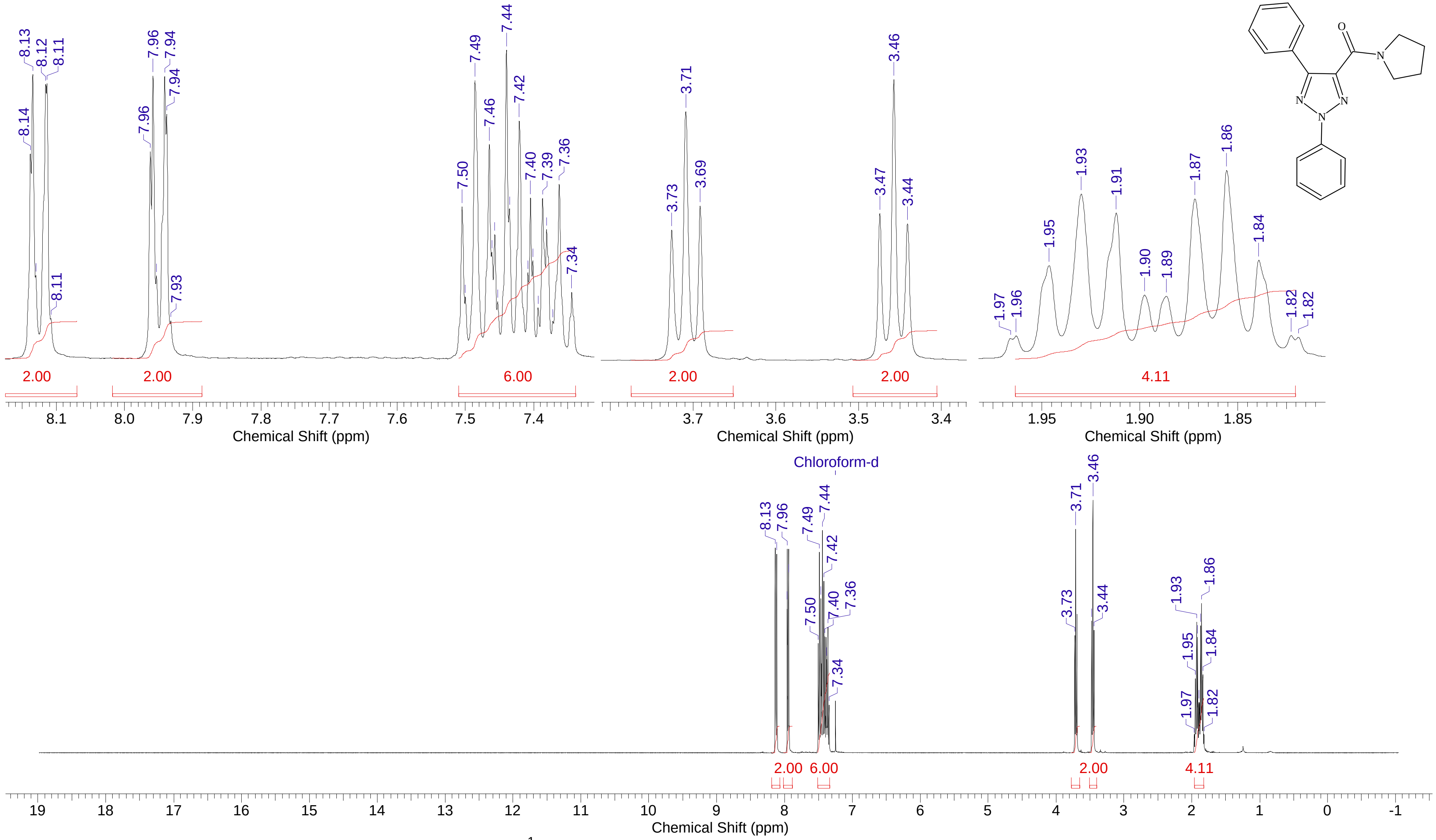

^{1}H NMR spectrum of **21** (400.1 MHz, $CDCl_3$)

4 Jan 2023

Acquisition Time (sec)	0.6783	***Comment***	Imported from UXNMR.			***Date***	17 Dec 2022 13:44:02
File Name	C:\DOCS\OUTPUT_301\2022\12.декабрь\BM-2702.C_002001r					***Frequency (MHz)***	100.61
Nucleus	13C	***Number of Transients***	175	***Original Points Count***	16384	***Points Count***	131072
Pulse Sequence	zgpg30	***Solvent***	DEUTERIUM OXIDE			***Sweep Width (Hz)***	24154.59
Temperature (degree C)	27.000						

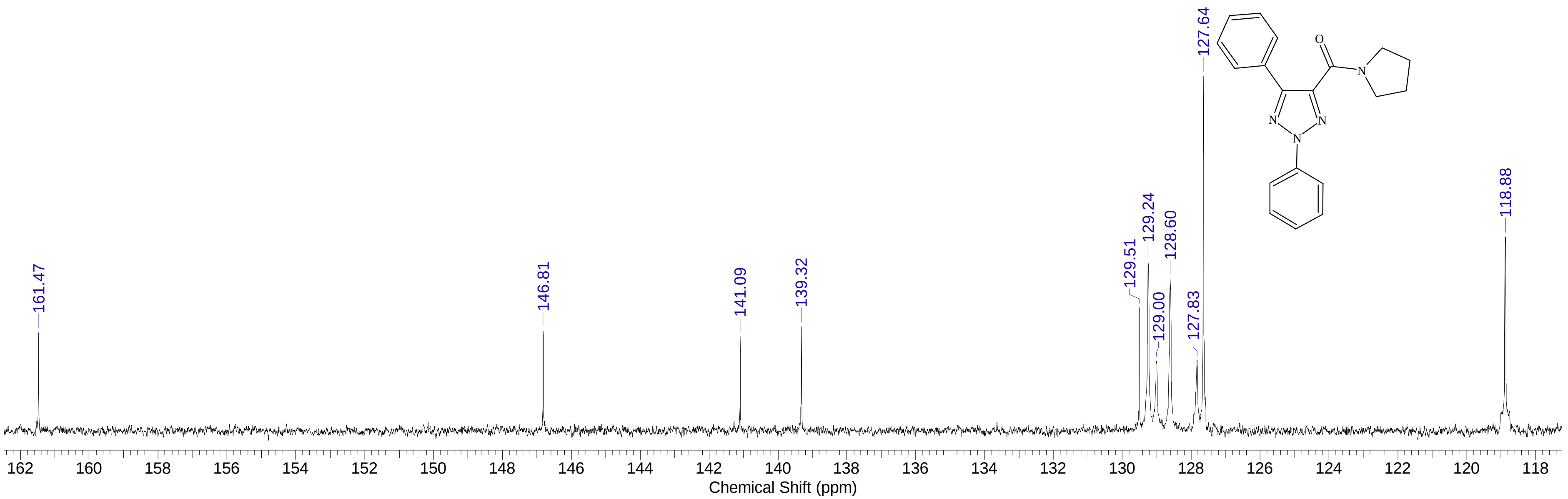

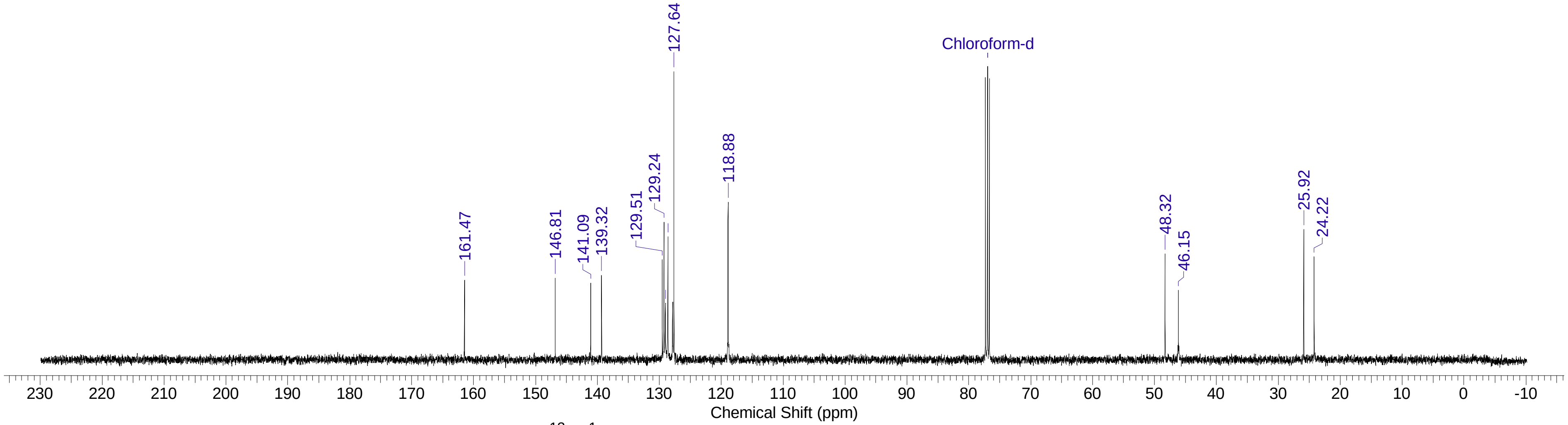

$^{13}C\{^{1}H\}$ NMR spectrum of **21** (100.6 MHz, $CDCl_3$)

4 Jan 2023

Acquisition Time (sec)	4.0894	***Comment***	Imported from UXNMR.			***Date***	20 Dec 2022 14:25:18
File Name	C:\DOCS\OUTPUT_301\2022\12.декабрь\SZA-BM-2706-16.H_001001r					***Frequency (MHz)***	400.13
Nucleus	1H	***Number of Transients***	4	***Original Points Count***	32768	***Points Count***	131072
Pulse Sequence	zg30	***Solvent***	CHLOROFORM-D			***Sweep Width (Hz)***	8012.82
Temperature (degree C)	27.000						

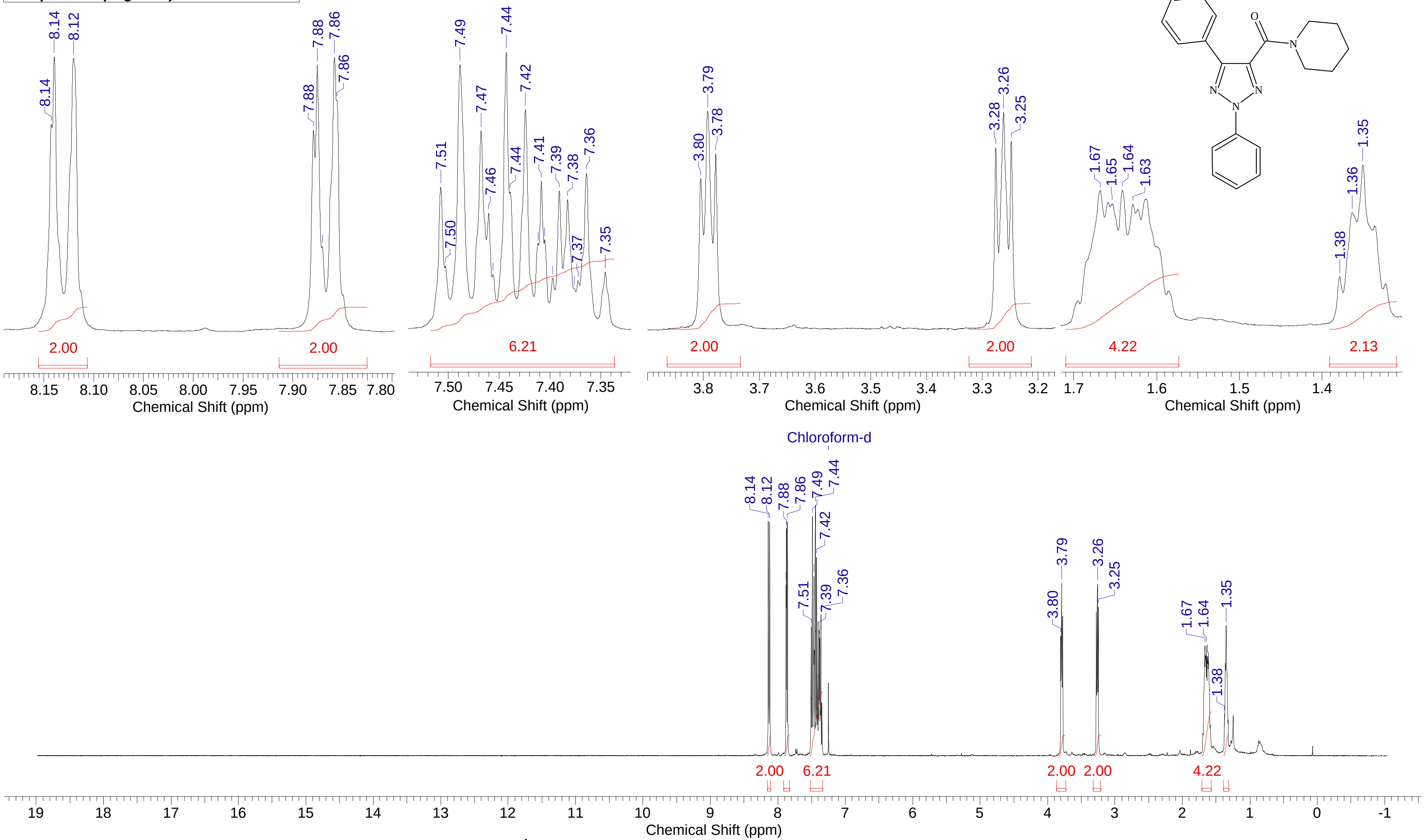

^{1}H NMR spectrum of **22** (400.1 MHz, $CDCl_3$)

5 Jan 2023

Acquisition Time (sec)	0.6783	***Comment***	Imported from UXNMR.			***Date***	20 Dec 2022 14:28:32
File Name	C:\DOCS\OUTPUT_301\2022\12.декабрь\SZA-BM-2706-16.C_002001r					***Frequency (MHz)***	100.61
Nucleus	13C	***Number of Transients***	65	***Original Points Count***	16384	***Points Count***	131072
Pulse Sequence	zgpg30	***Solvent***	DMSO-D6	***Sweep Width (Hz)***	24154.59	***Temperature (degree C)***	27.000

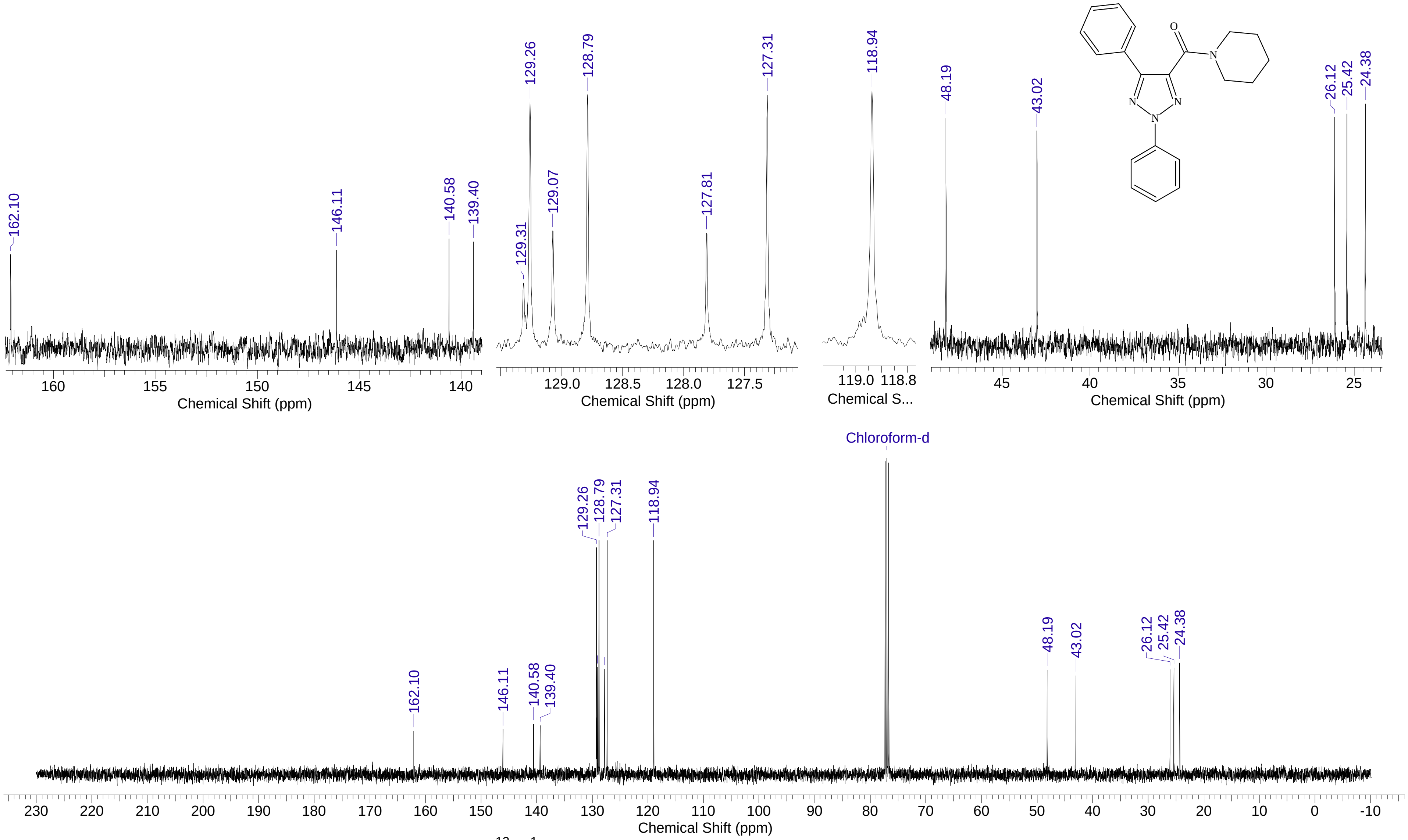

$^{13}C\{^{1}H\}$ NMR spectrum of **22** (100.6 MHz, $CDCl_3$)

5 Jan 2023

Acquisition Time (sec)	4.0894	***Comment***	Imported from UXNMR.	***Date***	20 Dec 2022 22:35:22
File Name	C:\BM_DATA\DOCS\ЯМР_соседи\2022\BM-2707-2\BM-2707-2_001001r			***Frequency (MHz)***	400.13
Nucleus	1H	***Number of Transients***	8 ***Original Points Count*** 32768	***Points Count***	131072
Pulse Sequence	zg30	***Solvent***	CHLOROFORM-D	***Sweep Width (Hz)***	8012.82
Temperature (degree C)	27.000				

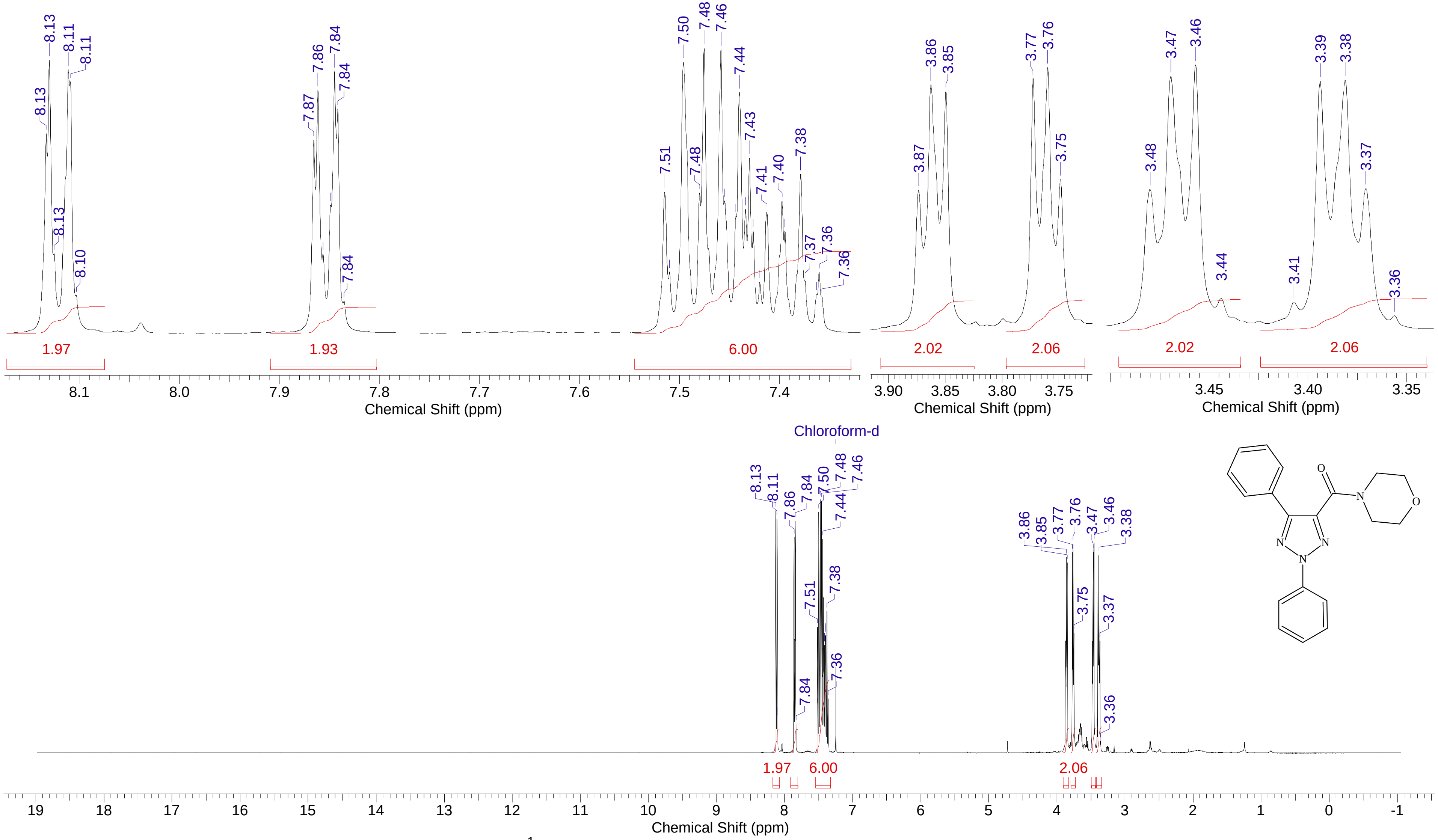

^{1}H NMR spectrum of **23** (400.1 MHz, $CDCl_3$)

5 Jan 2023

Acquisition Time (sec)	0.6783	***Comment***	Imported from UXNMR.			***Date***	20 Dec 2022 21:57:02
File Name	C:\BM_DATA\DOCS\ЯМР_соседи\2022\BM-2707-2\BM-2707-2_002001r					***Frequency (MHz)***	100.61
Nucleus	13C	***Number of Transients***	96	***Original Points Count***	16384	***Points Count***	131072
Pulse Sequence	zgpg30	***Solvent***	CHLOROFORM-D			***Sweep Width (Hz)***	24154.59
Temperature (degree C)	27.000						

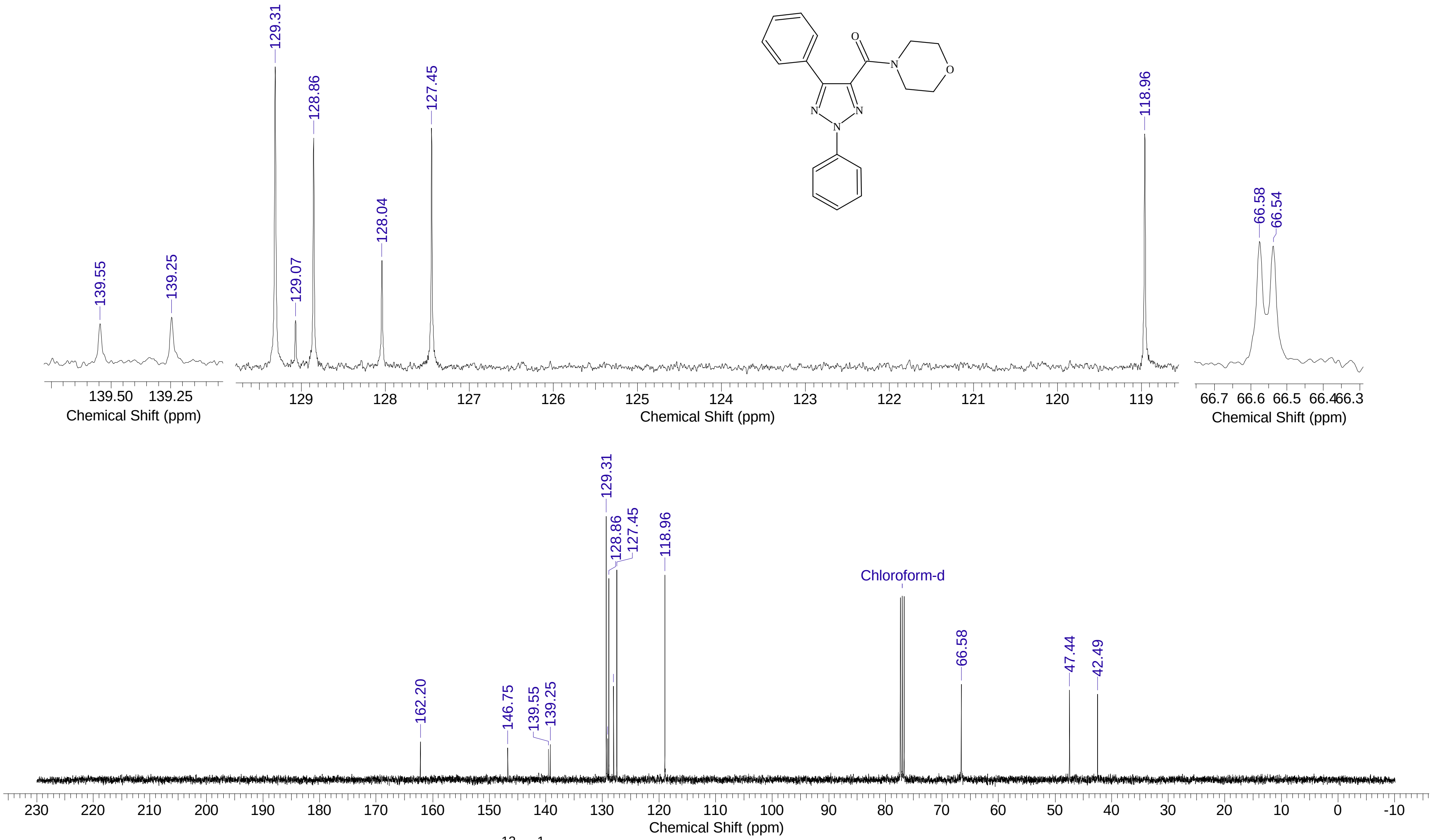

$^{13}C\{^1H\}$ NMR spectrum of **23** (100.6 MHz, $CDCl_3$)

5 Jan 2023

Acquisition Time (sec)	4.0894	***Comment***	Imported from UXNMR.			***Date***	20 Dec 2022 14:19:10
File Name	C:\DOCS\OUTPUT_301\2022\12.декабрь\BM-2708.H_001001r					***Frequency (MHz)***	400.13
Nucleus	1H	***Number of Transients***	4	***Original Points Count***	32768	***Points Count***	131072
Pulse Sequence	zg30	***Solvent***	CHLOROFORM-D			***Sweep Width (Hz)***	8012.82
Temperature (degree C)	27.000						

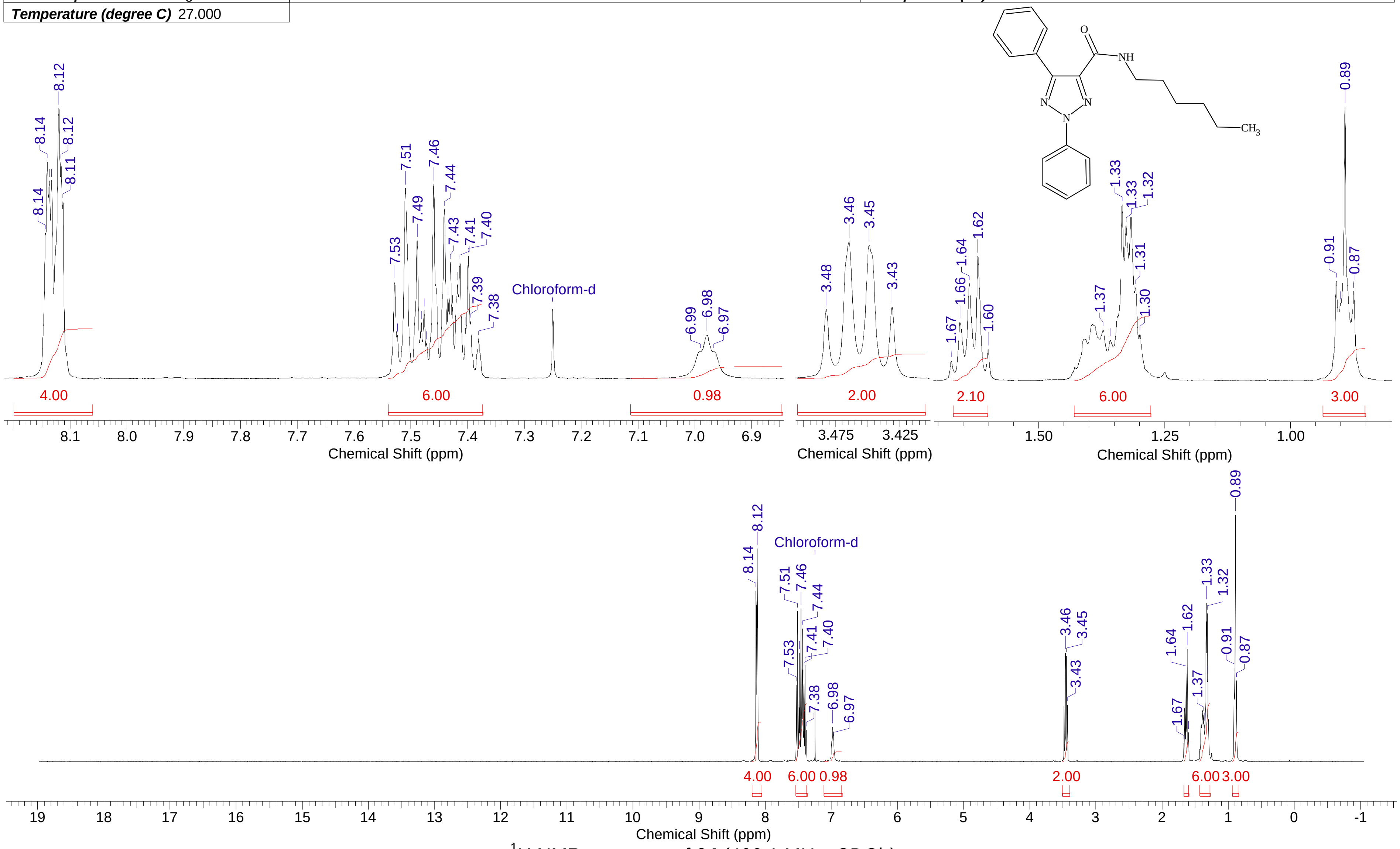

^{1}H NMR spectrum of **24** (400.1 MHz, $CDCl_3$)

5 Jan 2023

Acquisition Time (sec)	0.6783	***Comment***	Imported from UXNMR.			***Date***	20 Dec 2022 14:23:18
File Name	C:\DOCS\OUTPUT_301\2022\12.декабрь\BM-2708.C_002001r					***Frequency (MHz)***	100.61
Nucleus	13C	***Number of Transients***	69	***Original Points Count***	16384	***Points Count***	131072
Pulse Sequence	zgpg30	***Solvent***	DMSO-D6	***Sweep Width (Hz)***	24154.59	***Temperature (degree C)***	27.000

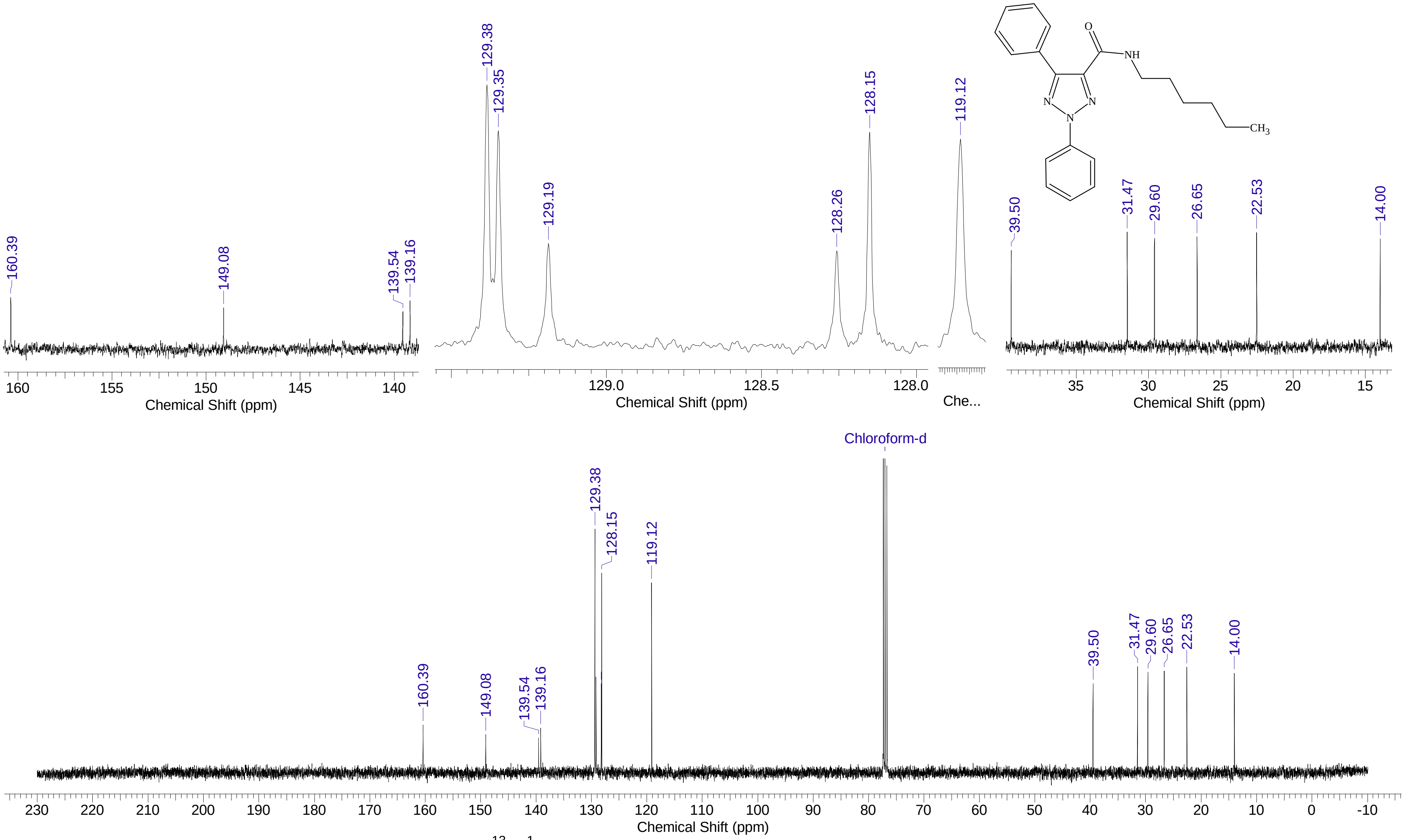

$^{13}C\{^{1}H\}$ NMR spectrum of **24** (100.6 MHz, $CDCl_3$)
